# Supplementary material for: Geographical accessibility to the supply of antiophidic sera in Brazil: Timely access possibilities
Source: PLoS One. 2022 Jan 13;17(1):e0260326. doi: 10.1371/journal.pone.0260326 (PMC8757981; doi:10.1371/journal.pone.0260326)
Supplement: S1 Appendix — (DOCX) [file pone.0260326.s001.docx]

**S1 Appendix: Snakebite accidents, Population, Incidence Rate and Uncovered Population (%) by Brazilian municipalities, 2019**

| Municipality | State | Snakebite Accidents 2019 | Population 2019 | Incidence Rate 2019 | Uncovered Population (%) |
| --- | --- | --- | --- | --- | --- |
| Alto Alegre | RR | 99 | 15.510 | 6,38 | 48,1 |
| Uiramutã | RR | 62 | 10.559 | 5,87 | 46,5 |
| Severiano Melo | RN | 12 | 2.440 | 4,92 | 29,0 |
| Mazagão | AP | 91 | 21.632 | 4,21 | 37,5 |
| Recursolândia | TO | 17 | 4.293 | 3,96 | 13,4 |
| Afuá | PA | 132 | 39.218 | 3,37 | 63,4 |
| Itaubal | AP | 17 | 5.503 | 3,09 | 90,1 |
| Arame | MA | 99 | 32.701 | 3,03 | 17,4 |
| Piraquê | TO | 9 | 3.028 | 2,97 | 25,4 |
| Anajás | PA | 86 | 29.277 | 2,94 | 100,0 |
| Lizarda | TO | 11 | 3.740 | 2,94 | 17,8 |
| Tibau | RN | 12 | 4.106 | 2,92 | 0,0 |
| Jenipapo dos Vieiras | MA | 47 | 16.515 | 2,85 | 17,6 |
| Pedro Teixeira | MG | 5 | 1.807 | 2,77 | 0,0 |
| Japurá | AM | 7 | 2.755 | 2,54 | 92,4 |
| São José do Jacuri | MG | 16 | 6.453 | 2,48 | 2,4 |
| Caapiranga | AM | 32 | 13.081 | 2,45 | 28,6 |
| Jacareacanga | PA | 20 | 8.239 | 2,43 | 47,7 |
| Senador José Porfírio | PA | 28 | 11.658 | 2,4 | 26,5 |
| São Domingos do Capim | PA | 75 | 31.989 | 2,34 | 3,9 |
| Lajes Pintadas | RN | 11 | 4.759 | 2,31 | 0,0 |
| Santa Cruz do Arari | PA | 23 | 10.128 | 2,27 | 10,6 |
| Campos Lindos | TO | 23 | 10.116 | 2,27 | 20,2 |
| Santa Bárbara do Tugúrio | MG | 10 | 4.430 | 2,26 | 0,0 |
| Planalto da Serra | MT | 6 | 2.662 | 2,25 | 8,3 |
| Goiatins | TO | 29 | 13.019 | 2,23 | 13,5 |
| Pau D'Arco | PA | 12 | 5.483 | 2,19 | 4,5 |
| Cutias | AP | 13 | 5.983 | 2,17 | 98,7 |
| Bujaru | PA | 63 | 29.132 | 2,16 | 18,7 |
| Pacaraima | RR | 37 | 17.401 | 2,13 | 28,4 |
| Oeiras do Pará | PA | 69 | 32.512 | 2,12 | 16,7 |
| Portel | PA | 131 | 62.043 | 2,11 | 38,8 |
| Ivolândia | GO | 5 | 2.370 | 2,11 | 7,4 |
| Chapada da Natividade | TO | 7 | 3.333 | 2,1 | 13,6 |
| São Pedro do Suaçuí | MG | 11 | 5.246 | 2,1 | 0,8 |
| Itaúba | MT | 8 | 3.802 | 2,1 | 5,6 |
| Gurupá | PA | 69 | 33.376 | 2,07 | 48,6 |
| São Bento do Tocantins | TO | 11 | 5.324 | 2,07 | 0,6 |
| Ribeirão do Largo | BA | 12 | 5.812 | 2,06 | 10,2 |
| Lamim | MG | 7 | 3.391 | 2,06 | 0,0 |
| Muniz Freire | ES | 36 | 17.465 | 2,06 | 0,0 |
| Maurilândia do Tocantins | TO | 7 | 3.426 | 2,04 | 4,6 |
| Pau Brasil | BA | 20 | 9.831 | 2,03 | 8,9 |
| Chaves | PA | 48 | 23.717 | 2,02 | 83,7 |
| Frei Lagonegro | MG | 7 | 3.478 | 2,01 | 12,3 |
| Santa Maria das Barreiras | PA | 43 | 21.449 | 2 | 43,3 |
| Tocantínia | TO | 15 | 7.545 | 1,99 | 9,4 |
| União do Sul | MT | 7 | 3.525 | 1,99 | 98,8 |
| Pacajá | PA | 94 | 47.706 | 1,97 | 20,5 |
| Dois Irmãos das Missões | RS | 4 | 2.026 | 1,97 | 0,0 |
| Amajari | RR | 25 | 12.796 | 1,95 | 92,3 |
| Goianorte | TO | 10 | 5.123 | 1,95 | 15,0 |
| Algodão de Jandaíra | PB | 5 | 2.567 | 1,95 | 96,6 |
| Ruy Barbosa | RN | 7 | 3.600 | 1,94 | 0,0 |
| São Gabriel da Cachoeira | AM | 88 | 45.564 | 1,93 | 42,1 |
| Nova Esperança do Piriá | PA | 41 | 21.368 | 1,92 | 12,6 |
| Dueré | TO | 9 | 4.686 | 1,92 | 1,9 |
| Itambé do Mato Dentro | MG | 4 | 2.081 | 1,92 | 0,1 |
| São Sebastião da Boa Vista | PA | 51 | 26.640 | 1,91 | 34,2 |
| São Raimundo do Doca Bezerra | MA | 10 | 5.237 | 1,91 | 11,8 |
| Ibitirama | ES | 17 | 8.889 | 1,91 | 0,0 |
| Mâncio Lima | AC | 36 | 18.977 | 1,9 | 13,4 |
| Manoel Urbano | AC | 18 | 9.459 | 1,9 | 9,9 |
| Irituia | PA | 62 | 32.550 | 1,9 | 1,6 |
| Abel Figueiredo | PA | 14 | 7.434 | 1,88 | 0,3 |
| Serra Azul de Minas | MG | 8 | 4.293 | 1,86 | 5,4 |
| Uarini | AM | 25 | 13.540 | 1,85 | 13,1 |
| Acorizal | MT | 10 | 5.399 | 1,85 | 2,0 |
| Pimenteiras do Oeste | RO | 4 | 2.169 | 1,84 | 100,0 |
| Barreirinha | AM | 59 | 32.041 | 1,84 | 33,8 |
| Monte Negro | RO | 29 | 15.852 | 1,83 | 25,3 |
| Barcelos | AM | 50 | 27.502 | 1,82 | 40,5 |
| Jutaí | AM | 26 | 14.317 | 1,82 | 25,8 |
| Brasilândia do Tocantins | TO | 4 | 2.201 | 1,82 | 6,3 |
| Brejinho de Nazaré | TO | 10 | 5.497 | 1,82 | 2,7 |
| Rio dos Índios | RS | 5 | 2.752 | 1,82 | 0,0 |
| Dois Irmãos do Tocantins | TO | 13 | 7.198 | 1,81 | 26,4 |
| Santa Efigênia de Minas | MG | 8 | 4.409 | 1,81 | 18,6 |
| Óbidos | PA | 94 | 52.137 | 1,8 | 16,7 |
| Nazaré | TO | 7 | 3.898 | 1,8 | 0,0 |
| Babaçulândia | TO | 19 | 10.664 | 1,78 | 8,0 |
| São Geraldo da Piedade | MG | 7 | 3.962 | 1,77 | 10,3 |
| Monte Santo do Tocantins | TO | 4 | 2.279 | 1,76 | 16,1 |
| Rio do Prado | MG | 9 | 5.150 | 1,75 | 9,8 |
| Uirapuru | GO | 5 | 2.854 | 1,75 | 15,2 |
| Itaipava do Grajaú | MA | 28 | 16.057 | 1,74 | 10,3 |
| Teresina de Goiás | GO | 6 | 3.458 | 1,74 | 0,5 |
| Santana do Manhuaçu | MG | 15 | 8.674 | 1,73 | 0,0 |
| União da Serra | RS | 2 | 1.154 | 1,73 | 13,2 |
| Centenário | TO | 5 | 2.905 | 1,72 | 24,6 |
| Nhamundá | AM | 36 | 21.173 | 1,7 | 46,9 |
| Nova Olinda do Norte | AM | 63 | 37.378 | 1,69 | 33,7 |
| Sossêgo | PB | 6 | 3.555 | 1,69 | 5,0 |
| Alvarães | AM | 27 | 16.041 | 1,68 | 18,8 |
| Aparecida do Rio Negro | TO | 8 | 4.795 | 1,67 | 0,8 |
| Virgolândia | MG | 9 | 5.380 | 1,67 | 0,2 |
| Itamarati | AM | 13 | 7.851 | 1,66 | 24,1 |
| Curralinho | PA | 57 | 34.448 | 1,65 | 50,0 |
| Amapá | AP | 15 | 9.109 | 1,65 | 15,4 |
| Fernando Pedroza | RN | 5 | 3.054 | 1,64 | 100,0 |
| Santa Luzia do Norte | AL | 12 | 7.296 | 1,64 | 0,0 |
| Boa Vista do Ramos | AM | 31 | 19.207 | 1,61 | 21,8 |
| Prainha | PA | 48 | 29.866 | 1,61 | 18,5 |
| Itacajá | TO | 12 | 7.433 | 1,61 | 25,2 |
| Breves | PA | 164 | 102.701 | 1,6 | 41,7 |
| Perolândia | GO | 5 | 3.129 | 1,6 | 14,1 |
| Dom Pedro de Alcântara | RS | 4 | 2.534 | 1,58 | 0,0 |
| Bagre | PA | 48 | 30.673 | 1,56 | 34,6 |
| Concórdia do Pará | PA | 52 | 33.318 | 1,56 | 0,8 |
| Tomé-Açu | PA | 99 | 63.447 | 1,56 | 8,9 |
| Santa Cruz do Xingu | MT | 4 | 2.564 | 1,56 | 100,0 |
| Melgaço | PA | 43 | 27.654 | 1,55 | 59,9 |
| Irati | SC | 3 | 1.930 | 1,55 | 0,0 |
| Paranhos | MS | 22 | 14.228 | 1,55 | 7,7 |
| Santa Terezinha | MT | 13 | 8.371 | 1,55 | 99,6 |
| Ferreira Gomes | AP | 12 | 7.780 | 1,54 | 1,0 |
| Borba | AM | 63 | 41.161 | 1,53 | 38,1 |
| Barra do Ouro | TO | 7 | 4.591 | 1,52 | 4,8 |
| Bom Jesus do Araguaia | MT | 10 | 6.580 | 1,52 | 6,3 |
| Faina | GO | 10 | 6.627 | 1,51 | 1,1 |
| Porto Walter | AC | 18 | 11.982 | 1,5 | 100,0 |
| Veríssimo | MG | 6 | 3.999 | 1,5 | 2,3 |
| Peixe-Boi | PA | 12 | 8.077 | 1,49 | 0,0 |
| Ipueiras | TO | 3 | 2.015 | 1,49 | 0,3 |
| Lagoa Grande do Maranhão | MA | 17 | 11.394 | 1,49 | 15,9 |
| Santo Antônio do Planalto | RS | 3 | 2.019 | 1,49 | 0,0 |
| Floresta do Araguaia | PA | 30 | 20.304 | 1,48 | 22,4 |
| Sandolândia | TO | 5 | 3.375 | 1,48 | 13,8 |
| Colinas do Sul | GO | 5 | 3.382 | 1,48 | 1,7 |
| São Valério do Sul | RS | 4 | 2.727 | 1,47 | 0,0 |
| Garrafão do Norte | PA | 38 | 26.066 | 1,46 | 0,9 |
| Oriximiná | PA | 107 | 73.096 | 1,46 | 17,8 |
| Junco do Maranhão | MA | 5 | 3.432 | 1,46 | 100,0 |
| Bandeira | MG | 7 | 4.795 | 1,46 | 4,1 |
| Braúnas | MG | 7 | 4.801 | 1,46 | 19,6 |
| Bonfim | RR | 18 | 12.409 | 1,45 | 49,9 |
| Tanque do Piauí | PI | 4 | 2.765 | 1,45 | 1,2 |
| São João da Paraúna | GO | 2 | 1.381 | 1,45 | 0,0 |
| Buriticupu | MA | 104 | 72.358 | 1,44 | 13,2 |
| Luisburgo | MG | 9 | 6.266 | 1,44 | 0,0 |
| Alegria | RS | 5 | 3.464 | 1,44 | 0,0 |
| Luciara | MT | 3 | 2.077 | 1,44 | 100,0 |
| Rodrigues Alves | AC | 27 | 18.930 | 1,43 | 10,1 |
| Monte das Gameleiras | RN | 3 | 2.105 | 1,43 | 1,6 |
| Chapada de Areia | TO | 2 | 1.406 | 1,42 | 80,7 |
| Nova Ubiratã | MT | 17 | 11.982 | 1,42 | 100,0 |
| Bandeirantes do Tocantins | TO | 5 | 3.553 | 1,41 | 4,8 |
| Rio dos Bois | TO | 4 | 2.834 | 1,41 | 5,8 |
| Brejo de Areia | MA | 13 | 9.188 | 1,41 | 0,8 |
| Grajaú | MA | 98 | 69.527 | 1,41 | 9,5 |
| São Paulo de Olivença | AM | 55 | 39.299 | 1,4 | 85,5 |
| Santa Tereza do Tocantins | TO | 4 | 2.866 | 1,4 | 0,0 |
| Alto Rio Novo | ES | 11 | 7.836 | 1,4 | 0,0 |
| Morretes | PR | 23 | 16.406 | 1,4 | 0,0 |
| Acará | PA | 77 | 55.591 | 1,39 | 11,9 |
| Lagoa do Tocantins | TO | 6 | 4.316 | 1,39 | 3,0 |
| Palmeira do Piauí | PI | 7 | 5.043 | 1,39 | 29,7 |
| União de Minas | MG | 6 | 4.304 | 1,39 | 14,3 |
| Mocajuba | PA | 43 | 31.136 | 1,38 | 6,6 |
| Novo Acordo | TO | 6 | 4.342 | 1,38 | 4,9 |
| Amarante do Maranhão | MA | 57 | 41.435 | 1,38 | 29,2 |
| Altamira do Maranhão | MA | 11 | 8.128 | 1,35 | 5,4 |
| Iracema | RR | 16 | 11.950 | 1,34 | 13,0 |
| Santarém Novo | PA | 9 | 6.709 | 1,34 | 0,0 |
| Jitaúna | BA | 15 | 11.166 | 1,34 | 4,3 |
| São João do Itaperiú | SC | 5 | 3.733 | 1,34 | 0,0 |
| Brasil Novo | PA | 20 | 15.086 | 1,33 | 30,5 |
| Tartarugalzinho | AP | 23 | 17.315 | 1,33 | 33,7 |
| Caatiba | BA | 9 | 6.758 | 1,33 | 2,9 |
| Marcelândia | MT | 14 | 10.499 | 1,33 | 6,4 |
| Anamã | AM | 18 | 13.614 | 1,32 | 81,0 |
| Vitória do Xingu | PA | 20 | 15.134 | 1,32 | 2,3 |
| Ouro Velho | PB | 4 | 3.039 | 1,32 | 100,0 |
| Medeiros | MG | 5 | 3.802 | 1,32 | 2,2 |
| Conceição de Ipanema | MG | 6 | 4.574 | 1,31 | 4,8 |
| Paiva | MG | 2 | 1.529 | 1,31 | 0,0 |
| Peçanha | MG | 23 | 17.541 | 1,31 | 10,8 |
| Santa Leopoldina | ES | 16 | 12.224 | 1,31 | 0,0 |
| Barra do Turvo | SP | 10 | 7.659 | 1,31 | 1,9 |
| Presidente Nereu | SC | 3 | 2.287 | 1,31 | 0,2 |
| Santa Isabel | GO | 5 | 3.809 | 1,31 | 0,6 |
| Moju | PA | 107 | 82.094 | 1,3 | 24,0 |
| Itagibá | BA | 19 | 14.579 | 1,3 | 0,9 |
| Coroaci | MG | 13 | 9.991 | 1,3 | 8,0 |
| Santo Antônio do Itambé | MG | 5 | 3.838 | 1,3 | 0,0 |
| Mucajaí | RR | 23 | 17.853 | 1,29 | 3,0 |
| São João do Araguaia | PA | 18 | 13.996 | 1,29 | 8,6 |
| São Pedro da União | MG | 6 | 4.659 | 1,29 | 0,0 |
| Itacoatiara | AM | 130 | 101.337 | 1,28 | 19,6 |
| Novo Repartimento | PA | 97 | 75.919 | 1,28 | 32,1 |
| Lajeado | TO | 4 | 3.134 | 1,28 | 7,8 |
| Marajá do Sena | MA | 10 | 7.792 | 1,28 | 54,7 |
| Santa Rita do Itueto | MG | 7 | 5.489 | 1,28 | 0,4 |
| Maués | AM | 81 | 63.905 | 1,27 | 39,2 |
| Araguacema | TO | 9 | 7.086 | 1,27 | 4,8 |
| Itapebi | BA | 13 | 10.259 | 1,27 | 0,8 |
| Açucena | MG | 12 | 9.470 | 1,27 | 25,9 |
| Iúna | ES | 37 | 29.161 | 1,27 | 0,0 |
| Feliz Natal | MT | 18 | 14.192 | 1,27 | 100,0 |
| Porto de Moz | PA | 52 | 41.135 | 1,26 | 34,0 |
| São Francisco do Pará | PA | 20 | 15.882 | 1,26 | 0,0 |
| Calçoene | AP | 14 | 11.117 | 1,26 | 98,6 |
| Ananás | TO | 12 | 9.549 | 1,26 | 0,8 |
| São Félix do Tocantins | TO | 2 | 1.585 | 1,26 | 82,7 |
| General Carneiro | MT | 7 | 5.540 | 1,26 | 55,5 |
| Potiraguá | BA | 9 | 7.224 | 1,25 | 0,2 |
| Rio Vermelho | MG | 16 | 12.846 | 1,25 | 12,9 |
| Campinápolis | MT | 20 | 15.980 | 1,25 | 42,6 |
| Divinópolis de Goiás | GO | 6 | 4.804 | 1,25 | 0,9 |
| Belterra | PA | 22 | 17.732 | 1,24 | 21,8 |
| Itaporã do Tocantins | TO | 3 | 2.427 | 1,24 | 8,1 |
| Passagem | PB | 3 | 2.419 | 1,24 | 99,6 |
| Itati | RS | 3 | 2.419 | 1,24 | 0,1 |
| Cavalcante | GO | 12 | 9.709 | 1,24 | 31,2 |
| São Miguel do Passa Quatro | GO | 5 | 4.057 | 1,23 | 0,0 |
| Beruri | AM | 24 | 19.679 | 1,22 | 28,6 |
| Pedra Preta | RN | 3 | 2.458 | 1,22 | 5,0 |
| Monte Formoso | MG | 6 | 4.906 | 1,22 | 2,1 |
| Tocos do Moji | MG | 5 | 4.101 | 1,22 | 0,0 |
| Gentil | RS | 2 | 1.634 | 1,22 | 0,0 |
| Muaná | PA | 49 | 40.349 | 1,21 | 43,4 |
| Trairão | PA | 23 | 18.989 | 1,21 | 3,8 |
| Mato Queimado | RS | 2 | 1.648 | 1,21 | 0,0 |
| Nossa Senhora do Livramento | MT | 16 | 13.216 | 1,21 | 19,5 |
| Nova Ipixuna | PA | 20 | 16.678 | 1,2 | 23,9 |
| Tufilândia | MA | 7 | 5.840 | 1,2 | 85,7 |
| Itaju do Colônia | BA | 8 | 6.682 | 1,2 | 1,1 |
| José Raydan | MG | 6 | 4.995 | 1,2 | 2,4 |
| Senador Modestino Gonçalves | MG | 5 | 4.156 | 1,2 | 32,4 |
| José Boiteux | SC | 6 | 4.997 | 1,2 | 4,3 |
| São Roberto | MA | 8 | 6.719 | 1,19 | 26,4 |
| Dom Joaquim | MG | 5 | 4.195 | 1,19 | 20,4 |
| Cássia dos Coqueiros | SP | 3 | 2.523 | 1,19 | 0,0 |
| Barra Bonita | SC | 2 | 1.677 | 1,19 | 0,0 |
| Pium | TO | 9 | 7.654 | 1,18 | 21,8 |
| Fervedouro | MG | 13 | 11.006 | 1,18 | 0,0 |
| Varre-Sai | RJ | 13 | 11.000 | 1,18 | 0,0 |
| Alto Paraíso de Goiás | GO | 9 | 7.624 | 1,18 | 0,3 |
| Taboleiro Grande | RN | 3 | 2.566 | 1,17 | 99,4 |
| Jaguaripe | BA | 22 | 18.788 | 1,17 | 0,1 |
| Vargem Alta | ES | 25 | 21.402 | 1,17 | 0,0 |
| Santa Rita do Trivelato | MT | 4 | 3.429 | 1,17 | 100,0 |
| Baliza | GO | 6 | 5.138 | 1,17 | 22,7 |
| Plácido de Castro | AC | 23 | 19.761 | 1,16 | 6,0 |
| Colares | PA | 14 | 12.085 | 1,16 | 0,0 |
| Palmeirante | TO | 7 | 6.026 | 1,16 | 14,8 |
| Mirante | BA | 10 | 8.641 | 1,16 | 32,4 |
| Novo Xingu | RS | 2 | 1.719 | 1,16 | 0,0 |
| São João d'Aliança | GO | 16 | 13.740 | 1,16 | 4,1 |
| Anapu | PA | 32 | 27.890 | 1,15 | 8,1 |
| Simão Pereira | MG | 3 | 2.615 | 1,15 | 0,0 |
| Umburatiba | MG | 3 | 2.611 | 1,15 | 4,4 |
| Autazes | AM | 45 | 39.565 | 1,14 | 21,5 |
| Fonte Boa | AM | 20 | 17.609 | 1,14 | 22,9 |
| Formoso do Araguaia | TO | 21 | 18.440 | 1,14 | 6,6 |
| Olaria | MG | 2 | 1.747 | 1,14 | 0,0 |
| Bodoquena | MS | 9 | 7.875 | 1,14 | 5,0 |
| Figueirópolis D'Oeste | MT | 4 | 3.494 | 1,14 | 19,6 |
| Campo Novo de Rondônia | RO | 16 | 14.139 | 1,13 | 82,7 |
| Amaturá | AM | 13 | 11.536 | 1,13 | 5,5 |
| Monte do Carmo | TO | 9 | 7.947 | 1,13 | 11,3 |
| Pedra Bonita | MG | 8 | 7.097 | 1,13 | 0,0 |
| Floriano Peixoto | RS | 2 | 1.766 | 1,13 | 35,7 |
| Mateiros | TO | 3 | 2.684 | 1,12 | 6,8 |
| São Bento do Trairí | RN | 5 | 4.449 | 1,12 | 0,5 |
| Cacimbas | PB | 8 | 7.173 | 1,12 | 100,0 |
| Antônio João | MS | 10 | 8.956 | 1,12 | 1,7 |
| Rio Preto da Eva | AM | 37 | 33.347 | 1,11 | 4,4 |
| Santo Antônio do Içá | AM | 24 | 21.602 | 1,11 | 88,0 |
| Bonito | PA | 18 | 16.286 | 1,11 | 0,0 |
| Inhangapi | PA | 13 | 11.711 | 1,11 | 0,0 |
| Choró | CE | 15 | 13.521 | 1,11 | 4,3 |
| Catuji | MG | 7 | 6.311 | 1,11 | 0,9 |
| São José do Goiabal | MG | 6 | 5.420 | 1,11 | 0,0 |
| Sardoá | MG | 7 | 6.300 | 1,11 | 8,8 |
| Três Forquilhas | RS | 3 | 2.697 | 1,11 | 0,0 |
| Torixoréu | MT | 4 | 3.609 | 1,11 | 8,3 |
| Guarinos | GO | 2 | 1.794 | 1,11 | 0,8 |
| Jucuruçu | BA | 10 | 9.126 | 1,1 | 8,5 |
| Campo Florido | MG | 9 | 8.151 | 1,1 | 6,3 |
| Cantagalo | MG | 5 | 4.525 | 1,1 | 0,0 |
| Aspásia | SP | 2 | 1.822 | 1,1 | 0,0 |
| Cantá | RR | 20 | 18.335 | 1,09 | 29,5 |
| Lima Campos | MA | 13 | 11.893 | 1,09 | 1,0 |
| São Félix de Balsas | MA | 5 | 4.585 | 1,09 | 16,4 |
| Caiçara do Rio do Vento | RN | 4 | 3.684 | 1,09 | 1,1 |
| Messias Targino | RN | 5 | 4.601 | 1,09 | 6,8 |
| São Tomé | RN | 12 | 11.055 | 1,09 | 4,0 |
| Sericita | MG | 8 | 7.326 | 1,09 | 0,0 |
| Santa Luzia D'Oeste | RO | 7 | 6.495 | 1,08 | 2,5 |
| Guajará | AM | 18 | 16.678 | 1,08 | 24,4 |
| Caroebe | RR | 11 | 10.169 | 1,08 | 5,4 |
| Almeirim | PA | 37 | 34.109 | 1,08 | 17,9 |
| Aurora do Pará | PA | 34 | 31.338 | 1,08 | 26,5 |
| Itapiúna | CE | 22 | 20.382 | 1,08 | 5,3 |
| Ingaí | MG | 3 | 2.767 | 1,08 | 0,0 |
| Boa Vista do Sul | RS | 3 | 2.783 | 1,08 | 0,0 |
| Vila Rica | MT | 28 | 26.037 | 1,08 | 100,0 |
| Talismã | TO | 3 | 2.793 | 1,07 | 3,0 |
| Presidente Vargas | MA | 12 | 11.193 | 1,07 | 0,0 |
| Diamante | PB | 7 | 6.552 | 1,07 | 24,9 |
| Barro Preto | BA | 6 | 5.591 | 1,07 | 0,0 |
| Matina | BA | 13 | 12.201 | 1,07 | 0,9 |
| Paraty | RJ | 46 | 43.165 | 1,07 | 1,3 |
| Araguainha | MT | 1 | 935 | 1,07 | 5,0 |
| São Domingos do Araguaia | PA | 27 | 25.557 | 1,06 | 4,3 |
| Itapiratins | TO | 4 | 3.777 | 1,06 | 35,5 |
| Apiacá | ES | 8 | 7.567 | 1,06 | 0,0 |
| Primavera de Rondônia | RO | 3 | 2.856 | 1,05 | 11,0 |
| Humaitá | AM | 58 | 55.080 | 1,05 | 19,5 |
| Tapauá | AM | 18 | 17.156 | 1,05 | 35,3 |
| Urucará | AM | 17 | 16.256 | 1,05 | 18,8 |
| Chalé | MG | 6 | 5.704 | 1,05 | 0,1 |
| Irupi | ES | 14 | 13.377 | 1,05 | 0,0 |
| Canabrava do Norte | MT | 5 | 4.743 | 1,05 | 100,0 |
| Gameleira de Goiás | GO | 4 | 3.818 | 1,05 | 1,1 |
| Aveiro | PA | 17 | 16.388 | 1,04 | 38,4 |
| Santa Bárbara do Pará | PA | 22 | 21.079 | 1,04 | 0,0 |
| Aragominas | TO | 6 | 5.758 | 1,04 | 50,1 |
| Arraias | TO | 11 | 10.567 | 1,04 | 13,9 |
| Lavandeira | TO | 2 | 1.923 | 1,04 | 0,5 |
| Miracema do Tocantins | TO | 19 | 18.248 | 1,04 | 1,4 |
| Abre Campo | MG | 14 | 13.454 | 1,04 | 0,0 |
| Placas | PA | 32 | 30.982 | 1,03 | 20,6 |
| Pedra Branca do Amapari | AP | 17 | 16.502 | 1,03 | 11,6 |
| Riacho de Santo Antônio | PB | 2 | 1.948 | 1,03 | 15,7 |
| Jussari | BA | 6 | 5.833 | 1,03 | 0,0 |
| Alto Caparaó | MG | 6 | 5.847 | 1,03 | 0,0 |
| Altamira do Paraná | PR | 2 | 1.942 | 1,03 | 0,0 |
| Nova Olinda | TO | 12 | 11.819 | 1,02 | 7,8 |
| Peixe | TO | 12 | 11.749 | 1,02 | 16,6 |
| Sete de Setembro | RS | 2 | 1.970 | 1,02 | 0,0 |
| Novo Aripuanã | AM | 26 | 25.644 | 1,01 | 20,2 |
| Terra Santa | PA | 19 | 18.769 | 1,01 | 7,9 |
| Divinópolis do Tocantins | TO | 7 | 6.900 | 1,01 | 3,9 |
| Capinzal do Norte | MA | 11 | 10.934 | 1,01 | 1,3 |
| São João do Carú | MA | 16 | 15.808 | 1,01 | 100,0 |
| Imbé de Minas | MG | 7 | 6.903 | 1,01 | 0,2 |
| Porto Estrela | MT | 3 | 2.963 | 1,01 | 83,1 |
| Cruzeiro do Sul | AC | 88 | 88.376 | 1 | 5,1 |
| Envira | AM | 20 | 20.033 | 1 | 20,9 |
| Tefé | AM | 60 | 59.849 | 1 | 10,9 |
| Urucurituba | AM | 23 | 23.065 | 1 | 30,9 |
| Vitória do Jari | AP | 16 | 15.931 | 1 | 13,2 |
| Ponte Alta do Tocantins | TO | 8 | 8.039 | 1 | 6,6 |
| Arataca | BA | 11 | 11.019 | 1 | 0,1 |
| Mutum | MG | 27 | 26.979 | 1 | 8,9 |
| Barra do Corda | MA | 87 | 88.212 | 0,99 | 11,7 |
| Carolina | MA | 24 | 24.322 | 0,99 | 15,1 |
| Pau D'Arco do Piauí | PI | 4 | 4.045 | 0,99 | 2,5 |
| Itueta | MG | 6 | 6.051 | 0,99 | 0,0 |
| Santa Margarida | MG | 16 | 16.208 | 0,99 | 0,0 |
| Conquista D'Oeste | MT | 4 | 4.038 | 0,99 | 96,6 |
| Novo São Joaquim | MT | 5 | 5.074 | 0,99 | 12,8 |
| Benjamin Constant | AM | 42 | 42.984 | 0,98 | 16,8 |
| Silves | AM | 9 | 9.171 | 0,98 | 4,8 |
| Tailândia | PA | 104 | 106.339 | 0,98 | 5,0 |
| Mantenópolis | ES | 15 | 15.350 | 0,98 | 0,0 |
| Vila Pavão | ES | 9 | 9.208 | 0,98 | 0,0 |
| Colniza | MT | 38 | 38.582 | 0,98 | 15,9 |
| Nobres | MT | 15 | 15.336 | 0,98 | 2,8 |
| São José do Povo | MT | 4 | 4.063 | 0,98 | 100,0 |
| Vera Mendes | PI | 3 | 3.077 | 0,97 | 24,0 |
| Afrânio | PE | 19 | 19.635 | 0,97 | 26,2 |
| Santo Hipólito | MG | 3 | 3.087 | 0,97 | 7,6 |
| Engenho Velho | RS | 1 | 1.034 | 0,97 | 0,0 |
| Araguaiana | MT | 3 | 3.100 | 0,97 | 20,8 |
| Gado Bravo | PB | 8 | 8.316 | 0,96 | 54,6 |
| Jundiá | AL | 4 | 4.155 | 0,96 | 100,0 |
| Gramado dos Loureiros | RS | 2 | 2.082 | 0,96 | 0,0 |
| Porto Alegre do Norte | MT | 12 | 12.517 | 0,96 | 100,0 |
| Manicoré | AM | 53 | 55.751 | 0,95 | 49,4 |
| Parazinho | RN | 5 | 5.237 | 0,95 | 0,0 |
| Iporanga | SP | 4 | 4.218 | 0,95 | 1,0 |
| Laguna Carapã | MS | 7 | 7.341 | 0,95 | 2,1 |
| Assis Brasil | AC | 7 | 7.417 | 0,94 | 12,6 |
| Breu Branco | PA | 62 | 66.046 | 0,94 | 9,1 |
| Magalhães Barata | PA | 8 | 8.548 | 0,94 | 0,0 |
| Nova Rosalândia | TO | 4 | 4.260 | 0,94 | 0,0 |
| Wanderlândia | TO | 11 | 11.683 | 0,94 | 0,6 |
| Riachão | MA | 19 | 20.195 | 0,94 | 15,4 |
| Areia de Baraúnas | PB | 2 | 2.128 | 0,94 | 100,0 |
| Nova Belém | MG | 3 | 3.190 | 0,94 | 0,0 |
| São José da Safira | MG | 4 | 4.268 | 0,94 | 0,0 |
| Conceição do Castelo | ES | 12 | 12.723 | 0,94 | 0,0 |
| Bozano | RS | 2 | 2.123 | 0,94 | 0,0 |
| Confresa | MT | 29 | 30.933 | 0,94 | 100,0 |
| Novo Airão | AM | 18 | 19.454 | 0,93 | 19,4 |
| Pauini | AM | 18 | 19.426 | 0,93 | 36,6 |
| Mojuí dos Campos | PA | 15 | 16.084 | 0,93 | 27,3 |
| Aliança do Tocantins | TO | 5 | 5.390 | 0,93 | 0,6 |
| Rio Sono | TO | 6 | 6.478 | 0,93 | 18,7 |
| Triunfo Potiguar | RN | 3 | 3.237 | 0,93 | 26,4 |
| Dário Meira | BA | 10 | 10.710 | 0,93 | 7,9 |
| Itajuípe | BA | 19 | 20.491 | 0,93 | 0,1 |
| Entre Folhas | MG | 5 | 5.370 | 0,93 | 0,0 |
| Jequitaí | MG | 7 | 7.531 | 0,93 | 13,7 |
| Divino de São Lourenço | ES | 4 | 4.304 | 0,93 | 0,0 |
| Coronel Domingos Soares | PR | 7 | 7.497 | 0,93 | 11,8 |
| Paranatinga | MT | 21 | 22.563 | 0,93 | 100,0 |
| Itabela | BA | 28 | 30.584 | 0,92 | 1,6 |
| Jordânia | MG | 10 | 10.812 | 0,92 | 13,8 |
| Nova Marilândia | MT | 3 | 3.278 | 0,92 | 17,2 |
| Nova Roma | GO | 3 | 3.264 | 0,92 | 1,0 |
| Vicentinópolis | GO | 8 | 8.743 | 0,92 | 1,8 |
| Brasiléia | AC | 24 | 26.278 | 0,91 | 8,0 |
| Eirunepé | AM | 32 | 35.273 | 0,91 | 22,6 |
| Santa Isabel do Rio Negro | AM | 23 | 25.156 | 0,91 | 45,6 |
| Igarapé-Miri | PA | 57 | 62.698 | 0,91 | 12,0 |
| Porto Grande | AP | 20 | 21.971 | 0,91 | 5,8 |
| Peritoró | MA | 21 | 23.196 | 0,91 | 0,7 |
| Coronel Ezequiel | RN | 5 | 5.506 | 0,91 | 5,2 |
| Sítio Novo | RN | 5 | 5.522 | 0,91 | 0,0 |
| Macarani | BA | 17 | 18.755 | 0,91 | 0,9 |
| Caiana | MG | 5 | 5.496 | 0,91 | 0,0 |
| São João do Pacuí | MG | 4 | 4.419 | 0,91 | 3,0 |
| Mucurici | ES | 5 | 5.524 | 0,91 | 0,0 |
| Charrua | RS | 3 | 3.279 | 0,91 | 9,5 |
| Cláudia | MT | 11 | 12.149 | 0,91 | 16,2 |
| Conceição do Araguaia | PA | 43 | 47.864 | 0,9 | 16,2 |
| Limoeiro do Ajuru | PA | 26 | 28.935 | 0,9 | 31,4 |
| Oliveira de Fátima | TO | 1 | 1.112 | 0,9 | 0,0 |
| Pindorama do Tocantins | TO | 4 | 4.447 | 0,9 | 0,6 |
| Roteiro | AL | 6 | 6.664 | 0,9 | 0,0 |
| Araças | BA | 11 | 12.177 | 0,9 | 0,0 |
| Presidente Tancredo Neves | BA | 25 | 27.719 | 0,9 | 3,3 |
| Materlândia | MG | 4 | 4.459 | 0,9 | 13,9 |
| Monjolos | MG | 2 | 2.220 | 0,9 | 5,3 |
| Santa Rosa da Serra | MG | 3 | 3.350 | 0,9 | 0,0 |
| São Sebastião do Maranhão | MG | 9 | 10.044 | 0,9 | 7,6 |
| Ribeira | SP | 3 | 3.340 | 0,9 | 0,0 |
| Normandia | RR | 10 | 11.290 | 0,89 | 36,7 |
| Novo Progresso | PA | 23 | 25.762 | 0,89 | 8,7 |
| Couto Magalhães | TO | 5 | 5.588 | 0,89 | 9,2 |
| Pedro Avelino | RN | 6 | 6.716 | 0,89 | 68,5 |
| Matinhas | PB | 4 | 4.500 | 0,89 | 4,7 |
| Zabelê | PB | 2 | 2.240 | 0,89 | 1,1 |
| Alagoinha | PE | 13 | 14.636 | 0,89 | 0,0 |
| Durandé | MG | 7 | 7.841 | 0,89 | 0,0 |
| São João Evangelista | MG | 14 | 15.774 | 0,89 | 0,9 |
| Brejetuba | ES | 11 | 12.404 | 0,89 | 0,0 |
| Nova Ramada | RS | 2 | 2.242 | 0,89 | 0,0 |
| Salto do Céu | MT | 3 | 3.365 | 0,89 | 15,9 |
| Xapuri | AC | 17 | 19.323 | 0,88 | 18,3 |
| Juruti | PA | 51 | 57.943 | 0,88 | 12,3 |
| Sucupira do Riachão | MA | 5 | 5.660 | 0,88 | 22,0 |
| Gurjão | PB | 3 | 3.428 | 0,88 | 100,0 |
| Botuporã | BA | 9 | 10.211 | 0,88 | 0,0 |
| Ponta de Pedras | PA | 27 | 31.082 | 0,87 | 26,7 |
| Angico | TO | 3 | 3.433 | 0,87 | 0,0 |
| Santa Maria do Tocantins | TO | 3 | 3.434 | 0,87 | 11,3 |
| Simonésia | MG | 17 | 19.633 | 0,87 | 0,0 |
| Nova Laranjeiras | PR | 10 | 11.554 | 0,87 | 6,8 |
| Paraíso | SC | 3 | 3.437 | 0,87 | 0,0 |
| Turuçu | RS | 3 | 3.438 | 0,87 | 0,0 |
| Água Fria de Goiás | GO | 5 | 5.735 | 0,87 | 1,0 |
| Britânia | GO | 5 | 5.779 | 0,87 | 0,8 |
| Itupiranga | PA | 46 | 53.269 | 0,86 | 27,3 |
| Santa Luzia do Pará | PA | 17 | 19.848 | 0,86 | 9,6 |
| Riachinho | TO | 4 | 4.645 | 0,86 | 0,3 |
| Aroazes | PI | 5 | 5.835 | 0,86 | 7,3 |
| Jati | CE | 7 | 8.111 | 0,86 | 6,2 |
| São Sebastião do Umbuzeiro | PB | 3 | 3.489 | 0,86 | 15,2 |
| Caputira | MG | 8 | 9.298 | 0,86 | 0,0 |
| Cedro do Abaeté | MG | 1 | 1.164 | 0,86 | 1,4 |
| Sete Barras | SP | 11 | 12.832 | 0,86 | 0,0 |
| Riozinho | RS | 4 | 4.653 | 0,86 | 0,0 |
| Iaciara | GO | 12 | 13.947 | 0,86 | 2,3 |
| Santa Rosa de Goiás | GO | 2 | 2.319 | 0,86 | 0,0 |
| Vila Propício | GO | 5 | 5.821 | 0,86 | 1,5 |
| Capixaba | AC | 10 | 11.733 | 0,85 | 37,1 |
| Boca do Acre | AM | 29 | 34.308 | 0,85 | 16,2 |
| São João da Baliza | RR | 7 | 8.201 | 0,85 | 1,9 |
| Igarapé do Meio | MA | 12 | 14.177 | 0,85 | 9,5 |
| Floresta Azul | BA | 9 | 10.629 | 0,85 | 3,1 |
| Senhora do Porto | MG | 3 | 3.523 | 0,85 | 4,7 |
| Riachão | PB | 3 | 3.588 | 0,84 | 2,8 |
| Santana do Mundaú | AL | 9 | 10.740 | 0,84 | 3,8 |
| Mairipotaba | GO | 2 | 2.368 | 0,84 | 0,0 |
| Palminópolis | GO | 3 | 3.585 | 0,84 | 0,0 |
| Espigão D'Oeste | RO | 27 | 32.374 | 0,83 | 20,3 |
| Faro | PA | 6 | 7.194 | 0,83 | 6,4 |
| Croatá | CE | 15 | 18.063 | 0,83 | 89,8 |
| Guamaré | RN | 13 | 15.659 | 0,83 | 1,7 |
| Pacatuba | SE | 12 | 14.428 | 0,83 | 0,0 |
| Mulungu do Morro | BA | 9 | 10.889 | 0,83 | 4,3 |
| Pedrinópolis | MG | 3 | 3.635 | 0,83 | 0,2 |
| Santana do Jacaré | MG | 4 | 4.821 | 0,83 | 0,0 |
| Porto Esperidião | MT | 10 | 12.017 | 0,83 | 46,6 |
| Maraã | AM | 15 | 18.224 | 0,82 | 39,7 |
| Caracaraí | RR | 18 | 21.926 | 0,82 | 22,7 |
| Bom Jesus do Tocantins | PA | 14 | 16.981 | 0,82 | 10,2 |
| Darcinópolis | TO | 5 | 6.097 | 0,82 | 12,6 |
| Bom Jesus das Selvas | MA | 28 | 34.028 | 0,82 | 25,3 |
| Senador Elói de Souza | RN | 5 | 6.086 | 0,82 | 0,0 |
| Nova Palmeira | PB | 4 | 4.906 | 0,82 | 84,6 |
| Apuarema | BA | 6 | 7.330 | 0,82 | 0,0 |
| Encruzilhada | BA | 14 | 17.006 | 0,82 | 1,8 |
| Guaratinga | BA | 17 | 20.843 | 0,82 | 6,6 |
| Setubinha | MG | 10 | 12.258 | 0,82 | 25,6 |
| São José das Palmeiras | PR | 3 | 3.654 | 0,82 | 0,0 |
| Pinheirinho do Vale | RS | 4 | 4.871 | 0,82 | 0,0 |
| Aral Moreira | MS | 10 | 12.149 | 0,82 | 0,3 |
| Miranda | MS | 23 | 28.013 | 0,82 | 1,7 |
| Rosário Oeste | MT | 14 | 17.151 | 0,82 | 10,4 |
| Campinaçu | GO | 3 | 3.640 | 0,82 | 4,7 |
| Doverlândia | GO | 6 | 7.322 | 0,82 | 5,5 |
| Corumbiara | RO | 6 | 7.391 | 0,81 | 100,0 |
| Sena Madureira | AC | 37 | 45.848 | 0,81 | 17,2 |
| Cerro Corá | RN | 9 | 11.179 | 0,81 | 3,0 |
| Bom Jesus da Serra | BA | 8 | 9.880 | 0,81 | 1,1 |
| Itagi | BA | 10 | 12.351 | 0,81 | 2,0 |
| Gonzaga | MG | 5 | 6.158 | 0,81 | 18,4 |
| Ipanema | MG | 16 | 19.861 | 0,81 | 1,1 |
| São João da Lagoa | MG | 4 | 4.915 | 0,81 | 8,0 |
| Rio Rufino | SC | 2 | 2.483 | 0,81 | 0,0 |
| União do Oeste | SC | 2 | 2.464 | 0,81 | 0,0 |
| Cândido Godói | RS | 5 | 6.198 | 0,81 | 0,0 |
| Chapada dos Guimarães | MT | 16 | 19.752 | 0,81 | 13,0 |
| Nova Santa Helena | MT | 3 | 3.718 | 0,81 | 3,7 |
| Nova Maringá | MT | 7 | 8.641 | 0,81 | 28,3 |
| Aparecida do Rio Doce | GO | 2 | 2.474 | 0,81 | 4,9 |
| Cezarina | GO | 7 | 8.606 | 0,81 | 0,0 |
| Salvaterra | PA | 19 | 23.752 | 0,8 | 3,1 |
| Santana do Araguaia | PA | 58 | 72.817 | 0,8 | 19,8 |
| Redenção do Gurguéia | PI | 7 | 8.778 | 0,8 | 6,1 |
| Lajes | RN | 9 | 11.277 | 0,8 | 5,1 |
| Paraú | RN | 3 | 3.768 | 0,8 | 6,5 |
| Divino das Laranjeiras | MG | 4 | 4.979 | 0,8 | 0,2 |
| Inhaúma | MG | 5 | 6.271 | 0,8 | 0,0 |
| Óleo | SP | 2 | 2.496 | 0,8 | 0,0 |
| Pedranópolis | SP | 2 | 2.494 | 0,8 | 0,0 |
| Cametá | PA | 109 | 137.890 | 0,79 | 5,7 |
| Santa Terezinha do Tocantins | TO | 2 | 2.529 | 0,79 | 0,0 |
| São Mateus do Maranhão | MA | 33 | 41.529 | 0,79 | 0,6 |
| São Raimundo das Mangabeiras | MA | 15 | 18.868 | 0,79 | 11,1 |
| Vitória do Mearim | MA | 26 | 32.764 | 0,79 | 12,0 |
| Curral Velho | PB | 2 | 2.516 | 0,79 | 6,7 |
| Maiquinique | BA | 8 | 10.112 | 0,79 | 2,8 |
| Santa Cruz da Vitória | BA | 5 | 6.315 | 0,79 | 3,0 |
| Una | BA | 15 | 19.002 | 0,79 | 3,3 |
| Santa Maria de Jetibá | ES | 32 | 40.431 | 0,79 | 0,0 |
| Coronel Sapucaia | MS | 12 | 15.253 | 0,79 | 0,6 |
| Inocência | MS | 6 | 7.610 | 0,79 | 16,3 |
| Bom Jardim de Goiás | GO | 7 | 8.841 | 0,79 | 10,2 |
| Campo Alegre de Goiás | GO | 6 | 7.589 | 0,79 | 0,1 |
| Nova Timboteua | PA | 12 | 15.363 | 0,78 | 0,0 |
| Abreulândia | TO | 2 | 2.579 | 0,78 | 21,8 |
| Fátima | TO | 3 | 3.835 | 0,78 | 0,0 |
| Palmeirópolis | TO | 6 | 7.659 | 0,78 | 3,3 |
| Xambioá | TO | 9 | 11.540 | 0,78 | 1,0 |
| Potiretama | CE | 5 | 6.419 | 0,78 | 100,0 |
| Itapitanga | BA | 8 | 10.313 | 0,78 | 16,6 |
| Palmeiras | BA | 7 | 9.019 | 0,78 | 3,2 |
| Arinos | MG | 14 | 17.875 | 0,78 | 6,0 |
| Conceição da Aparecida | MG | 8 | 10.292 | 0,78 | 3,5 |
| Itaipé | MG | 10 | 12.760 | 0,78 | 6,0 |
| Piratuba | SC | 3 | 3.854 | 0,78 | 0,0 |
| Ipiranga do Norte | MT | 6 | 7.667 | 0,78 | 100,0 |
| Apuí | AM | 17 | 21.973 | 0,77 | 5,4 |
| Careiro | AM | 29 | 37.869 | 0,77 | 37,4 |
| Capanema | PA | 53 | 69.027 | 0,77 | 0,0 |
| Terra Alta | PA | 9 | 11.720 | 0,77 | 0,0 |
| Paranã | TO | 8 | 10.449 | 0,77 | 23,1 |
| Santa Filomena do Maranhão | MA | 6 | 7.773 | 0,77 | 13,5 |
| Pedra Lavrada | PB | 6 | 7.843 | 0,77 | 88,5 |
| Santa Rosa de Lima | SE | 3 | 3.913 | 0,77 | 0,0 |
| Alvarenga | MG | 3 | 3.907 | 0,77 | 1,4 |
| Dores de Guanhães | MG | 4 | 5.169 | 0,77 | 1,3 |
| Areias | SP | 3 | 3.886 | 0,77 | 0,0 |
| São Félix do Araguaia | MT | 9 | 11.708 | 0,77 | 99,4 |
| Arenópolis | GO | 2 | 2.612 | 0,77 | 2,6 |
| Guarani de Goiás | GO | 3 | 3.893 | 0,77 | 9,5 |
| Mimoso de Goiás | GO | 2 | 2.597 | 0,77 | 24,2 |
| Colorado do Oeste | RO | 12 | 15.882 | 0,76 | 19,8 |
| Alvorada D'Oeste | RO | 11 | 14.411 | 0,76 | 60,9 |
| Alto Alegre dos Parecis | RO | 10 | 13.241 | 0,76 | 19,7 |
| Baião | PA | 36 | 47.446 | 0,76 | 22,2 |
| Curuá | PA | 11 | 14.393 | 0,76 | 24,8 |
| São Valério | TO | 3 | 3.960 | 0,76 | 6,3 |
| Paquetá | PI | 3 | 3.945 | 0,76 | 4,1 |
| Almenara | MG | 32 | 41.896 | 0,76 | 3,3 |
| Carmésia | MG | 2 | 2.632 | 0,76 | 7,0 |
| Itarana | ES | 8 | 10.555 | 0,76 | 0,0 |
| Miracatu | SP | 15 | 19.779 | 0,76 | 0,0 |
| Novo Santo Antônio | MT | 2 | 2.640 | 0,76 | 20,7 |
| Cabixi | RO | 4 | 5.312 | 0,75 | 100,0 |
| Candeias do Jamari | RO | 20 | 26.693 | 0,75 | 27,3 |
| Manacapuru | AM | 73 | 97.377 | 0,75 | 16,6 |
| Tonantins | AM | 14 | 18.755 | 0,75 | 24,2 |
| Arapoema | TO | 5 | 6.643 | 0,75 | 3,8 |
| Lagoa da Confusão | TO | 10 | 13.357 | 0,75 | 22,7 |
| Palhano | CE | 7 | 9.386 | 0,75 | 13,9 |
| Santana do Seridó | RN | 2 | 2.680 | 0,75 | 7,8 |
| Cardeal da Silva | BA | 7 | 9.295 | 0,75 | 2,5 |
| Manhumirim | MG | 17 | 22.707 | 0,75 | 0,0 |
| Orizânia | MG | 6 | 8.018 | 0,75 | 0,0 |
| Afonso Cláudio | MG | 23 | 30.586 | 0,75 | 0,0 |
| Rancho Alegre D'Oeste | PR | 2 | 2.655 | 0,75 | 0,0 |
| Cabeceiras | GO | 6 | 7.993 | 0,75 | 0,2 |
| Alta Floresta D'Oeste | RO | 17 | 22.945 | 0,74 | 21,4 |
| Lábrea | AM | 34 | 46.069 | 0,74 | 20,8 |
| Cumaru do Norte | PA | 10 | 13.473 | 0,74 | 55,5 |
| Monte Alegre | PA | 43 | 58.032 | 0,74 | 6,5 |
| São João de Pirabas | PA | 17 | 23.045 | 0,74 | 0,1 |
| Serra do Navio | AP | 4 | 5.397 | 0,74 | 3,6 |
| São Miguel do Tocantins | TO | 9 | 12.139 | 0,74 | 0,0 |
| Centro do Guilherme | MA | 10 | 13.458 | 0,74 | 100,0 |
| Conceição do Lago-Açu | MA | 12 | 16.237 | 0,74 | 33,0 |
| Nova Colinas | MA | 4 | 5.384 | 0,74 | 0,1 |
| Alvorada do Gurguéia | PI | 4 | 5.419 | 0,74 | 14,0 |
| Frutuoso Gomes | RN | 3 | 4.068 | 0,74 | 91,6 |
| Palmas de Monte Alto | BA | 16 | 21.750 | 0,74 | 9,4 |
| Carrancas | MG | 3 | 4.047 | 0,74 | 2,0 |
| Domingos Martins | ES | 25 | 33.850 | 0,74 | 0,0 |
| Dores do Rio Preto | ES | 5 | 6.749 | 0,74 | 0,0 |
| Zacarias | SP | 2 | 2.718 | 0,74 | 0,9 |
| Ilópolis | RS | 3 | 4.078 | 0,74 | 0,1 |
| Cachoeira de Goiás | GO | 1 | 1.351 | 0,74 | 1,0 |
| Ourém | PA | 13 | 17.842 | 0,73 | 0,0 |
| Viseu | PA | 45 | 61.403 | 0,73 | 81,5 |
| Vitorino Freire | MA | 23 | 31.523 | 0,73 | 8,2 |
| São Bento do Norte | RN | 2 | 2.747 | 0,73 | 5,2 |
| Barra do Choça | BA | 23 | 31.603 | 0,73 | 0,7 |
| Belmonte | BA | 17 | 23.328 | 0,73 | 3,6 |
| Alto Rio Doce | MG | 8 | 11.000 | 0,73 | 0,3 |
| Marmelópolis | MG | 2 | 2.755 | 0,73 | 0,0 |
| Ibatiba | ES | 19 | 26.082 | 0,73 | 0,0 |
| Pancas | ES | 17 | 23.184 | 0,73 | 0,0 |
| Espigão Alto do Iguaçu | PR | 3 | 4.117 | 0,73 | 0,0 |
| Camargo | RS | 2 | 2.733 | 0,73 | 0,0 |
| Alto Boa Vista | MT | 5 | 6.822 | 0,73 | 13,5 |
| Edéia | GO | 9 | 12.372 | 0,73 | 0,1 |
| Presidente Figueiredo | AM | 26 | 36.279 | 0,72 | 13,8 |
| Guajeru | BA | 5 | 6.937 | 0,72 | 7,1 |
| Córrego Novo | MG | 2 | 2.771 | 0,72 | 1,4 |
| Espera Feliz | MG | 18 | 24.951 | 0,72 | 0,0 |
| Lima Duarte | MG | 12 | 16.698 | 0,72 | 0,1 |
| Alto Jequitibá | MG | 6 | 8.317 | 0,72 | 0,0 |
| São José do Mantimento | MG | 2 | 2.791 | 0,72 | 0,0 |
| Sabino | SP | 4 | 5.590 | 0,72 | 0,0 |
| Santo Antônio do Leverger | MT | 12 | 16.628 | 0,72 | 35,6 |
| Corumbaíba | GO | 7 | 9.723 | 0,72 | 1,9 |
| Flores de Goiás | GO | 12 | 16.557 | 0,72 | 24,6 |
| São Sebastião do Uatumã | AM | 10 | 14.020 | 0,71 | 25,8 |
| Vigia | PA | 38 | 53.686 | 0,71 | 0,0 |
| Fortaleza dos Nogueiras | MA | 9 | 12.631 | 0,71 | 4,7 |
| Paulo Ramos | MA | 15 | 21.040 | 0,71 | 3,3 |
| Banabuiú | CE | 13 | 18.197 | 0,71 | 11,1 |
| Riacho de Santana | RN | 3 | 4.204 | 0,71 | 100,0 |
| São João do Cariri | PB | 3 | 4.199 | 0,71 | 100,0 |
| Umbuzeiro | PB | 7 | 9.907 | 0,71 | 46,0 |
| Santa Cruz | PE | 11 | 15.398 | 0,71 | 29,0 |
| Souto Soares | BA | 12 | 16.979 | 0,71 | 19,5 |
| Gurinhatã | MG | 4 | 5.639 | 0,71 | 4,0 |
| Salto da Divisa | MG | 5 | 7.009 | 0,71 | 6,8 |
| São Domingos das Dores | MG | 4 | 5.644 | 0,71 | 0,0 |
| Pedro de Toledo | SP | 8 | 11.331 | 0,71 | 0,6 |
| São Francisco | SP | 2 | 2.821 | 0,71 | 0,0 |
| Grandes Rios | PR | 4 | 5.618 | 0,71 | 0,0 |
| Nova Tebas | PR | 4 | 5.649 | 0,71 | 0,0 |
| Derrubadas | RS | 2 | 2.805 | 0,71 | 0,0 |
| São José do Xingu | MT | 4 | 5.595 | 0,71 | 100,0 |
| Três Ranchos | GO | 2 | 2.833 | 0,71 | 0,0 |
| Alenquer | PA | 40 | 56.789 | 0,7 | 15,8 |
| Altamira | PA | 80 | 114.594 | 0,7 | 7,8 |
| Capitão Poço | PA | 38 | 54.303 | 0,7 | 4,3 |
| São Miguel do Guamá | PA | 41 | 58.986 | 0,7 | 2,2 |
| Araguanã | TO | 4 | 5.729 | 0,7 | 1,0 |
| Tocantinópolis | TO | 16 | 22.870 | 0,7 | 0,2 |
| Sebastião Leal | PI | 3 | 4.294 | 0,7 | 17,3 |
| Santana do Matos | RN | 9 | 12.791 | 0,7 | 44,7 |
| Picuí | PB | 13 | 18.703 | 0,7 | 14,4 |
| Barra do Rocha | BA | 4 | 5.714 | 0,7 | 7,8 |
| Gongogi | BA | 5 | 7.128 | 0,7 | 4,4 |
| Itamaraju | BA | 45 | 64.486 | 0,7 | 4,0 |
| Malacacheta | MG | 13 | 18.650 | 0,7 | 1,2 |
| Rio Claro | RJ | 13 | 18.529 | 0,7 | 0,0 |
| Nova Canaã do Norte | MT | 9 | 12.787 | 0,7 | 18,9 |
| Nova Xavantina | MT | 15 | 21.374 | 0,7 | 10,0 |
| Santa Cruz de Goiás | GO | 2 | 2.855 | 0,7 | 7,3 |
| Marechal Thaumaturgo | AC | 13 | 18.867 | 0,69 | 41,5 |
| Careiro da Várzea | AM | 21 | 30.225 | 0,69 | 61,3 |
| Goianésia do Pará | PA | 28 | 40.475 | 0,69 | 15,6 |
| Francisco Ayres | PI | 3 | 4.348 | 0,69 | 0,7 |
| Nossa Senhora dos Remédios | PI | 6 | 8.692 | 0,69 | 6,1 |
| Mato Grosso | PB | 2 | 2.908 | 0,69 | 98,2 |
| Aurelino Leal | BA | 8 | 11.531 | 0,69 | 4,4 |
| Camacan | BA | 22 | 31.988 | 0,69 | 1,3 |
| Brás Pires | MG | 3 | 4.333 | 0,69 | 0,0 |
| Camacho | MG | 2 | 2.901 | 0,69 | 0,0 |
| Desterro do Melo | MG | 2 | 2.901 | 0,69 | 0,0 |
| Floreal | SP | 2 | 2.917 | 0,69 | 0,0 |
| Centenário | RS | 2 | 2.891 | 0,69 | 6,8 |
| Lagoa Bonita do Sul | RS | 2 | 2.903 | 0,69 | 5,1 |
| Maximiliano de Almeida | RS | 3 | 4.377 | 0,69 | 1,4 |
| Sede Nova | RS | 2 | 2.907 | 0,69 | 0,0 |
| Querência | MT | 12 | 17.479 | 0,69 | 24,4 |
| Bujari | AC | 7 | 10.266 | 0,68 | 27,3 |
| Coari | AM | 58 | 85.097 | 0,68 | 24,2 |
| Juruá | AM | 10 | 14.712 | 0,68 | 39,2 |
| Parintins | AM | 78 | 114.273 | 0,68 | 17,8 |
| Filadélfia | TO | 6 | 8.856 | 0,68 | 13,2 |
| Aratuba | CE | 8 | 11.847 | 0,68 | 22,6 |
| Santa Maria do Suaçuí | MG | 10 | 14.615 | 0,68 | 1,8 |
| Marquinho | PR | 3 | 4.405 | 0,68 | 17,5 |
| Poxoréo | MT | 11 | 16.219 | 0,68 | 100,0 |
| Nova Iguaçu de Goiás | GO | 2 | 2.929 | 0,68 | 0,1 |
| Cujubim | RO | 17 | 25.215 | 0,67 | 98,1 |
| Barcarena | PA | 84 | 124.680 | 0,67 | 0,2 |
| Igarapé-Açu | PA | 26 | 38.807 | 0,67 | 0,1 |
| Santo Antônio do Tauá | PA | 21 | 31.482 | 0,67 | 0,0 |
| Laranjal do Jari | AP | 34 | 50.410 | 0,67 | 1,8 |
| Santa Fé do Araguaia | TO | 5 | 7.512 | 0,67 | 24,4 |
| Nova Olinda do Maranhão | MA | 14 | 20.928 | 0,67 | 28,6 |
| Campo Grande do Piauí | PI | 4 | 5.943 | 0,67 | 3,4 |
| Frei Martinho | PB | 2 | 2.989 | 0,67 | 35,9 |
| Chã de Alegria | PE | 9 | 13.518 | 0,67 | 0,0 |
| Aiquara | BA | 3 | 4.446 | 0,67 | 0,0 |
| Caraíbas | BA | 6 | 8.951 | 0,67 | 10,6 |
| Laje | BA | 16 | 23.840 | 0,67 | 0,5 |
| Nazaré | BA | 19 | 28.525 | 0,67 | 0,0 |
| Dom Viçoso | MG | 2 | 3.001 | 0,67 | 0,0 |
| Maripá de Minas | MG | 2 | 2.973 | 0,67 | 0,0 |
| Novo Cruzeiro | MG | 21 | 31.331 | 0,67 | 11,3 |
| São Sebastião da Vargem Alegre | MG | 2 | 3.007 | 0,67 | 0,0 |
| Senhora dos Remédios | MG | 7 | 10.459 | 0,67 | 0,0 |
| Virginópolis | MG | 7 | 10.510 | 0,67 | 13,6 |
| Cajati | SP | 19 | 28.549 | 0,67 | 0,0 |
| Sulina | PR | 2 | 2.981 | 0,67 | 0,3 |
| Barão do Triunfo | RS | 5 | 7.487 | 0,67 | 99,3 |
| Mampituba | RS | 2 | 2.981 | 0,67 | 0,0 |
| Comodoro | MT | 14 | 20.763 | 0,67 | 15,9 |
| Cristianópolis | GO | 2 | 2.966 | 0,67 | 0,1 |
| Castanheiras | RO | 2 | 3.052 | 0,66 | 1,0 |
| Oiapoque | AP | 18 | 27.270 | 0,66 | 17,1 |
| Ingazeira | PE | 3 | 4.548 | 0,66 | 25,4 |
| Mucugê | BA | 6 | 9.062 | 0,66 | 1,5 |
| Água Boa | MG | 9 | 13.735 | 0,66 | 6,7 |
| Joanésia | MG | 3 | 4.573 | 0,66 | 0,0 |
| Westfalia | RS | 2 | 3.014 | 0,66 | 0,0 |
| Figueirão | MS | 2 | 3.051 | 0,66 | 19,7 |
| Glória D'Oeste | MT | 2 | 3.026 | 0,66 | 5,9 |
| Ponte Alta do Bom Jesus | TO | 3 | 4.596 | 0,65 | 7,4 |
| São Salvador do Tocantins | TO | 2 | 3.082 | 0,65 | 13,0 |
| Governador Archer | MA | 7 | 10.840 | 0,65 | 7,8 |
| Poranga | CE | 8 | 12.336 | 0,65 | 36,0 |
| São João do Jaguaribe | CE | 5 | 7.645 | 0,65 | 1,4 |
| Pocinhos | PB | 12 | 18.564 | 0,65 | 19,8 |
| Coronel Pacheco | MG | 2 | 3.086 | 0,65 | 0,0 |
| Jequeri | MG | 8 | 12.386 | 0,65 | 0,8 |
| Mercês | MG | 7 | 10.739 | 0,65 | 3,5 |
| Miradouro | MG | 7 | 10.754 | 0,65 | 1,8 |
| Sabinópolis | MG | 10 | 15.470 | 0,65 | 5,4 |
| Santa Salete | SP | 1 | 1.545 | 0,65 | 0,0 |
| Guaraqueçaba | PR | 5 | 7.636 | 0,65 | 28,3 |
| Riqueza | SC | 3 | 4.598 | 0,65 | 0,0 |
| Gaúcha do Norte | MT | 5 | 7.648 | 0,65 | 100,0 |
| Mirante da Serra | RO | 7 | 10.947 | 0,64 | 100,0 |
| Tabatinga | AM | 42 | 65.844 | 0,64 | 13,3 |
| Araguatins | TO | 23 | 35.761 | 0,64 | 0,1 |
| Luzinópolis | TO | 2 | 3.108 | 0,64 | 0,0 |
| Graça Aranha | MA | 4 | 6.262 | 0,64 | 0,0 |
| Pirapemas | MA | 12 | 18.625 | 0,64 | 4,4 |
| São Pedro dos Crentes | MA | 3 | 4.668 | 0,64 | 8,4 |
| Zé Doca | MA | 33 | 51.714 | 0,64 | 23,0 |
| Santa Cruz do Piauí | PI | 4 | 6.240 | 0,64 | 6,3 |
| Arneiroz | CE | 5 | 7.840 | 0,64 | 100,0 |
| Ibirapitanga | BA | 15 | 23.375 | 0,64 | 3,2 |
| Comendador Gomes | MG | 2 | 3.111 | 0,64 | 5,8 |
| Ervália | MG | 12 | 18.895 | 0,64 | 0,0 |
| Senador Firmino | MG | 5 | 7.812 | 0,64 | 0,0 |
| Taparuba | MG | 2 | 3.110 | 0,64 | 0,0 |
| Itaguaçu | ES | 9 | 14.066 | 0,64 | 0,0 |
| Areal | RJ | 8 | 12.572 | 0,64 | 0,0 |
| Sumidouro | RJ | 10 | 15.623 | 0,64 | 0,0 |
| Tapiraí | SP | 5 | 7.807 | 0,64 | 0,2 |
| Jardinópolis | SC | 1 | 1.570 | 0,64 | 0,0 |
| São Domingos | SC | 6 | 9.445 | 0,64 | 0,0 |
| Mozarlândia | GO | 10 | 15.659 | 0,64 | 0,1 |
| Paraúna | GO | 7 | 10.988 | 0,64 | 1,1 |
| Machadinho D'Oeste | RO | 25 | 39.991 | 0,63 | 100,0 |
| Dianópolis | TO | 14 | 22.139 | 0,63 | 1,7 |
| Monção | MA | 21 | 33.434 | 0,63 | 29,1 |
| São João Batista | MA | 13 | 20.665 | 0,63 | 7,3 |
| Dom Inocêncio | PI | 6 | 9.556 | 0,63 | 53,4 |
| Almino Afonso | RN | 3 | 4.735 | 0,63 | 20,5 |
| Serrinha dos Pintos | RN | 3 | 4.800 | 0,63 | 100,0 |
| Aroeiras | PB | 12 | 19.153 | 0,63 | 12,1 |
| Cachoeira | BA | 21 | 33.470 | 0,63 | 0,0 |
| Maetinga | BA | 2 | 3.161 | 0,63 | 18,5 |
| Antônio Prado de Minas | MG | 1 | 1.598 | 0,63 | 0,0 |
| Formoso | MG | 6 | 9.562 | 0,63 | 6,9 |
| Lagoa Grande | MG | 6 | 9.532 | 0,63 | 8,0 |
| Raul Soares | MG | 15 | 23.762 | 0,63 | 0,4 |
| Tapira | MG | 3 | 4.773 | 0,63 | 2,9 |
| Igaratá | SP | 6 | 9.534 | 0,63 | 0,0 |
| Corupá | SC | 10 | 15.909 | 0,63 | 0,0 |
| Flor do Sertão | SC | 1 | 1.582 | 0,63 | 0,0 |
| Ernestina | RS | 2 | 3.168 | 0,63 | 0,0 |
| Iranduba | AM | 30 | 48.296 | 0,62 | 1,6 |
| Curuçá | PA | 25 | 40.066 | 0,62 | 0,2 |
| Combinado | TO | 3 | 4.852 | 0,62 | 0,0 |
| Santa Rosa do Tocantins | TO | 3 | 4.829 | 0,62 | 2,3 |
| Granjeiro | CE | 3 | 4.844 | 0,62 | 0,0 |
| São José do Campestre | RN | 8 | 12.856 | 0,62 | 1,9 |
| Santa Filomena | PE | 9 | 14.477 | 0,62 | 52,4 |
| Faria Lemos | MG | 2 | 3.241 | 0,62 | 0,0 |
| Inhapim | MG | 15 | 24.140 | 0,62 | 1,6 |
| Monte Alegre de Minas | MG | 13 | 21.120 | 0,62 | 5,7 |
| Vargem Alegre | MG | 4 | 6.480 | 0,62 | 0,0 |
| Vermelho Novo | MG | 3 | 4.839 | 0,62 | 0,0 |
| Águia Branca | ES | 6 | 9.642 | 0,62 | 0,0 |
| Glicério | SP | 3 | 4.815 | 0,62 | 0,0 |
| Angelina | SC | 3 | 4.801 | 0,62 | 0,0 |
| Barra do Guarita | RS | 2 | 3.248 | 0,62 | 0,0 |
| Cristal | RS | 5 | 8.009 | 0,62 | 100,0 |
| Monte Alegre dos Campos | RS | 2 | 3.226 | 0,62 | 94,9 |
| Terra Nova do Norte | MT | 6 | 9.667 | 0,62 | 4,5 |
| Montes Claros de Goiás | GO | 5 | 8.055 | 0,62 | 9,3 |
| Rondon do Pará | PA | 32 | 52.357 | 0,61 | 17,6 |
| Afonso Cunha | MA | 4 | 6.524 | 0,61 | 5,6 |
| Pio XII | MA | 13 | 21.485 | 0,61 | 6,9 |
| Sítio Novo | MA | 11 | 18.081 | 0,61 | 43,4 |
| Baraúna | PB | 3 | 4.892 | 0,61 | 0,1 |
| Santa Cecília | PB | 4 | 6.558 | 0,61 | 62,9 |
| Itarantim | BA | 12 | 19.747 | 0,61 | 1,5 |
| Guarda-Mor | MG | 4 | 6.580 | 0,61 | 5,5 |
| São Francisco de Paula | MG | 4 | 6.527 | 0,61 | 0,5 |
| Uruana de Minas | MG | 2 | 3.264 | 0,61 | 5,5 |
| Mato Rico | PR | 2 | 3.272 | 0,61 | 0,0 |
| Descanso | SC | 5 | 8.250 | 0,61 | 0,0 |
| Serra Alta | SC | 2 | 3.263 | 0,61 | 0,0 |
| Tigrinhos | SC | 1 | 1.633 | 0,61 | 0,0 |
| Coronel Pilar | RS | 1 | 1.628 | 0,61 | 0,0 |
| Santa Cecília do Sul | RS | 1 | 1.639 | 0,61 | 0,3 |
| São Valentim | RS | 2 | 3.299 | 0,61 | 0,0 |
| Amambai | MS | 24 | 39.396 | 0,61 | 1,7 |
| Cotriguaçu | MT | 12 | 19.750 | 0,61 | 21,8 |
| Buritinópolis | GO | 2 | 3.292 | 0,61 | 0,0 |
| Costa Marques | RO | 11 | 18.331 | 0,6 | 16,1 |
| Atalaia do Norte | AM | 12 | 19.921 | 0,6 | 47,9 |
| Marapanim | PA | 17 | 28.336 | 0,6 | 0,8 |
| Alto Alegre do Pindaré | MA | 19 | 31.919 | 0,6 | 98,0 |
| Itatira | CE | 13 | 21.647 | 0,6 | 84,2 |
| Barra de Santana | PB | 5 | 8.359 | 0,6 | 68,7 |
| Bonito | BA | 10 | 16.764 | 0,6 | 0,8 |
| Bom Jesus do Galho | MG | 9 | 14.935 | 0,6 | 0,5 |
| Cândido de Abreu | PR | 9 | 15.018 | 0,6 | 4,7 |
| Lidianópolis | PR | 2 | 3.310 | 0,6 | 0,0 |
| Vitor Meireles | SC | 3 | 4.979 | 0,6 | 25,8 |
| Campos Borges | RS | 2 | 3.320 | 0,6 | 78,2 |
| Itatiba do Sul | RS | 2 | 3.324 | 0,6 | 2,3 |
| Novo Machado | RS | 2 | 3.323 | 0,6 | 0,0 |
| Itiquira | MT | 8 | 13.345 | 0,6 | 13,2 |
| Santa Rita do Novo Destino | GO | 2 | 3.343 | 0,6 | 8,4 |
| Codajás | AM | 17 | 28.637 | 0,59 | 26,4 |
| Cachoeira do Arari | PA | 14 | 23.767 | 0,59 | 26,5 |
| Soure | PA | 15 | 25.374 | 0,59 | 2,9 |
| Pracuúba | AP | 3 | 5.120 | 0,59 | 3,7 |
| Buriti Bravo | MA | 14 | 23.884 | 0,59 | 16,2 |
| Tasso Fragoso | MA | 5 | 8.521 | 0,59 | 20,5 |
| Serra do Mel | RN | 7 | 11.938 | 0,59 | 0,0 |
| Angelândia | MG | 5 | 8.520 | 0,59 | 0,0 |
| Araponga | MG | 5 | 8.439 | 0,59 | 0,0 |
| Berilo | MG | 7 | 11.932 | 0,59 | 0,7 |
| Divinésia | MG | 2 | 3.417 | 0,59 | 0,0 |
| Laranjal | MG | 4 | 6.810 | 0,59 | 0,0 |
| Pocrane | MG | 5 | 8.432 | 0,59 | 0,3 |
| Rubim | MG | 6 | 10.241 | 0,59 | 0,3 |
| São Félix de Minas | MG | 2 | 3.369 | 0,59 | 0,3 |
| Nazaré Paulista | SP | 11 | 18.524 | 0,59 | 0,0 |
| Iretama | PR | 6 | 10.169 | 0,59 | 0,0 |
| Sabáudia | PR | 4 | 6.827 | 0,59 | 0,0 |
| São João | PR | 6 | 10.241 | 0,59 | 0,0 |
| Nicolau Vergueiro | RS | 1 | 1.682 | 0,59 | 0,0 |
| Ribeirão Cascalheira | MT | 6 | 10.206 | 0,59 | 21,0 |
| Canutama | AM | 9 | 15.629 | 0,58 | 22,6 |
| Araguanã | MA | 9 | 15.426 | 0,58 | 49,1 |
| Fernando Falcão | MA | 6 | 10.360 | 0,58 | 44,1 |
| Guimarães | MA | 7 | 12.030 | 0,58 | 0,0 |
| Dom Expedito Lopes | PI | 4 | 6.904 | 0,58 | 0,0 |
| Guaramiranga | CE | 3 | 5.193 | 0,58 | 0,7 |
| Serra Caiada | RN | 6 | 10.395 | 0,58 | 0,0 |
| Ipubi | PE | 18 | 30.854 | 0,58 | 9,4 |
| Sertânia | PE | 21 | 35.907 | 0,58 | 3,5 |
| Alfredo Vasconcelos | MG | 4 | 6.907 | 0,58 | 0,0 |
| Capetinga | MG | 4 | 6.920 | 0,58 | 0,0 |
| Fernandes Tourinho | MG | 2 | 3.431 | 0,58 | 2,2 |
| Francisco Dumont | MG | 3 | 5.215 | 0,58 | 29,5 |
| Santa Rita de Ibitipoca | MG | 2 | 3.425 | 0,58 | 0,0 |
| Águas de São Pedro | SP | 2 | 3.451 | 0,58 | 0,0 |
| Trabiju | SP | 1 | 1.724 | 0,58 | 0,0 |
| Honório Serpa | PR | 3 | 5.211 | 0,58 | 0,0 |
| Bocaina do Sul | SC | 2 | 3.474 | 0,58 | 0,0 |
| Erval Seco | RS | 4 | 6.912 | 0,58 | 0,5 |
| Itacurubi | RS | 2 | 3.465 | 0,58 | 100,0 |
| Barão de Melgaço | MT | 5 | 8.564 | 0,58 | 47,9 |
| Monte Alegre de Goiás | GO | 5 | 8.606 | 0,58 | 15,2 |
| Piranhas | GO | 6 | 10.385 | 0,58 | 2,4 |
| Simolândia | GO | 4 | 6.856 | 0,58 | 1,6 |
| Nova União | RO | 4 | 6.970 | 0,57 | 100,0 |
| Uruará | PA | 26 | 45.476 | 0,57 | 16,7 |
| Aricanduva | MG | 3 | 5.231 | 0,57 | 28,4 |
| Novorizonte | MG | 3 | 5.299 | 0,57 | 0,0 |
| Prata | MG | 16 | 27.856 | 0,57 | 10,5 |
| Romaria | MG | 2 | 3.533 | 0,57 | 4,8 |
| Santo Antônio do Rio Abaixo | MG | 1 | 1.765 | 0,57 | 0,1 |
| São Romão | MG | 7 | 12.337 | 0,57 | 18,5 |
| Rio Bananal | ES | 11 | 19.141 | 0,57 | 0,0 |
| São Lourenço da Serra | SP | 9 | 15.825 | 0,57 | 0,0 |
| Botuverá | SC | 3 | 5.246 | 0,57 | 0,0 |
| Santa Terezinha de Goiás | GO | 5 | 8.744 | 0,57 | 0,0 |
| Taquaral de Goiás | GO | 2 | 3.529 | 0,57 | 0,0 |
| Senador Guiomard | AC | 13 | 23.024 | 0,56 | 9,0 |
| Rorainópolis | RR | 17 | 30.163 | 0,56 | 20,0 |
| Caseara | TO | 3 | 5.369 | 0,56 | 6,3 |
| Lago dos Rodrigues | MA | 5 | 8.873 | 0,56 | 0,0 |
| São Pedro do Piauí | PI | 8 | 14.291 | 0,56 | 0,9 |
| Caririaçu | CE | 15 | 26.965 | 0,56 | 1,3 |
| Damião | PB | 3 | 5.330 | 0,56 | 9,3 |
| São José do Brejo do Cruz | PB | 1 | 1.801 | 0,56 | 15,8 |
| Siriri | SE | 5 | 8.893 | 0,56 | 0,0 |
| Cravolândia | BA | 3 | 5.349 | 0,56 | 0,0 |
| Itambé | BA | 13 | 23.049 | 0,56 | 3,9 |
| Santa Luzia | BA | 7 | 12.597 | 0,56 | 0,7 |
| Coluna | MG | 5 | 8.873 | 0,56 | 0,0 |
| Consolação | MG | 1 | 1.783 | 0,56 | 0,0 |
| Fruta de Leite | MG | 3 | 5.369 | 0,56 | 11,9 |
| Pratinha | MG | 2 | 3.603 | 0,56 | 0,0 |
| Presidente Bernardes | MG | 3 | 5.369 | 0,56 | 0,0 |
| Santa Vitória | MG | 11 | 19.742 | 0,56 | 0,1 |
| Santo Antônio do Aventureiro | MG | 2 | 3.602 | 0,56 | 0,0 |
| Arroio Trinta | SC | 2 | 3.550 | 0,56 | 0,0 |
| Feijó | AC | 19 | 34.780 | 0,55 | 25,2 |
| Primavera | PA | 6 | 10.825 | 0,55 | 0,0 |
| Santarém | PA | 167 | 304.589 | 0,55 | 14,7 |
| São Caetano de Odivelas | PA | 10 | 18.050 | 0,55 | 0,0 |
| Tracuateua | PA | 17 | 30.959 | 0,55 | 2,8 |
| Pequizeiro | TO | 3 | 5.477 | 0,55 | 20,1 |
| São Pedro da Água Branca | MA | 7 | 12.690 | 0,55 | 0,6 |
| Timbiras | MA | 16 | 29.124 | 0,55 | 14,7 |
| Itaporanga d'Ajuda | SE | 19 | 34.356 | 0,55 | 0,0 |
| Jiquiriçá | BA | 8 | 14.537 | 0,55 | 22,2 |
| Pindaí | BA | 9 | 16.260 | 0,55 | 0,0 |
| Ubaíra | BA | 11 | 19.895 | 0,55 | 8,9 |
| Bonfinópolis de Minas | MG | 3 | 5.493 | 0,55 | 16,9 |
| Caparaó | MG | 3 | 5.438 | 0,55 | 0,0 |
| Caraí | MG | 13 | 23.685 | 0,55 | 5,5 |
| Iapu | MG | 6 | 11.004 | 0,55 | 1,0 |
| Lajinha | MG | 11 | 19.923 | 0,55 | 0,1 |
| Santa Maria de Itabira | MG | 6 | 10.847 | 0,55 | 3,5 |
| Caxambu do Sul | SC | 2 | 3.642 | 0,55 | 0,0 |
| São João do Sul | SC | 4 | 7.297 | 0,55 | 0,0 |
| Capão do Cipó | RS | 2 | 3.651 | 0,55 | 97,2 |
| Chuvisca | RS | 3 | 5.441 | 0,55 | 100,0 |
| Estrela Velha | RS | 2 | 3.655 | 0,55 | 95,7 |
| Rochedo | MS | 3 | 5.499 | 0,55 | 3,9 |
| Poconé | MT | 18 | 32.843 | 0,55 | 6,7 |
| Minaçu | GO | 16 | 29.070 | 0,55 | 1,8 |
| Nova Brasilândia D'Oeste | RO | 11 | 20.474 | 0,54 | 18,7 |
| Natividade | TO | 5 | 9.244 | 0,54 | 3,3 |
| Boa Vista do Gurupi | MA | 5 | 9.287 | 0,54 | 100,0 |
| Casserengue | PB | 4 | 7.468 | 0,54 | 45,1 |
| Carnaubeira da Penha | PE | 7 | 12.932 | 0,54 | 72,9 |
| Caturama | BA | 5 | 9.329 | 0,54 | 4,4 |
| Esplanada | BA | 20 | 37.237 | 0,54 | 0,1 |
| Seritinga | MG | 1 | 1.851 | 0,54 | 0,0 |
| Água Doce do Norte | ES | 6 | 11.019 | 0,54 | 0,0 |
| Vitória Brasil | SP | 1 | 1.840 | 0,54 | 0,0 |
| Inácio Martins | PR | 6 | 11.125 | 0,54 | 13,3 |
| Paraíso das Águas | MS | 3 | 5.555 | 0,54 | 31,1 |
| Água Boa | MT | 14 | 25.721 | 0,54 | 10,3 |
| Novo Mundo | MT | 5 | 9.178 | 0,54 | 22,3 |
| Corumbá de Goiás | GO | 6 | 11.110 | 0,54 | 0,0 |
| Nova Crixás | GO | 7 | 12.869 | 0,54 | 14,8 |
| Carauari | AM | 15 | 28.294 | 0,53 | 17,3 |
| Ipixuna do Pará | PA | 34 | 64.053 | 0,53 | 37,4 |
| Mãe do Rio | PA | 16 | 30.077 | 0,53 | 0,0 |
| Buriti do Tocantins | TO | 6 | 11.348 | 0,53 | 0,0 |
| Porto Nacional | TO | 28 | 53.010 | 0,53 | 1,1 |
| Lagoa do Mato | MA | 6 | 11.250 | 0,53 | 88,4 |
| São Luís Gonzaga do Maranhão | MA | 10 | 18.856 | 0,53 | 6,2 |
| Ilha Grande | PI | 5 | 9.426 | 0,53 | 0,0 |
| Caridade | CE | 12 | 22.547 | 0,53 | 15,2 |
| Jaguaruana | CE | 18 | 33.705 | 0,53 | 2,2 |
| Campo Redondo | RN | 6 | 11.217 | 0,53 | 0,6 |
| Lagoa de Pedras | RN | 4 | 7.544 | 0,53 | 0,0 |
| Aramari | BA | 6 | 11.397 | 0,53 | 0,0 |
| Coaraci | BA | 9 | 16.993 | 0,53 | 4,4 |
| Lajedinho | BA | 2 | 3.783 | 0,53 | 5,2 |
| São José da Vitória | BA | 3 | 5.657 | 0,53 | 0,3 |
| Bonito de Minas | MG | 6 | 11.230 | 0,53 | 59,6 |
| Capelinha | MG | 20 | 37.784 | 0,53 | 0,2 |
| Diogo de Vasconcelos | MG | 2 | 3.802 | 0,53 | 0,0 |
| Pintópolis | MG | 4 | 7.507 | 0,53 | 11,5 |
| Riacho dos Machados | MG | 5 | 9.481 | 0,53 | 24,7 |
| Santana dos Montes | MG | 2 | 3.777 | 0,53 | 0,0 |
| Tabuleiro | MG | 2 | 3.750 | 0,53 | 0,5 |
| Tapiraí | MG | 1 | 1.875 | 0,53 | 0,0 |
| Juquiá | SP | 10 | 18.812 | 0,53 | 0,0 |
| Rancho Alegre | PR | 2 | 3.808 | 0,53 | 0,0 |
| Ipuaçu | SC | 4 | 7.514 | 0,53 | 0,0 |
| Massaranduba | SC | 9 | 16.916 | 0,53 | 0,0 |
| Guarani das Missões | RS | 4 | 7.537 | 0,53 | 0,0 |
| Ipiranga do Sul | RS | 1 | 1.889 | 0,53 | 0,0 |
| Alto Paraguai | MT | 6 | 11.356 | 0,53 | 24,6 |
| São José dos Quatro Marcos | MT | 10 | 18.906 | 0,53 | 0,5 |
| Tabaporã | MT | 5 | 9.489 | 0,53 | 29,0 |
| Tesouro | MT | 2 | 3.805 | 0,53 | 100,0 |
| Vera | MT | 6 | 11.309 | 0,53 | 100,0 |
| Buriti Alegre | GO | 5 | 9.459 | 0,53 | 0,3 |
| Mara Rosa | GO | 5 | 9.503 | 0,53 | 2,4 |
| Mutunópolis | GO | 2 | 3.778 | 0,53 | 1,6 |
| Silvânia | GO | 11 | 20.695 | 0,53 | 0,2 |
| Alagoinha do Piauí | PI | 4 | 7.651 | 0,52 | 31,1 |
| Colônia do Piauí | PI | 4 | 7.656 | 0,52 | 3,0 |
| Jacobina do Piauí | PI | 3 | 5.741 | 0,52 | 33,1 |
| Ibaretama | CE | 7 | 13.353 | 0,52 | 13,5 |
| Taperoá | PB | 8 | 15.376 | 0,52 | 100,0 |
| Ibirataia | BA | 8 | 15.312 | 0,52 | 0,1 |
| Belo Vale | MG | 4 | 7.715 | 0,52 | 0,0 |
| Itabirinha | MG | 6 | 11.512 | 0,52 | 0,0 |
| Jenipapo de Minas | MG | 4 | 7.692 | 0,52 | 29,8 |
| Nova União | MG | 3 | 5.725 | 0,52 | 0,0 |
| Barra do Chapéu | SP | 3 | 5.724 | 0,52 | 0,9 |
| Eldorado | SP | 8 | 15.494 | 0,52 | 0,5 |
| Guapiara | SP | 9 | 17.157 | 0,52 | 0,0 |
| Luiziânia | SP | 3 | 5.790 | 0,52 | 0,0 |
| Mesópolis | SP | 1 | 1.908 | 0,52 | 0,0 |
| Quitandinha | PR | 10 | 19.049 | 0,52 | 0,0 |
| Dom Feliciano | RS | 8 | 15.414 | 0,52 | 97,9 |
| Pejuçara | RS | 2 | 3.874 | 0,52 | 0,0 |
| Pinhal da Serra | RS | 1 | 1.941 | 0,52 | 0,5 |
| Nova Brasilândia | MT | 2 | 3.829 | 0,52 | 5,8 |
| Itaituba | PA | 52 | 101.247 | 0,51 | 9,8 |
| Rurópolis | PA | 26 | 50.510 | 0,51 | 9,0 |
| Ulianópolis | PA | 30 | 59.210 | 0,51 | 12,8 |
| Axixá do Tocantins | TO | 5 | 9.756 | 0,51 | 0,0 |
| Itaguatins | TO | 3 | 5.864 | 0,51 | 6,0 |
| Sucupira | TO | 1 | 1.966 | 0,51 | 12,2 |
| Balsas | MA | 48 | 94.887 | 0,51 | 5,5 |
| Centro Novo do Maranhão | MA | 11 | 21.622 | 0,51 | 99,8 |
| Caraúbas do Piauí | PI | 3 | 5.868 | 0,51 | 8,5 |
| Madalena | CE | 10 | 19.691 | 0,51 | 40,8 |
| Tauá | CE | 30 | 58.859 | 0,51 | 100,0 |
| Angicos | RN | 6 | 11.714 | 0,51 | 99,1 |
| Olivedos | PB | 2 | 3.932 | 0,51 | 99,1 |
| Poço Dantas | PB | 2 | 3.888 | 0,51 | 100,0 |
| Quixabá | PB | 1 | 1.956 | 0,51 | 14,7 |
| Antônio Cardoso | BA | 6 | 11.683 | 0,51 | 0,0 |
| Minduri | MG | 2 | 3.894 | 0,51 | 0,0 |
| Ouro Verde de Minas | MG | 3 | 5.934 | 0,51 | 6,0 |
| Ilhabela | SP | 18 | 34.970 | 0,51 | 1,0 |
| Roncador | PR | 5 | 9.849 | 0,51 | 0,0 |
| Quilombo | SC | 5 | 9.887 | 0,51 | 0,0 |
| Rio dos Cedros | SC | 6 | 11.676 | 0,51 | 0,0 |
| Rio Verde de Mato Grosso | MS | 10 | 19.746 | 0,51 | 3,8 |
| Canarana | MT | 11 | 21.579 | 0,51 | 13,3 |
| Buritis | RO | 20 | 39.654 | 0,5 | 100,0 |
| Eldorado do Carajás | PA | 17 | 33.808 | 0,5 | 15,4 |
| Santa Luzia | MA | 36 | 72.667 | 0,5 | 21,6 |
| Santa Cruz dos Milagres | PI | 2 | 4.019 | 0,5 | 22,9 |
| Aracati | CE | 37 | 74.547 | 0,5 | 4,5 |
| Barcelona | RN | 2 | 3.998 | 0,5 | 0,0 |
| Felipe Guerra | RN | 3 | 5.985 | 0,5 | 8,9 |
| Assunção | PB | 2 | 3.990 | 0,5 | 6,7 |
| Juazeirinho | PB | 9 | 18.171 | 0,5 | 35,9 |
| Mãe d'Água | PB | 2 | 4.009 | 0,5 | 95,7 |
| Marechal Deodoro | AL | 26 | 51.901 | 0,5 | 0,0 |
| Mutuípe | BA | 11 | 22.221 | 0,5 | 14,7 |
| Prado | BA | 14 | 28.174 | 0,5 | 0,2 |
| Água Comprida | MG | 1 | 1.999 | 0,5 | 0,4 |
| Guimarânia | MG | 4 | 8.039 | 0,5 | 0,0 |
| Marliéria | MG | 2 | 4.039 | 0,5 | 0,0 |
| Pedra do Indaiá | MG | 2 | 3.972 | 0,5 | 0,0 |
| Pedro Canário | ES | 13 | 26.184 | 0,5 | 0,0 |
| São José do Vale do Rio Preto | RJ | 11 | 21.795 | 0,5 | 0,0 |
| Alambari | SP | 3 | 6.025 | 0,5 | 0,0 |
| Ivaí | PR | 7 | 13.879 | 0,5 | 0,0 |
| Virmond | PR | 2 | 4.023 | 0,5 | 0,4 |
| Garuva | SC | 9 | 18.145 | 0,5 | 0,0 |
| Iraceminha | SC | 2 | 3.976 | 0,5 | 0,0 |
| São Pedro das Missões | RS | 1 | 2.009 | 0,5 | 0,0 |
| Ubiretama | RS | 1 | 2.015 | 0,5 | 0,0 |
| Campo Verde | MT | 22 | 44.041 | 0,5 | 8,4 |
| Diamantino | MT | 11 | 22.041 | 0,5 | 7,0 |
| Nortelândia | MT | 3 | 5.989 | 0,5 | 5,7 |
| Guaraíta | GO | 1 | 1.996 | 0,5 | 0,0 |
| Portelândia | GO | 2 | 4.011 | 0,5 | 100,0 |
| São João da Ponta | PA | 3 | 6.139 | 0,49 | 0,0 |
| Açailândia | MA | 55 | 112.445 | 0,49 | 3,1 |
| Coelho Neto | MA | 24 | 49.435 | 0,49 | 5,2 |
| Buriti dos Montes | PI | 4 | 8.244 | 0,49 | 33,1 |
| Solonópole | CE | 9 | 18.324 | 0,49 | 65,1 |
| Caraúbas | RN | 10 | 20.493 | 0,49 | 10,6 |
| João Câmara | RN | 17 | 34.955 | 0,49 | 0,0 |
| Cuité | PB | 10 | 20.338 | 0,49 | 11,9 |
| Venturosa | PE | 9 | 18.482 | 0,49 | 0,2 |
| Riachuelo | SE | 5 | 10.213 | 0,49 | 0,0 |
| Barra da Estiva | BA | 10 | 20.598 | 0,49 | 1,7 |
| Boninal | BA | 7 | 14.368 | 0,49 | 2,6 |
| Dom Macedo Costa | BA | 2 | 4.058 | 0,49 | 0,0 |
| Ibicuí | BA | 8 | 16.198 | 0,49 | 1,2 |
| Ibiquera | BA | 2 | 4.044 | 0,49 | 27,3 |
| Nova Canaã | BA | 8 | 16.462 | 0,49 | 8,3 |
| Pai Pedro | MG | 3 | 6.089 | 0,49 | 18,3 |
| Passa-Vinte | MG | 1 | 2.039 | 0,49 | 0,0 |
| Santa Bárbara do Leste | MG | 4 | 8.147 | 0,49 | 0,0 |
| Anhumas | SP | 2 | 4.115 | 0,49 | 0,0 |
| Nova Santa Rosa | PR | 4 | 8.219 | 0,49 | 0,0 |
| Rio Branco do Ivaí | PR | 2 | 4.096 | 0,49 | 0,0 |
| Cerro Grande do Sul | RS | 6 | 12.239 | 0,49 | 100,0 |
| Santana da Boa Vista | RS | 4 | 8.098 | 0,49 | 100,0 |
| Barra do Bugres | MT | 17 | 34.966 | 0,49 | 12,2 |
| Goiás | GO | 11 | 22.645 | 0,49 | 1,2 |
| Vila Boa | GO | 3 | 6.171 | 0,49 | 0,3 |
| Guajará-Mirim | RO | 22 | 46.174 | 0,48 | 9,1 |
| Cacaulândia | RO | 3 | 6.230 | 0,48 | 44,3 |
| Jordão | AC | 4 | 8.317 | 0,48 | 55,0 |
| Jardim do Seridó | RN | 6 | 12.396 | 0,48 | 0,0 |
| Upanema | RN | 7 | 14.659 | 0,48 | 33,6 |
| Desterro | PB | 4 | 8.297 | 0,48 | 100,0 |
| Prata | PB | 2 | 4.209 | 0,48 | 98,2 |
| Branquinha | AL | 5 | 10.494 | 0,48 | 3,2 |
| Iguaí | BA | 13 | 26.916 | 0,48 | 14,0 |
| Ipiaú | BA | 22 | 45.873 | 0,48 | 0,1 |
| Vereda | BA | 3 | 6.205 | 0,48 | 4,4 |
| Guaraciaba | MG | 5 | 10.324 | 0,48 | 0,6 |
| Ibiaí | MG | 4 | 8.395 | 0,48 | 7,6 |
| Ubaí | MG | 6 | 12.533 | 0,48 | 0,4 |
| Marechal Floriano | ES | 8 | 16.694 | 0,48 | 0,0 |
| Montanha | ES | 9 | 18.833 | 0,48 | 0,0 |
| São Mateus | ES | 63 | 130.611 | 0,48 | 0,0 |
| Santa Maria Madalena | RJ | 5 | 10.404 | 0,48 | 3,3 |
| Aparecida d'Oeste | SP | 2 | 4.196 | 0,48 | 0,0 |
| Cananéia | SP | 6 | 12.540 | 0,48 | 1,6 |
| Guaraçaí | SP | 4 | 8.323 | 0,48 | 0,8 |
| Monte Castelo | SP | 2 | 4.166 | 0,48 | 0,0 |
| Guaraniaçu | PR | 6 | 12.472 | 0,48 | 2,0 |
| São José do Cerrito | SC | 4 | 8.295 | 0,48 | 0,0 |
| Alpestre | RS | 3 | 6.258 | 0,48 | 0,0 |
| Itapuca | RS | 1 | 2.094 | 0,48 | 6,6 |
| São José do Inhacorá | RS | 1 | 2.073 | 0,48 | 0,0 |
| Matupá | MT | 8 | 16.566 | 0,48 | 19,4 |
| Davinópolis | GO | 1 | 2.094 | 0,48 | 2,5 |
| Inaciolândia | GO | 3 | 6.194 | 0,48 | 0,1 |
| Redenção | PA | 40 | 84.787 | 0,47 | 1,6 |
| Araguaçu | TO | 4 | 8.517 | 0,47 | 4,6 |
| Rio da Conceição | TO | 1 | 2.130 | 0,47 | 0,0 |
| Arari | MA | 14 | 29.848 | 0,47 | 5,0 |
| Patos do Piauí | PI | 3 | 6.392 | 0,47 | 22,6 |
| Alto Santo | CE | 8 | 17.146 | 0,47 | 50,4 |
| Porto do Mangue | RN | 3 | 6.437 | 0,47 | 3,4 |
| São José de Caiana | PB | 3 | 6.359 | 0,47 | 100,0 |
| Betânia | PE | 6 | 12.719 | 0,47 | 25,6 |
| Boa Nova | BA | 6 | 12.633 | 0,47 | 8,6 |
| Jandaíra | BA | 5 | 10.709 | 0,47 | 0,7 |
| Ouriçangas | BA | 4 | 8.564 | 0,47 | 0,0 |
| Bom Jesus da Penha | MG | 2 | 4.217 | 0,47 | 0,0 |
| Candeias | MG | 7 | 14.886 | 0,47 | 0,0 |
| Jequitinhonha | MG | 12 | 25.391 | 0,47 | 9,0 |
| Piedade de Caratinga | MG | 4 | 8.566 | 0,47 | 0,0 |
| Ponto Chique | MG | 2 | 4.261 | 0,47 | 7,8 |
| Santa Helena de Minas | MG | 3 | 6.366 | 0,47 | 0,4 |
| Alegre | ES | 14 | 30.084 | 0,47 | 0,0 |
| Boa Esperança | ES | 7 | 15.037 | 0,47 | 0,0 |
| Arandu | SP | 3 | 6.357 | 0,47 | 0,0 |
| Marinópolis | SP | 1 | 2.112 | 0,47 | 0,0 |
| Pardinho | SP | 3 | 6.435 | 0,47 | 0,0 |
| Santa Clara d'Oeste | SP | 1 | 2.115 | 0,47 | 0,0 |
| Piên | PR | 6 | 12.746 | 0,47 | 0,0 |
| Santo Antônio do Paraíso | PR | 1 | 2.106 | 0,47 | 0,0 |
| Bom Jesus do Oeste | SC | 1 | 2.142 | 0,47 | 0,0 |
| Luiz Alves | SC | 6 | 12.859 | 0,47 | 0,0 |
| Santa Rosa de Lima | SC | 1 | 2.142 | 0,47 | 1,5 |
| Entre-Ijuís | RS | 4 | 8.475 | 0,47 | 0,1 |
| Jóia | RS | 4 | 8.560 | 0,47 | 3,3 |
| Porto Murtinho | MS | 8 | 17.131 | 0,47 | 18,8 |
| Alto Horizonte | GO | 3 | 6.414 | 0,47 | 0,0 |
| Serranópolis | GO | 4 | 8.544 | 0,47 | 7,7 |
| Santa Rosa do Purus | AC | 3 | 6.540 | 0,46 | 55,9 |
| Esperantinópolis | MA | 8 | 17.241 | 0,46 | 0,0 |
| Farias Brito | CE | 9 | 19.450 | 0,46 | 2,2 |
| Independência | CE | 12 | 26.178 | 0,46 | 100,0 |
| Tabuleiro do Norte | CE | 14 | 30.697 | 0,46 | 2,5 |
| Olho d'Água | PB | 3 | 6.526 | 0,46 | 99,6 |
| Carnaíba | PE | 9 | 19.551 | 0,46 | 15,8 |
| Aracatu | BA | 6 | 13.135 | 0,46 | 31,0 |
| Ibicoara | BA | 9 | 19.571 | 0,46 | 0,0 |
| Jaguaquara | BA | 25 | 54.423 | 0,46 | 1,3 |
| Dona Eusébia | MG | 3 | 6.572 | 0,46 | 0,0 |
| Gonçalves | MG | 2 | 4.350 | 0,46 | 0,0 |
| Itamarati de Minas | MG | 2 | 4.355 | 0,46 | 1,2 |
| Tiros | MG | 3 | 6.480 | 0,46 | 6,1 |
| Parisi | SP | 1 | 2.161 | 0,46 | 0,0 |
| Canguçu | RS | 26 | 56.045 | 0,46 | 8,3 |
| Catuípe | RS | 4 | 8.774 | 0,46 | 0,0 |
| Vila Maria | RS | 2 | 4.358 | 0,46 | 0,0 |
| Alto Taquari | MT | 5 | 10.847 | 0,46 | 1,0 |
| Alvorada do Norte | GO | 4 | 8.660 | 0,46 | 2,0 |
| Santana | AP | 55 | 121.364 | 0,45 | 0,8 |
| Bernardo Sayão | TO | 2 | 4.459 | 0,45 | 0,9 |
| Miranorte | TO | 6 | 13.434 | 0,45 | 2,2 |
| Alto Parnaíba | MA | 5 | 11.190 | 0,45 | 28,5 |
| Cajapió | MA | 5 | 11.177 | 0,45 | 1,7 |
| Poção de Pedras | MA | 8 | 17.873 | 0,45 | 1,4 |
| Wall Ferraz | PI | 2 | 4.462 | 0,45 | 19,9 |
| Morada Nova | CE | 28 | 61.890 | 0,45 | 32,1 |
| Quixeré | CE | 10 | 22.149 | 0,45 | 0,0 |
| Rodolfo Fernandes | RN | 2 | 4.467 | 0,45 | 99,9 |
| Amparo | PB | 1 | 2.238 | 0,45 | 100,0 |
| Boqueirão | PB | 8 | 17.804 | 0,45 | 22,9 |
| Pilões | PB | 3 | 6.635 | 0,45 | 0,0 |
| São João do Tigre | PB | 2 | 4.422 | 0,45 | 39,5 |
| Aratuípe | BA | 4 | 8.825 | 0,45 | 0,0 |
| Caravelas | BA | 10 | 22.016 | 0,45 | 2,5 |
| Manoel Vitorino | BA | 6 | 13.240 | 0,45 | 48,2 |
| Carangola | MG | 15 | 33.000 | 0,45 | 0,0 |
| Jaboticatubas | MG | 9 | 20.143 | 0,45 | 0,4 |
| Paineiras | MG | 2 | 4.486 | 0,45 | 5,6 |
| São Sebastião do Rio Verde | MG | 1 | 2.241 | 0,45 | 0,0 |
| Cantagalo | RJ | 9 | 20.172 | 0,45 | 0,0 |
| Juquitiba | SP | 14 | 31.444 | 0,45 | 0,0 |
| Cerro Azul | PR | 8 | 17.779 | 0,45 | 0,0 |
| Rio Bonito do Iguaçu | PR | 6 | 13.269 | 0,45 | 8,7 |
| Arvoredo | SC | 1 | 2.240 | 0,45 | 0,0 |
| Lacerdópolis | SC | 1 | 2.246 | 0,45 | 0,0 |
| Santa Helena | SC | 1 | 2.223 | 0,45 | 0,0 |
| Crissiumal | RS | 6 | 13.448 | 0,45 | 0,0 |
| Inhacorá | RS | 1 | 2.226 | 0,45 | 0,0 |
| Novo Tiradentes | RS | 1 | 2.211 | 0,45 | 0,0 |
| Juti | MS | 3 | 6.712 | 0,45 | 1,2 |
| Itanhangá | MT | 3 | 6.737 | 0,45 | 100,0 |
| Nova Lacerda | MT | 3 | 6.640 | 0,45 | 98,4 |
| Paranaíta | MT | 5 | 11.225 | 0,45 | 6,5 |
| Pontal do Araguaia | MT | 3 | 6.711 | 0,45 | 6,0 |
| Bonópolis | GO | 2 | 4.405 | 0,45 | 16,8 |
| Montividiu do Norte | GO | 2 | 4.479 | 0,45 | 16,1 |
| São Miguel do Araguaia | GO | 10 | 21.993 | 0,45 | 4,0 |
| São Francisco do Guaporé | RO | 9 | 20.266 | 0,44 | 8,5 |
| Itapiranga | AM | 4 | 9.148 | 0,44 | 4,8 |
| Medicilândia | PA | 14 | 31.597 | 0,44 | 16,1 |
| Rio Maria | PA | 8 | 18.193 | 0,44 | 9,2 |
| São Geraldo do Araguaia | PA | 11 | 24.847 | 0,44 | 11,2 |
| Cachoeirinha | TO | 1 | 2.275 | 0,44 | 0,0 |
| Duque Bacelar | MA | 5 | 11.349 | 0,44 | 10,5 |
| Florânia | RN | 4 | 9.116 | 0,44 | 12,9 |
| Buerarema | BA | 8 | 18.349 | 0,44 | 0,1 |
| Eunápolis | BA | 50 | 113.380 | 0,44 | 0,1 |
| Itagimirim | BA | 3 | 6.869 | 0,44 | 1,4 |
| Uruçuca | BA | 9 | 20.519 | 0,44 | 2,0 |
| Canaã | MG | 2 | 4.563 | 0,44 | 0,0 |
| Cipotânea | MG | 3 | 6.787 | 0,44 | 0,0 |
| Estiva | MG | 5 | 11.354 | 0,44 | 0,0 |
| Eugenópolis | MG | 5 | 11.275 | 0,44 | 1,4 |
| Galiléia | MG | 3 | 6.817 | 0,44 | 0,0 |
| Guanhães | MG | 15 | 34.319 | 0,44 | 0,2 |
| José Gonçalves de Minas | MG | 2 | 4.501 | 0,44 | 12,6 |
| Ladainha | MG | 8 | 18.111 | 0,44 | 19,8 |
| Rosário da Limeira | MG | 2 | 4.594 | 0,44 | 0,0 |
| Nova Venécia | ES | 22 | 50.110 | 0,44 | 0,0 |
| Lourdes | SP | 1 | 2.289 | 0,44 | 0,0 |
| Piquete | SP | 6 | 13.657 | 0,44 | 0,0 |
| Tejupá | SP | 2 | 4.532 | 0,44 | 0,0 |
| Iguatu | PR | 1 | 2.256 | 0,44 | 0,1 |
| Iracema do Oeste | PR | 1 | 2.288 | 0,44 | 0,0 |
| Tunápolis | SC | 2 | 4.543 | 0,44 | 0,0 |
| Roque Gonzales | RS | 3 | 6.847 | 0,44 | 6,8 |
| Bandeirantes | MS | 3 | 6.788 | 0,44 | 11,4 |
| Camapuã | MS | 6 | 13.711 | 0,44 | 8,0 |
| Juína | MT | 18 | 40.997 | 0,44 | 7,4 |
| Santa Carmem | MT | 2 | 4.525 | 0,44 | 100,0 |
| Itapaci | GO | 10 | 22.981 | 0,44 | 0,2 |
| Morro Agudo de Goiás | GO | 1 | 2.248 | 0,44 | 0,0 |
| Novo Planalto | GO | 2 | 4.495 | 0,44 | 7,2 |
| Pilar de Goiás | GO | 1 | 2.253 | 0,44 | 0,9 |
| Epitaciolândia | AC | 8 | 18.411 | 0,43 | 4,4 |
| Tucuruí | PA | 49 | 113.659 | 0,43 | 2,8 |
| Amapá do Maranhão | MA | 3 | 6.962 | 0,43 | 100,0 |
| Estreito | MA | 18 | 41.946 | 0,43 | 6,9 |
| Joselândia | MA | 7 | 16.168 | 0,43 | 2,7 |
| Nova Iorque | MA | 2 | 4.683 | 0,43 | 100,0 |
| Lagoa do Barro do Piauí | PI | 2 | 4.655 | 0,43 | 46,8 |
| Ubajara | CE | 15 | 34.792 | 0,43 | 1,8 |
| São José de Espinharas | PB | 2 | 4.682 | 0,43 | 7,9 |
| Serra Redonda | PB | 3 | 7.041 | 0,43 | 0,0 |
| Jaqueira | PE | 5 | 11.656 | 0,43 | 4,1 |
| Ibitiara | BA | 7 | 16.339 | 0,43 | 13,1 |
| Araçaí | MG | 1 | 2.347 | 0,43 | 0,0 |
| Bertópolis | MG | 2 | 4.604 | 0,43 | 7,4 |
| Cachoeira de Minas | MG | 5 | 11.547 | 0,43 | 0,0 |
| Central de Minas | MG | 3 | 7.032 | 0,43 | 0,0 |
| Conquista | MG | 3 | 6.939 | 0,43 | 0,0 |
| Coronel Murta | MG | 4 | 9.222 | 0,43 | 3,7 |
| Estrela Dalva | MG | 1 | 2.343 | 0,43 | 0,0 |
| Joaquim Felício | MG | 2 | 4.695 | 0,43 | 7,9 |
| Santo Antônio do Amparo | MG | 8 | 18.525 | 0,43 | 0,0 |
| São Roque de Minas | MG | 3 | 7.051 | 0,43 | 6,7 |
| Rio Novo do Sul | ES | 5 | 11.622 | 0,43 | 0,0 |
| Monteiro Lobato | SP | 2 | 4.653 | 0,43 | 0,0 |
| Nova Guataporanga | SP | 1 | 2.316 | 0,43 | 0,0 |
| Pontalinda | SP | 2 | 4.628 | 0,43 | 0,0 |
| São Bernardino | SC | 1 | 2.336 | 0,43 | 0,0 |
| Coqueiros do Sul | RS | 1 | 2.306 | 0,43 | 0,0 |
| Palmitinho | RS | 3 | 7.057 | 0,43 | 0,0 |
| Porto Lucena | RS | 2 | 4.678 | 0,43 | 0,0 |
| Unistalda | RS | 1 | 2.338 | 0,43 | 100,0 |
| Costa Rica | MS | 9 | 20.823 | 0,43 | 0,0 |
| Juara | MT | 15 | 34.974 | 0,43 | 9,7 |
| Vila Bela da Santíssima Trindade | MT | 7 | 16.128 | 0,43 | 37,2 |
| Itaguari | GO | 2 | 4.676 | 0,43 | 0,0 |
| Sampaio | TO | 2 | 4.711 | 0,42 | 0,0 |
| Santa Rita do Tocantins | TO | 1 | 2.365 | 0,42 | 23,4 |
| Buriti | MA | 12 | 28.678 | 0,42 | 19,9 |
| Formosa da Serra Negra | MA | 8 | 19.089 | 0,42 | 32,2 |
| Matões do Norte | MA | 7 | 16.745 | 0,42 | 9,2 |
| Presidente Dutra | MA | 20 | 47.804 | 0,42 | 0,0 |
| Ererê | CE | 3 | 7.198 | 0,42 | 100,0 |
| Várzea Alegre | CE | 17 | 40.721 | 0,42 | 12,5 |
| Antônio Martins | RN | 3 | 7.145 | 0,42 | 99,5 |
| Conceição | PB | 8 | 18.982 | 0,42 | 30,4 |
| Monte Horebe | PB | 2 | 4.816 | 0,42 | 100,0 |
| Feliz Deserto | AL | 2 | 4.754 | 0,42 | 0,0 |
| Miguel Calmon | BA | 11 | 26.023 | 0,42 | 0,1 |
| Santa Rita de Cássia | BA | 12 | 28.338 | 0,42 | 13,9 |
| Amparo do Serra | MG | 2 | 4.713 | 0,42 | 0,0 |
| Carlos Chagas | MG | 8 | 18.837 | 0,42 | 3,2 |
| Matipó | MG | 8 | 18.908 | 0,42 | 0,0 |
| Monte Santo de Minas | MG | 9 | 21.524 | 0,42 | 0,0 |
| Natércia | MG | 2 | 4.730 | 0,42 | 3,8 |
| Poço Fundo | MG | 7 | 16.791 | 0,42 | 0,0 |
| Reduto | MG | 3 | 7.154 | 0,42 | 0,0 |
| Ressaquinha | MG | 2 | 4.808 | 0,42 | 0,0 |
| Mimoso do Sul | ES | 11 | 26.153 | 0,42 | 0,0 |
| Caconde | SP | 8 | 18.985 | 0,42 | 0,2 |
| Gastão Vidigal | SP | 2 | 4.808 | 0,42 | 0,0 |
| Antonina | PR | 8 | 18.980 | 0,42 | 1,5 |
| Marmeleiro | PR | 6 | 14.367 | 0,42 | 0,0 |
| Rosário do Ivaí | PR | 2 | 4.786 | 0,42 | 2,4 |
| Campo Alegre | SC | 5 | 11.978 | 0,42 | 0,0 |
| Romelândia | SC | 2 | 4.786 | 0,42 | 0,0 |
| Eugênio de Castro | RS | 1 | 2.396 | 0,42 | 0,0 |
| Ibiaçá | RS | 2 | 4.709 | 0,42 | 3,7 |
| Tabaí | RS | 2 | 4.719 | 0,42 | 0,0 |
| Aquidauana | MS | 20 | 47.871 | 0,42 | 4,2 |
| Caiapônia | GO | 8 | 18.913 | 0,42 | 8,2 |
| Palmelo | GO | 1 | 2.381 | 0,42 | 0,0 |
| Pimenta Bueno | RO | 15 | 36.660 | 0,41 | 7,5 |
| Abaetetuba | PA | 64 | 157.698 | 0,41 | 12,2 |
| Aldeias Altas | MA | 11 | 26.532 | 0,41 | 25,5 |
| São João da Varjota | PI | 2 | 4.840 | 0,41 | 0,1 |
| Timbaúba dos Batistas | RN | 1 | 2.414 | 0,41 | 0,0 |
| Catingueira | PB | 2 | 4.932 | 0,41 | 100,0 |
| Caturité | PB | 2 | 4.852 | 0,41 | 11,6 |
| Livramento | PB | 3 | 7.256 | 0,41 | 100,0 |
| Exu | PE | 13 | 31.825 | 0,41 | 10,7 |
| Ipupiara | BA | 4 | 9.865 | 0,41 | 0,7 |
| Pedrão | BA | 3 | 7.347 | 0,41 | 0,0 |
| Caldas | MG | 6 | 14.480 | 0,41 | 4,7 |
| Campina Verde | MG | 8 | 19.745 | 0,41 | 0,3 |
| Canápolis | MG | 5 | 12.150 | 0,41 | 0,0 |
| Indaiabira | MG | 3 | 7.351 | 0,41 | 22,6 |
| Jacinto | MG | 5 | 12.326 | 0,41 | 4,0 |
| Josenópolis | MG | 2 | 4.867 | 0,41 | 29,3 |
| Leme do Prado | MG | 2 | 4.918 | 0,41 | 0,1 |
| Morro da Garça | MG | 1 | 2.462 | 0,41 | 1,0 |
| Paulistas | MG | 2 | 4.830 | 0,41 | 0,0 |
| Rio Paranaíba | MG | 5 | 12.313 | 0,41 | 1,5 |
| São Francisco do Glória | MG | 2 | 4.844 | 0,41 | 0,0 |
| Alfredo Chaves | ES | 6 | 14.601 | 0,41 | 0,0 |
| Embaúba | SP | 1 | 2.452 | 0,41 | 0,0 |
| Lavrinhas | SP | 3 | 7.260 | 0,41 | 0,0 |
| Mirassolândia | SP | 2 | 4.871 | 0,41 | 0,0 |
| Patrocínio Paulista | SP | 6 | 14.670 | 0,41 | 0,0 |
| Brunópolis | SC | 1 | 2.420 | 0,41 | 0,8 |
| Ouro | SC | 3 | 7.295 | 0,41 | 0,0 |
| Urupema | SC | 1 | 2.465 | 0,41 | 1,4 |
| Caibaté | RS | 2 | 4.846 | 0,41 | 0,0 |
| Dezesseis de Novembro | RS | 1 | 2.427 | 0,41 | 1,5 |
| Cáceres | MT | 39 | 94.376 | 0,41 | 4,9 |
| Crixás | GO | 7 | 16.949 | 0,41 | 1,4 |
| Hidrolândia | GO | 9 | 21.706 | 0,41 | 0,0 |
| Mundo Novo | GO | 2 | 4.887 | 0,41 | 5,8 |
| Ipixuna | AM | 12 | 29.689 | 0,4 | 34,2 |
| Manaquiri | AM | 13 | 32.105 | 0,4 | 20,1 |
| Dom Eliseu | PA | 24 | 59.719 | 0,4 | 9,3 |
| Paragominas | PA | 45 | 113.145 | 0,4 | 8,3 |
| Colinas do Tocantins | TO | 14 | 35.424 | 0,4 | 0,3 |
| Codó | MA | 49 | 122.859 | 0,4 | 3,4 |
| Ibicuitinga | CE | 5 | 12.525 | 0,4 | 9,5 |
| Icapuí | CE | 8 | 19.934 | 0,4 | 1,1 |
| Japi | RN | 2 | 5.055 | 0,4 | 6,6 |
| Santa Cruz | RN | 16 | 39.674 | 0,4 | 0,0 |
| Santa Cruz da Baixa Verde | PE | 5 | 12.592 | 0,4 | 0,0 |
| Terra Nova | PE | 4 | 10.096 | 0,4 | 0,0 |
| Medeiros Neto | BA | 9 | 22.688 | 0,4 | 3,2 |
| Muniz Ferreira | BA | 3 | 7.422 | 0,4 | 0,0 |
| Ourolândia | BA | 7 | 17.451 | 0,4 | 4,6 |
| Santa Cruz Cabrália | BA | 11 | 27.778 | 0,4 | 0,6 |
| Teodoro Sampaio | BA | 3 | 7.425 | 0,4 | 0,0 |
| Catuti | MG | 2 | 4.986 | 0,4 | 6,3 |
| Claro dos Poções | MG | 3 | 7.551 | 0,4 | 11,2 |
| Ibertioga | MG | 2 | 5.021 | 0,4 | 0,0 |
| Moema | MG | 3 | 7.517 | 0,4 | 0,0 |
| Pedra Dourada | MG | 1 | 2.504 | 0,4 | 0,0 |
| Piedade dos Gerais | MG | 2 | 4.982 | 0,4 | 0,0 |
| São Domingos do Prata | MG | 7 | 17.359 | 0,4 | 0,0 |
| São João Batista do Glória | MG | 3 | 7.453 | 0,4 | 0,0 |
| Ubaporanga | MG | 5 | 12.471 | 0,4 | 0,0 |
| São Gabriel da Palha | ES | 15 | 37.947 | 0,4 | 0,0 |
| Paty do Alferes | RJ | 11 | 27.769 | 0,4 | 0,0 |
| Ubatuba | SP | 36 | 90.799 | 0,4 | 0,0 |
| São João do Triunfo | PR | 6 | 15.120 | 0,4 | 0,0 |
| Coronel Barros | RS | 1 | 2.519 | 0,4 | 0,0 |
| Planalto | RS | 4 | 10.084 | 0,4 | 0,0 |
| Guia Lopes da Laguna | MS | 4 | 9.895 | 0,4 | 2,3 |
| Rio Brilhante | MS | 15 | 37.514 | 0,4 | 2,6 |
| Pontes e Lacerda | MT | 18 | 45.436 | 0,4 | 5,8 |
| Adelândia | GO | 1 | 2.516 | 0,4 | 0,0 |
| Campinorte | GO | 5 | 12.626 | 0,4 | 2,5 |
| Diorama | GO | 1 | 2.484 | 0,4 | 10,0 |
| Itapirapuã | GO | 2 | 4.997 | 0,4 | 3,8 |
| Jesúpolis | GO | 1 | 2.490 | 0,4 | 0,0 |
| Paranaiguara | GO | 4 | 10.057 | 0,4 | 2,6 |
| São Felipe D'Oeste | RO | 2 | 5.172 | 0,39 | 2,2 |
| Acrelândia | AC | 6 | 15.256 | 0,39 | 12,6 |
| Piçarra | PA | 5 | 12.981 | 0,39 | 19,2 |
| Araguaína | TO | 71 | 180.470 | 0,39 | 1,0 |
| Carmolândia | TO | 1 | 2.580 | 0,39 | 1,6 |
| Fortaleza do Tabocão | TO | 1 | 2.589 | 0,39 | 0,1 |
| Marianópolis do Tocantins | TO | 2 | 5.175 | 0,39 | 1,2 |
| Governador Nunes Freire | MA | 10 | 25.577 | 0,39 | 25,7 |
| Vargem Grande | MA | 22 | 56.510 | 0,39 | 17,9 |
| Aroeiras do Itaim | PI | 1 | 2.551 | 0,39 | 0,1 |
| Porto de Pedras | AL | 3 | 7.786 | 0,39 | 13,7 |
| Brotas de Macaúbas | BA | 4 | 10.231 | 0,39 | 20,7 |
| Formosa do Rio Preto | BA | 10 | 25.591 | 0,39 | 4,1 |
| São Desidério | BA | 13 | 33.742 | 0,39 | 8,3 |
| Bocaina de Minas | MG | 2 | 5.090 | 0,39 | 0,0 |
| Capitão Enéas | MG | 6 | 15.234 | 0,39 | 1,6 |
| Centralina | MG | 4 | 10.350 | 0,39 | 0,0 |
| Dionísio | MG | 3 | 7.729 | 0,39 | 0,0 |
| Dom Cavati | MG | 2 | 5.072 | 0,39 | 0,0 |
| Joaíma | MG | 6 | 15.432 | 0,39 | 1,3 |
| Ninheira | MG | 4 | 10.295 | 0,39 | 16,7 |
| Governador Lindenberg | ES | 5 | 12.709 | 0,39 | 0,0 |
| Jacupiranga | SP | 7 | 17.866 | 0,39 | 0,0 |
| São João das Duas Pontes | SP | 1 | 2.568 | 0,39 | 0,0 |
| Sud Mennucci | SP | 3 | 7.718 | 0,39 | 0,0 |
| Catanduvas | PR | 4 | 10.189 | 0,39 | 0,2 |
| Diamante do Norte | PR | 2 | 5.087 | 0,39 | 0,0 |
| Manfrinópolis | PR | 1 | 2.571 | 0,39 | 0,0 |
| Barra Funda | RS | 1 | 2.539 | 0,39 | 0,0 |
| Encruzilhada do Sul | RS | 10 | 25.877 | 0,39 | 20,1 |
| Lajeado do Bugre | RS | 1 | 2.564 | 0,39 | 0,0 |
| Santo Antônio das Missões | RS | 4 | 10.175 | 0,39 | 76,7 |
| Araguapaz | GO | 3 | 7.770 | 0,39 | 1,3 |
| Pontalina | GO | 7 | 17.819 | 0,39 | 0,9 |
| Theobroma | RO | 4 | 10.444 | 0,38 | 99,2 |
| Marabá | PA | 106 | 279.349 | 0,38 | 3,6 |
| Figueirópolis | TO | 2 | 5.263 | 0,38 | 5,0 |
| Coroatá | MA | 25 | 65.296 | 0,38 | 1,6 |
| Fartura do Piauí | PI | 2 | 5.307 | 0,38 | 13,4 |
| Landri Sales | PI | 2 | 5.295 | 0,38 | 99,7 |
| Saboeiro | CE | 6 | 15.819 | 0,38 | 100,0 |
| Governador Dix-Sept Rosado | RN | 5 | 13.037 | 0,38 | 1,2 |
| Borborema | PB | 2 | 5.263 | 0,38 | 0,0 |
| Dona Inês | PB | 4 | 10.453 | 0,38 | 19,7 |
| São Domingos do Cariri | PB | 1 | 2.615 | 0,38 | 100,0 |
| São Miguel dos Milagres | AL | 3 | 7.951 | 0,38 | 1,9 |
| Andaraí | BA | 5 | 13.143 | 0,38 | 14,0 |
| Macururé | BA | 3 | 7.824 | 0,38 | 28,1 |
| Santa Teresinha | BA | 4 | 10.405 | 0,38 | 0,0 |
| Baldim | MG | 3 | 7.826 | 0,38 | 0,9 |
| Bambuí | MG | 9 | 23.829 | 0,38 | 0,0 |
| Bom Repouso | MG | 4 | 10.547 | 0,38 | 1,0 |
| Dom Silvério | MG | 2 | 5.237 | 0,38 | 0,0 |
| Estrela do Sul | MG | 3 | 7.978 | 0,38 | 13,3 |
| Grão Mogol | MG | 6 | 15.836 | 0,38 | 18,7 |
| Japonvar | MG | 3 | 7.969 | 0,38 | 0,9 |
| Juruaia | MG | 4 | 10.563 | 0,38 | 0,0 |
| Lagoa Dourada | MG | 5 | 13.009 | 0,38 | 0,0 |
| Monte Belo | MG | 5 | 13.166 | 0,38 | 3,0 |
| Santa Maria do Salto | MG | 2 | 5.232 | 0,38 | 20,7 |
| Sapucaia | RJ | 7 | 18.228 | 0,38 | 0,0 |
| Borebi | SP | 1 | 2.653 | 0,38 | 0,0 |
| Lutécia | SP | 1 | 2.649 | 0,38 | 0,4 |
| Piedade | SP | 21 | 55.348 | 0,38 | 0,1 |
| Pratânia | SP | 2 | 5.261 | 0,38 | 0,0 |
| Cantagalo | PR | 5 | 13.317 | 0,38 | 12,7 |
| Prudentópolis | PR | 20 | 52.241 | 0,38 | 3,9 |
| Antônio Prado | RS | 5 | 13.050 | 0,38 | 0,0 |
| Sagrada Família | RS | 1 | 2.609 | 0,38 | 0,0 |
| Saldanha Marinho | RS | 1 | 2.650 | 0,38 | 0,2 |
| Curvelândia | MT | 2 | 5.219 | 0,38 | 0,0 |
| Itaguaru | GO | 2 | 5.237 | 0,38 | 0,0 |
| Orizona | GO | 6 | 15.615 | 0,38 | 1,2 |
| Panamá | GO | 1 | 2.615 | 0,38 | 4,5 |
| Presidente Médici | RO | 7 | 18.986 | 0,37 | 25,0 |
| Maracanã | PA | 11 | 29.473 | 0,37 | 0,0 |
| Quatipuru | PA | 5 | 13.608 | 0,37 | 0,0 |
| Pugmil | TO | 1 | 2.688 | 0,37 | 0,0 |
| Anajatuba | MA | 10 | 26.803 | 0,37 | 3,1 |
| Lago do Junco | MA | 4 | 10.840 | 0,37 | 0,1 |
| Caxingó | PI | 2 | 5.424 | 0,37 | 3,1 |
| Geminiano | PI | 2 | 5.442 | 0,37 | 0,0 |
| Cariús | CE | 7 | 18.699 | 0,37 | 89,0 |
| Jardim | CE | 10 | 27.174 | 0,37 | 2,9 |
| Pereiro | CE | 6 | 16.307 | 0,37 | 100,0 |
| Carnaúba dos Dantas | RN | 3 | 8.180 | 0,37 | 0,9 |
| Parelhas | RN | 8 | 21.477 | 0,37 | 2,0 |
| Tenente Ananias | RN | 4 | 10.786 | 0,37 | 100,0 |
| Boa Ventura | PB | 2 | 5.366 | 0,37 | 8,5 |
| Lastro | PB | 1 | 2.737 | 0,37 | 100,0 |
| Cumaru | PE | 4 | 10.906 | 0,37 | 0,0 |
| Tuparetama | PE | 3 | 8.202 | 0,37 | 99,6 |
| Almadina | BA | 2 | 5.464 | 0,37 | 0,9 |
| Paramirim | BA | 8 | 21.607 | 0,37 | 0,8 |
| Ubaitaba | BA | 7 | 19.056 | 0,37 | 0,0 |
| Alagoa | MG | 1 | 2.674 | 0,37 | 0,0 |
| Cachoeira Dourada | MG | 1 | 2.692 | 0,37 | 0,0 |
| Capitão Andrade | MG | 2 | 5.468 | 0,37 | 0,0 |
| Chapada Gaúcha | MG | 5 | 13.680 | 0,37 | 26,8 |
| Datas | MG | 2 | 5.410 | 0,37 | 0,0 |
| Mirabela | MG | 5 | 13.589 | 0,37 | 2,3 |
| Novo Oriente de Minas | MG | 4 | 10.755 | 0,37 | 3,3 |
| Rio Espera | MG | 2 | 5.474 | 0,37 | 0,0 |
| Tombos | MG | 3 | 8.022 | 0,37 | 0,0 |
| Bananal | SP | 4 | 10.945 | 0,37 | 0,0 |
| Macaubal | SP | 3 | 8.120 | 0,37 | 0,0 |
| Pinhal de São Bento | PR | 1 | 2.731 | 0,37 | 0,0 |
| Apiúna | SC | 4 | 10.743 | 0,37 | 0,0 |
| Bandeirante | SC | 1 | 2.678 | 0,37 | 0,0 |
| Jacuizinho | RS | 1 | 2.692 | 0,37 | 100,0 |
| Machadinho | RS | 2 | 5.445 | 0,37 | 0,0 |
| Nova Candelária | RS | 1 | 2.698 | 0,37 | 0,0 |
| Salvador das Missões | RS | 1 | 2.733 | 0,37 | 0,0 |
| Sertão | RS | 2 | 5.415 | 0,37 | 1,7 |
| Três Arroios | RS | 1 | 2.668 | 0,37 | 0,0 |
| Alcinópolis | MS | 2 | 5.343 | 0,37 | 14,0 |
| Angélica | MS | 4 | 10.780 | 0,37 | 1,1 |
| Porto dos Gaúchos | MT | 2 | 5.410 | 0,37 | 10,4 |
| Reserva do Cabaçal | MT | 1 | 2.732 | 0,37 | 6,2 |
| Porangatu | GO | 17 | 45.394 | 0,37 | 3,1 |
| Terezópolis de Goiás | GO | 3 | 8.043 | 0,37 | 0,0 |
| Vale do Anari | RO | 4 | 11.204 | 0,36 | 100,0 |
| Santa Maria do Pará | PA | 9 | 24.861 | 0,36 | 0,0 |
| Governador Eugênio Barros | MA | 6 | 16.828 | 0,36 | 5,2 |
| São João do Paraíso | MA | 4 | 11.177 | 0,36 | 36,2 |
| Campinas do Piauí | PI | 2 | 5.613 | 0,36 | 31,6 |
| Júlio Borges | PI | 2 | 5.627 | 0,36 | 100,0 |
| Afonso Bezerra | RN | 4 | 11.035 | 0,36 | 100,0 |
| Galinhos | RN | 1 | 2.786 | 0,36 | 18,0 |
| Monteiro | PB | 12 | 33.222 | 0,36 | 16,2 |
| Várzea | PB | 1 | 2.810 | 0,36 | 1,7 |
| Calçado | PE | 4 | 11.053 | 0,36 | 8,6 |
| Barra de São Miguel | AL | 3 | 8.322 | 0,36 | 0,0 |
| Carmópolis | SE | 6 | 16.634 | 0,36 | 0,0 |
| Itaquara | BA | 3 | 8.319 | 0,36 | 0,1 |
| Jaborandi | BA | 3 | 8.385 | 0,36 | 9,9 |
| Arantina | MG | 1 | 2.795 | 0,36 | 0,0 |
| Cabo Verde | MG | 5 | 14.075 | 0,36 | 0,0 |
| Conceição das Pedras | MG | 1 | 2.812 | 0,36 | 0,8 |
| Guiricema | MG | 3 | 8.392 | 0,36 | 0,0 |
| Mesquita | MG | 2 | 5.605 | 0,36 | 0,0 |
| Pains | MG | 3 | 8.283 | 0,36 | 0,0 |
| Piau | MG | 1 | 2.748 | 0,36 | 0,0 |
| Vila Valério | ES | 5 | 14.080 | 0,36 | 0,0 |
| Engenheiro Paulo de Frontin | RJ | 5 | 14.002 | 0,36 | 0,0 |
| Estrela do Norte | SP | 1 | 2.766 | 0,36 | 0,0 |
| Pariquera-Açu | SP | 7 | 19.648 | 0,36 | 0,0 |
| Riversul | SP | 2 | 5.524 | 0,36 | 0,0 |
| Anahy | PR | 1 | 2.801 | 0,36 | 0,0 |
| Araruna | PR | 5 | 13.970 | 0,36 | 0,0 |
| Pato Bragado | PR | 2 | 5.610 | 0,36 | 0,0 |
| São Jerônimo da Serra | PR | 4 | 11.170 | 0,36 | 0,0 |
| Cunha Porã | SC | 4 | 11.086 | 0,36 | 0,0 |
| Santa Rosa do Sul | SC | 3 | 8.358 | 0,36 | 0,0 |
| Coxilha | RS | 1 | 2.756 | 0,36 | 0,0 |
| Gaurama | RS | 2 | 5.534 | 0,36 | 0,0 |
| Vista Alegre | RS | 1 | 2.752 | 0,36 | 0,0 |
| Araputanga | MT | 6 | 16.822 | 0,36 | 0,2 |
| Guarantã do Norte | MT | 13 | 35.816 | 0,36 | 10,1 |
| Indiavaí | MT | 1 | 2.752 | 0,36 | 6,6 |
| Juscimeira | MT | 4 | 11.221 | 0,36 | 100,0 |
| Novo Horizonte do Oeste | RO | 3 | 8.538 | 0,35 | 8,6 |
| Igarapé Grande | MA | 4 | 11.320 | 0,35 | 0,0 |
| Itapecuru Mirim | MA | 24 | 68.203 | 0,35 | 0,2 |
| Nina Rodrigues | MA | 5 | 14.454 | 0,35 | 7,1 |
| Sambaíba | MA | 2 | 5.671 | 0,35 | 27,7 |
| Caldeirão Grande do Piauí | PI | 2 | 5.776 | 0,35 | 44,5 |
| Itainópolis | PI | 4 | 11.530 | 0,35 | 6,4 |
| Lagoinha do Piauí | PI | 1 | 2.845 | 0,35 | 0,0 |
| Iracema | CE | 5 | 14.297 | 0,35 | 100,0 |
| Jaguaribara | CE | 4 | 11.401 | 0,35 | 100,0 |
| Mauriti | CE | 17 | 47.962 | 0,35 | 21,7 |
| Francisco Dantas | RN | 1 | 2.824 | 0,35 | 100,0 |
| Serra de São Bento | RN | 2 | 5.762 | 0,35 | 0,0 |
| Aguiar | PB | 2 | 5.640 | 0,35 | 100,0 |
| Poção | PE | 4 | 11.302 | 0,35 | 1,2 |
| Jequiá da Praia | AL | 4 | 11.580 | 0,35 | 0,0 |
| Quebrangulo | AL | 4 | 11.294 | 0,35 | 0,0 |
| Canavieiras | BA | 11 | 31.099 | 0,35 | 2,0 |
| Ibirapuã | BA | 3 | 8.637 | 0,35 | 0,9 |
| Arapuá | MG | 1 | 2.834 | 0,35 | 0,0 |
| Capitólio | MG | 3 | 8.632 | 0,35 | 0,0 |
| Divino | MG | 7 | 19.931 | 0,35 | 0,0 |
| Juvenília | MG | 2 | 5.724 | 0,35 | 0,2 |
| Manhuaçu | MG | 32 | 90.229 | 0,35 | 0,0 |
| Senhora de Oliveira | MG | 2 | 5.786 | 0,35 | 0,0 |
| Tarumirim | MG | 5 | 14.326 | 0,35 | 0,0 |
| Castelo | ES | 13 | 37.534 | 0,35 | 0,0 |
| São Domingos do Norte | ES | 3 | 8.638 | 0,35 | 0,0 |
| Guaimbê | SP | 2 | 5.765 | 0,35 | 0,0 |
| Salesópolis | SP | 6 | 17.139 | 0,35 | 0,0 |
| Itaúna do Sul | PR | 1 | 2.865 | 0,35 | 0,0 |
| Novo Itacolomi | PR | 1 | 2.844 | 0,35 | 0,0 |
| Anchieta | SC | 2 | 5.638 | 0,35 | 0,0 |
| Antônio Carlos | SC | 3 | 8.513 | 0,35 | 0,0 |
| Aurora | SC | 2 | 5.679 | 0,35 | 0,0 |
| Morro Grande | SC | 1 | 2.893 | 0,35 | 0,0 |
| Paverama | RS | 3 | 8.487 | 0,35 | 0,0 |
| Redentora | RS | 4 | 11.549 | 0,35 | 0,1 |
| São Paulo das Missões | RS | 2 | 5.790 | 0,35 | 0,0 |
| Tiradentes do Sul | RS | 2 | 5.704 | 0,35 | 0,0 |
| Guapó | GO | 5 | 14.209 | 0,35 | 0,0 |
| Ipiranga de Goiás | GO | 1 | 2.893 | 0,35 | 0,0 |
| Jaupaci | GO | 1 | 2.879 | 0,35 | 0,6 |
| São Félix do Piauí | PI | 1 | 2.942 | 0,34 | 9,0 |
| Carnaubal | CE | 6 | 17.606 | 0,34 | 10,7 |
| Santana do Cariri | CE | 6 | 17.700 | 0,34 | 13,5 |
| Tamboril | CE | 9 | 26.251 | 0,34 | 100,0 |
| Itaú | RN | 2 | 5.878 | 0,34 | 2,5 |
| Tenente Laurentino Cruz | RN | 2 | 5.952 | 0,34 | 6,9 |
| Gararu | SE | 4 | 11.604 | 0,34 | 0,4 |
| Governador Mangabeira | BA | 7 | 20.722 | 0,34 | 0,0 |
| Itapé | BA | 3 | 8.761 | 0,34 | 0,0 |
| São Félix | BA | 5 | 14.740 | 0,34 | 0,0 |
| Varzedo | BA | 3 | 8.838 | 0,34 | 0,0 |
| Coração de Jesus | MG | 9 | 26.602 | 0,34 | 3,5 |
| Fortuna de Minas | MG | 1 | 2.947 | 0,34 | 0,0 |
| Francisco Sá | MG | 9 | 26.277 | 0,34 | 17,0 |
| Frei Gaspar | MG | 2 | 5.880 | 0,34 | 14,8 |
| Guarani | MG | 3 | 8.911 | 0,34 | 1,1 |
| Montalvânia | MG | 5 | 14.877 | 0,34 | 1,6 |
| Muzambinho | MG | 7 | 20.569 | 0,34 | 0,2 |
| Pouso Alto | MG | 2 | 5.940 | 0,34 | 0,0 |
| Salinas | MG | 14 | 41.527 | 0,34 | 2,0 |
| São João do Manteninha | MG | 2 | 5.859 | 0,34 | 0,0 |
| Serra dos Aimorés | MG | 3 | 8.699 | 0,34 | 0,0 |
| Santa Teresa | ES | 8 | 23.590 | 0,34 | 0,0 |
| Américo de Campos | SP | 2 | 5.969 | 0,34 | 0,0 |
| Ibiúna | SP | 27 | 78.878 | 0,34 | 0,0 |
| Tambaú | SP | 8 | 23.207 | 0,34 | 0,0 |
| Três Fronteiras | SP | 2 | 5.807 | 0,34 | 0,0 |
| Adrianópolis | PR | 2 | 5.919 | 0,34 | 7,6 |
| Laranjal | PR | 2 | 5.852 | 0,34 | 16,4 |
| Marilândia do Sul | PR | 3 | 8.836 | 0,34 | 0,0 |
| Paula Freitas | PR | 2 | 5.873 | 0,34 | 0,0 |
| Petrolândia | SC | 2 | 5.937 | 0,34 | 0,0 |
| Santa Terezinha | SC | 3 | 8.787 | 0,34 | 0,0 |
| Alecrim | RS | 2 | 5.950 | 0,34 | 0,0 |
| Arroio do Padre | RS | 1 | 2.937 | 0,34 | 0,0 |
| Esperança do Sul | RS | 1 | 2.926 | 0,34 | 0,0 |
| Garruchos | RS | 1 | 2.924 | 0,34 | 100,0 |
| Piratini | RS | 7 | 20.663 | 0,34 | 13,1 |
| São Lourenço do Sul | RS | 15 | 43.582 | 0,34 | 6,8 |
| São Pedro do Butiá | RS | 1 | 2.947 | 0,34 | 0,0 |
| São Vicente do Sul | RS | 3 | 8.721 | 0,34 | 19,0 |
| São Gabriel do Oeste | MS | 9 | 26.771 | 0,34 | 3,0 |
| Jauru | MT | 3 | 8.793 | 0,34 | 22,7 |
| Pedra Preta | MT | 6 | 17.626 | 0,34 | 95,9 |
| Mambaí | GO | 3 | 8.882 | 0,34 | 0,1 |
| Novo Brasil | GO | 1 | 2.913 | 0,34 | 0,0 |
| Nova Mamoré | RO | 10 | 30.583 | 0,33 | 45,7 |
| Parecis | RO | 2 | 6.074 | 0,33 | 40,6 |
| Bernardo do Mearim | MA | 2 | 6.043 | 0,33 | 0,0 |
| Gonçalves Dias | MA | 6 | 17.934 | 0,33 | 8,0 |
| Matões | MA | 11 | 33.782 | 0,33 | 8,7 |
| Mirador | MA | 7 | 21.015 | 0,33 | 35,4 |
| Montes Altos | MA | 3 | 9.160 | 0,33 | 6,9 |
| Tuntum | MA | 14 | 41.832 | 0,33 | 31,9 |
| Esperantina | PI | 13 | 39.737 | 0,33 | 1,1 |
| Novo Santo Antônio | PI | 1 | 2.991 | 0,33 | 23,9 |
| São João da Serra | PI | 2 | 6.139 | 0,33 | 22,6 |
| Russas | CE | 26 | 78.194 | 0,33 | 2,9 |
| Apodi | RN | 12 | 35.845 | 0,33 | 19,6 |
| Equador | RN | 2 | 6.045 | 0,33 | 9,4 |
| Camalaú | PB | 2 | 6.013 | 0,33 | 44,5 |
| Santa Luzia | PB | 5 | 15.382 | 0,33 | 1,6 |
| Soledade | PB | 5 | 14.989 | 0,33 | 99,8 |
| Floresta | PE | 11 | 32.873 | 0,33 | 22,9 |
| Iguaracy | PE | 4 | 12.228 | 0,33 | 23,6 |
| Santa Maria da Boa Vista | PE | 14 | 41.931 | 0,33 | 27,8 |
| Solidão | PE | 2 | 6.007 | 0,33 | 0,3 |
| Cafarnaum | BA | 6 | 18.437 | 0,33 | 0,4 |
| Dom Basílio | BA | 4 | 12.199 | 0,33 | 0,0 |
| Valença | BA | 32 | 96.562 | 0,33 | 2,6 |
| Alvinópolis | MG | 5 | 15.203 | 0,33 | 0,0 |
| Campestre | MG | 7 | 21.055 | 0,33 | 1,5 |
| Cascalho Rico | MG | 1 | 3.075 | 0,33 | 16,2 |
| Divisa Nova | MG | 2 | 6.011 | 0,33 | 0,0 |
| Fronteira | MG | 6 | 18.103 | 0,33 | 0,0 |
| Ibituruna | MG | 1 | 2.989 | 0,33 | 0,0 |
| Itinga | MG | 5 | 14.990 | 0,33 | 21,7 |
| Lagoa Formosa | MG | 6 | 18.052 | 0,33 | 0,5 |
| Mantena | MG | 9 | 27.644 | 0,33 | 0,2 |
| Patis | MG | 2 | 5.972 | 0,33 | 1,1 |
| Ponto dos Volantes | MG | 4 | 12.121 | 0,33 | 6,7 |
| Prados | MG | 3 | 9.031 | 0,33 | 0,0 |
| Rubelita | MG | 2 | 5.995 | 0,33 | 24,4 |
| Campina do Monte Alegre | SP | 2 | 6.024 | 0,33 | 0,0 |
| Paraibuna | SP | 6 | 18.222 | 0,33 | 0,0 |
| Pinhalzinho | SP | 5 | 15.207 | 0,33 | 0,0 |
| Piracaia | SP | 9 | 27.303 | 0,33 | 0,0 |
| São Miguel Arcanjo | SP | 11 | 32.931 | 0,33 | 0,0 |
| São Sebastião da Grama | SP | 4 | 12.182 | 0,33 | 0,0 |
| Ouro Verde do Oeste | PR | 2 | 5.996 | 0,33 | 0,0 |
| Bom Jesus | SC | 1 | 3.010 | 0,33 | 0,0 |
| Caibi | SC | 2 | 6.148 | 0,33 | 0,0 |
| Chapadão do Lageado | SC | 1 | 2.988 | 0,33 | 0,0 |
| Iporã do Oeste | SC | 3 | 8.996 | 0,33 | 0,0 |
| Lebon Régis | SC | 4 | 12.107 | 0,33 | 0,0 |
| Alto Feliz | RS | 1 | 3.028 | 0,33 | 0,0 |
| Dona Francisca | RS | 1 | 3.041 | 0,33 | 0,0 |
| Erebango | RS | 1 | 2.991 | 0,33 | 0,0 |
| Anaurilândia | MS | 3 | 9.035 | 0,33 | 6,4 |
| Caracol | MS | 2 | 6.116 | 0,33 | 2,2 |
| Coxim | MS | 11 | 33.543 | 0,33 | 1,5 |
| Alto Garças | MT | 4 | 12.030 | 0,33 | 100,0 |
| Guiratinga | MT | 5 | 15.141 | 0,33 | 99,9 |
| Ipameri | GO | 9 | 26.985 | 0,33 | 1,0 |
| Itaberaí | GO | 14 | 42.900 | 0,33 | 0,0 |
| Posse | GO | 12 | 36.900 | 0,33 | 1,4 |
| Sítio d'Abadia | GO | 1 | 2.989 | 0,33 | 4,4 |
| Urutaí | GO | 1 | 3.072 | 0,33 | 0,5 |
| Cachoeira do Piriá | PA | 11 | 33.900 | 0,32 | 75,6 |
| Macapá | AP | 161 | 503.327 | 0,32 | 3,1 |
| Buritirana | MA | 5 | 15.430 | 0,32 | 1,0 |
| Lago da Pedra | MA | 16 | 50.266 | 0,32 | 8,6 |
| Miranda do Norte | MA | 9 | 28.381 | 0,32 | 0,0 |
| Pedro do Rosário | MA | 8 | 25.144 | 0,32 | 15,7 |
| Presidente Sarney | MA | 6 | 18.918 | 0,32 | 17,3 |
| São João do Soter | MA | 6 | 18.543 | 0,32 | 0,0 |
| Trizidela do Vale | MA | 7 | 21.998 | 0,32 | 0,0 |
| Uruçuí | PI | 7 | 21.558 | 0,32 | 15,1 |
| Limoeiro do Norte | CE | 19 | 59.540 | 0,32 | 0,0 |
| Areia Branca | RN | 9 | 27.774 | 0,32 | 0,0 |
| São João do Sabugi | RN | 2 | 6.193 | 0,32 | 0,2 |
| Murici | AL | 9 | 28.236 | 0,32 | 1,7 |
| Tanque d'Arca | AL | 2 | 6.162 | 0,32 | 0,0 |
| Amargosa | BA | 12 | 37.241 | 0,32 | 0,1 |
| Anagé | BA | 7 | 22.005 | 0,32 | 30,3 |
| Iraquara | BA | 8 | 25.216 | 0,32 | 1,6 |
| Planaltino | BA | 3 | 9.322 | 0,32 | 4,7 |
| Presidente Jânio Quadros | BA | 4 | 12.338 | 0,32 | 15,8 |
| Seabra | BA | 14 | 44.091 | 0,32 | 1,5 |
| Aimorés | MG | 8 | 25.167 | 0,32 | 0,6 |
| Cachoeira de Pajeú | MG | 3 | 9.412 | 0,32 | 19,0 |
| Buritis | MG | 8 | 24.841 | 0,32 | 6,3 |
| Carbonita | MG | 3 | 9.405 | 0,32 | 4,0 |
| Chácara | MG | 1 | 3.154 | 0,32 | 0,0 |
| Coromandel | MG | 9 | 27.974 | 0,32 | 13,0 |
| Córrego Fundo | MG | 2 | 6.337 | 0,32 | 0,0 |
| Glaucilândia | MG | 1 | 3.150 | 0,32 | 0,3 |
| Ibiá | MG | 8 | 25.199 | 0,32 | 0,5 |
| Jaguaraçu | MG | 1 | 3.133 | 0,32 | 0,0 |
| São Francisco de Sales | MG | 2 | 6.238 | 0,32 | 2,3 |
| Silvianópolis | MG | 2 | 6.238 | 0,32 | 0,0 |
| Verdelândia | MG | 3 | 9.355 | 0,32 | 5,3 |
| São Roque do Canaã | ES | 4 | 12.415 | 0,32 | 0,0 |
| Rio das Flores | RJ | 3 | 9.284 | 0,32 | 0,0 |
| Colômbia | SP | 2 | 6.210 | 0,32 | 0,6 |
| Iguape | SP | 10 | 30.857 | 0,32 | 0,1 |
| Jeriquara | SP | 1 | 3.159 | 0,32 | 0,0 |
| Magda | SP | 1 | 3.119 | 0,32 | 0,0 |
| Mira Estrela | SP | 1 | 3.086 | 0,32 | 0,0 |
| Rubiácea | SP | 1 | 3.128 | 0,32 | 0,0 |
| Rubinéia | SP | 1 | 3.148 | 0,32 | 0,0 |
| Santa Maria da Serra | SP | 2 | 6.173 | 0,32 | 0,0 |
| Silveiras | SP | 2 | 6.302 | 0,32 | 0,0 |
| Farol | PR | 1 | 3.089 | 0,32 | 0,0 |
| Ortigueira | PR | 7 | 22.141 | 0,32 | 1,1 |
| Vidal Ramos | SC | 2 | 6.338 | 0,32 | 0,0 |
| Mormaço | RS | 1 | 3.085 | 0,32 | 0,0 |
| Muitos Capões | RS | 1 | 3.162 | 0,32 | 31,3 |
| Santa Maria do Herval | RS | 2 | 6.331 | 0,32 | 0,0 |
| Jaciara | MT | 9 | 27.776 | 0,32 | 99,5 |
| Mirassol d'Oeste | MT | 9 | 27.739 | 0,32 | 0,0 |
| Santo Afonso | MT | 1 | 3.146 | 0,32 | 23,3 |
| Vale de São Domingos | MT | 1 | 3.127 | 0,32 | 5,0 |
| Amorinópolis | GO | 1 | 3.126 | 0,32 | 0,0 |
| Cachoeira Alta | GO | 4 | 12.484 | 0,32 | 1,5 |
| Guaraí | TO | 8 | 25.923 | 0,31 | 1,3 |
| Deputado Irapuan Pinheiro | CE | 3 | 9.625 | 0,31 | 100,0 |
| Pacujá | CE | 2 | 6.533 | 0,31 | 0,0 |
| Quixeramobim | CE | 25 | 81.082 | 0,31 | 14,8 |
| Água Nova | RN | 1 | 3.252 | 0,31 | 100,0 |
| Rafael Godeiro | RN | 1 | 3.201 | 0,31 | 25,1 |
| São Vicente | RN | 2 | 6.424 | 0,31 | 13,7 |
| Cajazeirinhas | PB | 1 | 3.193 | 0,31 | 100,0 |
| Piancó | PB | 5 | 16.075 | 0,31 | 19,6 |
| Remígio | PB | 6 | 19.621 | 0,31 | 4,5 |
| Flores | PE | 7 | 22.624 | 0,31 | 6,4 |
| Serrita | PE | 6 | 19.165 | 0,31 | 33,3 |
| Barra de Santo Antônio | AL | 5 | 15.932 | 0,31 | 0,0 |
| Nossa Senhora de Lourdes | SE | 2 | 6.483 | 0,31 | 0,0 |
| Alcobaça | BA | 7 | 22.470 | 0,31 | 3,0 |
| Itanhém | BA | 6 | 19.405 | 0,31 | 7,9 |
| Borda da Mata | MG | 6 | 19.412 | 0,31 | 0,0 |
| Conselheiro Pena | MG | 7 | 22.921 | 0,31 | 1,6 |
| Ferros | MG | 3 | 9.820 | 0,31 | 9,6 |
| Lassance | MG | 2 | 6.512 | 0,31 | 6,3 |
| Morro do Pilar | MG | 1 | 3.182 | 0,31 | 7,2 |
| Nacip Raydan | MG | 1 | 3.220 | 0,31 | 2,1 |
| Piracema | MG | 2 | 6.409 | 0,31 | 0,0 |
| Presidente Olegário | MG | 6 | 19.573 | 0,31 | 6,2 |
| Sacramento | MG | 8 | 26.185 | 0,31 | 2,1 |
| São João das Missões | MG | 4 | 13.014 | 0,31 | 20,0 |
| São Sebastião do Anta | MG | 2 | 6.555 | 0,31 | 0,0 |
| Mangaratiba | RJ | 14 | 44.468 | 0,31 | 0,6 |
| Pilar do Sul | SP | 9 | 29.185 | 0,31 | 0,0 |
| Bituruna | PR | 5 | 16.389 | 0,31 | 2,3 |
| Boa Ventura de São Roque | PR | 2 | 6.387 | 0,31 | 1,4 |
| Bom Sucesso do Sul | PR | 1 | 3.264 | 0,31 | 0,0 |
| Capanema | PR | 6 | 19.124 | 0,31 | 0,0 |
| Ibema | PR | 2 | 6.352 | 0,31 | 0,0 |
| Imbaú | PR | 4 | 13.111 | 0,31 | 0,0 |
| Pitangueiras | PR | 1 | 3.224 | 0,31 | 0,0 |
| Porto Barreiro | PR | 1 | 3.237 | 0,31 | 11,3 |
| Ribeirão do Pinhal | PR | 4 | 13.029 | 0,31 | 0,2 |
| Rio Bom | PR | 1 | 3.203 | 0,31 | 0,0 |
| Águas de Chapecó | SC | 2 | 6.486 | 0,31 | 0,0 |
| Entre Rios | SC | 1 | 3.203 | 0,31 | 0,0 |
| Mostardas | RS | 4 | 12.804 | 0,31 | 82,7 |
| Passo do Sobrado | RS | 2 | 6.535 | 0,31 | 0,0 |
| Selvíria | MS | 2 | 6.529 | 0,31 | 8,9 |
| Aripuanã | MT | 7 | 22.354 | 0,31 | 9,0 |
| Barra do Garças | MT | 19 | 61.012 | 0,31 | 6,8 |
| Caçu | GO | 5 | 16.009 | 0,31 | 0,8 |
| Cristalina | GO | 18 | 58.997 | 0,31 | 3,9 |
| Estrela do Norte | GO | 1 | 3.275 | 0,31 | 0,0 |
| Professor Jamil | GO | 1 | 3.223 | 0,31 | 0,1 |
| Bannach | PA | 1 | 3.286 | 0,3 | 46,4 |
| Aguiarnópolis | TO | 2 | 6.733 | 0,3 | 0,0 |
| Palmeiras do Tocantins | TO | 2 | 6.658 | 0,3 | 0,7 |
| Caxias | MA | 49 | 164.880 | 0,3 | 4,3 |
| Urbano Santos | MA | 10 | 33.122 | 0,3 | 4,1 |
| Milton Brandão | PI | 2 | 6.613 | 0,3 | 35,8 |
| Pajeú do Piauí | PI | 1 | 3.389 | 0,3 | 95,3 |
| Assaré | CE | 7 | 23.417 | 0,3 | 22,0 |
| Viçosa do Ceará | CE | 18 | 60.889 | 0,3 | 55,9 |
| Araruna | PB | 6 | 20.312 | 0,3 | 6,2 |
| Juru | PB | 3 | 9.867 | 0,3 | 0,0 |
| Afogados da Ingazeira | PE | 11 | 37.259 | 0,3 | 1,3 |
| Brejo da Madre de Deus | PE | 15 | 50.742 | 0,3 | 3,5 |
| Lagoa do Ouro | PE | 4 | 13.145 | 0,3 | 0,0 |
| Belo Monte | AL | 2 | 6.704 | 0,3 | 0,0 |
| Japoatã | SE | 4 | 13.434 | 0,3 | 0,0 |
| Caldeirão Grande | BA | 4 | 13.327 | 0,3 | 0,0 |
| Malhada | BA | 5 | 16.845 | 0,3 | 0,1 |
| Tremedal | BA | 5 | 16.394 | 0,3 | 6,8 |
| Bias Fortes | MG | 1 | 3.379 | 0,3 | 2,1 |
| Bocaiúva | MG | 15 | 49.979 | 0,3 | 3,6 |
| Careaçu | MG | 2 | 6.757 | 0,3 | 0,0 |
| Crisólita | MG | 2 | 6.704 | 0,3 | 0,0 |
| Goiabeira | MG | 1 | 3.353 | 0,3 | 0,0 |
| Itaguara | MG | 4 | 13.358 | 0,3 | 0,0 |
| Luislândia | MG | 2 | 6.699 | 0,3 | 0,0 |
| São João do Paraíso | MG | 7 | 23.618 | 0,3 | 9,4 |
| Tumiritinga | MG | 2 | 6.732 | 0,3 | 0,0 |
| João Neiva | ES | 5 | 16.668 | 0,3 | 0,0 |
| Bom Jesus do Itabapoana | RJ | 11 | 37.096 | 0,3 | 0,1 |
| Anhembi | SP | 2 | 6.724 | 0,3 | 0,0 |
| Itaóca | SP | 1 | 3.328 | 0,3 | 0,0 |
| Natividade da Serra | SP | 2 | 6.661 | 0,3 | 0,1 |
| Rinópolis | SP | 3 | 9.981 | 0,3 | 0,2 |
| Torrinha | SP | 3 | 10.010 | 0,3 | 0,0 |
| Floresta | PR | 2 | 6.774 | 0,3 | 0,0 |
| Manoel Ribas | PR | 4 | 13.502 | 0,3 | 0,0 |
| Palmital | PR | 4 | 13.172 | 0,3 | 7,4 |
| Sapopema | PR | 2 | 6.736 | 0,3 | 0,0 |
| Tijucas do Sul | PR | 5 | 16.868 | 0,3 | 0,0 |
| Guaraciaba | SC | 3 | 10.090 | 0,3 | 0,0 |
| Monte Carlo | SC | 3 | 9.866 | 0,3 | 0,0 |
| Braga | RS | 1 | 3.353 | 0,3 | 0,0 |
| Maquiné | RS | 2 | 6.714 | 0,3 | 0,0 |
| Nova Bréscia | RS | 1 | 3.330 | 0,3 | 0,0 |
| Caarapó | MS | 9 | 30.174 | 0,3 | 0,1 |
| Brasnorte | MT | 6 | 19.695 | 0,3 | 18,0 |
| Colíder | MT | 10 | 33.438 | 0,3 | 2,4 |
| Nova Olímpia | MT | 6 | 20.301 | 0,3 | 7,4 |
| Cocalzinho de Goiás | GO | 6 | 20.240 | 0,3 | 0,8 |
| Damianópolis | GO | 1 | 3.311 | 0,3 | 0,0 |
| Morrinhos | GO | 14 | 46.136 | 0,3 | 0,1 |
| Santa Tereza de Goiás | GO | 1 | 3.355 | 0,3 | 0,0 |
| Ariquemes | RO | 31 | 107.863 | 0,29 | 5,4 |
| Vale do Paraíso | RO | 2 | 6.825 | 0,29 | 100,0 |
| Jacundá | PA | 17 | 59.155 | 0,29 | 4,5 |
| Pedro Afonso | TO | 4 | 13.578 | 0,29 | 2,1 |
| Bacabeira | MA | 5 | 17.055 | 0,29 | 0,9 |
| Bom Jardim | MA | 12 | 41.630 | 0,29 | 44,0 |
| Boa Hora | PI | 2 | 6.781 | 0,29 | 1,9 |
| Cocal | PI | 8 | 27.787 | 0,29 | 21,3 |
| Morro do Chapéu do Piauí | PI | 2 | 6.796 | 0,29 | 1,4 |
| Jaguaribe | CE | 10 | 34.682 | 0,29 | 100,0 |
| Monsenhor Tabosa | CE | 5 | 17.234 | 0,29 | 100,0 |
| Parambu | CE | 9 | 31.521 | 0,29 | 97,9 |
| Currais Novos | RN | 13 | 44.786 | 0,29 | 0,5 |
| Grossos | RN | 3 | 10.383 | 0,29 | 0,0 |
| Natuba | PB | 3 | 10.454 | 0,29 | 59,7 |
| Cabrobó | PE | 10 | 34.221 | 0,29 | 3,5 |
| Itapetim | PE | 4 | 13.616 | 0,29 | 98,9 |
| Itaquitinga | PE | 5 | 16.955 | 0,29 | 0,0 |
| Traipu | AL | 8 | 27.715 | 0,29 | 0,0 |
| Santa Luzia do Itanhy | SE | 4 | 14.035 | 0,29 | 0,0 |
| Catu | BA | 16 | 54.704 | 0,29 | 0,0 |
| Iaçu | BA | 7 | 24.305 | 0,29 | 5,4 |
| Porto Seguro | BA | 43 | 148.686 | 0,29 | 1,2 |
| Abadia dos Dourados | MG | 2 | 6.989 | 0,29 | 88,7 |
| Bonfim | MG | 2 | 6.868 | 0,29 | 0,0 |
| Comercinho | MG | 2 | 6.929 | 0,29 | 13,3 |
| Conceição das Alagoas | MG | 8 | 27.893 | 0,29 | 0,4 |
| Coronel Xavier Chaves | MG | 1 | 3.434 | 0,29 | 0,0 |
| Frutal | MG | 17 | 59.496 | 0,29 | 0,3 |
| Ibitiúra de Minas | MG | 1 | 3.488 | 0,29 | 0,0 |
| Itamarandiba | MG | 10 | 34.735 | 0,29 | 7,0 |
| Monte Azul | MG | 6 | 20.854 | 0,29 | 0,0 |
| Naque | MG | 2 | 6.996 | 0,29 | 1,7 |
| Periquito | MG | 2 | 6.810 | 0,29 | 0,4 |
| São Miguel do Anta | MG | 2 | 6.938 | 0,29 | 0,0 |
| Barra de São Francisco | ES | 13 | 44.650 | 0,29 | 0,0 |
| Buritama | SP | 5 | 17.144 | 0,29 | 0,0 |
| Itariri | SP | 5 | 17.436 | 0,29 | 0,0 |
| Mombuca | SP | 1 | 3.493 | 0,29 | 0,0 |
| Morungaba | SP | 4 | 13.622 | 0,29 | 0,0 |
| Corbélia | PR | 5 | 17.071 | 0,29 | 0,0 |
| Diamante do Sul | PR | 1 | 3.439 | 0,29 | 0,4 |
| Faxinal | PR | 5 | 17.251 | 0,29 | 0,0 |
| Jacinto Machado | SC | 3 | 10.416 | 0,29 | 0,0 |
| Major Gercino | SC | 1 | 3.442 | 0,29 | 0,2 |
| Sinimbu | RS | 3 | 10.172 | 0,29 | 2,7 |
| Vale Verde | RS | 1 | 3.497 | 0,29 | 0,0 |
| Nioaque | MS | 4 | 13.930 | 0,29 | 15,8 |
| Carlinda | MT | 3 | 10.305 | 0,29 | 6,2 |
| Peixoto de Azevedo | MT | 10 | 34.976 | 0,29 | 17,6 |
| Alexânia | GO | 8 | 27.653 | 0,29 | 0,0 |
| Aragoiânia | GO | 3 | 10.308 | 0,29 | 0,0 |
| Cromínia | GO | 1 | 3.486 | 0,29 | 0,0 |
| Petrolina de Goiás | GO | 3 | 10.281 | 0,29 | 0,0 |
| Piracanjuba | GO | 7 | 24.524 | 0,29 | 1,0 |
| Trombas | GO | 1 | 3.500 | 0,29 | 2,8 |
| Vianópolis | GO | 4 | 13.863 | 0,29 | 0,0 |
| Tarauacá | AC | 12 | 42.567 | 0,28 | 28,8 |
| Muricilândia | TO | 1 | 3.551 | 0,28 | 23,7 |
| Bacabal | MA | 29 | 104.949 | 0,28 | 1,4 |
| Alto Longá | PI | 4 | 14.304 | 0,28 | 9,8 |
| Belém do Piauí | PI | 1 | 3.566 | 0,28 | 23,5 |
| Bom Jesus | PI | 7 | 25.179 | 0,28 | 3,1 |
| Palmeirais | PI | 4 | 14.539 | 0,28 | 3,1 |
| Boa Viagem | CE | 15 | 54.470 | 0,28 | 100,0 |
| Jaguaretama | CE | 5 | 18.162 | 0,28 | 94,3 |
| Mulungu | CE | 3 | 10.823 | 0,28 | 16,5 |
| Quixadá | CE | 25 | 87.728 | 0,28 | 0,0 |
| Emas | PB | 1 | 3.522 | 0,28 | 100,0 |
| Itaporanga | PB | 7 | 24.692 | 0,28 | 13,4 |
| Junco do Seridó | PB | 2 | 7.150 | 0,28 | 19,9 |
| Nova Floresta | PB | 3 | 10.638 | 0,28 | 1,0 |
| Santana dos Garrotes | PB | 2 | 7.031 | 0,28 | 20,5 |
| São José dos Cordeiros | PB | 1 | 3.628 | 0,28 | 100,0 |
| Bezerros | PE | 17 | 60.798 | 0,28 | 0,0 |
| Palmares | PE | 18 | 63.250 | 0,28 | 8,6 |
| Jacuípe | AL | 2 | 7.021 | 0,28 | 99,9 |
| Piaçabuçu | AL | 5 | 17.827 | 0,28 | 0,0 |
| Indiaroba | SE | 5 | 17.957 | 0,28 | 0,0 |
| Érico Cardoso | BA | 3 | 10.610 | 0,28 | 14,2 |
| Camamu | BA | 10 | 35.316 | 0,28 | 5,6 |
| Ibicaraí | BA | 6 | 21.689 | 0,28 | 0,0 |
| Queimadas | BA | 7 | 25.439 | 0,28 | 9,4 |
| Taperoá | BA | 6 | 21.074 | 0,28 | 20,1 |
| Alvorada de Minas | MG | 1 | 3.606 | 0,28 | 8,4 |
| Brazópolis | MG | 4 | 14.459 | 0,28 | 0,0 |
| Cruzeiro da Fortaleza | MG | 1 | 3.626 | 0,28 | 0,0 |
| Guidoval | MG | 2 | 7.078 | 0,28 | 0,0 |
| Nova Módica | MG | 1 | 3.600 | 0,28 | 17,2 |
| São Tomás de Aquino | MG | 2 | 7.021 | 0,28 | 0,1 |
| Varjão de Minas | MG | 2 | 7.036 | 0,28 | 5,1 |
| Várzea da Palma | MG | 11 | 39.493 | 0,28 | 0,5 |
| Vieiras | MG | 1 | 3.608 | 0,28 | 0,0 |
| São José do Calçado | ES | 3 | 10.556 | 0,28 | 0,0 |
| Adolfo | SP | 1 | 3.562 | 0,28 | 0,0 |
| Cunha | SP | 6 | 21.547 | 0,28 | 0,0 |
| General Salgado | SP | 3 | 10.869 | 0,28 | 0,0 |
| São Luís do Paraitinga | SP | 3 | 10.687 | 0,28 | 0,0 |
| Sebastianópolis do Sul | SP | 1 | 3.513 | 0,28 | 0,0 |
| Luiziana | PR | 2 | 7.262 | 0,28 | 0,6 |
| Missal | PR | 3 | 10.702 | 0,28 | 0,0 |
| Pinhão | PR | 9 | 32.391 | 0,28 | 1,0 |
| Salgado Filho | PR | 1 | 3.580 | 0,28 | 0,0 |
| Faxinal dos Guedes | SC | 3 | 10.667 | 0,28 | 0,0 |
| Ilhota | SC | 4 | 14.184 | 0,28 | 0,0 |
| Vargeão | SC | 1 | 3.573 | 0,28 | 0,0 |
| Amaral Ferrador | RS | 2 | 7.031 | 0,28 | 100,0 |
| Áurea | RS | 1 | 3.554 | 0,28 | 0,0 |
| São José dos Ausentes | RS | 1 | 3.527 | 0,28 | 0,1 |
| Itaquiraí | MS | 6 | 21.142 | 0,28 | 0,0 |
| Sidrolândia | MS | 16 | 57.665 | 0,28 | 11,1 |
| Taquarussu | MS | 1 | 3.588 | 0,28 | 0,7 |
| Sorriso | MT | 25 | 90.313 | 0,28 | 100,0 |
| Formosa | GO | 34 | 121.617 | 0,28 | 1,1 |
| Pirenópolis | GO | 7 | 24.908 | 0,28 | 0,2 |
| Rolim de Moura | RO | 15 | 55.058 | 0,27 | 0,6 |
| Chupinguaia | RO | 3 | 11.182 | 0,27 | 100,0 |
| Porto Acre | AC | 5 | 18.504 | 0,27 | 16,7 |
| Parauapebas | PA | 57 | 208.273 | 0,27 | 0,7 |
| Aurora do Tocantins | TO | 1 | 3.757 | 0,27 | 1,8 |
| Cristalândia | TO | 2 | 7.289 | 0,27 | 0,5 |
| Esperantina | TO | 3 | 10.996 | 0,27 | 0,0 |
| Brejo | MA | 10 | 36.397 | 0,27 | 3,9 |
| Itinga do Maranhão | MA | 7 | 26.000 | 0,27 | 17,7 |
| Santa Quitéria do Maranhão | MA | 7 | 25.642 | 0,27 | 22,1 |
| Senador Alexandre Costa | MA | 3 | 11.141 | 0,27 | 0,0 |
| Elesbão Veloso | PI | 4 | 14.602 | 0,27 | 8,2 |
| Ribeiro Gonçalves | PI | 2 | 7.341 | 0,27 | 98,3 |
| União | PI | 12 | 44.485 | 0,27 | 0,1 |
| Canindé | CE | 21 | 76.997 | 0,27 | 27,0 |
| Guaraciaba do Norte | CE | 11 | 40.642 | 0,27 | 23,8 |
| Porteiras | CE | 4 | 14.996 | 0,27 | 24,2 |
| Jucurutu | RN | 5 | 18.295 | 0,27 | 8,2 |
| Belém do Brejo do Cruz | PB | 2 | 7.342 | 0,27 | 15,3 |
| Fagundes | PB | 3 | 11.253 | 0,27 | 7,9 |
| Itatuba | PB | 3 | 10.962 | 0,27 | 0,3 |
| Tavares | PB | 4 | 14.726 | 0,27 | 0,0 |
| Brejinho | PE | 2 | 7.487 | 0,27 | 69,9 |
| Correntes | PE | 5 | 18.207 | 0,27 | 0,2 |
| Itaíba | PE | 7 | 26.349 | 0,27 | 18,4 |
| São José do Belmonte | PE | 9 | 33.959 | 0,27 | 2,9 |
| Serra Talhada | PE | 23 | 86.350 | 0,27 | 1,3 |
| Moreilândia | PE | 3 | 11.270 | 0,27 | 21,8 |
| Xexéu | PE | 4 | 14.725 | 0,27 | 9,5 |
| Ibipitanga | BA | 4 | 14.902 | 0,27 | 8,6 |
| Lafaiete Coutinho | BA | 1 | 3.724 | 0,27 | 16,0 |
| Riachão das Neves | BA | 6 | 22.339 | 0,27 | 3,5 |
| Teolândia | BA | 4 | 14.943 | 0,27 | 7,0 |
| Alpercata | MG | 2 | 7.424 | 0,27 | 0,3 |
| Areado | MG | 4 | 15.070 | 0,27 | 0,0 |
| Campanário | MG | 1 | 3.721 | 0,27 | 3,4 |
| Carmo de Minas | MG | 4 | 14.859 | 0,27 | 0,0 |
| Catas Altas da Noruega | MG | 1 | 3.641 | 0,27 | 0,0 |
| Engenheiro Caldas | MG | 3 | 11.134 | 0,27 | 0,0 |
| Manga | MG | 5 | 18.407 | 0,27 | 1,8 |
| Matias Cardoso | MG | 3 | 11.157 | 0,27 | 2,9 |
| Matutina | MG | 1 | 3.749 | 0,27 | 0,1 |
| Unaí | MG | 23 | 84.378 | 0,27 | 2,6 |
| Laranja da Terra | ES | 3 | 10.947 | 0,27 | 0,0 |
| Laje do Muriaé | RJ | 2 | 7.355 | 0,27 | 0,1 |
| Piraí | RJ | 8 | 29.277 | 0,27 | 0,0 |
| Álvares Florence | SP | 1 | 3.679 | 0,27 | 0,0 |
| Divinolândia | SP | 3 | 11.146 | 0,27 | 0,0 |
| Nuporanga | SP | 2 | 7.432 | 0,27 | 0,1 |
| Paulicéia | SP | 2 | 7.366 | 0,27 | 0,0 |
| Sarutaiá | SP | 1 | 3.638 | 0,27 | 0,0 |
| Antônio Olinto | PR | 2 | 7.434 | 0,27 | 9,7 |
| Cruz Machado | PR | 5 | 18.708 | 0,27 | 5,6 |
| Juranda | PR | 2 | 7.340 | 0,27 | 0,0 |
| Paulo Frontin | PR | 2 | 7.354 | 0,27 | 0,0 |
| Rio do Oeste | SC | 2 | 7.489 | 0,27 | 0,5 |
| Ametista do Sul | RS | 2 | 7.409 | 0,27 | 0,0 |
| Barros Cassal | RS | 3 | 11.199 | 0,27 | 10,9 |
| Coronel Bicaco | RS | 2 | 7.325 | 0,27 | 0,1 |
| Nova Roma do Sul | RS | 1 | 3.689 | 0,27 | 0,1 |
| São Francisco de Assis | RS | 5 | 18.335 | 0,27 | 23,7 |
| Triunfo | RS | 8 | 29.538 | 0,27 | 0,0 |
| Bonito | MS | 6 | 21.976 | 0,27 | 5,2 |
| Anicuns | GO | 6 | 21.850 | 0,27 | 0,0 |
| Brazabrantes | GO | 1 | 3.703 | 0,27 | 0,0 |
| Heitoraí | GO | 1 | 3.724 | 0,27 | 0,0 |
| Joviânia | GO | 2 | 7.387 | 0,27 | 0,0 |
| Jussara | GO | 5 | 18.478 | 0,27 | 2,6 |
| Ouro Verde de Goiás | GO | 1 | 3.759 | 0,27 | 0,0 |
| Uruaçu | GO | 11 | 40.532 | 0,27 | 1,1 |
| Cacoal | RO | 22 | 85.359 | 0,26 | 0,5 |
| Governador Jorge Teixeira | RO | 2 | 7.767 | 0,26 | 99,7 |
| Augusto Corrêa | PA | 12 | 45.998 | 0,26 | 17,3 |
| Cajari | MA | 5 | 19.379 | 0,26 | 51,0 |
| Fortuna | MA | 4 | 15.552 | 0,26 | 0,6 |
| Governador Luiz Rocha | MA | 2 | 7.807 | 0,26 | 0,7 |
| Lajeado Novo | MA | 2 | 7.550 | 0,26 | 10,6 |
| Parnarama | MA | 9 | 34.907 | 0,26 | 17,0 |
| Monsenhor Hipólito | PI | 2 | 7.749 | 0,26 | 5,7 |
| Santo Inácio do Piauí | PI | 1 | 3.798 | 0,26 | 9,5 |
| Nova Olinda | CE | 4 | 15.565 | 0,26 | 0,7 |
| Açu | RN | 15 | 58.017 | 0,26 | 43,3 |
| Ipanguaçu | RN | 4 | 15.491 | 0,26 | 100,0 |
| Itajá | RN | 2 | 7.548 | 0,26 | 99,2 |
| Jundiá | RN | 1 | 3.898 | 0,26 | 0,0 |
| Lagoa Nova | RN | 4 | 15.614 | 0,26 | 7,3 |
| Vista Serrana | PB | 1 | 3.798 | 0,26 | 0,0 |
| Salgadinho | PB | 1 | 3.885 | 0,26 | 96,4 |
| Ibirajuba | PE | 2 | 7.762 | 0,26 | 2,0 |
| Mirandiba | PE | 4 | 15.390 | 0,26 | 16,1 |
| Paulo Jacinto | AL | 2 | 7.564 | 0,26 | 0,0 |
| Ituaçu | BA | 5 | 18.962 | 0,26 | 6,1 |
| Macajuba | BA | 3 | 11.348 | 0,26 | 21,0 |
| Antônio Carlos | MG | 3 | 11.445 | 0,26 | 0,0 |
| Caetanópolis | MG | 3 | 11.624 | 0,26 | 0,0 |
| Campo Azul | MG | 1 | 3.817 | 0,26 | 8,1 |
| Carmo da Mata | MG | 3 | 11.476 | 0,26 | 0,0 |
| Coimbra | MG | 2 | 7.556 | 0,26 | 0,0 |
| Conceição dos Ouros | MG | 3 | 11.638 | 0,26 | 0,0 |
| Divinolândia de Minas | MG | 2 | 7.571 | 0,26 | 1,1 |
| Guarará | MG | 1 | 3.796 | 0,26 | 0,0 |
| Itutinga | MG | 1 | 3.788 | 0,26 | 0,3 |
| Santana de Cataguases | MG | 1 | 3.872 | 0,26 | 0,0 |
| Santana de Pirapama | MG | 2 | 7.642 | 0,26 | 28,9 |
| Santo Antônio do Grama | MG | 1 | 3.911 | 0,26 | 0,0 |
| São João do Manhuaçu | MG | 3 | 11.559 | 0,26 | 0,0 |
| Ecoporanga | ES | 6 | 22.923 | 0,26 | 0,2 |
| Guaçuí | ES | 8 | 30.867 | 0,26 | 0,0 |
| Jaguaré | ES | 8 | 30.477 | 0,26 | 0,0 |
| Muqui | ES | 4 | 15.449 | 0,26 | 0,0 |
| Natividade | RJ | 4 | 15.317 | 0,26 | 0,0 |
| Itobi | SP | 2 | 7.841 | 0,26 | 0,0 |
| Nhandeara | SP | 3 | 11.478 | 0,26 | 0,0 |
| Redenção da Serra | SP | 1 | 3.851 | 0,26 | 0,0 |
| Boa Vista da Aparecida | PR | 2 | 7.591 | 0,26 | 0,0 |
| Campina do Simão | PR | 1 | 3.887 | 0,26 | 0,6 |
| Guapirama | PR | 1 | 3.802 | 0,26 | 0,0 |
| Ipiranga | PR | 4 | 15.172 | 0,26 | 0,0 |
| Matinhos | PR | 9 | 34.720 | 0,26 | 0,0 |
| Pitanga | PR | 8 | 30.310 | 0,26 | 0,9 |
| Santa Helena | PR | 7 | 26.490 | 0,26 | 0,0 |
| Santa Mariana | PR | 3 | 11.724 | 0,26 | 0,0 |
| Cotiporã | RS | 1 | 3.853 | 0,26 | 4,7 |
| Mariana Pimentel | RS | 1 | 3.885 | 0,26 | 21,3 |
| Paim Filho | RS | 1 | 3.828 | 0,26 | 8,5 |
| Pontão | RS | 1 | 3.904 | 0,26 | 0,0 |
| Putinga | RS | 1 | 3.919 | 0,26 | 1,0 |
| Quinze de Novembro | RS | 1 | 3.796 | 0,26 | 31,3 |
| São Miguel das Missões | RS | 2 | 7.673 | 0,26 | 4,5 |
| São Pedro da Serra | RS | 1 | 3.801 | 0,26 | 0,0 |
| Dois Irmãos do Buriti | MS | 3 | 11.385 | 0,26 | 17,0 |
| Fátima do Sul | MS | 5 | 19.189 | 0,26 | 0,0 |
| Paranaíba | MS | 11 | 42.148 | 0,26 | 0,9 |
| Pedro Gomes | MS | 2 | 7.674 | 0,26 | 8,5 |
| Sonora | MS | 5 | 19.274 | 0,26 | 3,2 |
| Nova Nazaré | MT | 1 | 3.849 | 0,26 | 10,2 |
| Nova Mutum | MT | 12 | 45.378 | 0,26 | 18,8 |
| Primavera do Leste | MT | 16 | 62.019 | 0,26 | 100,0 |
| Amaralina | GO | 1 | 3.812 | 0,26 | 38,6 |
| Caldazinha | GO | 1 | 3.804 | 0,26 | 0,0 |
| Indiara | GO | 4 | 15.611 | 0,26 | 0,3 |
| Jataí | GO | 26 | 100.882 | 0,26 | 1,7 |
| Leopoldo de Bulhões | GO | 2 | 7.647 | 0,26 | 0,0 |
| Quirinópolis | GO | 13 | 50.065 | 0,26 | 2,7 |
| Rio Verde | GO | 62 | 235.647 | 0,26 | 1,0 |
| Varjão | GO | 1 | 3.827 | 0,26 | 0,4 |
| São Luiz | RR | 2 | 7.986 | 0,25 | 0,6 |
| Bragança | PA | 32 | 127.686 | 0,25 | 0,8 |
| Salinópolis | PA | 10 | 40.675 | 0,25 | 0,0 |
| Carutapera | MA | 6 | 23.807 | 0,25 | 100,0 |
| Coivaras | PI | 1 | 4.020 | 0,25 | 1,1 |
| Lagoa do Piauí | PI | 1 | 4.064 | 0,25 | 1,7 |
| São João do Arraial | PI | 2 | 7.989 | 0,25 | 0,1 |
| Major Sales | RN | 1 | 4.020 | 0,25 | 100,0 |
| Tangará | RN | 4 | 15.727 | 0,25 | 3,5 |
| Bonito de Santa Fé | PB | 3 | 11.917 | 0,25 | 100,0 |
| Juarez Távora | PB | 2 | 7.936 | 0,25 | 0,0 |
| Belém de Maria | PE | 3 | 12.073 | 0,25 | 0,0 |
| Joaquim Gomes | AL | 6 | 23.993 | 0,25 | 13,0 |
| Canhoba | SE | 1 | 4.008 | 0,25 | 0,0 |
| Santo Amaro das Brotas | SE | 3 | 12.102 | 0,25 | 0,0 |
| Antônio Gonçalves | BA | 3 | 11.798 | 0,25 | 0,4 |
| Itamari | BA | 2 | 8.035 | 0,25 | 0,0 |
| Pindobaçu | BA | 5 | 20.150 | 0,25 | 0,0 |
| Wanderley | BA | 3 | 12.238 | 0,25 | 18,2 |
| Buritizeiro | MG | 7 | 28.056 | 0,25 | 6,7 |
| Cajuri | MG | 1 | 3.987 | 0,25 | 0,0 |
| Capinópolis | MG | 4 | 16.173 | 0,25 | 0,0 |
| Carmo da Cachoeira | MG | 3 | 12.170 | 0,25 | 0,0 |
| Conceição da Barra de Minas | MG | 1 | 3.954 | 0,25 | 0,3 |
| Iguatama | MG | 2 | 7.947 | 0,25 | 0,0 |
| Januária | MG | 17 | 67.742 | 0,25 | 9,9 |
| João Pinheiro | MG | 12 | 47.452 | 0,25 | 5,0 |
| Maravilhas | MG | 2 | 7.976 | 0,25 | 0,0 |
| Patrocínio | MG | 23 | 90.757 | 0,25 | 3,0 |
| Pedra Azul | MG | 6 | 24.324 | 0,25 | 3,8 |
| Planura | MG | 3 | 12.133 | 0,25 | 0,0 |
| Pompéu | MG | 8 | 31.812 | 0,25 | 0,6 |
| São Francisco | MG | 14 | 56.323 | 0,25 | 6,6 |
| Taquaraçu de Minas | MG | 1 | 4.077 | 0,25 | 0,0 |
| Atilio Vivacqua | ES | 3 | 11.936 | 0,25 | 0,0 |
| Jerônimo Monteiro | ES | 3 | 12.192 | 0,25 | 0,0 |
| Cachoeiras de Macacu | RJ | 15 | 58.937 | 0,25 | 0,0 |
| Monte Alegre do Sul | SP | 2 | 8.038 | 0,25 | 0,0 |
| Paranapuã | SP | 1 | 4.078 | 0,25 | 0,0 |
| Registro | SP | 14 | 56.322 | 0,25 | 0,0 |
| Suzanápolis | SP | 1 | 3.963 | 0,25 | 0,0 |
| Icaraíma | PR | 2 | 7.904 | 0,25 | 0,0 |
| Leópolis | PR | 1 | 3.954 | 0,25 | 0,7 |
| Nova Fátima | PR | 2 | 8.153 | 0,25 | 0,4 |
| Porto Vitória | PR | 1 | 4.065 | 0,25 | 0,0 |
| Querência do Norte | PR | 3 | 12.206 | 0,25 | 0,0 |
| Rio Branco do Sul | PR | 8 | 32.397 | 0,25 | 0,1 |
| Tomazina | PR | 2 | 7.918 | 0,25 | 0,0 |
| Jaborá | SC | 1 | 3.936 | 0,25 | 0,0 |
| Major Vieira | SC | 2 | 8.103 | 0,25 | 0,1 |
| Witmarsum | SC | 1 | 3.965 | 0,25 | 15,4 |
| Xavantina | SC | 1 | 3.933 | 0,25 | 0,0 |
| Getúlio Vargas | RS | 4 | 16.212 | 0,25 | 0,0 |
| Ibirapuitã | RS | 1 | 4.013 | 0,25 | 0,0 |
| Santa Bárbara do Sul | RS | 2 | 7.994 | 0,25 | 0,3 |
| Tuparendi | RS | 2 | 7.893 | 0,25 | 0,0 |
| Juruena | MT | 4 | 15.865 | 0,25 | 6,5 |
| Rubiataba | GO | 5 | 19.882 | 0,25 | 0,0 |
| Porto Velho | RO | 129 | 529.544 | 0,24 | 7,5 |
| Anori | AM | 5 | 21.010 | 0,24 | 19,4 |
| Carrasco Bonito | TO | 1 | 4.095 | 0,24 | 0,0 |
| Conceição do Tocantins | TO | 1 | 4.105 | 0,24 | 4,6 |
| Colméia | TO | 2 | 8.205 | 0,24 | 1,8 |
| Água Doce do Maranhão | MA | 3 | 12.571 | 0,24 | 0,0 |
| Feira Nova do Maranhão | MA | 2 | 8.504 | 0,24 | 15,9 |
| Milagres do Maranhão | MA | 2 | 8.464 | 0,24 | 20,0 |
| Pacoti | CE | 3 | 12.261 | 0,24 | 0,0 |
| Salitre | CE | 4 | 16.554 | 0,24 | 58,3 |
| Maxaranguape | RN | 3 | 12.371 | 0,24 | 0,0 |
| Paraná | RN | 1 | 4.254 | 0,24 | 100,0 |
| Patu | RN | 3 | 12.755 | 0,24 | 5,8 |
| Venha-Ver | RN | 1 | 4.177 | 0,24 | 100,0 |
| Caraúbas | PB | 1 | 4.162 | 0,24 | 100,0 |
| Mataraca | PB | 2 | 8.434 | 0,24 | 0,7 |
| Sumé | PB | 4 | 16.966 | 0,24 | 99,9 |
| Ibimirim | PE | 7 | 29.235 | 0,24 | 12,9 |
| Ouricuri | PE | 17 | 69.459 | 0,24 | 12,7 |
| Pesqueira | PE | 16 | 67.395 | 0,24 | 0,4 |
| São Caitano | PE | 9 | 37.245 | 0,24 | 0,0 |
| Vertentes | PE | 5 | 20.731 | 0,24 | 1,7 |
| Novo Lino | AL | 3 | 12.690 | 0,24 | 30,4 |
| Brejo Grande | SE | 2 | 8.309 | 0,24 | 0,0 |
| Itiruçu | BA | 3 | 12.576 | 0,24 | 3,9 |
| Jacobina | BA | 19 | 80.518 | 0,24 | 0,0 |
| Malhada de Pedras | BA | 2 | 8.393 | 0,24 | 10,3 |
| Remanso | BA | 10 | 41.008 | 0,24 | 12,2 |
| Campanha | MG | 4 | 16.665 | 0,24 | 0,0 |
| Carmo do Rio Claro | MG | 5 | 21.225 | 0,24 | 0,6 |
| Ilicínea | MG | 3 | 12.375 | 0,24 | 0,0 |
| Ipiaçu | MG | 1 | 4.221 | 0,24 | 0,0 |
| Itaobim | MG | 5 | 21.062 | 0,24 | 6,9 |
| Lagoa dos Patos | MG | 1 | 4.102 | 0,24 | 2,1 |
| Martins Soares | MG | 2 | 8.417 | 0,24 | 0,0 |
| Mato Verde | MG | 3 | 12.459 | 0,24 | 0,1 |
| Medina | MG | 5 | 20.820 | 0,24 | 13,3 |
| Montezuma | MG | 2 | 8.249 | 0,24 | 2,5 |
| Pavão | MG | 2 | 8.450 | 0,24 | 5,6 |
| São João da Ponte | MG | 6 | 25.165 | 0,24 | 1,2 |
| Serro | MG | 5 | 20.966 | 0,24 | 0,8 |
| Tupaciguara | MG | 6 | 25.327 | 0,24 | 0,4 |
| Altair | SP | 1 | 4.160 | 0,24 | 0,0 |
| Cardoso | SP | 3 | 12.326 | 0,24 | 0,0 |
| Nova Luzitânia | SP | 1 | 4.101 | 0,24 | 0,0 |
| Pedregulho | SP | 4 | 16.744 | 0,24 | 0,0 |
| Saltinho | SP | 2 | 8.286 | 0,24 | 0,0 |
| Serra Negra | SP | 7 | 29.229 | 0,24 | 0,0 |
| Kaloré | PR | 1 | 4.100 | 0,24 | 0,0 |
| Nova Santa Bárbara | PR | 1 | 4.249 | 0,24 | 0,0 |
| Teixeira Soares | PR | 3 | 12.367 | 0,24 | 0,0 |
| Modelo | SC | 1 | 4.209 | 0,24 | 0,0 |
| Passos Maia | SC | 1 | 4.147 | 0,24 | 0,1 |
| Caraá | RS | 2 | 8.270 | 0,24 | 0,0 |
| General Câmara | RS | 2 | 8.385 | 0,24 | 0,0 |
| Novo Cabrais | RS | 1 | 4.196 | 0,24 | 0,0 |
| Anastácio | MS | 6 | 25.135 | 0,24 | 3,3 |
| Jangada | MT | 2 | 8.409 | 0,24 | 3,7 |
| Mineiros | GO | 16 | 66.801 | 0,24 | 1,2 |
| Jaru | RO | 12 | 51.775 | 0,23 | 99,9 |
| Ji-Paraná | RO | 30 | 128.969 | 0,23 | 8,3 |
| Alto Paraíso | RO | 5 | 21.428 | 0,23 | 29,4 |
| Tucumã | PA | 9 | 39.602 | 0,23 | 4,8 |
| Bequimão | MA | 5 | 21.280 | 0,23 | 1,0 |
| Cantanhede | MA | 5 | 21.995 | 0,23 | 0,9 |
| Pedreiras | MA | 9 | 39.229 | 0,23 | 0,0 |
| Curralinhos | PI | 1 | 4.443 | 0,23 | 0,0 |
| Aiuaba | CE | 4 | 17.399 | 0,23 | 100,0 |
| Mombaça | CE | 10 | 43.797 | 0,23 | 100,0 |
| São Benedito | CE | 11 | 47.903 | 0,23 | 1,0 |
| Olho-d'Água do Borges | RN | 1 | 4.258 | 0,23 | 19,4 |
| São Paulo do Potengi | RN | 4 | 17.579 | 0,23 | 0,0 |
| Cacimba de Dentro | PB | 4 | 17.187 | 0,23 | 4,0 |
| Poço de José de Moura | PB | 1 | 4.307 | 0,23 | 100,0 |
| Sanharó | PE | 6 | 26.462 | 0,23 | 1,0 |
| Anadia | AL | 4 | 17.545 | 0,23 | 0,0 |
| Flexeiras | AL | 3 | 12.790 | 0,23 | 1,0 |
| Maribondo | AL | 3 | 13.264 | 0,23 | 0,0 |
| São Miguel dos Campos | AL | 14 | 61.251 | 0,23 | 0,0 |
| Canindé de São Francisco | SE | 7 | 29.900 | 0,23 | 0,7 |
| Ilha das Flores | SE | 2 | 8.520 | 0,23 | 0,0 |
| Abaíra | BA | 2 | 8.739 | 0,23 | 0,0 |
| Cordeiros | BA | 2 | 8.614 | 0,23 | 1,1 |
| Igrapiúna | BA | 3 | 13.226 | 0,23 | 24,2 |
| Morro do Chapéu | BA | 8 | 35.413 | 0,23 | 4,3 |
| Piatã | BA | 4 | 17.123 | 0,23 | 1,8 |
| Pilão Arcado | BA | 8 | 35.048 | 0,23 | 29,9 |
| Planalto | BA | 6 | 26.265 | 0,23 | 16,0 |
| Bom Sucesso | MG | 4 | 17.603 | 0,23 | 0,0 |
| Dores do Turvo | MG | 1 | 4.259 | 0,23 | 0,0 |
| Funilândia | MG | 1 | 4.349 | 0,23 | 0,0 |
| Jaíba | MG | 9 | 38.909 | 0,23 | 2,9 |
| Mar de Espanha | MG | 3 | 12.814 | 0,23 | 0,0 |
| Mata Verde | MG | 2 | 8.586 | 0,23 | 0,1 |
| Pequi | MG | 1 | 4.406 | 0,23 | 0,0 |
| Piranga | MG | 4 | 17.626 | 0,23 | 0,0 |
| Pratápolis | MG | 2 | 8.603 | 0,23 | 0,0 |
| Santana do Riacho | MG | 1 | 4.295 | 0,23 | 29,6 |
| Virgínia | MG | 2 | 8.674 | 0,23 | 0,0 |
| Baixo Guandu | ES | 7 | 30.998 | 0,23 | 0,0 |
| Capão Bonito | SP | 11 | 47.138 | 0,23 | 0,0 |
| Guararema | SP | 7 | 29.798 | 0,23 | 0,0 |
| Igarapava | SP | 7 | 30.432 | 0,23 | 0,0 |
| Itaberá | SP | 4 | 17.556 | 0,23 | 0,0 |
| Mongaguá | SP | 13 | 56.702 | 0,23 | 0,0 |
| Nova Granada | SP | 5 | 21.500 | 0,23 | 0,0 |
| Onda Verde | SP | 1 | 4.381 | 0,23 | 0,0 |
| Santa Lúcia | SP | 2 | 8.817 | 0,23 | 0,0 |
| Tanabi | SP | 6 | 25.967 | 0,23 | 0,0 |
| Guamiranga | PR | 2 | 8.739 | 0,23 | 0,0 |
| Planaltina do Paraná | PR | 1 | 4.263 | 0,23 | 0,0 |
| Balneário Arroio do Silva | SC | 3 | 13.071 | 0,23 | 0,0 |
| Campo Erê | SC | 2 | 8.526 | 0,23 | 0,0 |
| Maravilha | SC | 6 | 25.762 | 0,23 | 0,0 |
| Pinhal Grande | RS | 1 | 4.350 | 0,23 | 27,7 |
| Cassilândia | MS | 5 | 21.939 | 0,23 | 3,2 |
| Terenos | MS | 5 | 21.806 | 0,23 | 24,3 |
| Sinop | MT | 33 | 142.996 | 0,23 | 100,0 |
| Matrinchã | GO | 1 | 4.351 | 0,23 | 3,2 |
| Mossâmedes | GO | 1 | 4.290 | 0,23 | 0,0 |
| Santa Rita do Araguaia | GO | 2 | 8.756 | 0,23 | 4,5 |
| São Miguel do Guaporé | RO | 5 | 23.005 | 0,22 | 40,5 |
| Rio Branco | AC | 91 | 407.319 | 0,22 | 0,9 |
| Água Azul do Norte | PA | 6 | 27.430 | 0,22 | 25,6 |
| Augustinópolis | TO | 4 | 18.412 | 0,22 | 0,0 |
| Sítio Novo do Tocantins | TO | 2 | 9.029 | 0,22 | 0,0 |
| Satubinha | MA | 3 | 13.914 | 0,22 | 9,6 |
| Vila Nova dos Martírios | MA | 3 | 13.392 | 0,22 | 1,8 |
| Bocaina | PI | 1 | 4.500 | 0,22 | 0,1 |
| Flores do Piauí | PI | 1 | 4.463 | 0,22 | 100,0 |
| Santana do Piauí | PI | 1 | 4.634 | 0,22 | 0,0 |
| São Lourenço do Piauí | PI | 1 | 4.573 | 0,22 | 2,4 |
| Lavras da Mangabeira | CE | 7 | 31.508 | 0,22 | 23,0 |
| Tianguá | CE | 17 | 75.946 | 0,22 | 2,6 |
| São José do Seridó | RN | 1 | 4.634 | 0,22 | 0,6 |
| Baía da Traição | PB | 2 | 8.993 | 0,22 | 89,3 |
| Altinho | PE | 5 | 22.972 | 0,22 | 0,0 |
| Custódia | PE | 8 | 37.111 | 0,22 | 21,5 |
| Pedra | PE | 5 | 22.617 | 0,22 | 4,5 |
| São João | PE | 5 | 22.793 | 0,22 | 9,4 |
| Messias | AL | 4 | 17.856 | 0,22 | 0,0 |
| Satuba | AL | 3 | 13.828 | 0,22 | 0,0 |
| Conceição da Feira | BA | 5 | 22.581 | 0,22 | 0,0 |
| Cotegipe | BA | 3 | 13.782 | 0,22 | 6,3 |
| Entre Rios | BA | 9 | 41.780 | 0,22 | 0,1 |
| Fátima | BA | 4 | 17.890 | 0,22 | 0,0 |
| Gandu | BA | 7 | 32.403 | 0,22 | 0,0 |
| Maragogipe | BA | 10 | 44.677 | 0,22 | 0,0 |
| Mascote | BA | 3 | 13.822 | 0,22 | 2,4 |
| Mirangaba | BA | 4 | 18.338 | 0,22 | 31,4 |
| Rio Real | BA | 9 | 40.732 | 0,22 | 0,4 |
| Araújos | MG | 2 | 9.273 | 0,22 | 0,0 |
| Brumadinho | MG | 9 | 40.103 | 0,22 | 0,0 |
| Caratinga | MG | 20 | 92.062 | 0,22 | 0,2 |
| Fronteira dos Vales | MG | 1 | 4.581 | 0,22 | 0,0 |
| Itambacuri | MG | 5 | 23.211 | 0,22 | 3,5 |
| Rio Casca | MG | 3 | 13.564 | 0,22 | 0,0 |
| Rio Pomba | MG | 4 | 17.910 | 0,22 | 0,1 |
| Três Marias | MG | 7 | 32.356 | 0,22 | 1,7 |
| Vassouras | RJ | 8 | 36.896 | 0,22 | 0,0 |
| Bálsamo | SP | 2 | 9.068 | 0,22 | 0,0 |
| Buritizal | SP | 1 | 4.481 | 0,22 | 0,0 |
| Queluz | SP | 3 | 13.420 | 0,22 | 0,2 |
| Ribeirão do Sul | SP | 1 | 4.541 | 0,22 | 0,0 |
| Contenda | PR | 4 | 18.584 | 0,22 | 0,0 |
| Cruzeiro do Sul | PR | 1 | 4.469 | 0,22 | 0,0 |
| Entre Rios do Oeste | PR | 1 | 4.539 | 0,22 | 0,0 |
| Mallet | PR | 3 | 13.630 | 0,22 | 0,1 |
| Mandirituba | PR | 6 | 26.869 | 0,22 | 0,0 |
| Matelândia | PR | 4 | 17.943 | 0,22 | 0,5 |
| Planalto | PR | 3 | 13.479 | 0,22 | 0,0 |
| Ramilândia | PR | 1 | 4.451 | 0,22 | 0,0 |
| Cordilheira Alta | SC | 1 | 4.453 | 0,22 | 0,0 |
| Garopaba | SC | 5 | 23.078 | 0,22 | 0,0 |
| Rio Fortuna | SC | 1 | 4.611 | 0,22 | 0,0 |
| Campo Novo | RS | 1 | 4.484 | 0,22 | 0,1 |
| Chapada | RS | 2 | 9.269 | 0,22 | 0,0 |
| Rondonópolis | MT | 51 | 232.491 | 0,22 | 6,1 |
| Tangará da Serra | MT | 23 | 103.750 | 0,22 | 31,0 |
| Tapurah | MT | 3 | 13.705 | 0,22 | 99,8 |
| Acreúna | GO | 5 | 22.366 | 0,22 | 0,0 |
| Itajá | GO | 1 | 4.539 | 0,22 | 8,1 |
| Montividiu | GO | 3 | 13.396 | 0,22 | 0,7 |
| Nazário | GO | 2 | 9.142 | 0,22 | 0,0 |
| Niquelândia | GO | 10 | 46.388 | 0,22 | 3,8 |
| Ministro Andreazza | RO | 2 | 9.660 | 0,21 | 15,3 |
| Pau D'Arco | TO | 1 | 4.849 | 0,21 | 19,9 |
| Morros | MA | 4 | 19.433 | 0,21 | 20,5 |
| Passagem Franca | MA | 4 | 19.019 | 0,21 | 97,5 |
| Pastos Bons | MA | 4 | 19.472 | 0,21 | 97,5 |
| Pindaré-Mirim | MA | 7 | 32.941 | 0,21 | 5,6 |
| Porto Franco | MA | 5 | 23.885 | 0,21 | 3,4 |
| São Benedito do Rio Preto | MA | 4 | 18.663 | 0,21 | 2,1 |
| Viana | MA | 11 | 52.441 | 0,21 | 9,0 |
| Francisco Santos | PI | 2 | 9.319 | 0,21 | 1,0 |
| Jatobá do Piauí | PI | 1 | 4.865 | 0,21 | 11,1 |
| Frecheirinha | CE | 3 | 14.072 | 0,21 | 0,1 |
| Ouro Branco | RN | 1 | 4.812 | 0,21 | 10,8 |
| Pureza | RN | 2 | 9.621 | 0,21 | 0,0 |
| Touros | RN | 7 | 33.287 | 0,21 | 0,7 |
| Congo | PB | 1 | 4.786 | 0,21 | 100,0 |
| Bodocó | PE | 8 | 38.146 | 0,21 | 12,1 |
| Dormentes | PE | 4 | 18.908 | 0,21 | 48,2 |
| Iati | PE | 4 | 19.197 | 0,21 | 15,0 |
| Passira | PE | 6 | 28.933 | 0,21 | 0,0 |
| Tabira | PE | 6 | 28.534 | 0,21 | 5,1 |
| Verdejante | PE | 2 | 9.534 | 0,21 | 0,0 |
| Atalaia | AL | 10 | 47.185 | 0,21 | 0,0 |
| Campo Grande | AL | 2 | 9.558 | 0,21 | 0,0 |
| Japaratuba | SE | 4 | 18.743 | 0,21 | 0,0 |
| Neópolis | SE | 4 | 18.719 | 0,21 | 0,0 |
| Pedrinhas | SE | 2 | 9.602 | 0,21 | 0,0 |
| Andorinha | BA | 3 | 14.595 | 0,21 | 7,5 |
| Angical | BA | 3 | 13.977 | 0,21 | 0,0 |
| Barra | BA | 11 | 53.578 | 0,21 | 18,8 |
| Brejões | BA | 3 | 14.295 | 0,21 | 0,0 |
| Candiba | BA | 3 | 14.319 | 0,21 | 0,0 |
| Coribe | BA | 3 | 14.194 | 0,21 | 3,3 |
| Irará | BA | 6 | 29.034 | 0,21 | 0,0 |
| Itacaré | BA | 6 | 28.296 | 0,21 | 5,4 |
| Paripiranga | BA | 6 | 28.989 | 0,21 | 0,0 |
| Rodelas | BA | 2 | 9.331 | 0,21 | 3,5 |
| Astolfo Dutra | MG | 3 | 14.179 | 0,21 | 0,1 |
| Augusto de Lima | MG | 1 | 4.869 | 0,21 | 22,8 |
| Capela Nova | MG | 1 | 4.653 | 0,21 | 0,0 |
| Elói Mendes | MG | 6 | 28.076 | 0,21 | 0,0 |
| Felício dos Santos | MG | 1 | 4.753 | 0,21 | 16,1 |
| Lontra | MG | 2 | 9.661 | 0,21 | 0,1 |
| Oratórios | MG | 1 | 4.655 | 0,21 | 0,0 |
| Paula Cândido | MG | 2 | 9.571 | 0,21 | 0,0 |
| Santa Cruz do Escalvado | MG | 1 | 4.758 | 0,21 | 0,0 |
| Santa Juliana | MG | 3 | 14.003 | 0,21 | 0,3 |
| Serranópolis de Minas | MG | 1 | 4.781 | 0,21 | 20,5 |
| Quatis | RJ | 3 | 14.302 | 0,21 | 0,0 |
| Cachoeira Paulista | SP | 7 | 33.327 | 0,21 | 0,0 |
| Coronel Macedo | SP | 1 | 4.681 | 0,21 | 0,0 |
| ES do Turvo | SP | 1 | 4.829 | 0,21 | 0,1 |
| Euclides da Cunha Paulista | SP | 2 | 9.371 | 0,21 | 0,4 |
| Júlio Mesquita | SP | 1 | 4.776 | 0,21 | 0,0 |
| Nova Campina | SP | 2 | 9.755 | 0,21 | 0,0 |
| Pirapora do Bom Jesus | SP | 4 | 18.895 | 0,21 | 0,0 |
| Santa Isabel | SP | 12 | 57.386 | 0,21 | 0,0 |
| Santo Antônio de Posse | SP | 5 | 23.310 | 0,21 | 0,0 |
| Ubirajara | SP | 1 | 4.780 | 0,21 | 0,0 |
| Chopinzinho | PR | 4 | 19.254 | 0,21 | 0,3 |
| Lobato | PR | 1 | 4.787 | 0,21 | 0,0 |
| Marumbi | PR | 1 | 4.679 | 0,21 | 0,0 |
| Porto Amazonas | PR | 1 | 4.848 | 0,21 | 0,0 |
| Wenceslau Braz | PR | 4 | 19.414 | 0,21 | 0,0 |
| Araquari | SC | 8 | 38.129 | 0,21 | 0,0 |
| Guatambú | SC | 1 | 4.704 | 0,21 | 0,0 |
| Nova Trento | SC | 3 | 14.549 | 0,21 | 0,5 |
| Papanduva | SC | 4 | 19.320 | 0,21 | 0,1 |
| Pomerode | SC | 7 | 33.447 | 0,21 | 0,0 |
| Caiçara | RS | 1 | 4.743 | 0,21 | 0,0 |
| Capivari do Sul | RS | 1 | 4.660 | 0,21 | 0,0 |
| Cerro Branco | RS | 1 | 4.691 | 0,21 | 0,2 |
| Ciríaco | RS | 1 | 4.747 | 0,21 | 0,9 |
| Erval Grande | RS | 1 | 4.859 | 0,21 | 0,6 |
| Humaitá | RS | 1 | 4.762 | 0,21 | 0,0 |
| São Jerônimo | RS | 5 | 24.248 | 0,21 | 17,0 |
| São Luiz Gonzaga | RS | 7 | 33.468 | 0,21 | 1,0 |
| Vicente Dutra | RS | 1 | 4.670 | 0,21 | 0,0 |
| São Pedro da Cipa | MT | 1 | 4.727 | 0,21 | 100,0 |
| Goiatuba | GO | 7 | 34.095 | 0,21 | 0,4 |
| Palmeiras de Goiás | GO | 6 | 28.858 | 0,21 | 0,0 |
| Paraíso do Tocantins | TO | 10 | 51.252 | 0,2 | 0,1 |
| Cândido Mendes | MA | 4 | 20.178 | 0,2 | 98,8 |
| Cidelândia | MA | 3 | 14.697 | 0,2 | 12,6 |
| Governador Newton Bello | MA | 2 | 10.180 | 0,2 | 53,4 |
| Mirinzal | MA | 3 | 14.962 | 0,2 | 0,9 |
| Caridade do Piauí | PI | 1 | 5.067 | 0,2 | 57,6 |
| Castelo do Piauí | PI | 4 | 19.716 | 0,2 | 4,3 |
| Currais | PI | 1 | 4.954 | 0,2 | 19,3 |
| Ipiranga do Piauí | PI | 2 | 9.811 | 0,2 | 0,4 |
| Aurora | CE | 5 | 24.654 | 0,2 | 11,6 |
| Pedro Velho | RN | 3 | 14.806 | 0,2 | 14,1 |
| Pendências | RN | 3 | 15.129 | 0,2 | 91,1 |
| Rafael Fernandes | RN | 1 | 5.098 | 0,2 | 100,0 |
| Água Branca | PB | 2 | 10.234 | 0,2 | 0,7 |
| Barra de Santa Rosa | PB | 3 | 15.384 | 0,2 | 26,3 |
| Bom Sucesso | PB | 1 | 4.975 | 0,2 | 57,7 |
| Mulungu | PB | 2 | 9.902 | 0,2 | 0,0 |
| Pilõezinhos | PB | 1 | 4.976 | 0,2 | 0,0 |
| Capoeiras | PE | 4 | 20.048 | 0,2 | 17,3 |
| Itacuruba | PE | 1 | 4.918 | 0,2 | 1,4 |
| Limoeiro | PE | 11 | 56.250 | 0,2 | 1,5 |
| Trindade | PE | 6 | 30.521 | 0,2 | 0,0 |
| Água Branca | AL | 4 | 20.196 | 0,2 | 0,0 |
| Pilar | AL | 7 | 35.111 | 0,2 | 0,0 |
| São Luís do Quitunde | AL | 7 | 34.555 | 0,2 | 1,9 |
| Itabi | SE | 1 | 4.903 | 0,2 | 4,1 |
| Abaré | BA | 4 | 20.086 | 0,2 | 6,9 |
| Acajutiba | BA | 3 | 15.159 | 0,2 | 0,0 |
| Amélia Rodrigues | BA | 5 | 25.102 | 0,2 | 0,0 |
| Piraí do Norte | BA | 2 | 10.023 | 0,2 | 0,0 |
| Presidente Dutra | BA | 3 | 15.142 | 0,2 | 0,0 |
| Carandaí | MG | 5 | 25.501 | 0,2 | 0,0 |
| Carneirinho | MG | 2 | 10.027 | 0,2 | 2,9 |
| Entre Rios de Minas | MG | 3 | 15.298 | 0,2 | 0,0 |
| Gameleiras | MG | 1 | 5.109 | 0,2 | 11,1 |
| Itamogi | MG | 2 | 10.192 | 0,2 | 0,0 |
| Jeceaba | MG | 1 | 4.912 | 0,2 | 0,0 |
| Madre de Deus de Minas | MG | 1 | 5.098 | 0,2 | 0,0 |
| Miravânia | MG | 1 | 4.888 | 0,2 | 2,2 |
| Padre Paraíso | MG | 4 | 20.154 | 0,2 | 2,9 |
| Paraopeba | MG | 5 | 24.540 | 0,2 | 0,6 |
| Santa Rita de Jacutinga | MG | 1 | 4.884 | 0,2 | 0,0 |
| Turmalina | MG | 4 | 19.964 | 0,2 | 1,1 |
| Turvolândia | MG | 1 | 5.040 | 0,2 | 0,0 |
| Aracruz | ES | 20 | 101.220 | 0,2 | 0,0 |
| Sooretama | ES | 6 | 30.070 | 0,2 | 0,0 |
| Angatuba | SP | 5 | 25.228 | 0,2 | 0,3 |
| Campos Novos Paulista | SP | 1 | 4.965 | 0,2 | 3,6 |
| Luís Antônio | SP | 3 | 14.947 | 0,2 | 0,0 |
| Manduri | SP | 2 | 9.846 | 0,2 | 0,0 |
| Santa Branca | SP | 3 | 14.788 | 0,2 | 0,0 |
| Lupionópolis | PR | 1 | 4.920 | 0,2 | 0,0 |
| Nova Esperança do Sudoeste | PR | 1 | 5.046 | 0,2 | 0,0 |
| Piraí do Sul | PR | 5 | 25.463 | 0,2 | 0,0 |
| Rebouças | PR | 3 | 14.899 | 0,2 | 0,0 |
| Santo Antônio do Sudoeste | PR | 4 | 20.166 | 0,2 | 0,0 |
| Tamarana | PR | 3 | 14.797 | 0,2 | 0,0 |
| Coronel Freitas | SC | 2 | 9.981 | 0,2 | 0,0 |
| Guaramirim | SC | 9 | 44.819 | 0,2 | 0,0 |
| Nova Erechim | SC | 1 | 5.019 | 0,2 | 0,0 |
| Brochier | RS | 1 | 5.074 | 0,2 | 0,0 |
| Cacique Doble | RS | 1 | 5.065 | 0,2 | 5,8 |
| Miraguaí | RS | 1 | 4.925 | 0,2 | 0,0 |
| Selbach | RS | 1 | 5.100 | 0,2 | 0,2 |
| Apiacás | MT | 2 | 10.133 | 0,2 | 7,5 |
| Bela Vista de Goiás | GO | 6 | 29.975 | 0,2 | 0,0 |
| Bom Jesus de Goiás | GO | 5 | 25.216 | 0,2 | 1,3 |
| Campos Belos | GO | 4 | 19.887 | 0,2 | 0,0 |
| Caturaí | GO | 1 | 5.070 | 0,2 | 0,0 |
| Itapuã do Oeste | RO | 2 | 10.458 | 0,19 | 97,4 |
| São Félix do Xingu | PA | 24 | 128.481 | 0,19 | 24,0 |
| Silvanópolis | TO | 1 | 5.403 | 0,19 | 0,9 |
| São João dos Patos | MA | 5 | 25.929 | 0,19 | 8,1 |
| Batalha | PI | 5 | 26.857 | 0,19 | 3,0 |
| Cristino Castro | PI | 2 | 10.423 | 0,19 | 8,8 |
| Curral Novo do Piauí | PI | 1 | 5.343 | 0,19 | 99,9 |
| Monsenhor Gil | PI | 2 | 10.564 | 0,19 | 0,0 |
| Parnaguá | PI | 2 | 10.791 | 0,19 | 100,0 |
| Ocara | CE | 5 | 25.703 | 0,19 | 13,6 |
| Carnaubais | RN | 2 | 10.759 | 0,19 | 81,9 |
| Janduís | RN | 1 | 5.268 | 0,19 | 21,9 |
| São Miguel do Gostoso | RN | 2 | 10.282 | 0,19 | 0,0 |
| Umarizal | RN | 2 | 10.555 | 0,19 | 92,5 |
| Curral de Cima | PB | 1 | 5.227 | 0,19 | 55,4 |
| Santana de Mangueira | PB | 1 | 5.162 | 0,19 | 17,2 |
| Solânea | PB | 5 | 26.407 | 0,19 | 4,4 |
| Belém do São Francisco | PE | 4 | 20.729 | 0,19 | 6,5 |
| Joaquim Nabuco | PE | 3 | 16.023 | 0,19 | 2,3 |
| Panelas | PE | 5 | 26.474 | 0,19 | 1,8 |
| São José da Coroa Grande | PE | 4 | 21.298 | 0,19 | 100,0 |
| Estância | SE | 13 | 69.184 | 0,19 | 0,0 |
| América Dourada | BA | 3 | 16.094 | 0,19 | 0,4 |
| Barreiras | BA | 29 | 155.439 | 0,19 | 0,7 |
| Boquira | BA | 4 | 21.508 | 0,19 | 17,5 |
| Canarana | BA | 5 | 26.176 | 0,19 | 0,0 |
| Conde | BA | 5 | 25.837 | 0,19 | 0,4 |
| Coronel João Sá | BA | 3 | 15.895 | 0,19 | 11,5 |
| Ibotirama | BA | 5 | 26.927 | 0,19 | 2,9 |
| Igaporã | BA | 3 | 15.640 | 0,19 | 0,0 |
| Maracás | BA | 4 | 20.834 | 0,19 | 10,9 |
| Maraú | BA | 4 | 20.570 | 0,19 | 0,3 |
| Mucuri | BA | 8 | 41.748 | 0,19 | 0,2 |
| Poções | BA | 9 | 46.871 | 0,19 | 2,6 |
| Santa Inês | BA | 2 | 10.631 | 0,19 | 0,5 |
| São Félix do Coribe | BA | 3 | 15.391 | 0,19 | 5,6 |
| São Felipe | BA | 4 | 21.074 | 0,19 | 0,0 |
| Wenceslau Guimarães | BA | 4 | 21.101 | 0,19 | 1,2 |
| Arceburgo | MG | 2 | 10.772 | 0,19 | 0,0 |
| Barra Longa | MG | 1 | 5.131 | 0,19 | 0,0 |
| Belo Oriente | MG | 5 | 26.700 | 0,19 | 0,0 |
| Campos Altos | MG | 3 | 15.461 | 0,19 | 0,1 |
| Ibiracatu | MG | 1 | 5.400 | 0,19 | 0,4 |
| Jampruca | MG | 1 | 5.404 | 0,19 | 15,4 |
| Jequitibá | MG | 1 | 5.211 | 0,19 | 8,0 |
| Perdizes | MG | 3 | 16.168 | 0,19 | 3,0 |
| Rio Pardo de Minas | MG | 6 | 30.914 | 0,19 | 13,5 |
| Santos Dumont | MG | 9 | 46.487 | 0,19 | 0,0 |
| São Bento Abade | MG | 1 | 5.286 | 0,19 | 0,0 |
| Urucânia | MG | 2 | 10.358 | 0,19 | 0,0 |
| Volta Grande | MG | 1 | 5.252 | 0,19 | 0,0 |
| Linhares | ES | 33 | 173.555 | 0,19 | 0,0 |
| Cambuci | RJ | 3 | 15.505 | 0,19 | 0,5 |
| Álvaro de Carvalho | SP | 1 | 5.227 | 0,19 | 0,0 |
| Avaí | SP | 1 | 5.403 | 0,19 | 0,1 |
| Canas | SP | 1 | 5.138 | 0,19 | 0,0 |
| Ilha Solteira | SP | 5 | 26.686 | 0,19 | 0,0 |
| Ipiguá | SP | 1 | 5.392 | 0,19 | 0,0 |
| Nipoã | SP | 1 | 5.213 | 0,19 | 0,0 |
| Rincão | SP | 2 | 10.799 | 0,19 | 0,0 |
| Salmourão | SP | 1 | 5.300 | 0,19 | 0,0 |
| São Sebastião | SP | 17 | 88.980 | 0,19 | 0,0 |
| Sarapuí | SP | 2 | 10.285 | 0,19 | 0,0 |
| Coronel Vivida | PR | 4 | 20.734 | 0,19 | 0,0 |
| Jaboti | PR | 1 | 5.274 | 0,19 | 0,0 |
| Janiópolis | PR | 1 | 5.245 | 0,19 | 0,0 |
| Mauá da Serra | PR | 2 | 10.601 | 0,19 | 0,0 |
| Nova Cantu | PR | 1 | 5.302 | 0,19 | 0,0 |
| Pranchita | PR | 1 | 5.157 | 0,19 | 0,0 |
| Reserva | PR | 5 | 26.715 | 0,19 | 1,0 |
| Ribeirão Claro | PR | 2 | 10.668 | 0,19 | 0,0 |
| Tapejara | PR | 3 | 16.205 | 0,19 | 0,0 |
| Balneário Barra do Sul | SC | 2 | 10.795 | 0,19 | 0,0 |
| Guarujá do Sul | SC | 1 | 5.160 | 0,19 | 0,0 |
| Itapoá | SC | 4 | 20.576 | 0,19 | 0,0 |
| Palmitos | SC | 3 | 16.169 | 0,19 | 0,0 |
| Porto Belo | SC | 4 | 21.388 | 0,19 | 0,0 |
| Timbé do Sul | SC | 1 | 5.348 | 0,19 | 0,0 |
| Barracão | RS | 1 | 5.275 | 0,19 | 0,2 |
| Giruá | RS | 3 | 16.004 | 0,19 | 0,0 |
| Ronda Alta | RS | 2 | 10.601 | 0,19 | 2,6 |
| São Gabriel | RS | 12 | 62.105 | 0,19 | 97,6 |
| São Nicolau | RS | 1 | 5.265 | 0,19 | 30,6 |
| Seberi | RS | 2 | 10.750 | 0,19 | 0,0 |
| Água Clara | MS | 3 | 15.522 | 0,19 | 11,8 |
| Aparecida do Taboado | MS | 5 | 25.745 | 0,19 | 1,4 |
| Corumbá | MS | 21 | 111.435 | 0,19 | 5,4 |
| Santo Antônio do Leste | MT | 1 | 5.174 | 0,19 | 100,0 |
| Goianésia | GO | 13 | 70.084 | 0,19 | 0,0 |
| São Luíz do Norte | GO | 1 | 5.167 | 0,19 | 1,2 |
| Barrolândia | TO | 1 | 5.632 | 0,18 | 3,8 |
| Taguatinga | TO | 3 | 16.683 | 0,18 | 8,5 |
| Bacurituba | MA | 1 | 5.644 | 0,18 | 11,7 |
| Bela Vista do Maranhão | MA | 2 | 11.209 | 0,18 | 6,3 |
| Benedito Leite | MA | 1 | 5.632 | 0,18 | 11,5 |
| Mata Roma | MA | 3 | 16.829 | 0,18 | 8,2 |
| São Bento | MA | 8 | 45.211 | 0,18 | 2,8 |
| Bertolínia | PI | 1 | 5.501 | 0,18 | 19,0 |
| Curimatá | PI | 2 | 11.388 | 0,18 | 98,7 |
| Juazeiro do Piauí | PI | 1 | 5.478 | 0,18 | 24,3 |
| Ararendá | CE | 2 | 10.935 | 0,18 | 62,3 |
| Barbalha | CE | 11 | 60.781 | 0,18 | 0,7 |
| Campos Sales | CE | 5 | 27.426 | 0,18 | 100,0 |
| Ipueiras | CE | 7 | 38.166 | 0,18 | 98,3 |
| Martinópole | CE | 2 | 11.233 | 0,18 | 17,8 |
| Milagres | CE | 5 | 27.512 | 0,18 | 5,8 |
| Potengi | CE | 2 | 11.045 | 0,18 | 24,6 |
| Bento Fernandes | RN | 1 | 5.497 | 0,18 | 0,8 |
| Rio do Fogo | RN | 2 | 10.848 | 0,18 | 0,0 |
| Alcantil | PB | 1 | 5.492 | 0,18 | 31,8 |
| Lagoa Seca | PB | 5 | 27.503 | 0,18 | 0,0 |
| Manaíra | PB | 2 | 10.955 | 0,18 | 0,0 |
| Montadas | PB | 1 | 5.669 | 0,18 | 0,9 |
| Goiana | PE | 14 | 79.758 | 0,18 | 0,1 |
| Lagoa dos Gatos | PE | 3 | 16.290 | 0,18 | 14,2 |
| Boca da Mata | AL | 5 | 27.281 | 0,18 | 0,0 |
| Maragogi | AL | 6 | 32.704 | 0,18 | 100,0 |
| Água Fria | BA | 3 | 16.970 | 0,18 | 7,3 |
| Anguera | BA | 2 | 11.221 | 0,18 | 0,0 |
| Conceição do Jacuípe | BA | 6 | 33.153 | 0,18 | 0,0 |
| Gentio do Ouro | BA | 2 | 11.233 | 0,18 | 14,3 |
| Jaguarari | BA | 6 | 33.570 | 0,18 | 4,7 |
| Jequié | BA | 28 | 155.966 | 0,18 | 1,4 |
| Lençóis | BA | 2 | 11.409 | 0,18 | 8,4 |
| Milagres | BA | 2 | 10.950 | 0,18 | 0,9 |
| Rafael Jambeiro | BA | 4 | 22.621 | 0,18 | 2,2 |
| Araxá | MG | 19 | 106.229 | 0,18 | 0,0 |
| Brasilândia de Minas | MG | 3 | 16.538 | 0,18 | 2,6 |
| Conceição do Pará | MG | 1 | 5.507 | 0,18 | 0,0 |
| Itaverava | MG | 1 | 5.419 | 0,18 | 0,0 |
| Iturama | MG | 7 | 39.263 | 0,18 | 0,0 |
| Nova Resende | MG | 3 | 16.723 | 0,18 | 7,9 |
| Palmópolis | MG | 1 | 5.507 | 0,18 | 0,5 |
| Piranguçu | MG | 1 | 5.472 | 0,18 | 0,0 |
| Pitangui | MG | 5 | 27.989 | 0,18 | 0,0 |
| Porteirinha | MG | 7 | 37.906 | 0,18 | 2,0 |
| Santa Rita do Sapucaí | MG | 8 | 43.260 | 0,18 | 0,1 |
| Santo Antônio do Monte | MG | 5 | 28.243 | 0,18 | 0,0 |
| São Gonçalo do Rio Abaixo | MG | 2 | 10.920 | 0,18 | 0,0 |
| São Tiago | MG | 2 | 10.941 | 0,18 | 0,2 |
| Sobrália | MG | 1 | 5.553 | 0,18 | 0,0 |
| Angra dos Reis | RJ | 37 | 203.785 | 0,18 | 2,1 |
| Macuco | RJ | 1 | 5.599 | 0,18 | 0,0 |
| Biritiba-Mirim | SP | 6 | 32.598 | 0,18 | 0,0 |
| Braúna | SP | 1 | 5.686 | 0,18 | 0,0 |
| Getulina | SP | 2 | 11.409 | 0,18 | 0,0 |
| Ilha Comprida | SP | 2 | 11.166 | 0,18 | 0,0 |
| Ribeirão Branco | SP | 3 | 16.444 | 0,18 | 0,4 |
| Santa Ernestina | SP | 1 | 5.599 | 0,18 | 0,0 |
| Santa Rita do Passa Quatro | SP | 5 | 27.557 | 0,18 | 0,0 |
| São Bento do Sapucaí | SP | 2 | 10.878 | 0,18 | 0,0 |
| Braganey | PR | 1 | 5.427 | 0,18 | 0,1 |
| Fernandes Pinheiro | PR | 1 | 5.646 | 0,18 | 0,8 |
| Maripá | PR | 1 | 5.603 | 0,18 | 0,0 |
| Mercedes | PR | 1 | 5.536 | 0,18 | 0,0 |
| Palmeira | PR | 6 | 33.877 | 0,18 | 0,1 |
| Saudade do Iguaçu | PR | 1 | 5.500 | 0,18 | 0,0 |
| Sertanópolis | PR | 3 | 16.369 | 0,18 | 0,0 |
| Xambrê | PR | 1 | 5.679 | 0,18 | 0,0 |
| Agronômica | SC | 1 | 5.448 | 0,18 | 0,0 |
| Capinzal | SC | 4 | 22.848 | 0,18 | 0,0 |
| Imbituba | SC | 8 | 44.853 | 0,18 | 0,0 |
| Itaiópolis | SC | 4 | 21.669 | 0,18 | 0,1 |
| Luzerna | SC | 1 | 5.685 | 0,18 | 0,0 |
| São Cristovão do Sul | SC | 1 | 5.549 | 0,18 | 0,0 |
| Araricá | RS | 1 | 5.698 | 0,18 | 0,0 |
| Caçapava do Sul | RS | 6 | 33.624 | 0,18 | 15,5 |
| Campinas do Sul | RS | 1 | 5.454 | 0,18 | 0,1 |
| Jaguari | RS | 2 | 10.848 | 0,18 | 100,0 |
| Passa Sete | RS | 1 | 5.702 | 0,18 | 17,1 |
| São Borja | RS | 11 | 60.282 | 0,18 | 100,0 |
| Tavares | RS | 1 | 5.481 | 0,18 | 100,0 |
| Nova Alvorada do Sul | MS | 4 | 21.882 | 0,18 | 7,1 |
| Cocalinho | MT | 1 | 5.700 | 0,18 | 14,9 |
| Goianápolis | GO | 2 | 11.231 | 0,18 | 0,0 |
| Goiandira | GO | 1 | 5.600 | 0,18 | 0,6 |
| Padre Bernardo | GO | 6 | 33.835 | 0,18 | 0,9 |
| Santa Fé de Goiás | GO | 1 | 5.459 | 0,18 | 1,1 |
| Vilhena | RO | 17 | 99.854 | 0,17 | 1,4 |
| Urupá | RO | 2 | 11.467 | 0,17 | 100,0 |
| Castanhal | PA | 34 | 200.793 | 0,17 | 0,0 |
| Gurupi | TO | 15 | 86.647 | 0,17 | 1,0 |
| Apicum-Açu | MA | 3 | 17.239 | 0,17 | 0,1 |
| Dom Pedro | MA | 4 | 23.350 | 0,17 | 0,0 |
| Baixa Grande do Ribeiro | PI | 2 | 11.586 | 0,17 | 100,0 |
| Santa Luz | PI | 1 | 5.860 | 0,17 | 4,1 |
| Capistrano | CE | 3 | 17.738 | 0,17 | 5,0 |
| Crateús | CE | 13 | 75.074 | 0,17 | 97,5 |
| José da Penha | RN | 1 | 5.951 | 0,17 | 100,0 |
| São Pedro | RN | 1 | 5.971 | 0,17 | 0,0 |
| Barra de São Miguel | PB | 1 | 6.036 | 0,17 | 98,5 |
| Ibiara | PB | 1 | 5.929 | 0,17 | 11,6 |
| Malta | PB | 1 | 5.759 | 0,17 | 0,8 |
| Nova Olinda | PB | 1 | 5.949 | 0,17 | 0,0 |
| Santa Helena | PB | 1 | 5.889 | 0,17 | 100,0 |
| Araripina | PE | 14 | 84.418 | 0,17 | 8,8 |
| Caetés | PE | 5 | 28.739 | 0,17 | 16,0 |
| Calumbi | PE | 1 | 5.750 | 0,17 | 0,0 |
| Jataúba | PE | 3 | 17.150 | 0,17 | 33,5 |
| Paranatama | PE | 2 | 11.523 | 0,17 | 28,7 |
| Penedo | AL | 11 | 63.683 | 0,17 | 0,0 |
| Cristinápolis | SE | 3 | 17.874 | 0,17 | 0,0 |
| Cairu | BA | 3 | 18.176 | 0,17 | 23,8 |
| Cansanção | BA | 6 | 34.834 | 0,17 | 1,0 |
| Ipirá | BA | 10 | 59.595 | 0,17 | 10,3 |
| Itiúba | BA | 6 | 36.089 | 0,17 | 21,5 |
| São Miguel das Matas | BA | 2 | 11.675 | 0,17 | 0,0 |
| Sapeaçu | BA | 3 | 17.398 | 0,17 | 0,0 |
| Sátiro Dias | BA | 3 | 17.273 | 0,17 | 5,2 |
| Uauá | BA | 4 | 24.240 | 0,17 | 13,6 |
| Aiuruoca | MG | 1 | 6.003 | 0,17 | 0,6 |
| Bandeira do Sul | MG | 1 | 5.746 | 0,17 | 0,0 |
| Campo do Meio | MG | 2 | 11.655 | 0,17 | 0,0 |
| Cláudio | MG | 5 | 28.617 | 0,17 | 0,0 |
| Conceição do Mato Dentro | MG | 3 | 17.842 | 0,17 | 6,3 |
| Ituiutaba | MG | 18 | 104.671 | 0,17 | 0,0 |
| Paracatu | MG | 16 | 93.158 | 0,17 | 3,2 |
| Resplendor | MG | 3 | 17.397 | 0,17 | 0,0 |
| Santo Antônio do Jacinto | MG | 2 | 11.640 | 0,17 | 0,5 |
| São Gotardo | MG | 6 | 35.469 | 0,17 | 0,5 |
| São Sebastião do Paraíso | MG | 12 | 70.956 | 0,17 | 0,0 |
| Teixeiras | MG | 2 | 11.661 | 0,17 | 0,0 |
| Teófilo Otoni | MG | 24 | 140.592 | 0,17 | 2,3 |
| Veredinha | MG | 1 | 5.720 | 0,17 | 0,3 |
| Itaocara | RJ | 4 | 23.234 | 0,17 | 0,6 |
| Nova Friburgo | RJ | 33 | 190.631 | 0,17 | 0,0 |
| Amparo | SP | 12 | 72.195 | 0,17 | 0,0 |
| Bofete | SP | 2 | 11.730 | 0,17 | 1,2 |
| Conchas | SP | 3 | 17.896 | 0,17 | 0,0 |
| Itanhaém | SP | 17 | 101.816 | 0,17 | 0,0 |
| Marabá Paulista | SP | 1 | 5.853 | 0,17 | 0,0 |
| Mirandópolis | SP | 5 | 29.564 | 0,17 | 0,1 |
| Santo Antônio do Jardim | SP | 1 | 5.954 | 0,17 | 0,0 |
| Severínia | SP | 3 | 17.496 | 0,17 | 0,0 |
| Tremembé | SP | 8 | 47.185 | 0,17 | 0,0 |
| Barbosa Ferraz | PR | 2 | 11.568 | 0,17 | 0,0 |
| Três Barras do Paraná | PR | 2 | 12.040 | 0,17 | 0,0 |
| Abelardo Luz | SC | 3 | 17.904 | 0,17 | 0,0 |
| Benedito Novo | SC | 2 | 11.652 | 0,17 | 0,0 |
| Mondaí | SC | 2 | 11.742 | 0,17 | 0,0 |
| Ponte Serrada | SC | 2 | 11.593 | 0,17 | 0,0 |
| Pouso Redondo | SC | 3 | 17.453 | 0,17 | 0,0 |
| Rio do Campo | SC | 1 | 5.940 | 0,17 | 2,2 |
| Rodeio | SC | 2 | 11.551 | 0,17 | 0,0 |
| São Francisco do Sul | SC | 9 | 52.721 | 0,17 | 0,0 |
| São Pedro de Alcântara | SC | 1 | 5.823 | 0,17 | 0,0 |
| Anta Gorda | RS | 1 | 5.981 | 0,17 | 1,4 |
| Cachoeira do Sul | RS | 14 | 82.201 | 0,17 | 0,6 |
| Capela de Santana | RS | 2 | 11.940 | 0,17 | 0,0 |
| Lindolfo Collor | RS | 1 | 6.054 | 0,17 | 0,0 |
| Rodeio Bonito | RS | 1 | 5.867 | 0,17 | 0,0 |
| Três de Maio | RS | 4 | 23.906 | 0,17 | 0,0 |
| Trindade do Sul | RS | 1 | 5.802 | 0,17 | 4,1 |
| Douradina | MS | 1 | 5.924 | 0,17 | 0,0 |
| Tacuru | MS | 2 | 11.552 | 0,17 | 0,2 |
| Lucas do Rio Verde | MT | 11 | 65.534 | 0,17 | 99,0 |
| Santa Izabel do Pará | PA | 11 | 70.801 | 0,16 | 0,0 |
| Xinguara | PA | 7 | 44.751 | 0,16 | 6,1 |
| Bacuri | MA | 3 | 18.582 | 0,16 | 11,2 |
| São Francisco do Maranhão | MA | 2 | 12.210 | 0,16 | 15,8 |
| Betânia do Piauí | PI | 1 | 6.205 | 0,16 | 31,0 |
| Cocal dos Alves | PI | 1 | 6.153 | 0,16 | 28,6 |
| Massapê do Piauí | PI | 1 | 6.441 | 0,16 | 58,6 |
| Oeiras | PI | 6 | 37.029 | 0,16 | 3,9 |
| Pimenteiras | PI | 2 | 12.127 | 0,16 | 37,0 |
| Porto | PI | 2 | 12.568 | 0,16 | 1,1 |
| Santa Filomena | PI | 1 | 6.253 | 0,16 | 17,1 |
| Santo Antônio de Lisboa | PI | 1 | 6.415 | 0,16 | 2,9 |
| Ibiapina | CE | 4 | 24.997 | 0,16 | 0,6 |
| Pentecoste | CE | 6 | 37.751 | 0,16 | 21,0 |
| Caicó | RN | 11 | 67.952 | 0,16 | 0,3 |
| Pau dos Ferros | RN | 5 | 30.394 | 0,16 | 100,0 |
| Taipu | RN | 2 | 12.279 | 0,16 | 0,0 |
| Conde | PB | 4 | 24.670 | 0,16 | 0,0 |
| Pedro Régis | PB | 1 | 6.089 | 0,16 | 32,9 |
| Salgado de São Félix | PB | 2 | 12.140 | 0,16 | 0,0 |
| Águas Belas | PE | 7 | 43.443 | 0,16 | 32,5 |
| Gameleira | PE | 5 | 31.052 | 0,16 | 12,6 |
| Batalha | AL | 3 | 18.232 | 0,16 | 0,1 |
| Igreja Nova | AL | 4 | 24.499 | 0,16 | 0,0 |
| Matriz de Camaragibe | AL | 4 | 24.642 | 0,16 | 2,9 |
| Piranhas | AL | 4 | 25.039 | 0,16 | 0,0 |
| Teotônio Vilela | AL | 7 | 44.169 | 0,16 | 0,0 |
| Areia Branca | SE | 3 | 18.542 | 0,16 | 0,0 |
| Cabaceiras do Paraguaçu | BA | 3 | 18.807 | 0,16 | 0,0 |
| Cândido Sales | BA | 4 | 25.189 | 0,16 | 0,1 |
| Itanagra | BA | 1 | 6.441 | 0,16 | 3,9 |
| Itapetinga | BA | 12 | 76.147 | 0,16 | 0,0 |
| Jussiape | BA | 1 | 6.186 | 0,16 | 0,0 |
| Macaúbas | BA | 8 | 49.826 | 0,16 | 6,9 |
| Nova Viçosa | BA | 7 | 43.376 | 0,16 | 1,3 |
| Novo Horizonte | BA | 2 | 12.385 | 0,16 | 0,0 |
| São Gabriel | BA | 3 | 18.793 | 0,16 | 7,0 |
| Várzea Nova | BA | 2 | 12.697 | 0,16 | 1,9 |
| Andrelândia | MG | 2 | 12.224 | 0,16 | 0,1 |
| Araçuaí | MG | 6 | 36.708 | 0,16 | 7,2 |
| Baependi | MG | 3 | 19.148 | 0,16 | 0,0 |
| Botumirim | MG | 1 | 6.319 | 0,16 | 29,8 |
| Carmo do Paranaíba | MG | 5 | 30.329 | 0,16 | 1,9 |
| Cristais | MG | 2 | 12.798 | 0,16 | 0,0 |
| Itanhomi | MG | 2 | 12.228 | 0,16 | 0,0 |
| Padre Carvalho | MG | 1 | 6.378 | 0,16 | 4,2 |
| São Geraldo | MG | 2 | 12.366 | 0,16 | 0,0 |
| São Gonçalo do Pará | MG | 2 | 12.411 | 0,16 | 0,0 |
| São Gonçalo do Sapucaí | MG | 4 | 25.449 | 0,16 | 0,0 |
| Soledade de Minas | MG | 1 | 6.151 | 0,16 | 0,0 |
| Venda Nova do Imigrante | ES | 4 | 25.277 | 0,16 | 0,0 |
| Porciúncula | RJ | 3 | 18.847 | 0,16 | 0,0 |
| Valença | RJ | 12 | 76.523 | 0,16 | 0,0 |
| Echaporã | SP | 1 | 6.102 | 0,16 | 0,9 |
| Ibirá | SP | 2 | 12.393 | 0,16 | 0,0 |
| Pedra Bela | SP | 1 | 6.093 | 0,16 | 0,0 |
| Peruíbe | SP | 11 | 68.284 | 0,16 | 0,0 |
| Riolândia | SP | 2 | 12.518 | 0,16 | 0,0 |
| Sengés | PR | 3 | 19.327 | 0,16 | 0,4 |
| Correia Pinto | SC | 2 | 12.795 | 0,16 | 0,3 |
| Rio das Antas | SC | 1 | 6.205 | 0,16 | 0,0 |
| São João do Oeste | SC | 1 | 6.381 | 0,16 | 0,0 |
| Taió | SC | 3 | 18.395 | 0,16 | 0,0 |
| Bossoroca | RS | 1 | 6.279 | 0,16 | 86,2 |
| Horizontina | RS | 3 | 19.329 | 0,16 | 0,0 |
| Progresso | RS | 1 | 6.244 | 0,16 | 0,0 |
| Rio Pardo | RS | 6 | 38.275 | 0,16 | 0,1 |
| Eldorado | MS | 2 | 12.353 | 0,16 | 0,1 |
| Ribas do Rio Pardo | MS | 4 | 24.615 | 0,16 | 17,7 |
| Alto Araguaia | MT | 3 | 19.044 | 0,16 | 9,6 |
| Lambari D'Oeste | MT | 1 | 6.121 | 0,16 | 4,1 |
| Americano do Brasil | GO | 1 | 6.111 | 0,16 | 0,0 |
| Itapuranga | GO | 4 | 25.768 | 0,16 | 0,0 |
| Palmas | TO | 46 | 299.127 | 0,15 | 0,0 |
| Alto Alegre do Maranhão | MA | 4 | 27.053 | 0,15 | 0,1 |
| Chapadinha | MA | 12 | 79.675 | 0,15 | 9,2 |
| Colinas | MA | 6 | 41.178 | 0,15 | 19,7 |
| Altos | PI | 6 | 40.524 | 0,15 | 0,8 |
| Barras | PI | 7 | 47.066 | 0,15 | 4,6 |
| Buriti dos Lopes | PI | 3 | 19.781 | 0,15 | 0,1 |
| Colônia do Gurguéia | PI | 1 | 6.484 | 0,15 | 100,0 |
| Lagoa de São Francisco | PI | 1 | 6.758 | 0,15 | 0,0 |
| Novo Oriente do Piauí | PI | 1 | 6.565 | 0,15 | 9,0 |
| Padre Marcos | PI | 1 | 6.868 | 0,15 | 18,4 |
| São José do Piauí | PI | 1 | 6.705 | 0,15 | 0,1 |
| Icó | CE | 10 | 68.018 | 0,15 | 100,0 |
| Milhã | CE | 2 | 13.155 | 0,15 | 100,0 |
| Nova Russas | CE | 5 | 32.328 | 0,15 | 100,0 |
| Goianinha | RN | 4 | 26.328 | 0,15 | 0,0 |
| Lagoa d'Anta | RN | 1 | 6.769 | 0,15 | 0,0 |
| Arara | PB | 2 | 13.470 | 0,15 | 15,9 |
| Condado | PB | 1 | 6.654 | 0,15 | 19,3 |
| Cuitegi | PB | 1 | 6.803 | 0,15 | 0,0 |
| Lucena | PB | 2 | 13.080 | 0,15 | 0,0 |
| Maturéia | PB | 1 | 6.569 | 0,15 | 2,7 |
| Araçoiaba | PE | 3 | 20.524 | 0,15 | 0,0 |
| Buenos Aires | PE | 2 | 13.155 | 0,15 | 3,7 |
| João Alfredo | PE | 5 | 33.822 | 0,15 | 0,2 |
| Pombos | PE | 4 | 27.091 | 0,15 | 0,0 |
| São José do Egito | PE | 5 | 33.951 | 0,15 | 22,6 |
| Paripueira | AL | 2 | 13.176 | 0,15 | 0,0 |
| União dos Palmares | AL | 10 | 65.611 | 0,15 | 1,4 |
| Pinhão | SE | 1 | 6.576 | 0,15 | 0,0 |
| Salgado | SE | 3 | 19.998 | 0,15 | 0,0 |
| Campo Formoso | BA | 11 | 71.206 | 0,15 | 21,9 |
| Itabuna | BA | 33 | 213.223 | 0,15 | 0,0 |
| Itororó | BA | 3 | 20.382 | 0,15 | 0,0 |
| Nova Ibiá | BA | 1 | 6.591 | 0,15 | 0,0 |
| Santa Maria da Vitória | BA | 6 | 39.845 | 0,15 | 8,8 |
| Serrolândia | BA | 2 | 13.397 | 0,15 | 0,0 |
| Ubatã | BA | 4 | 27.035 | 0,15 | 12,7 |
| Alpinópolis | MG | 3 | 19.853 | 0,15 | 0,0 |
| Carmópolis de Minas | MG | 3 | 19.355 | 0,15 | 0,0 |
| Conceição do Rio Verde | MG | 2 | 13.638 | 0,15 | 0,0 |
| Indianópolis | MG | 1 | 6.891 | 0,15 | 2,9 |
| Martinho Campos | MG | 2 | 13.388 | 0,15 | 0,7 |
| Nanuque | MG | 6 | 40.750 | 0,15 | 0,5 |
| Ouro Fino | MG | 5 | 33.639 | 0,15 | 0,0 |
| Palma | MG | 1 | 6.616 | 0,15 | 0,0 |
| São Sebastião do Oeste | MG | 1 | 6.775 | 0,15 | 0,0 |
| Virgem da Lapa | MG | 2 | 13.752 | 0,15 | 15,1 |
| Itapemirim | ES | 5 | 34.348 | 0,15 | 0,0 |
| Bom Jardim | RJ | 4 | 27.446 | 0,15 | 0,0 |
| Porto Real | RJ | 3 | 19.683 | 0,15 | 0,0 |
| Tanguá | RJ | 5 | 34.309 | 0,15 | 0,0 |
| Teresópolis | RJ | 27 | 182.594 | 0,15 | 0,0 |
| Gália | SP | 1 | 6.548 | 0,15 | 0,2 |
| Guarantã | SP | 1 | 6.664 | 0,15 | 0,0 |
| Itirapuã | SP | 1 | 6.499 | 0,15 | 0,0 |
| Joanópolis | SP | 2 | 13.220 | 0,15 | 0,0 |
| Oriente | SP | 1 | 6.515 | 0,15 | 0,0 |
| Presidente Bernardes | SP | 2 | 13.106 | 0,15 | 0,2 |
| Santo Antônio do Pinhal | SP | 1 | 6.811 | 0,15 | 0,0 |
| Tapiratiba | SP | 2 | 12.960 | 0,15 | 0,0 |
| Tuiuti | SP | 1 | 6.894 | 0,15 | 0,0 |
| Borrazópolis | PR | 1 | 6.592 | 0,15 | 0,0 |
| Formosa do Oeste | PR | 1 | 6.578 | 0,15 | 0,0 |
| General Carneiro | PR | 2 | 13.710 | 0,15 | 1,8 |
| Mamborê | PR | 2 | 13.132 | 0,15 | 1,5 |
| Pontal do Paraná | PR | 4 | 27.284 | 0,15 | 0,0 |
| Tibagi | PR | 3 | 20.522 | 0,15 | 0,0 |
| Turvo | PR | 2 | 13.215 | 0,15 | 0,1 |
| Vitorino | PR | 1 | 6.838 | 0,15 | 0,0 |
| Águas Mornas | SC | 1 | 6.469 | 0,15 | 0,0 |
| Bombinhas | SC | 3 | 19.769 | 0,15 | 0,0 |
| Jaguaruna | SC | 3 | 20.024 | 0,15 | 0,0 |
| São Joaquim | SC | 4 | 26.952 | 0,15 | 2,9 |
| Arroio do Tigre | RS | 2 | 13.373 | 0,15 | 25,8 |
| Augusto Pestana | RS | 1 | 6.661 | 0,15 | 0,0 |
| Boa Vista do Buricá | RS | 1 | 6.712 | 0,15 | 0,0 |
| Camaquã | RS | 10 | 66.261 | 0,15 | 100,0 |
| Herval | RS | 1 | 6.821 | 0,15 | 99,4 |
| Formigueiro | RS | 1 | 6.664 | 0,15 | 0,6 |
| Morro Redondo | RS | 1 | 6.568 | 0,15 | 0,0 |
| Palmeira das Missões | RS | 5 | 33.303 | 0,15 | 0,0 |
| São José do Norte | RS | 4 | 27.568 | 0,15 | 18,0 |
| Campos de Júlio | MT | 1 | 6.891 | 0,15 | 7,6 |
| São José do Rio Claro | MT | 3 | 20.664 | 0,15 | 0,9 |
| Abadiânia | GO | 3 | 20.042 | 0,15 | 0,0 |
| Santa Bárbara de Goiás | GO | 1 | 6.560 | 0,15 | 0,0 |
| São Domingos | GO | 2 | 12.949 | 0,15 | 13,2 |
| Almas | TO | 1 | 7.055 | 0,14 | 7,8 |
| Campestre do Maranhão | MA | 2 | 14.374 | 0,14 | 0,9 |
| Olinda Nova do Maranhão | MA | 2 | 14.701 | 0,14 | 18,3 |
| Presidente Médici | MA | 1 | 7.015 | 0,14 | 94,6 |
| Campo Largo do Piauí | PI | 1 | 7.279 | 0,14 | 19,8 |
| Dirceu Arcoverde | PI | 1 | 7.011 | 0,14 | 33,6 |
| Joaquim Pires | PI | 2 | 14.354 | 0,14 | 0,1 |
| Nazaré do Piauí | PI | 1 | 7.339 | 0,14 | 18,6 |
| Simões | PI | 2 | 14.633 | 0,14 | 46,3 |
| Antonina do Norte | CE | 1 | 7.353 | 0,14 | 100,0 |
| Mucambo | CE | 2 | 14.537 | 0,14 | 0,0 |
| Orós | CE | 3 | 21.427 | 0,14 | 100,0 |
| Pedra Branca | CE | 6 | 43.258 | 0,14 | 100,0 |
| Alto do Rodrigues | RN | 2 | 14.529 | 0,14 | 100,0 |
| Bananeiras | PB | 3 | 21.318 | 0,14 | 27,3 |
| Boa Vista | PB | 1 | 7.051 | 0,14 | 26,6 |
| Gurinhém | PB | 2 | 14.129 | 0,14 | 0,1 |
| Queimadas | PB | 6 | 43.967 | 0,14 | 6,1 |
| Feira Nova | PE | 3 | 22.131 | 0,14 | 0,0 |
| Ipojuca | PE | 13 | 96.204 | 0,14 | 1,1 |
| Manari | PE | 3 | 21.434 | 0,14 | 15,8 |
| Petrolândia | PE | 5 | 36.548 | 0,14 | 5,0 |
| Terezinha | PE | 1 | 7.169 | 0,14 | 0,0 |
| Vitória de Santo Antão | PE | 19 | 138.757 | 0,14 | 0,2 |
| Campestre | AL | 1 | 6.936 | 0,14 | 18,9 |
| Chã Preta | AL | 1 | 7.311 | 0,14 | 0,0 |
| Limoeiro de Anadia | AL | 4 | 28.635 | 0,14 | 0,0 |
| Carira | SE | 3 | 22.082 | 0,14 | 3,0 |
| Macambira | SE | 1 | 6.919 | 0,14 | 0,0 |
| Baianópolis | BA | 2 | 13.877 | 0,14 | 3,5 |
| Barra do Mendes | BA | 2 | 13.865 | 0,14 | 11,2 |
| Bom Jesus da Lapa | BA | 10 | 69.148 | 0,14 | 4,4 |
| Carinhanha | BA | 4 | 29.018 | 0,14 | 10,3 |
| Cruz das Almas | BA | 9 | 63.239 | 0,14 | 0,0 |
| Ipecaetá | BA | 2 | 14.487 | 0,14 | 0,0 |
| Itaguaçu da Bahia | BA | 2 | 14.429 | 0,14 | 9,9 |
| Itatim | BA | 2 | 14.487 | 0,14 | 0,0 |
| Ituberá | BA | 4 | 28.602 | 0,14 | 2,1 |
| Nilo Peçanha | BA | 2 | 13.997 | 0,14 | 0,5 |
| Oliveira dos Brejinhos | BA | 3 | 21.825 | 0,14 | 9,0 |
| Ribeira do Amparo | BA | 2 | 14.592 | 0,14 | 0,1 |
| Uibaí | BA | 2 | 13.887 | 0,14 | 0,0 |
| Várzea da Roça | BA | 2 | 14.104 | 0,14 | 0,0 |
| Alterosa | MG | 2 | 14.466 | 0,14 | 3,0 |
| Engenheiro Navarro | MG | 1 | 7.242 | 0,14 | 2,7 |
| Governador Valadares | MG | 38 | 279.885 | 0,14 | 0,3 |
| Iraí de Minas | MG | 1 | 6.987 | 0,14 | 1,5 |
| Itapecerica | MG | 3 | 21.762 | 0,14 | 0,0 |
| Machacalis | MG | 1 | 7.111 | 0,14 | 0,0 |
| Machado | MG | 6 | 42.133 | 0,14 | 0,0 |
| Matias Barbosa | MG | 2 | 14.468 | 0,14 | 0,0 |
| Piumhi | MG | 5 | 34.691 | 0,14 | 0,0 |
| Santana da Vargem | MG | 1 | 7.100 | 0,14 | 0,0 |
| Santo Antônio do Retiro | MG | 1 | 7.277 | 0,14 | 13,1 |
| São Thomé das Letras | MG | 1 | 7.089 | 0,14 | 0,0 |
| Casimiro de Abreu | RJ | 6 | 44.184 | 0,14 | 0,0 |
| Santo Antônio de Pádua | RJ | 6 | 42.479 | 0,14 | 0,0 |
| São José de Ubá | RJ | 1 | 7.171 | 0,14 | 0,0 |
| Barbosa | SP | 1 | 7.402 | 0,14 | 0,0 |
| Capela do Alto | SP | 3 | 20.706 | 0,14 | 0,0 |
| Castilho | SP | 3 | 21.006 | 0,14 | 0,0 |
| Cosmorama | SP | 1 | 7.307 | 0,14 | 0,0 |
| Lucélia | SP | 3 | 21.747 | 0,14 | 0,0 |
| Santo Anastácio | SP | 3 | 20.878 | 0,14 | 0,0 |
| Santo Antônio da Alegria | SP | 1 | 6.929 | 0,14 | 0,0 |
| Altônia | PR | 3 | 22.056 | 0,14 | 0,0 |
| Bom Sucesso | PR | 1 | 7.032 | 0,14 | 0,0 |
| Campina da Lagoa | PR | 2 | 14.202 | 0,14 | 0,0 |
| Itaperuçu | PR | 4 | 28.634 | 0,14 | 0,9 |
| Ubiratã | PR | 3 | 21.013 | 0,14 | 0,0 |
| Água Doce | SC | 1 | 7.145 | 0,14 | 5,9 |
| Anita Garibaldi | SC | 1 | 7.133 | 0,14 | 2,2 |
| Campo Belo do Sul | SC | 1 | 7.016 | 0,14 | 0,0 |
| Maracajá | SC | 1 | 7.293 | 0,14 | 0,0 |
| Meleiro | SC | 1 | 7.015 | 0,14 | 0,0 |
| Porto União | SC | 5 | 35.398 | 0,14 | 0,0 |
| São José do Cedro | SC | 2 | 13.829 | 0,14 | 0,0 |
| Siderópolis | SC | 2 | 14.007 | 0,14 | 0,0 |
| Treze de Maio | SC | 1 | 7.081 | 0,14 | 0,0 |
| Iraí | RS | 1 | 7.241 | 0,14 | 0,0 |
| Manoel Viana | RS | 1 | 7.299 | 0,14 | 14,1 |
| Marau | RS | 6 | 44.161 | 0,14 | 0,0 |
| Santiago | RS | 7 | 49.425 | 0,14 | 100,0 |
| Jaraguari | MS | 1 | 7.187 | 0,14 | 12,1 |
| Alta Floresta | MT | 7 | 51.782 | 0,14 | 3,1 |
| Itarumã | GO | 1 | 7.178 | 0,14 | 9,8 |
| Uruana | GO | 2 | 13.843 | 0,14 | 0,0 |
| Boa Vista | RR | 50 | 399.213 | 0,13 | 0,2 |
| Canaã dos Carajás | PA | 5 | 37.085 | 0,13 | 0,7 |
| Anapurus | MA | 2 | 15.732 | 0,13 | 1,3 |
| Ribamar Fiquene | MA | 1 | 7.791 | 0,13 | 10,0 |
| São Vicente Ferrer | MA | 3 | 22.247 | 0,13 | 9,8 |
| Pedro II | PI | 5 | 38.742 | 0,13 | 4,5 |
| Altaneira | CE | 1 | 7.586 | 0,13 | 0,0 |
| Itaiçaba | CE | 1 | 7.827 | 0,13 | 0,3 |
| Senador Sá | CE | 1 | 7.623 | 0,13 | 4,5 |
| Cruzeta | RN | 1 | 7.998 | 0,13 | 0,6 |
| Jardim de Piranhas | RN | 2 | 14.837 | 0,13 | 4,5 |
| Portalegre | RN | 1 | 7.867 | 0,13 | 100,0 |
| São Miguel | RN | 3 | 23.519 | 0,13 | 100,0 |
| Coremas | PB | 2 | 15.445 | 0,13 | 100,0 |
| Cubati | PB | 1 | 7.797 | 0,13 | 99,3 |
| Jericó | PB | 1 | 7.739 | 0,13 | 83,3 |
| Princesa Isabel | PB | 3 | 23.345 | 0,13 | 0,3 |
| São Mamede | PB | 1 | 7.724 | 0,13 | 9,8 |
| Amaraji | PE | 3 | 22.829 | 0,13 | 9,6 |
| Belo Jardim | PE | 10 | 76.439 | 0,13 | 2,1 |
| Granito | PE | 1 | 7.486 | 0,13 | 34,9 |
| Primavera | PE | 2 | 14.966 | 0,13 | 1,3 |
| Rio Formoso | PE | 3 | 23.535 | 0,13 | 15,1 |
| Salgueiro | PE | 8 | 60.930 | 0,13 | 1,4 |
| Tamandaré | PE | 3 | 23.388 | 0,13 | 27,4 |
| Triunfo | PE | 2 | 15.254 | 0,13 | 0,0 |
| Ibateguara | AL | 2 | 15.616 | 0,13 | 33,2 |
| Passo de Camaragibe | AL | 2 | 15.245 | 0,13 | 0,5 |
| Santana do São Francisco | SE | 1 | 7.780 | 0,13 | 0,0 |
| Biritinga | BA | 2 | 15.979 | 0,13 | 3,0 |
| Brumado | BA | 9 | 67.195 | 0,13 | 3,5 |
| Glória | BA | 2 | 15.221 | 0,13 | 2,6 |
| Jussara | BA | 2 | 15.284 | 0,13 | 4,0 |
| Lagoa Real | BA | 2 | 15.665 | 0,13 | 1,3 |
| Ponto Novo | BA | 2 | 14.914 | 0,13 | 0,0 |
| Ruy Barbosa | BA | 4 | 30.814 | 0,13 | 7,2 |
| Salinas da Margarida | BA | 2 | 15.667 | 0,13 | 0,0 |
| São Gonçalo dos Campos | BA | 5 | 37.550 | 0,13 | 0,0 |
| Senhor do Bonfim | BA | 10 | 79.015 | 0,13 | 0,0 |
| Serra do Ramalho | BA | 4 | 31.472 | 0,13 | 0,3 |
| Serra Preta | BA | 2 | 14.878 | 0,13 | 10,7 |
| Xique-Xique | BA | 6 | 46.483 | 0,13 | 4,7 |
| Abaeté | MG | 3 | 23.237 | 0,13 | 1,0 |
| Araguari | MG | 15 | 117.267 | 0,13 | 0,6 |
| Botelhos | MG | 2 | 14.971 | 0,13 | 0,0 |
| Caeté | MG | 6 | 44.718 | 0,13 | 0,0 |
| Cruzília | MG | 2 | 15.417 | 0,13 | 0,0 |
| Florestal | MG | 1 | 7.461 | 0,13 | 0,0 |
| Formiga | MG | 9 | 67.683 | 0,13 | 0,4 |
| Itabirito | MG | 7 | 51.875 | 0,13 | 0,0 |
| Itapagipe | MG | 2 | 15.243 | 0,13 | 4,7 |
| Jacuí | MG | 1 | 7.686 | 0,13 | 0,0 |
| Lagamar | MG | 1 | 7.613 | 0,13 | 3,2 |
| Limeira do Oeste | MG | 1 | 7.536 | 0,13 | 12,0 |
| Mariana | MG | 8 | 60.724 | 0,13 | 0,0 |
| Mário Campos | MG | 2 | 15.416 | 0,13 | 0,0 |
| Minas Novas | MG | 4 | 31.484 | 0,13 | 31,4 |
| Miraí | MG | 2 | 15.014 | 0,13 | 0,0 |
| Monte Carmelo | MG | 6 | 47.809 | 0,13 | 6,7 |
| Montes Claros | MG | 52 | 409.341 | 0,13 | 0,1 |
| Monte Sião | MG | 3 | 23.803 | 0,13 | 0,0 |
| Nova Ponte | MG | 2 | 15.545 | 0,13 | 0,9 |
| Nova Porteirinha | MG | 1 | 7.500 | 0,13 | 0,0 |
| São João do Oriente | MG | 1 | 7.498 | 0,13 | 0,0 |
| São Pedro dos Ferros | MG | 1 | 7.781 | 0,13 | 2,0 |
| São Vicente de Minas | MG | 1 | 7.753 | 0,13 | 0,0 |
| Serrania | MG | 1 | 7.669 | 0,13 | 0,0 |
| Viçosa | MG | 10 | 78.846 | 0,13 | 0,0 |
| Ponto Belo | ES | 1 | 7.863 | 0,13 | 0,0 |
| Araçariguama | SP | 3 | 22.364 | 0,13 | 0,0 |
| Ibirarema | SP | 1 | 7.753 | 0,13 | 0,0 |
| Mairinque | SP | 6 | 47.150 | 0,13 | 0,0 |
| Ribeirão Grande | SP | 1 | 7.673 | 0,13 | 0,0 |
| São Pedro do Turvo | SP | 1 | 7.666 | 0,13 | 1,0 |
| Assaí | PR | 2 | 15.119 | 0,13 | 0,0 |
| Curiúva | PR | 2 | 15.101 | 0,13 | 0,0 |
| Figueira | PR | 1 | 7.770 | 0,13 | 0,0 |
| Marechal Cândido Rondon | PR | 7 | 52.944 | 0,13 | 0,0 |
| Curitibanos | SC | 5 | 39.745 | 0,13 | 0,0 |
| Guabiruba | SC | 3 | 23.832 | 0,13 | 0,0 |
| Herval d'Oeste | SC | 3 | 22.606 | 0,13 | 0,0 |
| Ipumirim | SC | 1 | 7.593 | 0,13 | 0,0 |
| Orleans | SC | 3 | 22.912 | 0,13 | 0,0 |
| Paulo Lopes | SC | 1 | 7.494 | 0,13 | 0,0 |
| Lavras do Sul | RS | 1 | 7.480 | 0,13 | 20,2 |
| Paraí | RS | 1 | 7.657 | 0,13 | 2,3 |
| Paraíso do Sul | RS | 1 | 7.611 | 0,13 | 0,0 |
| Rosário do Sul | RS | 5 | 39.422 | 0,13 | 8,0 |
| São Sepé | RS | 3 | 23.621 | 0,13 | 9,1 |
| Santa Rita do Pardo | MS | 1 | 7.851 | 0,13 | 37,5 |
| Nova Bandeirantes | MT | 2 | 15.288 | 0,13 | 22,0 |
| Caldas Novas | GO | 12 | 91.162 | 0,13 | 0,0 |
| Campo Limpo de Goiás | GO | 1 | 7.789 | 0,13 | 0,0 |
| Pires do Rio | GO | 4 | 31.458 | 0,13 | 0,2 |
| Planaltina | GO | 12 | 89.918 | 0,13 | 0,2 |
| Cerejeiras | RO | 2 | 16.323 | 0,12 | 100,0 |
| Ourilândia do Norte | PA | 4 | 32.832 | 0,12 | 10,8 |
| Alvorada | TO | 1 | 8.412 | 0,12 | 0,0 |
| Praia Norte | TO | 1 | 8.432 | 0,12 | 0,0 |
| Central do Maranhão | MA | 1 | 8.673 | 0,12 | 0,0 |
| Maranhãozinho | MA | 2 | 16.265 | 0,12 | 31,8 |
| São Domingos do Maranhão | MA | 4 | 34.376 | 0,12 | 0,6 |
| Floriano | PI | 7 | 59.935 | 0,12 | 3,0 |
| Luzilândia | PI | 3 | 25.486 | 0,12 | 3,1 |
| Nazária | PI | 1 | 8.570 | 0,12 | 0,0 |
| Brejo Santo | CE | 6 | 49.477 | 0,12 | 7,5 |
| Fortim | CE | 2 | 16.480 | 0,12 | 15,7 |
| Guaiúba | CE | 3 | 26.064 | 0,12 | 0,0 |
| Quixelô | CE | 2 | 16.178 | 0,12 | 100,0 |
| São Gonçalo do Amarante | CE | 6 | 48.422 | 0,12 | 1,7 |
| Tururu | CE | 2 | 16.271 | 0,12 | 49,4 |
| São Rafael | RN | 1 | 8.202 | 0,12 | 6,2 |
| Araçagi | PB | 2 | 16.987 | 0,12 | 19,7 |
| Esperança | PB | 4 | 33.007 | 0,12 | 11,8 |
| Marcação | PB | 1 | 8.558 | 0,12 | 31,1 |
| Pombal | PB | 4 | 32.801 | 0,12 | 17,7 |
| Rio Tinto | PB | 3 | 24.176 | 0,12 | 9,6 |
| Buíque | PE | 7 | 58.378 | 0,12 | 1,1 |
| Machados | PE | 2 | 16.088 | 0,12 | 5,8 |
| Quipapá | PE | 3 | 26.037 | 0,12 | 12,5 |
| Capela | AL | 2 | 17.053 | 0,12 | 0,5 |
| Pão de Açúcar | AL | 3 | 24.399 | 0,12 | 0,0 |
| São José da Tapera | AL | 4 | 32.260 | 0,12 | 0,0 |
| Maruim | SE | 2 | 17.213 | 0,12 | 0,0 |
| Simão Dias | SE | 5 | 40.484 | 0,12 | 0,0 |
| Canudos | BA | 2 | 16.668 | 0,12 | 14,7 |
| Cícero Dantas | BA | 4 | 32.515 | 0,12 | 0,1 |
| Conceição do Almeida | BA | 2 | 17.247 | 0,12 | 0,0 |
| Condeúba | BA | 2 | 17.247 | 0,12 | 2,5 |
| Correntina | BA | 4 | 32.137 | 0,12 | 16,0 |
| Elísio Medrado | BA | 1 | 8.122 | 0,12 | 0,0 |
| Ibititá | BA | 2 | 17.113 | 0,12 | 0,0 |
| Itaeté | BA | 2 | 16.056 | 0,12 | 0,1 |
| Jeremoabo | BA | 5 | 40.463 | 0,12 | 4,5 |
| Lamarão | BA | 1 | 8.307 | 0,12 | 0,0 |
| Morpará | BA | 1 | 8.519 | 0,12 | 25,0 |
| Sento Sé | BA | 5 | 40.684 | 0,12 | 14,5 |
| Serra Dourada | BA | 2 | 17.386 | 0,12 | 4,4 |
| Urandi | BA | 2 | 16.658 | 0,12 | 0,1 |
| Barão de Cocais | MG | 4 | 32.485 | 0,12 | 0,0 |
| Brasília de Minas | MG | 4 | 32.347 | 0,12 | 1,6 |
| Monsenhor Paulo | MG | 1 | 8.688 | 0,12 | 0,0 |
| Nazareno | MG | 1 | 8.608 | 0,12 | 0,0 |
| Passa Tempo | MG | 1 | 8.084 | 0,12 | 0,0 |
| Pimenta | MG | 1 | 8.660 | 0,12 | 0,0 |
| Poté | MG | 2 | 16.555 | 0,12 | 1,9 |
| Riachinho | MG | 1 | 8.136 | 0,12 | 13,0 |
| Santana do Paraíso | MG | 4 | 34.663 | 0,12 | 0,0 |
| Taiobeiras | MG | 4 | 34.132 | 0,12 | 1,5 |
| Uberaba | MG | 40 | 333.783 | 0,12 | 0,0 |
| Urucuia | MG | 2 | 16.865 | 0,12 | 3,6 |
| Armação dos Búzios | RJ | 4 | 33.870 | 0,12 | 0,0 |
| Comendador Levy Gasparian | RJ | 1 | 8.561 | 0,12 | 0,0 |
| Petrópolis | RJ | 36 | 306.191 | 0,12 | 0,0 |
| Águas da Prata | SP | 1 | 8.180 | 0,12 | 0,0 |
| Arealva | SP | 1 | 8.560 | 0,12 | 0,0 |
| Cabreúva | SP | 6 | 49.707 | 0,12 | 0,0 |
| Caraguatatuba | SP | 14 | 121.532 | 0,12 | 0,0 |
| Clementina | SP | 1 | 8.617 | 0,12 | 0,0 |
| Cristais Paulista | SP | 1 | 8.631 | 0,12 | 0,0 |
| Descalvado | SP | 4 | 33.718 | 0,12 | 0,0 |
| Estrela d'Oeste | SP | 1 | 8.419 | 0,12 | 0,0 |
| Macatuba | SP | 2 | 17.163 | 0,12 | 0,0 |
| Monte Aprazível | SP | 3 | 25.087 | 0,12 | 0,0 |
| Potim | SP | 3 | 24.643 | 0,12 | 0,0 |
| Potirendaba | SP | 2 | 17.361 | 0,12 | 0,0 |
| Rosana | SP | 2 | 16.643 | 0,12 | 1,0 |
| Santa Cruz das Palmeiras | SP | 4 | 34.361 | 0,12 | 0,0 |
| Santo Antônio do Aracanguá | SP | 1 | 8.420 | 0,12 | 0,0 |
| Tabatinga | SP | 2 | 16.496 | 0,12 | 0,0 |
| Califórnia | PR | 1 | 8.570 | 0,12 | 0,0 |
| Clevelândia | PR | 2 | 16.559 | 0,12 | 0,0 |
| Imbituva | PR | 4 | 32.564 | 0,12 | 0,0 |
| Jesuítas | PR | 1 | 8.412 | 0,12 | 0,0 |
| Lapa | PR | 6 | 48.163 | 0,12 | 0,1 |
| Mangueirinha | PR | 2 | 16.714 | 0,12 | 0,6 |
| Santa Isabel do Ivaí | PR | 1 | 8.562 | 0,12 | 0,0 |
| Vera Cruz do Oeste | PR | 1 | 8.521 | 0,12 | 0,3 |
| Monte Castelo | SC | 1 | 8.275 | 0,12 | 0,5 |
| Santa Cecília | SC | 2 | 16.830 | 0,12 | 0,0 |
| São Miguel do Oeste | SC | 5 | 40.482 | 0,12 | 0,0 |
| Tangará | SC | 1 | 8.676 | 0,12 | 0,0 |
| Glorinha | RS | 1 | 8.098 | 0,12 | 0,0 |
| Minas do Leão | RS | 1 | 8.075 | 0,12 | 1,1 |
| São Pedro do Sul | RS | 2 | 16.198 | 0,12 | 0,4 |
| Sarandi | RS | 3 | 24.489 | 0,12 | 0,0 |
| Tapes | RS | 2 | 17.300 | 0,12 | 99,8 |
| Teutônia | RS | 4 | 33.232 | 0,12 | 0,0 |
| Bela Vista | MS | 3 | 24.629 | 0,12 | 8,4 |
| Chapadão do Sul | MS | 3 | 25.218 | 0,12 | 3,1 |
| Dom Aquino | MT | 1 | 8.178 | 0,12 | 99,5 |
| São Luís de Montes Belos | GO | 4 | 33.817 | 0,12 | 0,0 |
| Ouro Preto do Oeste | RO | 4 | 36.035 | 0,11 | 100,0 |
| Barreirinhas | MA | 7 | 62.528 | 0,11 | 8,6 |
| Cachoeira Grande | MA | 1 | 9.431 | 0,11 | 25,6 |
| Icatu | MA | 3 | 27.113 | 0,11 | 29,9 |
| Santa Rita | MA | 4 | 37.855 | 0,11 | 1,8 |
| São Bernardo | MA | 3 | 28.507 | 0,11 | 0,0 |
| Turiaçu | MA | 4 | 35.604 | 0,11 | 35,5 |
| Água Branca | PI | 2 | 17.411 | 0,11 | 0,0 |
| Pio IX | PI | 2 | 18.425 | 0,11 | 82,3 |
| Queimada Nova | PI | 1 | 8.992 | 0,11 | 30,1 |
| São Miguel do Tapuio | PI | 2 | 17.662 | 0,11 | 17,8 |
| Crato | CE | 15 | 132.123 | 0,11 | 0,3 |
| Missão Velha | CE | 4 | 35.393 | 0,11 | 1,8 |
| Santa Quitéria | CE | 5 | 43.703 | 0,11 | 27,1 |
| Baraúna | RN | 3 | 28.374 | 0,11 | 0,1 |
| Ceará-Mirim | RN | 8 | 73.497 | 0,11 | 0,0 |
| Jaçanã | RN | 1 | 9.133 | 0,11 | 1,4 |
| Nova Cruz | RN | 4 | 37.343 | 0,11 | 7,1 |
| São João do Rio do Peixe | PB | 2 | 18.034 | 0,11 | 100,0 |
| Água Preta | PE | 4 | 36.771 | 0,11 | 31,5 |
| Brejão | PE | 1 | 8.993 | 0,11 | 0,0 |
| Gravatá | PE | 9 | 84.074 | 0,11 | 0,3 |
| Lagoa do Carro | PE | 2 | 18.071 | 0,11 | 0,0 |
| Paudalho | PE | 6 | 56.506 | 0,11 | 0,0 |
| Tupanatinga | PE | 3 | 27.304 | 0,11 | 5,0 |
| Campo Alegre | AL | 6 | 57.063 | 0,11 | 0,0 |
| Carneiros | AL | 1 | 9.081 | 0,11 | 0,0 |
| Coruripe | AL | 6 | 56.933 | 0,11 | 0,0 |
| Maravilha | AL | 1 | 9.163 | 0,11 | 1,5 |
| Olho d'Água do Casado | AL | 1 | 9.373 | 0,11 | 0,0 |
| Nossa Senhora das Dores | SE | 3 | 26.629 | 0,11 | 0,1 |
| Pirambu | SE | 1 | 9.280 | 0,11 | 0,0 |
| Alagoinhas | BA | 17 | 151.596 | 0,11 | 0,0 |
| Araci | BA | 6 | 54.379 | 0,11 | 0,0 |
| Boa Vista do Tupim | BA | 2 | 18.576 | 0,11 | 6,1 |
| Casa Nova | BA | 8 | 71.969 | 0,11 | 23,0 |
| Cocos | BA | 2 | 18.777 | 0,11 | 12,4 |
| Guanambi | BA | 9 | 84.481 | 0,11 | 0,0 |
| Ibipeba | BA | 2 | 18.211 | 0,11 | 0,9 |
| Ilhéus | BA | 18 | 162.327 | 0,11 | 0,5 |
| Lapão | BA | 3 | 27.223 | 0,11 | 0,0 |
| Livramento de Nossa Senhora | BA | 5 | 45.732 | 0,11 | 0,4 |
| Riacho de Santana | BA | 4 | 35.421 | 0,11 | 15,2 |
| Santanópolis | BA | 1 | 8.943 | 0,11 | 0,0 |
| Santo Estêvão | BA | 6 | 52.850 | 0,11 | 0,0 |
| Teixeira de Freitas | BA | 17 | 160.487 | 0,11 | 0,1 |
| Valente | BA | 3 | 28.473 | 0,11 | 0,0 |
| Além Paraíba | MG | 4 | 35.362 | 0,11 | 0,0 |
| Antônio Dias | MG | 1 | 9.318 | 0,11 | 0,6 |
| Coqueiral | MG | 1 | 9.159 | 0,11 | 0,0 |
| Cordisburgo | MG | 1 | 8.890 | 0,11 | 10,6 |
| Itacarambi | MG | 2 | 18.153 | 0,11 | 4,5 |
| Rio Novo | MG | 1 | 8.949 | 0,11 | 0,0 |
| São João Nepomuceno | MG | 3 | 26.361 | 0,11 | 0,0 |
| Varginha | MG | 15 | 135.558 | 0,11 | 0,0 |
| Magé | RJ | 27 | 245.071 | 0,11 | 0,0 |
| Mendes | RJ | 2 | 18.614 | 0,11 | 0,0 |
| Miracema | RJ | 3 | 27.174 | 0,11 | 0,0 |
| Paracambi | RJ | 6 | 52.257 | 0,11 | 0,0 |
| Agudos | SP | 4 | 37.214 | 0,11 | 0,2 |
| Alumínio | SP | 2 | 18.628 | 0,11 | 0,0 |
| Cajuru | SP | 3 | 26.167 | 0,11 | 0,0 |
| Cedral | SP | 1 | 9.237 | 0,11 | 0,0 |
| Cesário Lange | SP | 2 | 18.148 | 0,11 | 0,0 |
| Conchal | SP | 3 | 28.050 | 0,11 | 0,0 |
| Dobrada | SP | 1 | 8.929 | 0,11 | 0,0 |
| Guaratinguetá | SP | 13 | 121.798 | 0,11 | 0,0 |
| Guareí | SP | 2 | 18.520 | 0,11 | 0,6 |
| Itirapina | SP | 2 | 18.157 | 0,11 | 0,0 |
| José Bonifácio | SP | 4 | 37.015 | 0,11 | 0,0 |
| Mirante do Paranapanema | SP | 2 | 18.259 | 0,11 | 5,0 |
| Neves Paulista | SP | 1 | 8.930 | 0,11 | 0,0 |
| Palmeira d'Oeste | SP | 1 | 9.283 | 0,11 | 0,0 |
| Paulo de Faria | SP | 1 | 8.945 | 0,11 | 0,1 |
| Pederneiras | SP | 5 | 46.687 | 0,11 | 0,0 |
| Porto Feliz | SP | 6 | 53.098 | 0,11 | 0,0 |
| Santa Rosa de Viterbo | SP | 3 | 26.540 | 0,11 | 0,0 |
| São José do Rio Pardo | SP | 6 | 54.946 | 0,11 | 0,0 |
| Urânia | SP | 1 | 9.114 | 0,11 | 0,0 |
| Vista Alegre do Alto | SP | 1 | 8.810 | 0,11 | 0,0 |
| Agudos do Sul | PR | 1 | 9.371 | 0,11 | 0,0 |
| Castro | PR | 8 | 71.484 | 0,11 | 0,0 |
| Congonhinhas | PR | 1 | 8.818 | 0,11 | 0,5 |
| São Jorge d'Oeste | PR | 1 | 9.050 | 0,11 | 0,0 |
| São Sebastião da Amoreira | PR | 1 | 8.853 | 0,11 | 0,0 |
| Terra Roxa | PR | 2 | 17.481 | 0,11 | 0,0 |
| Fraiburgo | SC | 4 | 36.443 | 0,11 | 0,0 |
| Ibirama | SC | 2 | 18.950 | 0,11 | 0,0 |
| Seara | SC | 2 | 17.541 | 0,11 | 0,0 |
| Osório | RS | 5 | 45.994 | 0,11 | 0,0 |
| Portão | RS | 4 | 37.079 | 0,11 | 0,0 |
| Vacaria | RS | 7 | 66.218 | 0,11 | 41,6 |
| Jardim | MS | 3 | 26.097 | 0,11 | 2,0 |
| Mundo Novo | MS | 2 | 18.366 | 0,11 | 0,1 |
| Castanheira | MT | 1 | 8.729 | 0,11 | 13,0 |
| Denise | MT | 1 | 9.462 | 0,11 | 3,2 |
| Várzea Grande | MT | 30 | 284.971 | 0,11 | 0,2 |
| Nova Monte Verde | MT | 1 | 9.178 | 0,11 | 3,9 |
| Abadia de Goiás | GO | 1 | 8.773 | 0,11 | 0,0 |
| Luziânia | GO | 22 | 208.299 | 0,11 | 0,3 |
| Marituba | PA | 13 | 131.521 | 0,1 | 0,0 |
| Jatobá | MA | 1 | 10.153 | 0,1 | 0,1 |
| Santa Inês | MA | 9 | 89.044 | 0,1 | 1,8 |
| Serrano do Maranhão | MA | 1 | 10.253 | 0,1 | 26,6 |
| Guadalupe | PI | 1 | 10.499 | 0,1 | 3,3 |
| Valença do Piauí | PI | 2 | 20.918 | 0,1 | 0,5 |
| Ipu | CE | 4 | 41.964 | 0,1 | 35,3 |
| Jijoca de Jericoacoara | CE | 2 | 19.816 | 0,1 | 100,0 |
| Augusto Severo | RN | 1 | 9.655 | 0,1 | 30,7 |
| Januário Cicco | RN | 1 | 10.181 | 0,1 | 1,6 |
| Macaíba | RN | 8 | 80.792 | 0,1 | 0,0 |
| Alhandra | PB | 2 | 19.588 | 0,1 | 0,0 |
| Bom Jardim | PE | 4 | 39.184 | 0,1 | 19,3 |
| Bonito | PE | 4 | 38.134 | 0,1 | 2,9 |
| Glória do Goitá | PE | 3 | 30.604 | 0,1 | 1,6 |
| Sairé | PE | 1 | 9.932 | 0,1 | 0,0 |
| São Lourenço da Mata | PE | 11 | 113.230 | 0,1 | 0,0 |
| Taquaritinga do Norte | PE | 3 | 28.775 | 0,1 | 6,7 |
| Girau do Ponciano | AL | 4 | 40.917 | 0,1 | 0,0 |
| Arauá | SE | 1 | 10.056 | 0,1 | 0,0 |
| Itabaianinha | SE | 4 | 41.928 | 0,1 | 0,0 |
| Laranjeiras | SE | 3 | 29.826 | 0,1 | 0,0 |
| São Cristóvão | SE | 9 | 90.072 | 0,1 | 0,0 |
| Baixa Grande | BA | 2 | 20.468 | 0,1 | 0,7 |
| Campo Alegre de Lourdes | BA | 3 | 28.798 | 0,1 | 4,0 |
| Irecê | BA | 7 | 72.967 | 0,1 | 0,0 |
| Pintadas | BA | 1 | 10.437 | 0,1 | 2,7 |
| Santa Bárbara | BA | 2 | 20.791 | 0,1 | 0,0 |
| Águas Formosas | MG | 2 | 19.207 | 0,1 | 0,0 |
| Alfenas | MG | 8 | 79.996 | 0,1 | 0,0 |
| Arcos | MG | 4 | 40.092 | 0,1 | 0,0 |
| Barbacena | MG | 14 | 137.313 | 0,1 | 0,0 |
| Bela Vista de Minas | MG | 1 | 10.255 | 0,1 | 0,0 |
| Bom Despacho | MG | 5 | 50.605 | 0,1 | 0,0 |
| Cristina | MG | 1 | 10.242 | 0,1 | 0,0 |
| Curvelo | MG | 8 | 80.129 | 0,1 | 0,4 |
| Diamantina | MG | 5 | 47.723 | 0,1 | 2,9 |
| Francisco Badaró | MG | 1 | 10.332 | 0,1 | 0,2 |
| Frei Inocêncio | MG | 1 | 9.611 | 0,1 | 7,0 |
| Ipuiúna | MG | 1 | 10.079 | 0,1 | 3,0 |
| Itabira | MG | 12 | 120.060 | 0,1 | 0,0 |
| Lambari | MG | 2 | 20.814 | 0,1 | 0,0 |
| Mateus Leme | MG | 3 | 31.086 | 0,1 | 0,0 |
| Ubá | MG | 12 | 115.552 | 0,1 | 0,0 |
| Varzelândia | MG | 2 | 19.320 | 0,1 | 5,0 |
| Vazante | MG | 2 | 20.590 | 0,1 | 0,6 |
| Conceição da Barra | ES | 3 | 31.063 | 0,1 | 0,0 |
| Arraial do Cabo | RJ | 3 | 30.349 | 0,1 | 0,0 |
| Guapimirim | RJ | 6 | 60.517 | 0,1 | 0,0 |
| Rio Bonito | RJ | 6 | 60.201 | 0,1 | 0,0 |
| Ariranha | SP | 1 | 9.668 | 0,1 | 0,0 |
| Botucatu | SP | 14 | 146.497 | 0,1 | 0,0 |
| Buri | SP | 2 | 19.878 | 0,1 | 0,0 |
| Cerqueira César | SP | 2 | 19.985 | 0,1 | 0,0 |
| Dumont | SP | 1 | 9.868 | 0,1 | 0,0 |
| Paranapanema | SP | 2 | 20.197 | 0,1 | 0,3 |
| Porangaba | SP | 1 | 9.925 | 0,1 | 0,0 |
| Reginópolis | SP | 1 | 9.621 | 0,1 | 0,0 |
| Ampére | PR | 2 | 19.152 | 0,1 | 0,0 |
| Cruzeiro do Oeste | PR | 2 | 20.932 | 0,1 | 0,0 |
| Ibaiti | PR | 3 | 31.364 | 0,1 | 0,0 |
| Mariluz | PR | 1 | 10.345 | 0,1 | 0,0 |
| Nova Aurora | PR | 1 | 10.472 | 0,1 | 0,0 |
| Rondon | PR | 1 | 9.579 | 0,1 | 0,0 |
| Santa Maria do Oeste | PR | 1 | 9.615 | 0,1 | 2,8 |
| Pinhalzinho | SC | 2 | 20.313 | 0,1 | 0,0 |
| Sombrio | SC | 3 | 30.374 | 0,1 | 0,0 |
| Arroio do Sal | RS | 1 | 10.065 | 0,1 | 0,0 |
| Arvorezinha | RS | 1 | 10.424 | 0,1 | 6,0 |
| Candiota | RS | 1 | 9.584 | 0,1 | 1,4 |
| Fontoura Xavier | RS | 1 | 10.304 | 0,1 | 10,6 |
| Frederico Westphalen | RS | 3 | 31.313 | 0,1 | 0,0 |
| Júlio de Castilhos | RS | 2 | 19.293 | 0,1 | 0,9 |
| Nova Santa Rita | RS | 3 | 29.300 | 0,1 | 0,0 |
| Porto Xavier | RS | 1 | 10.246 | 0,1 | 0,1 |
| Santa Vitória do Palmar | RS | 3 | 29.676 | 0,1 | 99,9 |
| Soledade | RS | 3 | 31.002 | 0,1 | 2,0 |
| Ponta Porã | MS | 9 | 92.526 | 0,1 | 4,5 |
| Arenápolis | MT | 1 | 9.607 | 0,1 | 0,6 |
| Aragarças | GO | 2 | 20.118 | 0,1 | 0,0 |
| Aruanã | GO | 1 | 9.875 | 0,1 | 1,3 |
| Carmo do Rio Verde | GO | 1 | 10.082 | 0,1 | 0,0 |
| Iporá | GO | 3 | 31.531 | 0,1 | 0,0 |
| Itumbiara | GO | 10 | 104.742 | 0,1 | 0,2 |
| Nova Veneza | GO | 1 | 9.853 | 0,1 | 0,0 |
| São Simão | GO | 2 | 20.645 | 0,1 | 0,0 |
| Cururupu | MA | 3 | 32.695 | 0,09 | 16,6 |
| Imperatriz | MA | 22 | 258.682 | 0,09 | 0,0 |
| Maracaçumé | MA | 2 | 21.395 | 0,09 | 14,3 |
| Paraibano | MA | 2 | 21.386 | 0,09 | 99,2 |
| Santa Helena | MA | 4 | 42.130 | 0,09 | 30,0 |
| Sucupira do Norte | MA | 1 | 10.636 | 0,09 | 99,6 |
| Avelino Lopes | PI | 1 | 11.289 | 0,09 | 100,0 |
| Cabeceiras do Piauí | PI | 1 | 10.586 | 0,09 | 0,0 |
| Fronteiras | PI | 1 | 11.625 | 0,09 | 27,4 |
| Gilbués | PI | 1 | 10.690 | 0,09 | 98,2 |
| Itaueira | PI | 1 | 11.019 | 0,09 | 36,8 |
| Miguel Alves | PI | 3 | 33.760 | 0,09 | 6,1 |
| Monte Alegre do Piauí | PI | 1 | 10.613 | 0,09 | 92,9 |
| Picos | PI | 7 | 78.222 | 0,09 | 0,0 |
| Araripe | CE | 2 | 21.600 | 0,09 | 83,6 |
| Pires Ferreira | CE | 1 | 10.951 | 0,09 | 19,1 |
| Quiterianópolis | CE | 2 | 21.084 | 0,09 | 95,6 |
| Uruburetama | CE | 2 | 21.850 | 0,09 | 86,2 |
| Acari | RN | 1 | 11.136 | 0,09 | 1,8 |
| Canguaretama | RN | 3 | 34.276 | 0,09 | 0,2 |
| Macau | RN | 3 | 31.814 | 0,09 | 8,7 |
| Monte Alegre | RN | 2 | 22.451 | 0,09 | 0,0 |
| Mossoró | RN | 27 | 297.378 | 0,09 | 0,0 |
| Juripiranga | PB | 1 | 10.756 | 0,09 | 0,0 |
| Mamanguape | PB | 4 | 44.882 | 0,09 | 4,3 |
| Pirpirituba | PB | 1 | 10.579 | 0,09 | 0,5 |
| São Vicente do Seridó | PB | 1 | 10.775 | 0,09 | 100,0 |
| Abreu e Lima | PE | 9 | 99.990 | 0,09 | 0,0 |
| Angelim | PE | 1 | 11.150 | 0,09 | 14,3 |
| Barreiros | PE | 4 | 42.659 | 0,09 | 62,4 |
| Camaragibe | PE | 14 | 157.828 | 0,09 | 0,0 |
| Catende | PE | 4 | 42.892 | 0,09 | 4,1 |
| Chã Grande | PE | 2 | 21.698 | 0,09 | 2,2 |
| Inajá | PE | 2 | 23.247 | 0,09 | 31,7 |
| Parnamirim | PE | 2 | 22.010 | 0,09 | 36,7 |
| Cacimbinhas | AL | 1 | 10.858 | 0,09 | 0,0 |
| Colônia Leopoldina | AL | 2 | 21.698 | 0,09 | 84,9 |
| Olho d'Água das Flores | AL | 2 | 21.688 | 0,09 | 0,0 |
| Pariconha | AL | 1 | 10.533 | 0,09 | 0,0 |
| Rio Largo | AL | 7 | 75.120 | 0,09 | 0,0 |
| São Sebastião | AL | 3 | 34.152 | 0,09 | 0,0 |
| Capela | SE | 3 | 34.213 | 0,09 | 0,0 |
| Rosário do Catete | SE | 1 | 10.855 | 0,09 | 0,0 |
| São Domingos | SE | 1 | 11.137 | 0,09 | 0,0 |
| Chorrochó | BA | 1 | 11.177 | 0,09 | 15,7 |
| Coração de Maria | BA | 2 | 22.605 | 0,09 | 0,0 |
| Itaparica | BA | 2 | 22.228 | 0,09 | 0,0 |
| Luís Eduardo Magalhães | BA | 8 | 87.519 | 0,09 | 0,0 |
| Teofilândia | BA | 2 | 22.518 | 0,09 | 0,9 |
| Bueno Brandão | MG | 1 | 11.001 | 0,09 | 0,0 |
| Carmo do Cajuru | MG | 2 | 22.478 | 0,09 | 0,0 |
| Delta | MG | 1 | 10.533 | 0,09 | 0,0 |
| Divisópolis | MG | 1 | 11.019 | 0,09 | 1,0 |
| Esmeraldas | MG | 6 | 70.552 | 0,09 | 0,0 |
| Igarapé | MG | 4 | 43.045 | 0,09 | 0,0 |
| Itatiaiuçu | MG | 1 | 11.146 | 0,09 | 0,0 |
| Pedralva | MG | 1 | 11.195 | 0,09 | 0,0 |
| Perdigão | MG | 1 | 11.506 | 0,09 | 0,0 |
| Pirapetinga | MG | 1 | 10.752 | 0,09 | 0,0 |
| Piraúba | MG | 1 | 10.787 | 0,09 | 0,0 |
| Porto Firme | MG | 1 | 11.279 | 0,09 | 0,0 |
| Resende Costa | MG | 1 | 11.500 | 0,09 | 0,0 |
| Timóteo | MG | 8 | 89.842 | 0,09 | 0,0 |
| Fundão | ES | 2 | 21.509 | 0,09 | 0,0 |
| Presidente Kennedy | ES | 1 | 11.574 | 0,09 | 0,0 |
| Aperibé | RJ | 1 | 11.759 | 0,09 | 0,0 |
| Duas Barras | RJ | 1 | 11.492 | 0,09 | 0,0 |
| Itatiaia | RJ | 3 | 31.805 | 0,09 | 0,0 |
| Paraíba do Sul | RJ | 4 | 44.285 | 0,09 | 0,0 |
| Saquarema | RJ | 8 | 89.170 | 0,09 | 0,0 |
| Areiópolis | SP | 1 | 11.129 | 0,09 | 0,0 |
| Bernardino de Campos | SP | 1 | 11.148 | 0,09 | 0,0 |
| Cajobi | SP | 1 | 10.542 | 0,09 | 0,0 |
| Guapiaçu | SP | 2 | 21.454 | 0,09 | 0,0 |
| Iacanga | SP | 1 | 11.710 | 0,09 | 0,0 |
| Jardinópolis | SP | 4 | 44.380 | 0,09 | 0,0 |
| Mococa | SP | 6 | 68.885 | 0,09 | 0,0 |
| Penápolis | SP | 6 | 63.407 | 0,09 | 0,0 |
| Porto Ferreira | SP | 5 | 56.150 | 0,09 | 0,0 |
| Roseira | SP | 1 | 10.712 | 0,09 | 0,0 |
| Vargem | SP | 1 | 10.537 | 0,09 | 0,0 |
| Estiva Gerbi | SP | 1 | 11.304 | 0,09 | 0,0 |
| Alvorada do Sul | PR | 1 | 11.406 | 0,09 | 2,6 |
| Assis Chateaubriand | PR | 3 | 33.362 | 0,09 | 0,0 |
| Campina Grande do Sul | PR | 4 | 43.288 | 0,09 | 0,1 |
| Centenário do Sul | PR | 1 | 10.827 | 0,09 | 0,4 |
| Laranjeiras do Sul | PR | 3 | 32.073 | 0,09 | 0,0 |
| Loanda | PR | 2 | 23.086 | 0,09 | 0,0 |
| Nova Prata do Iguaçu | PR | 1 | 10.548 | 0,09 | 0,0 |
| Paranaguá | PR | 14 | 154.936 | 0,09 | 0,5 |
| Uraí | PR | 1 | 11.314 | 0,09 | 0,0 |
| Agrolândia | SC | 1 | 10.864 | 0,09 | 0,0 |
| Balneário Gaivota | SC | 1 | 10.979 | 0,09 | 0,0 |
| Brusque | SC | 12 | 134.723 | 0,09 | 0,0 |
| Catanduvas | SC | 1 | 10.861 | 0,09 | 0,7 |
| Concórdia | SC | 7 | 74.641 | 0,09 | 0,0 |
| Irineópolis | SC | 1 | 11.222 | 0,09 | 0,0 |
| Itapema | SC | 6 | 65.312 | 0,09 | 0,0 |
| Santo Amaro da Imperatriz | SC | 2 | 23.245 | 0,09 | 0,0 |
| São Carlos | SC | 1 | 11.281 | 0,09 | 0,0 |
| Urubici | SC | 1 | 11.235 | 0,09 | 0,6 |
| Bom Jesus | RS | 1 | 11.349 | 0,09 | 7,3 |
| Palmares do Sul | RS | 1 | 11.318 | 0,09 | 0,1 |
| Panambi | RS | 4 | 43.667 | 0,09 | 0,0 |
| Roca Sales | RS | 1 | 11.393 | 0,09 | 0,0 |
| Santo Antônio da Patrulha | RS | 4 | 42.894 | 0,09 | 0,0 |
| Bataguassu | MS | 2 | 23.024 | 0,09 | 5,0 |
| Batayporã | MS | 1 | 11.329 | 0,09 | 0,1 |
| Ivinhema | MS | 2 | 23.187 | 0,09 | 0,0 |
| Naviraí | MS | 5 | 54.878 | 0,09 | 0,0 |
| Nova Andradina | MS | 5 | 54.374 | 0,09 | 2,5 |
| Sete Quedas | MS | 1 | 10.791 | 0,09 | 0,2 |
| Três Lagoas | MS | 11 | 121.388 | 0,09 | 1,2 |
| Anápolis | GO | 36 | 386.923 | 0,09 | 0,0 |
| Catalão | GO | 10 | 108.823 | 0,09 | 0,5 |
| Inhumas | GO | 5 | 52.866 | 0,09 | 0,0 |
| Seringueiras | RO | 1 | 11.856 | 0,08 | 37,0 |
| Benevides | PA | 5 | 62.737 | 0,08 | 0,0 |
| Davinópolis | MA | 1 | 12.908 | 0,08 | 0,0 |
| Godofredo Viana | MA | 1 | 11.819 | 0,08 | 100,0 |
| João Lisboa | MA | 2 | 23.632 | 0,08 | 2,1 |
| Loreto | MA | 1 | 12.157 | 0,08 | 19,6 |
| Presidente Juscelino | MA | 1 | 12.734 | 0,08 | 6,6 |
| São Francisco do Brejão | MA | 1 | 11.798 | 0,08 | 0,7 |
| Simplício Mendes | PI | 1 | 12.711 | 0,08 | 13,7 |
| Aracoiaba | CE | 2 | 26.469 | 0,08 | 1,7 |
| Chaval | CE | 1 | 13.069 | 0,08 | 0,5 |
| Jucás | CE | 2 | 24.833 | 0,08 | 100,0 |
| Paramoti | CE | 1 | 12.226 | 0,08 | 47,4 |
| Brejinho | RN | 1 | 12.699 | 0,08 | 0,0 |
| Passa e Fica | RN | 1 | 13.277 | 0,08 | 0,0 |
| Imaculada | PB | 1 | 11.819 | 0,08 | 5,5 |
| Mogeiro | PB | 1 | 13.284 | 0,08 | 0,0 |
| Paulista | PB | 1 | 12.347 | 0,08 | 8,0 |
| Agrestina | PE | 2 | 24.885 | 0,08 | 0,0 |
| Aliança | PE | 3 | 38.386 | 0,08 | 0,0 |
| Cabo de Santo Agostinho | PE | 16 | 207.048 | 0,08 | 0,1 |
| Canhotinho | PE | 2 | 24.804 | 0,08 | 19,6 |
| Caruaru | PE | 30 | 361.118 | 0,08 | 0,0 |
| Cedro | PE | 1 | 11.807 | 0,08 | 1,2 |
| Condado | PE | 2 | 26.421 | 0,08 | 0,0 |
| Cortês | PE | 1 | 12.578 | 0,08 | 9,9 |
| Lagoa Grande | PE | 2 | 25.601 | 0,08 | 10,2 |
| Macaparana | PE | 2 | 25.376 | 0,08 | 0,0 |
| Orobó | PE | 2 | 23.884 | 0,08 | 27,5 |
| Petrolina | PE | 28 | 349.145 | 0,08 | 2,0 |
| Santa Terezinha | PE | 1 | 11.815 | 0,08 | 9,2 |
| Tacaimbó | PE | 1 | 12.874 | 0,08 | 0,0 |
| Tacaratu | PE | 2 | 25.765 | 0,08 | 0,3 |
| Timbaúba | PE | 4 | 53.022 | 0,08 | 0,0 |
| Craíbas | AL | 2 | 24.219 | 0,08 | 0,0 |
| Delmiro Gouveia | AL | 4 | 52.016 | 0,08 | 0,0 |
| Igaci | AL | 2 | 25.631 | 0,08 | 0,0 |
| Junqueiro | AL | 2 | 24.727 | 0,08 | 0,0 |
| Santana do Ipanema | AL | 4 | 47.654 | 0,08 | 0,0 |
| Viçosa | AL | 2 | 25.733 | 0,08 | 0,4 |
| Lagarto | SE | 8 | 104.408 | 0,08 | 0,0 |
| Malhador | SE | 1 | 12.618 | 0,08 | 0,0 |
| Umbaúba | SE | 2 | 25.294 | 0,08 | 0,0 |
| Banzaê | BA | 1 | 13.229 | 0,08 | 0,0 |
| Castro Alves | BA | 2 | 26.264 | 0,08 | 0,1 |
| João Dourado | BA | 2 | 25.188 | 0,08 | 0,1 |
| Licínio de Almeida | BA | 1 | 12.388 | 0,08 | 0,0 |
| Santana | BA | 2 | 26.614 | 0,08 | 1,1 |
| Saubara | BA | 1 | 12.043 | 0,08 | 0,0 |
| Saúde | BA | 1 | 12.913 | 0,08 | 0,0 |
| Sítio do Mato | BA | 1 | 13.012 | 0,08 | 0,5 |
| Terra Nova | BA | 1 | 13.033 | 0,08 | 0,0 |
| Corinto | MG | 2 | 23.731 | 0,08 | 5,3 |
| Itajubá | MG | 8 | 96.869 | 0,08 | 0,0 |
| Lagoa da Prata | MG | 4 | 52.165 | 0,08 | 0,0 |
| Lagoa Santa | MG | 5 | 64.527 | 0,08 | 0,0 |
| Patos de Minas | MG | 12 | 152.488 | 0,08 | 0,3 |
| Pedras de Maria da Cruz | MG | 1 | 12.107 | 0,08 | 7,4 |
| Colatina | ES | 10 | 122.499 | 0,08 | 0,0 |
| Marilândia | ES | 1 | 12.833 | 0,08 | 0,0 |
| Barra do Piraí | RJ | 8 | 100.374 | 0,08 | 0,0 |
| Cardoso Moreira | RJ | 1 | 12.823 | 0,08 | 0,0 |
| São Fidélis | RJ | 3 | 38.669 | 0,08 | 4,9 |
| Arujá | SP | 7 | 89.824 | 0,08 | 0,0 |
| Atibaia | SP | 12 | 142.761 | 0,08 | 0,0 |
| Bocaina | SP | 1 | 12.329 | 0,08 | 0,0 |
| Duartina | SP | 1 | 12.445 | 0,08 | 0,0 |
| Guaiçara | SP | 1 | 12.168 | 0,08 | 0,0 |
| Itapetininga | SP | 13 | 163.901 | 0,08 | 0,0 |
| Lavínia | SP | 1 | 11.980 | 0,08 | 0,0 |
| Mineiros do Tietê | SP | 1 | 12.908 | 0,08 | 0,0 |
| Palestina | SP | 1 | 12.957 | 0,08 | 0,0 |
| Palmares Paulista | SP | 1 | 13.275 | 0,08 | 0,0 |
| Pereira Barreto | SP | 2 | 25.669 | 0,08 | 0,0 |
| Rio Grande da Serra | SP | 4 | 50.846 | 0,08 | 0,0 |
| Santa Cruz do Rio Pardo | SP | 4 | 47.673 | 0,08 | 0,1 |
| Valentim Gentil | SP | 1 | 13.326 | 0,08 | 0,0 |
| Vargem Grande Paulista | SP | 4 | 52.597 | 0,08 | 0,0 |
| Balsa Nova | PR | 1 | 12.941 | 0,08 | 0,0 |
| Bocaiúva do Sul | PR | 1 | 12.944 | 0,08 | 0,2 |
| Cidade Gaúcha | PR | 1 | 12.652 | 0,08 | 0,0 |
| Guaratuba | PR | 3 | 37.067 | 0,08 | 0,1 |
| Itapejara d'Oeste | PR | 1 | 11.964 | 0,08 | 0,0 |
| Jataizinho | PR | 1 | 12.588 | 0,08 | 0,0 |
| Marialva | PR | 3 | 35.496 | 0,08 | 0,0 |
| Moreira Sales | PR | 1 | 12.121 | 0,08 | 0,0 |
| Quatro Barras | PR | 2 | 23.559 | 0,08 | 0,0 |
| Canelinha | SC | 1 | 12.240 | 0,08 | 0,0 |
| Sangão | SC | 1 | 12.678 | 0,08 | 0,0 |
| São Lourenço do Oeste | SC | 2 | 24.076 | 0,08 | 0,0 |
| Turvo | SC | 1 | 12.899 | 0,08 | 0,0 |
| Alegrete | RS | 6 | 73.589 | 0,08 | 1,3 |
| Bom Retiro do Sul | RS | 1 | 12.328 | 0,08 | 0,0 |
| Cacequi | RS | 1 | 12.561 | 0,08 | 98,3 |
| Guaporé | RS | 2 | 25.727 | 0,08 | 0,1 |
| Igrejinha | RS | 3 | 36.899 | 0,08 | 0,0 |
| Pinheiro Machado | RS | 1 | 12.271 | 0,08 | 13,6 |
| Salto do Jacuí | RS | 1 | 12.449 | 0,08 | 99,6 |
| Tapejara | RS | 2 | 24.111 | 0,08 | 0,7 |
| Três Passos | RS | 2 | 23.906 | 0,08 | 0,0 |
| Veranópolis | RS | 2 | 26.241 | 0,08 | 1,3 |
| Deodápolis | MS | 1 | 12.924 | 0,08 | 0,3 |
| Maracaju | MS | 4 | 47.083 | 0,08 | 4,2 |
| Campo Novo do Parecis | MT | 3 | 35.360 | 0,08 | 100,0 |
| Cuiabá | MT | 52 | 612.547 | 0,08 | 0,1 |
| Sapezal | MT | 2 | 25.881 | 0,08 | 10,5 |
| Senador Canedo | GO | 9 | 115.371 | 0,08 | 0,0 |
| Peri Mirim | MA | 1 | 14.318 | 0,07 | 0,0 |
| Rosário | MA | 3 | 42.740 | 0,07 | 0,4 |
| Santana do Maranhão | MA | 1 | 13.386 | 0,07 | 11,5 |
| Senador La Rocque | MA | 1 | 14.293 | 0,07 | 4,1 |
| Inhuma | PI | 1 | 15.308 | 0,07 | 1,2 |
| Luís Correia | PI | 2 | 30.311 | 0,07 | 16,1 |
| Acarape | CE | 1 | 14.929 | 0,07 | 0,0 |
| Beberibe | CE | 4 | 53.573 | 0,07 | 52,5 |
| Cascavel | CE | 5 | 71.743 | 0,07 | 5,1 |
| Eusébio | CE | 4 | 53.618 | 0,07 | 0,0 |
| Graça | CE | 1 | 14.399 | 0,07 | 4,9 |
| Granja | CE | 4 | 54.748 | 0,07 | 53,1 |
| Novo Oriente | CE | 2 | 28.607 | 0,07 | 94,3 |
| Pacajus | CE | 5 | 72.203 | 0,07 | 0,0 |
| Redenção | CE | 2 | 29.053 | 0,07 | 0,0 |
| Uruoca | CE | 1 | 13.840 | 0,07 | 12,1 |
| Alexandria | RN | 1 | 13.577 | 0,07 | 100,0 |
| Arês | RN | 1 | 14.306 | 0,07 | 0,0 |
| Extremoz | RN | 2 | 28.583 | 0,07 | 0,0 |
| Ielmo Marinho | RN | 1 | 13.766 | 0,07 | 2,5 |
| São Gonçalo do Amarante | RN | 7 | 102.400 | 0,07 | 0,0 |
| São José de Mipibu | RN | 3 | 43.899 | 0,07 | 0,0 |
| Alagoa Grande | PB | 2 | 28.496 | 0,07 | 0,7 |
| Brejo do Cruz | PB | 1 | 14.122 | 0,07 | 1,4 |
| Catolé do Rocha | PB | 2 | 30.546 | 0,07 | 5,0 |
| Jacaraú | PB | 1 | 14.431 | 0,07 | 28,4 |
| Massaranduba | PB | 1 | 13.918 | 0,07 | 2,3 |
| Puxinanã | PB | 1 | 13.680 | 0,07 | 1,2 |
| Santa Rita | PB | 9 | 136.586 | 0,07 | 0,1 |
| Serra Branca | PB | 1 | 13.699 | 0,07 | 100,0 |
| Teixeira | PB | 1 | 15.161 | 0,07 | 96,7 |
| Arcoverde | PE | 5 | 74.338 | 0,07 | 0,0 |
| Casinhas | PE | 1 | 14.341 | 0,07 | 39,4 |
| Jatobá | PE | 1 | 14.796 | 0,07 | 0,0 |
| Jurema | PE | 1 | 15.378 | 0,07 | 13,4 |
| Orocó | PE | 1 | 14.991 | 0,07 | 22,3 |
| Santa Cruz do Capibaribe | PE | 8 | 107.937 | 0,07 | 0,3 |
| São Bento do Una | PE | 4 | 59.504 | 0,07 | 21,2 |
| Porto Calvo | AL | 2 | 27.165 | 0,07 | 9,7 |
| Monte Alegre de Sergipe | SE | 1 | 15.031 | 0,07 | 0,0 |
| Tomar do Geru | SE | 1 | 13.536 | 0,07 | 0,0 |
| Caetanos | BA | 1 | 14.542 | 0,07 | 15,7 |
| Cristópolis | BA | 1 | 13.910 | 0,07 | 2,4 |
| Mundo Novo | BA | 2 | 26.776 | 0,07 | 26,3 |
| Retirolândia | BA | 1 | 14.396 | 0,07 | 0,0 |
| Ribeira do Pombal | BA | 4 | 53.807 | 0,07 | 0,0 |
| Rio do Antônio | BA | 1 | 15.371 | 0,07 | 0,5 |
| Santa Brígida | BA | 1 | 14.213 | 0,07 | 2,2 |
| Vera Cruz | BA | 3 | 43.223 | 0,07 | 0,0 |
| Vitória da Conquista | BA | 24 | 338.480 | 0,07 | 0,2 |
| Águas Vermelhas | MG | 1 | 13.539 | 0,07 | 4,5 |
| Boa Esperança | MG | 3 | 40.127 | 0,07 | 0,0 |
| Cataguases | MG | 5 | 75.123 | 0,07 | 0,0 |
| Chapada do Norte | MG | 1 | 15.356 | 0,07 | 26,1 |
| Congonhas | MG | 4 | 54.762 | 0,07 | 0,0 |
| Dores do Indaiá | MG | 1 | 13.483 | 0,07 | 0,0 |
| Guapé | MG | 1 | 14.245 | 0,07 | 0,0 |
| Ibiraci | MG | 1 | 13.828 | 0,07 | 1,2 |
| Janaúba | MG | 5 | 71.648 | 0,07 | 0,8 |
| Maria da Fé | MG | 1 | 14.095 | 0,07 | 0,0 |
| Nepomuceno | MG | 2 | 26.769 | 0,07 | 0,0 |
| Ouro Preto | MG | 5 | 74.281 | 0,07 | 0,0 |
| Pirapora | MG | 4 | 56.428 | 0,07 | 0,1 |
| Pouso Alegre | MG | 11 | 150.737 | 0,07 | 0,0 |
| Três Pontas | MG | 4 | 56.746 | 0,07 | 0,0 |
| Anchieta | ES | 2 | 29.263 | 0,07 | 0,0 |
| Pinheiros | ES | 2 | 27.047 | 0,07 | 0,0 |
| Iguaba Grande | RJ | 2 | 28.310 | 0,07 | 0,0 |
| Itaperuna | RJ | 7 | 103.224 | 0,07 | 0,0 |
| São Francisco de Itabapoana | RJ | 3 | 42.205 | 0,07 | 0,2 |
| Três Rios | RJ | 6 | 81.804 | 0,07 | 0,0 |
| Avanhandava | SP | 1 | 13.649 | 0,07 | 0,0 |
| Birigui | SP | 9 | 123.638 | 0,07 | 0,0 |
| Boa Esperança do Sul | SP | 1 | 14.923 | 0,07 | 0,0 |
| Capivari | SP | 4 | 55.768 | 0,07 | 0,0 |
| Catanduva | SP | 8 | 121.862 | 0,07 | 0,0 |
| Dois Córregos | SP | 2 | 27.315 | 0,07 | 0,0 |
| Flórida Paulista | SP | 1 | 14.640 | 0,07 | 0,0 |
| Itapecerica da Serra | SP | 13 | 175.693 | 0,07 | 0,0 |
| Itapeva | SP | 7 | 94.354 | 0,07 | 0,1 |
| Itápolis | SP | 3 | 43.120 | 0,07 | 0,1 |
| Itaporanga | SP | 1 | 15.149 | 0,07 | 0,0 |
| Itu | SP | 13 | 173.939 | 0,07 | 0,0 |
| Jarinu | SP | 2 | 30.044 | 0,07 | 0,0 |
| Mairiporã | SP | 7 | 100.179 | 0,07 | 0,0 |
| Pacaembu | SP | 1 | 14.197 | 0,07 | 0,0 |
| Pindamonhangaba | SP | 12 | 168.328 | 0,07 | 0,0 |
| Piraju | SP | 2 | 29.806 | 0,07 | 0,0 |
| Promissão | SP | 3 | 40.432 | 0,07 | 0,0 |
| São João da Boa Vista | SP | 6 | 91.211 | 0,07 | 0,0 |
| Socorro | SP | 3 | 41.005 | 0,07 | 0,0 |
| Carlópolis | PR | 1 | 14.320 | 0,07 | 0,0 |
| Dois Vizinhos | PR | 3 | 40.641 | 0,07 | 0,0 |
| Iporã | PR | 1 | 13.926 | 0,07 | 0,0 |
| Jaguapitã | PR | 1 | 13.620 | 0,07 | 0,2 |
| Paraíso do Norte | PR | 1 | 13.830 | 0,07 | 0,0 |
| Rio Azul | PR | 1 | 15.236 | 0,07 | 0,0 |
| Salto do Lontra | PR | 1 | 14.785 | 0,07 | 0,0 |
| Santa Izabel do Oeste | PR | 1 | 14.659 | 0,07 | 0,0 |
| São Miguel do Iguaçu | PR | 2 | 27.452 | 0,07 | 0,4 |
| Barra Velha | SC | 2 | 29.168 | 0,07 | 0,0 |
| Blumenau | SC | 26 | 357.199 | 0,07 | 0,0 |
| Gaspar | SC | 5 | 69.639 | 0,07 | 0,0 |
| Indaial | SC | 5 | 69.425 | 0,07 | 0,0 |
| Jaraguá do Sul | SC | 12 | 177.697 | 0,07 | 0,0 |
| Lauro Muller | SC | 1 | 15.244 | 0,07 | 0,0 |
| Nova Veneza | SC | 1 | 15.166 | 0,07 | 0,0 |
| Rio Negrinho | SC | 3 | 42.302 | 0,07 | 0,0 |
| Arroio dos Ratos | RS | 1 | 14.151 | 0,07 | 0,5 |
| Balneário Pinhal | RS | 1 | 14.068 | 0,07 | 0,0 |
| Barra do Ribeiro | RS | 1 | 13.491 | 0,07 | 1,9 |
| Cerro Largo | RS | 1 | 14.133 | 0,07 | 0,0 |
| Santa Rosa | RS | 5 | 73.254 | 0,07 | 0,0 |
| Sobradinho | RS | 1 | 14.967 | 0,07 | 0,3 |
| Tenente Portela | RS | 1 | 13.485 | 0,07 | 0,1 |
| Vera Cruz | RS | 2 | 26.863 | 0,07 | 0,0 |
| Cidade Ocidental | GO | 5 | 71.376 | 0,07 | 0,0 |
| Trindade | GO | 9 | 127.599 | 0,07 | 0,0 |
| Lago Verde | MA | 1 | 16.257 | 0,06 | 5,6 |
| Paulino Neves | MA | 1 | 16.035 | 0,06 | 27,1 |
| Pinheiro | MA | 5 | 83.387 | 0,06 | 0,9 |
| Santo Amaro do Maranhão | MA | 1 | 15.846 | 0,06 | 6,2 |
| Piripiri | PI | 4 | 63.742 | 0,06 | 1,1 |
| São Raimundo Nonato | PI | 2 | 34.710 | 0,06 | 0,3 |
| Acopiara | CE | 3 | 54.270 | 0,06 | 100,0 |
| Poço Branco | RN | 1 | 15.413 | 0,06 | 0,0 |
| Belém | PB | 1 | 17.676 | 0,06 | 1,9 |
| Cajazeiras | PB | 4 | 61.993 | 0,06 | 100,0 |
| Cruz do ES | PB | 1 | 17.319 | 0,06 | 0,0 |
| Ingá | PB | 1 | 18.103 | 0,06 | 0,0 |
| Carpina | PE | 5 | 83.641 | 0,06 | 0,1 |
| Escada | PE | 4 | 68.875 | 0,06 | 0,5 |
| São Benedito do Sul | PE | 1 | 15.895 | 0,06 | 19,6 |
| Lagoa da Canoa | AL | 1 | 17.852 | 0,06 | 0,0 |
| Frei Paulo | SE | 1 | 15.421 | 0,06 | 0,0 |
| Itabaiana | SE | 6 | 95.427 | 0,06 | 0,0 |
| Aporá | BA | 1 | 17.731 | 0,06 | 0,7 |
| Belo Campo | BA | 1 | 17.211 | 0,06 | 4,9 |
| Filadélfia | BA | 1 | 16.377 | 0,06 | 0,1 |
| Itaberaba | BA | 4 | 64.489 | 0,06 | 3,2 |
| Itapicuru | BA | 2 | 35.576 | 0,06 | 0,4 |
| Juazeiro | BA | 12 | 216.707 | 0,06 | 1,3 |
| Mata de São João | BA | 3 | 46.583 | 0,06 | 0,5 |
| Monte Santo | BA | 3 | 49.418 | 0,06 | 8,5 |
| Novo Triunfo | BA | 1 | 15.440 | 0,06 | 6,7 |
| Pedro Alexandre | BA | 1 | 16.667 | 0,06 | 7,0 |
| Santo Antônio de Jesus | BA | 6 | 101.512 | 0,06 | 0,0 |
| Tanque Novo | BA | 1 | 17.366 | 0,06 | 0,6 |
| Tapiramutá | BA | 1 | 17.010 | 0,06 | 1,0 |
| Coronel Fabriciano | MG | 7 | 109.855 | 0,06 | 0,0 |
| Extrema | MG | 2 | 36.225 | 0,06 | 0,0 |
| Itaú de Minas | MG | 1 | 16.108 | 0,06 | 0,0 |
| Muriaé | MG | 7 | 108.763 | 0,06 | 0,0 |
| Nova Era | MG | 1 | 17.578 | 0,06 | 0,0 |
| Papagaios | MG | 1 | 15.674 | 0,06 | 1,0 |
| Pará de Minas | MG | 6 | 93.969 | 0,06 | 0,0 |
| Passos | MG | 7 | 114.679 | 0,06 | 0,1 |
| São Joaquim de Bicas | MG | 2 | 31.578 | 0,06 | 0,0 |
| Tocantins | MG | 1 | 16.659 | 0,06 | 0,0 |
| Três Corações | MG | 5 | 79.482 | 0,06 | 0,0 |
| Uberlândia | MG | 40 | 691.305 | 0,06 | 0,0 |
| Guarapari | ES | 8 | 124.859 | 0,06 | 0,0 |
| São João da Barra | RJ | 2 | 36.102 | 0,06 | 0,0 |
| Aguaí | SP | 2 | 36.305 | 0,06 | 0,0 |
| Altinópolis | SP | 1 | 16.184 | 0,06 | 0,1 |
| Bady Bassitt | SP | 1 | 17.502 | 0,06 | 0,0 |
| Bariri | SP | 2 | 35.264 | 0,06 | 0,0 |
| Batatais | SP | 4 | 62.508 | 0,06 | 0,0 |
| Bertioga | SP | 4 | 63.249 | 0,06 | 0,0 |
| Borborema | SP | 1 | 16.046 | 0,06 | 0,1 |
| Cotia | SP | 15 | 249.210 | 0,06 | 0,0 |
| Cruzeiro | SP | 5 | 82.238 | 0,06 | 0,0 |
| Fartura | SP | 1 | 16.036 | 0,06 | 0,0 |
| Fernandópolis | SP | 4 | 69.116 | 0,06 | 0,0 |
| Francisco Morato | SP | 11 | 175.844 | 0,06 | 0,0 |
| Guararapes | SP | 2 | 32.939 | 0,06 | 0,0 |
| Ipuã | SP | 1 | 16.409 | 0,06 | 0,0 |
| Itararé | SP | 3 | 50.503 | 0,06 | 0,0 |
| Jales | SP | 3 | 49.107 | 0,06 | 0,0 |
| Matão | SP | 5 | 83.170 | 0,06 | 0,0 |
| Mogi Mirim | SP | 6 | 93.189 | 0,06 | 0,0 |
| Pindorama | SP | 1 | 17.049 | 0,06 | 0,0 |
| Ribeirão Pires | SP | 7 | 123.393 | 0,06 | 0,0 |
| Cafelândia | PR | 1 | 18.120 | 0,06 | 0,0 |
| Candói | PR | 1 | 15.979 | 0,06 | 5,0 |
| Capitão Leônidas Marques | PR | 1 | 15.780 | 0,06 | 1,2 |
| Cornélio Procópio | PR | 3 | 47.845 | 0,06 | 0,0 |
| Guaíra | PR | 2 | 33.119 | 0,06 | 0,0 |
| Quedas do Iguaçu | PR | 2 | 34.103 | 0,06 | 0,0 |
| Realeza | PR | 1 | 16.922 | 0,06 | 0,0 |
| Rio Negro | PR | 2 | 34.170 | 0,06 | 0,0 |
| Terra Boa | PR | 1 | 17.094 | 0,06 | 0,0 |
| Toledo | PR | 8 | 140.635 | 0,06 | 0,0 |
| Biguaçu | SC | 4 | 68.481 | 0,06 | 0,0 |
| Dionísio Cerqueira | SC | 1 | 15.498 | 0,06 | 0,0 |
| Itapiranga | SC | 1 | 16.872 | 0,06 | 0,0 |
| Morro da Fumaça | SC | 1 | 17.796 | 0,06 | 0,0 |
| Presidente Getúlio | SC | 1 | 17.471 | 0,06 | 1,4 |
| Carazinho | RS | 4 | 62.110 | 0,06 | 0,0 |
| Farroupilha | RS | 4 | 72.331 | 0,06 | 0,0 |
| Ijuí | RS | 5 | 83.475 | 0,06 | 0,0 |
| Sananduva | RS | 1 | 16.270 | 0,06 | 7,1 |
| Venâncio Aires | RS | 4 | 71.554 | 0,06 | 0,0 |
| Dourados | MS | 13 | 222.949 | 0,06 | 0,4 |
| Novo Gama | GO | 7 | 115.711 | 0,06 | 0,0 |
| Manaus | AM | 102 | 2.182.763 | 0,05 | 0,2 |
| Barão de Grajaú | MA | 1 | 18.820 | 0,05 | 27,2 |
| Governador Edison Lobão | MA | 1 | 18.296 | 0,05 | 0,0 |
| Olho d'Água das Cunhãs | MA | 1 | 19.505 | 0,05 | 0,2 |
| Palmeirândia | MA | 1 | 19.722 | 0,05 | 3,2 |
| Tutóia | MA | 3 | 58.860 | 0,05 | 4,2 |
| José de Freitas | PI | 2 | 39.208 | 0,05 | 1,7 |
| Parnaíba | PI | 8 | 153.078 | 0,05 | 0,0 |
| São João do Piauí | PI | 1 | 20.601 | 0,05 | 0,3 |
| Amontada | CE | 2 | 43.452 | 0,05 | 58,3 |
| Camocim | CE | 3 | 63.661 | 0,05 | 96,8 |
| Catarina | CE | 1 | 20.698 | 0,05 | 100,0 |
| Chorozinho | CE | 1 | 20.264 | 0,05 | 6,9 |
| Iguatu | CE | 5 | 102.498 | 0,05 | 100,0 |
| Itaitinga | CE | 2 | 37.980 | 0,05 | 0,0 |
| Itarema | CE | 2 | 41.826 | 0,05 | 92,0 |
| Pindoretama | CE | 1 | 20.567 | 0,05 | 0,0 |
| Umirim | CE | 1 | 19.825 | 0,05 | 40,0 |
| Caaporã | PB | 1 | 21.828 | 0,05 | 0,0 |
| Campina Grande | PB | 22 | 409.731 | 0,05 | 0,5 |
| Itapororoca | PB | 1 | 18.664 | 0,05 | 13,7 |
| Pitimbu | PB | 1 | 19.065 | 0,05 | 0,0 |
| São José de Piranhas | PB | 1 | 20.251 | 0,05 | 100,0 |
| Igarassu | PE | 6 | 117.019 | 0,05 | 0,0 |
| Jaboatão dos Guararapes | PE | 36 | 702.298 | 0,05 | 0,0 |
| Lajedo | PE | 2 | 40.288 | 0,05 | 0,1 |
| Paulista | PE | 16 | 331.774 | 0,05 | 0,0 |
| Riacho das Almas | PE | 1 | 20.546 | 0,05 | 0,3 |
| São Joaquim do Monte | PE | 1 | 21.356 | 0,05 | 0,0 |
| Surubim | PE | 3 | 65.089 | 0,05 | 1,5 |
| Arapiraca | AL | 12 | 231.747 | 0,05 | 0,0 |
| Cajueiro | AL | 1 | 21.264 | 0,05 | 0,0 |
| Feira Grande | AL | 1 | 22.166 | 0,05 | 0,0 |
| Inhapi | AL | 1 | 18.385 | 0,05 | 0,0 |
| Palmeira dos Índios | AL | 4 | 73.218 | 0,05 | 0,0 |
| Taquarana | AL | 1 | 19.980 | 0,05 | 0,0 |
| Aquidabã | SE | 1 | 21.563 | 0,05 | 0,0 |
| Riachão do Dantas | SE | 1 | 19.805 | 0,05 | 0,0 |
| Buritirama | BA | 1 | 21.174 | 0,05 | 18,5 |
| Crisópolis | BA | 1 | 21.103 | 0,05 | 2,0 |
| Euclides da Cunha | BA | 3 | 60.585 | 0,05 | 3,4 |
| Inhambupe | BA | 2 | 39.926 | 0,05 | 6,6 |
| Mairi | BA | 1 | 18.676 | 0,05 | 2,3 |
| Santaluz | BA | 2 | 37.348 | 0,05 | 1,2 |
| Tanhaçu | BA | 1 | 20.403 | 0,05 | 1,1 |
| Umburanas | BA | 1 | 19.222 | 0,05 | 57,9 |
| Barroso | MG | 1 | 20.810 | 0,05 | 0,0 |
| Camanducaia | MG | 1 | 21.770 | 0,05 | 0,0 |
| Conselheiro Lafaiete | MG | 6 | 128.589 | 0,05 | 0,0 |
| Guaranésia | MG | 1 | 19.021 | 0,05 | 0,0 |
| Ipatinga | MG | 13 | 263.410 | 0,05 | 0,0 |
| Juiz de Fora | MG | 26 | 568.873 | 0,05 | 0,0 |
| Luz | MG | 1 | 18.215 | 0,05 | 0,0 |
| Nova Lima | MG | 5 | 94.889 | 0,05 | 0,0 |
| Nova Serrana | MG | 5 | 102.693 | 0,05 | 0,0 |
| Oliveira | MG | 2 | 41.687 | 0,05 | 0,0 |
| Paraguaçu | MG | 1 | 21.513 | 0,05 | 0,0 |
| Paraisópolis | MG | 1 | 21.083 | 0,05 | 0,0 |
| Sete Lagoas | MG | 11 | 239.639 | 0,05 | 0,0 |
| Cachoeiro de Itapemirim | ES | 11 | 208.972 | 0,05 | 0,0 |
| Piúma | ES | 1 | 21.711 | 0,05 | 0,0 |
| Araruama | RJ | 6 | 132.400 | 0,05 | 0,0 |
| Carmo | RJ | 1 | 18.895 | 0,05 | 0,0 |
| Cordeiro | RJ | 1 | 21.926 | 0,05 | 0,0 |
| Itaguaí | RJ | 7 | 133.019 | 0,05 | 0,0 |
| Maricá | RJ | 8 | 161.207 | 0,05 | 0,0 |
| Resende | RJ | 7 | 131.341 | 0,05 | 0,0 |
| Águas de Lindóia | SP | 1 | 18.705 | 0,05 | 0,0 |
| Bastos | SP | 1 | 20.953 | 0,05 | 0,0 |
| Campo Limpo Paulista | SP | 4 | 84.650 | 0,05 | 0,0 |
| Cubatão | SP | 6 | 130.705 | 0,05 | 0,0 |
| Guariba | SP | 2 | 40.105 | 0,05 | 0,0 |
| Ibitinga | SP | 3 | 60.033 | 0,05 | 0,0 |
| Iperó | SP | 2 | 37.133 | 0,05 | 0,0 |
| Itatiba | SP | 6 | 120.858 | 0,05 | 0,0 |
| Itatinga | SP | 1 | 20.697 | 0,05 | 0,1 |
| Ituverava | SP | 2 | 41.824 | 0,05 | 0,0 |
| Jaguariúna | SP | 3 | 57.488 | 0,05 | 0,0 |
| Junqueirópolis | SP | 1 | 20.679 | 0,05 | 0,0 |
| Lins | SP | 4 | 78.013 | 0,05 | 0,0 |
| Lorena | SP | 4 | 88.706 | 0,05 | 0,0 |
| Mogi Guaçu | SP | 7 | 151.888 | 0,05 | 0,0 |
| Novo Horizonte | SP | 2 | 41.052 | 0,05 | 0,0 |
| Pradópolis | SP | 1 | 21.496 | 0,05 | 0,0 |
| São Roque | SP | 5 | 91.016 | 0,05 | 0,0 |
| Taquaritinga | SP | 3 | 57.177 | 0,05 | 0,0 |
| Tatuí | SP | 6 | 121.766 | 0,05 | 0,0 |
| Tupã | SP | 3 | 65.524 | 0,05 | 0,0 |
| Arapongas | PR | 6 | 123.027 | 0,05 | 0,0 |
| Campo Largo | PR | 6 | 132.002 | 0,05 | 0,0 |
| Paranavaí | PR | 4 | 88.374 | 0,05 | 0,0 |
| Siqueira Campos | PR | 1 | 21.016 | 0,05 | 0,0 |
| Telêmaco Borba | PR | 4 | 78.974 | 0,05 | 0,0 |
| Caçador | SC | 4 | 78.595 | 0,05 | 0,0 |
| Criciúma | SC | 11 | 215.186 | 0,05 | 0,0 |
| Mafra | SC | 3 | 56.292 | 0,05 | 0,9 |
| Navegantes | SC | 4 | 81.475 | 0,05 | 0,0 |
| Otacílio Costa | SC | 1 | 18.744 | 0,05 | 0,0 |
| Palhoça | SC | 8 | 171.797 | 0,05 | 0,0 |
| Schroeder | SC | 1 | 21.365 | 0,05 | 0,0 |
| Tijucas | SC | 2 | 38.407 | 0,05 | 0,0 |
| Timbó | SC | 2 | 44.238 | 0,05 | 0,0 |
| Tubarão | SC | 5 | 105.686 | 0,05 | 0,0 |
| Urussanga | SC | 1 | 21.268 | 0,05 | 0,0 |
| Arroio do Meio | RS | 1 | 20.805 | 0,05 | 0,0 |
| Butiá | RS | 1 | 20.941 | 0,05 | 0,6 |
| Charqueadas | RS | 2 | 40.789 | 0,05 | 0,0 |
| Cruz Alta | RS | 3 | 60.299 | 0,05 | 0,4 |
| Pelotas | RS | 16 | 342.405 | 0,05 | 0,0 |
| Rio Grande | RS | 11 | 211.005 | 0,05 | 0,1 |
| Rolante | RS | 1 | 21.349 | 0,05 | 0,0 |
| Santa Cruz do Sul | RS | 6 | 130.416 | 0,05 | 0,0 |
| Santa Maria | RS | 13 | 282.123 | 0,05 | 0,0 |
| Santo Ângelo | RS | 4 | 77.593 | 0,05 | 0,0 |
| Ceres | GO | 1 | 22.191 | 0,05 | 0,0 |
| Goianira | GO | 2 | 44.289 | 0,05 | 0,0 |
| Santa Helena de Goiás | GO | 2 | 38.648 | 0,05 | 0,0 |
| Belém | PA | 59 | 1.492.745 | 0,04 | 0,0 |
| Matinha | MA | 1 | 23.370 | 0,04 | 11,6 |
| Corrente | PI | 1 | 26.644 | 0,04 | 22,9 |
| Aquiraz | CE | 3 | 80.271 | 0,04 | 0,0 |
| Barreira | CE | 1 | 22.425 | 0,04 | 0,6 |
| Barro | CE | 1 | 22.680 | 0,04 | 32,9 |
| Cedro | CE | 1 | 25.557 | 0,04 | 34,0 |
| Coreaú | CE | 1 | 23.136 | 0,04 | 1,4 |
| Cruz | CE | 1 | 24.827 | 0,04 | 55,9 |
| Horizonte | CE | 3 | 67.337 | 0,04 | 0,0 |
| Irauçuba | CE | 1 | 24.156 | 0,04 | 28,2 |
| Marco | CE | 1 | 27.361 | 0,04 | 2,5 |
| Morrinhos | CE | 1 | 22.534 | 0,04 | 2,1 |
| Trairi | CE | 2 | 55.918 | 0,04 | 98,9 |
| Nísia Floresta | RN | 1 | 27.602 | 0,04 | 0,0 |
| Santo Antônio | RN | 1 | 24.136 | 0,04 | 2,5 |
| Areia | PB | 1 | 22.819 | 0,04 | 4,7 |
| Bayeux | PB | 4 | 96.880 | 0,04 | 0,0 |
| Itabaiana | PB | 1 | 24.477 | 0,04 | 0,0 |
| Patos | PB | 4 | 107.605 | 0,04 | 1,4 |
| Pedras de Fogo | PB | 1 | 28.458 | 0,04 | 0,0 |
| Sapé | PB | 2 | 52.625 | 0,04 | 1,5 |
| Garanhuns | PE | 6 | 139.788 | 0,04 | 0,4 |
| Itapissuma | PE | 1 | 26.651 | 0,04 | 0,0 |
| Olinda | PE | 16 | 392.482 | 0,04 | 0,0 |
| Sirinhaém | PE | 2 | 45.865 | 0,04 | 0,8 |
| Maceió | AL | 37 | 1.018.948 | 0,04 | 0,0 |
| São José da Laje | AL | 1 | 23.927 | 0,04 | 12,1 |
| Nossa Senhora do Socorro | SE | 8 | 183.628 | 0,04 | 0,0 |
| Poço Verde | SE | 1 | 23.728 | 0,04 | 0,0 |
| Caculé | BA | 1 | 23.170 | 0,04 | 0,2 |
| Caetité | BA | 2 | 50.975 | 0,04 | 1,0 |
| Nova Soure | BA | 1 | 26.947 | 0,04 | 0,0 |
| Olindina | BA | 1 | 28.229 | 0,04 | 0,0 |
| Campo Belo | MG | 2 | 54.029 | 0,04 | 0,0 |
| Guaxupé | MG | 2 | 51.917 | 0,04 | 0,0 |
| Jacutinga | MG | 1 | 25.979 | 0,04 | 0,0 |
| Juatuba | MG | 1 | 26.946 | 0,04 | 0,0 |
| Lavras | MG | 4 | 103.773 | 0,04 | 0,0 |
| São Lourenço | MG | 2 | 45.851 | 0,04 | 0,0 |
| Cabo Frio | RJ | 8 | 226.525 | 0,04 | 0,0 |
| Conceição de Macabu | RJ | 1 | 23.228 | 0,04 | 0,0 |
| Japeri | RJ | 4 | 104.768 | 0,04 | 0,0 |
| Macaé | RJ | 9 | 256.672 | 0,04 | 0,0 |
| Miguel Pereira | RJ | 1 | 25.538 | 0,04 | 0,0 |
| Pinheiral | RJ | 1 | 25.156 | 0,04 | 0,0 |
| Quissamã | RJ | 1 | 24.700 | 0,04 | 0,1 |
| Seropédica | RJ | 3 | 82.312 | 0,04 | 0,0 |
| Apiaí | SP | 1 | 24.374 | 0,04 | 0,0 |
| Araçatuba | SP | 7 | 197.016 | 0,04 | 0,3 |
| Barretos | SP | 5 | 122.098 | 0,04 | 0,0 |
| Bebedouro | SP | 3 | 77.496 | 0,04 | 0,0 |
| Bragança Paulista | SP | 6 | 168.668 | 0,04 | 0,0 |
| Brodowski | SP | 1 | 24.939 | 0,04 | 0,0 |
| Brotas | SP | 1 | 24.403 | 0,04 | 0,0 |
| Caçapava | SP | 4 | 94.263 | 0,04 | 0,0 |
| Cajamar | SP | 3 | 76.801 | 0,04 | 0,0 |
| Cordeirópolis | SP | 1 | 24.528 | 0,04 | 0,0 |
| Igaraçu do Tietê | SP | 1 | 24.674 | 0,04 | 0,0 |
| Iracemápolis | SP | 1 | 24.235 | 0,04 | 0,0 |
| Louveira | SP | 2 | 48.885 | 0,04 | 0,0 |
| Miguelópolis | SP | 1 | 22.226 | 0,04 | 0,4 |
| Mogi das Cruzes | SP | 20 | 445.842 | 0,04 | 0,0 |
| Olímpia | SP | 2 | 54.772 | 0,04 | 0,0 |
| Piracicaba | SP | 15 | 404.142 | 0,04 | 0,0 |
| Pirajuí | SP | 1 | 25.492 | 0,04 | 0,0 |
| Pirapozinho | SP | 1 | 27.527 | 0,04 | 0,1 |
| Salto de Pirapora | SP | 2 | 45.422 | 0,04 | 0,0 |
| Suzano | SP | 11 | 297.637 | 0,04 | 0,0 |
| Valparaíso | SP | 1 | 26.480 | 0,04 | 0,1 |
| Votuporanga | SP | 4 | 94.547 | 0,04 | 0,0 |
| Arapoti | PR | 1 | 28.115 | 0,04 | 0,0 |
| Cascavel | PR | 12 | 328.454 | 0,04 | 0,1 |
| Colombo | PR | 9 | 243.726 | 0,04 | 0,0 |
| Colorado | PR | 1 | 24.012 | 0,04 | 0,0 |
| Foz do Iguaçu | PR | 11 | 258.532 | 0,04 | 0,0 |
| Mandaguaçu | PR | 1 | 22.819 | 0,04 | 0,0 |
| Nova Esperança | PR | 1 | 27.904 | 0,04 | 0,0 |
| Piraquara | PR | 4 | 113.036 | 0,04 | 0,0 |
| Santa Terezinha de Itaipu | PR | 1 | 23.465 | 0,04 | 0,0 |
| Santo Antônio da Platina | PR | 2 | 45.993 | 0,04 | 0,0 |
| São Mateus do Sul | PR | 2 | 46.261 | 0,04 | 0,0 |
| Umuarama | PR | 5 | 111.557 | 0,04 | 0,0 |
| Araranguá | SC | 3 | 68.228 | 0,04 | 0,0 |
| Balneário Camboriú | SC | 6 | 142.295 | 0,04 | 0,0 |
| Florianópolis | SC | 20 | 500.973 | 0,04 | 0,0 |
| Ituporanga | SC | 1 | 25.086 | 0,04 | 0,0 |
| Joinville | SC | 24 | 590.466 | 0,04 | 0,0 |
| Balneário Piçarras | SC | 1 | 23.147 | 0,04 | 0,0 |
| Rio do Sul | SC | 3 | 71.061 | 0,04 | 0,0 |
| São José | SC | 9 | 246.586 | 0,04 | 0,0 |
| Videira | SC | 2 | 53.065 | 0,04 | 0,0 |
| Bagé | RS | 5 | 121.143 | 0,04 | 0,2 |
| Capão da Canoa | RS | 2 | 53.049 | 0,04 | 0,0 |
| Capão do Leão | RS | 1 | 25.354 | 0,04 | 0,0 |
| Jaguarão | RS | 1 | 26.680 | 0,04 | 100,0 |
| Lagoa Vermelha | RS | 1 | 27.807 | 0,04 | 7,0 |
| Nova Prata | RS | 1 | 27.257 | 0,04 | 1,0 |
| Sant'Ana do Livramento | RS | 3 | 77.027 | 0,04 | 100,0 |
| São Sebastião do Caí | RS | 1 | 25.685 | 0,04 | 0,0 |
| Taquari | RS | 1 | 26.862 | 0,04 | 0,0 |
| Itaporã | MS | 1 | 24.839 | 0,04 | 0,0 |
| Ladário | MS | 1 | 23.331 | 0,04 | 0,0 |
| Jaraguá | GO | 2 | 50.511 | 0,04 | 0,0 |
| Brasília | DF | 125 | 3.015.268 | 0,04 | 0,0 |
| Ananindeua | PA | 15 | 530.598 | 0,03 | 0,0 |
| Penalva | MA | 1 | 38.470 | 0,03 | 21,9 |
| Raposa | MA | 1 | 30.761 | 0,03 | 100,0 |
| Timon | MA | 5 | 169.107 | 0,03 | 1,5 |
| Baturité | CE | 1 | 35.750 | 0,03 | 5,7 |
| Bela Cruz | CE | 1 | 32.591 | 0,03 | 24,6 |
| Massapê | CE | 1 | 38.737 | 0,03 | 0,0 |
| Paracuru | CE | 1 | 35.076 | 0,03 | 1,6 |
| Paraipaba | CE | 1 | 32.744 | 0,03 | 26,5 |
| Natal | RN | 27 | 884.122 | 0,03 | 0,0 |
| Guarabira | PB | 2 | 58.833 | 0,03 | 0,1 |
| João Pessoa | PB | 28 | 809.015 | 0,03 | 0,0 |
| Sousa | PB | 2 | 69.444 | 0,03 | 100,0 |
| Itambé | PE | 1 | 36.447 | 0,03 | 0,0 |
| Nazaré da Mata | PE | 1 | 32.471 | 0,03 | 0,0 |
| Vicência | PE | 1 | 32.643 | 0,03 | 6,5 |
| Barra dos Coqueiros | SE | 1 | 30.407 | 0,03 | 0,0 |
| Poço Redondo | SE | 1 | 34.775 | 0,03 | 0,5 |
| Propriá | SE | 1 | 29.626 | 0,03 | 0,0 |
| Capim Grosso | BA | 1 | 30.662 | 0,03 | 0,0 |
| Conceição do Coité | BA | 2 | 66.612 | 0,03 | 0,0 |
| Feira de Santana | BA | 19 | 614.872 | 0,03 | 0,2 |
| Muritiba | BA | 1 | 29.399 | 0,03 | 0,0 |
| Paratinga | BA | 1 | 32.000 | 0,03 | 25,6 |
| Paulo Afonso | BA | 4 | 117.782 | 0,03 | 0,2 |
| Campos Gerais | MG | 1 | 28.774 | 0,03 | 0,0 |
| Espinosa | MG | 1 | 31.617 | 0,03 | 3,6 |
| Ibirité | MG | 5 | 180.204 | 0,03 | 0,0 |
| Itaúna | MG | 3 | 93.214 | 0,03 | 0,0 |
| Matozinhos | MG | 1 | 37.820 | 0,03 | 0,0 |
| Ouro Branco | MG | 1 | 39.500 | 0,03 | 0,0 |
| Pedro Leopoldo | MG | 2 | 64.258 | 0,03 | 0,0 |
| Poços de Caldas | MG | 5 | 167.397 | 0,03 | 0,0 |
| Ponte Nova | MG | 2 | 59.742 | 0,03 | 0,0 |
| Niterói | RJ | 16 | 513.584 | 0,03 | 0,0 |
| Volta Redonda | RJ | 8 | 273.012 | 0,03 | 0,0 |
| Adamantina | SP | 1 | 35.068 | 0,03 | 0,0 |
| Aparecida | SP | 1 | 36.157 | 0,03 | 0,1 |
| Araçoiaba da Serra | SP | 1 | 34.146 | 0,03 | 0,0 |
| Assis | SP | 3 | 104.386 | 0,03 | 0,0 |
| Barra Bonita | SP | 1 | 36.126 | 0,03 | 0,0 |
| Barrinha | SP | 1 | 32.812 | 0,03 | 0,0 |
| Boituva | SP | 2 | 60.997 | 0,03 | 0,0 |
| Caieiras | SP | 3 | 101.470 | 0,03 | 0,0 |
| Cândido Mota | SP | 1 | 31.280 | 0,03 | 0,0 |
| Casa Branca | SP | 1 | 30.380 | 0,03 | 0,0 |
| Guarujá | SP | 9 | 320.459 | 0,03 | 0,0 |
| Ibaté | SP | 1 | 35.104 | 0,03 | 0,0 |
| Itupeva | SP | 2 | 61.252 | 0,03 | 0,0 |
| Jaboticabal | SP | 2 | 77.263 | 0,03 | 0,0 |
| Jacareí | SP | 6 | 233.662 | 0,03 | 0,0 |
| Limeira | SP | 9 | 306.114 | 0,03 | 0,0 |
| Mirassol | SP | 2 | 59.824 | 0,03 | 0,0 |
| Osvaldo Cruz | SP | 1 | 32.879 | 0,03 | 0,0 |
| Ourinhos | SP | 3 | 113.542 | 0,03 | 0,0 |
| Pirassununga | SP | 2 | 76.409 | 0,03 | 0,0 |
| Presidente Venceslau | SP | 1 | 39.516 | 0,03 | 0,0 |
| Rancharia | SP | 1 | 29.707 | 0,03 | 0,0 |
| Santa Fé do Sul | SP | 1 | 32.322 | 0,03 | 0,0 |
| São José do Rio Preto | SP | 15 | 460.671 | 0,03 | 0,0 |
| São José dos Campos | SP | 22 | 721.944 | 0,03 | 0,0 |
| São Pedro | SP | 1 | 35.653 | 0,03 | 0,0 |
| Taubaté | SP | 11 | 314.924 | 0,03 | 0,0 |
| Votorantim | SP | 4 | 122.480 | 0,03 | 0,0 |
| Almirante Tamandaré | PR | 4 | 118.623 | 0,03 | 0,0 |
| Apucarana | PR | 4 | 134.996 | 0,03 | 0,0 |
| Francisco Beltrão | PR | 3 | 91.093 | 0,03 | 0,0 |
| Goioerê | PR | 1 | 28.884 | 0,03 | 0,0 |
| Guarapuava | PR | 5 | 181.504 | 0,03 | 0,2 |
| Irati | PR | 2 | 60.727 | 0,03 | 0,0 |
| Jaguariaíva | PR | 1 | 34.857 | 0,03 | 0,1 |
| Mandaguari | PR | 1 | 34.400 | 0,03 | 0,0 |
| Maringá | PR | 11 | 423.666 | 0,03 | 0,0 |
| Palotina | PR | 1 | 31.846 | 0,03 | 0,0 |
| Ponta Grossa | PR | 9 | 351.736 | 0,03 | 0,0 |
| Braço do Norte | SC | 1 | 33.450 | 0,03 | 0,0 |
| Campos Novos | SC | 1 | 36.244 | 0,03 | 0,0 |
| Chapecó | SC | 7 | 220.367 | 0,03 | 0,0 |
| Penha | SC | 1 | 32.531 | 0,03 | 0,0 |
| São João Batista | SC | 1 | 37.424 | 0,03 | 0,0 |
| Candelária | RS | 1 | 31.365 | 0,03 | 0,1 |
| Caxias do Sul | RS | 16 | 510.906 | 0,03 | 0,0 |
| Dois Irmãos | RS | 1 | 32.671 | 0,03 | 0,0 |
| Estrela | RS | 1 | 34.116 | 0,03 | 0,0 |
| Flores da Cunha | RS | 1 | 30.745 | 0,03 | 0,0 |
| Garibaldi | RS | 1 | 35.070 | 0,03 | 0,0 |
| Gramado | RS | 1 | 36.232 | 0,03 | 0,0 |
| Parobé | RS | 2 | 58.272 | 0,03 | 0,0 |
| Torres | RS | 1 | 38.732 | 0,03 | 0,0 |
| Uruguaiana | RS | 4 | 126.970 | 0,03 | 99,8 |
| Campo Grande | MS | 29 | 895.982 | 0,03 | 0,1 |
| Águas Lindas de Goiás | GO | 6 | 212.440 | 0,03 | 0,0 |
| Aparecida de Goiânia | GO | 17 | 578.179 | 0,03 | 0,0 |
| Goiânia | GO | 46 | 1.516.113 | 0,03 | 0,0 |
| Nerópolis | GO | 1 | 29.850 | 0,03 | 0,0 |
| Santo Antônio do Descoberto | GO | 2 | 74.744 | 0,03 | 0,0 |
| Valparaíso de Goiás | GO | 5 | 168.468 | 0,03 | 0,0 |
| Araioses | MA | 1 | 46.440 | 0,02 | 2,8 |
| Teresina | PI | 17 | 864.845 | 0,02 | 0,0 |
| Caucaia | CE | 9 | 361.400 | 0,02 | 0,3 |
| Itapagé | CE | 1 | 52.675 | 0,02 | 18,4 |
| Itapipoca | CE | 3 | 129.358 | 0,02 | 85,2 |
| Juazeiro do Norte | CE | 5 | 274.207 | 0,02 | 0,0 |
| Maranguape | CE | 2 | 128.978 | 0,02 | 0,2 |
| Sobral | CE | 4 | 208.935 | 0,02 | 0,6 |
| Parnamirim | RN | 4 | 261.469 | 0,02 | 0,0 |
| Bom Conselho | PE | 1 | 48.554 | 0,02 | 0,0 |
| Moreno | PE | 1 | 62.784 | 0,02 | 0,0 |
| Recife | PE | 35 | 1.645.727 | 0,02 | 0,0 |
| Ribeirão | PE | 1 | 47.415 | 0,02 | 2,4 |
| Toritama | PE | 1 | 45.219 | 0,02 | 0,0 |
| Aracaju | SE | 12 | 657.013 | 0,02 | 0,0 |
| Simões Filho | BA | 3 | 134.377 | 0,02 | 0,0 |
| Andradas | MG | 1 | 41.077 | 0,02 | 0,0 |
| Betim | MG | 8 | 439.340 | 0,02 | 0,0 |
| Leopoldina | MG | 1 | 52.587 | 0,02 | 0,0 |
| Ribeirão das Neves | MG | 6 | 334.858 | 0,02 | 0,0 |
| Vespasiano | MG | 3 | 127.601 | 0,02 | 0,0 |
| Visconde do Rio Branco | MG | 1 | 42.564 | 0,02 | 0,0 |
| Cariacica | ES | 7 | 381.285 | 0,02 | 0,0 |
| Campos dos Goytacazes | RJ | 9 | 507.548 | 0,02 | 0,0 |
| Itaboraí | RJ | 6 | 240.592 | 0,02 | 0,0 |
| Nova Iguaçu | RJ | 16 | 821.128 | 0,02 | 0,0 |
| Rio das Ostras | RJ | 3 | 150.674 | 0,02 | 0,0 |
| Andradina | SP | 1 | 57.157 | 0,02 | 0,0 |
| Avaré | SP | 2 | 90.655 | 0,02 | 0,0 |
| Bauru | SP | 9 | 376.818 | 0,02 | 0,0 |
| Campos do Jordão | SP | 1 | 52.088 | 0,02 | 0,0 |
| Dracena | SP | 1 | 46.793 | 0,02 | 0,0 |
| Embu das Artes | SP | 5 | 273.726 | 0,02 | 0,0 |
| Ferraz de Vasconcelos | SP | 3 | 194.276 | 0,02 | 0,0 |
| Guaíra | SP | 1 | 40.790 | 0,02 | 0,2 |
| Itapevi | SP | 4 | 237.700 | 0,02 | 0,0 |
| Itaquaquecetuba | SP | 9 | 370.821 | 0,02 | 0,0 |
| Jandira | SP | 2 | 124.937 | 0,02 | 0,0 |
| Jundiaí | SP | 8 | 418.962 | 0,02 | 0,0 |
| Marília | SP | 4 | 238.882 | 0,02 | 0,0 |
| Monte Alto | SP | 1 | 50.498 | 0,02 | 0,0 |
| Monte Mor | SP | 1 | 59.772 | 0,02 | 0,0 |
| Pedreira | SP | 1 | 47.919 | 0,02 | 0,0 |
| Praia Grande | SP | 8 | 325.073 | 0,02 | 0,0 |
| Presidente Epitácio | SP | 1 | 44.200 | 0,02 | 0,1 |
| Ribeirão Preto | SP | 15 | 703.293 | 0,02 | 0,0 |
| Rio Claro | SP | 4 | 206.424 | 0,02 | 0,0 |
| Salto | SP | 2 | 118.663 | 0,02 | 0,0 |
| Santana de Parnaíba | SP | 3 | 139.447 | 0,02 | 0,0 |
| Santos | SP | 7 | 433.311 | 0,02 | 0,0 |
| São Bernardo do Campo | SP | 17 | 838.936 | 0,02 | 0,0 |
| São Carlos | SP | 5 | 251.983 | 0,02 | 0,0 |
| São Joaquim da Barra | SP | 1 | 51.888 | 0,02 | 0,0 |
| São Manuel | SP | 1 | 40.954 | 0,02 | 0,0 |
| São Vicente | SP | 8 | 365.798 | 0,02 | 0,0 |
| Sertãozinho | SP | 3 | 125.815 | 0,02 | 0,0 |
| Tietê | SP | 1 | 42.076 | 0,02 | 0,0 |
| Vargem Grande do Sul | SP | 1 | 42.845 | 0,02 | 0,0 |
| Várzea Paulista | SP | 2 | 121.838 | 0,02 | 0,0 |
| Araucária | PR | 3 | 143.843 | 0,02 | 0,0 |
| Cambé | PR | 2 | 106.533 | 0,02 | 0,0 |
| Campo Mourão | PR | 2 | 94.859 | 0,02 | 0,0 |
| Cianorte | PR | 2 | 82.620 | 0,02 | 0,0 |
| Fazenda Rio Grande | PR | 2 | 100.209 | 0,02 | 0,0 |
| Palmas | PR | 1 | 50.986 | 0,02 | 0,6 |
| Pinhais | PR | 2 | 132.157 | 0,02 | 0,0 |
| Camboriú | SC | 2 | 82.989 | 0,02 | 0,0 |
| Canoinhas | SC | 1 | 54.401 | 0,02 | 0,0 |
| Içara | SC | 1 | 56.421 | 0,02 | 0,0 |
| Itajaí | SC | 4 | 219.536 | 0,02 | 0,0 |
| Lages | SC | 3 | 157.544 | 0,02 | 0,1 |
| Laguna | SC | 1 | 45.814 | 0,02 | 0,0 |
| São Bento do Sul | SC | 2 | 84.507 | 0,02 | 0,0 |
| Xanxerê | SC | 1 | 50.982 | 0,02 | 0,0 |
| Bento Gonçalves | RS | 3 | 120.454 | 0,02 | 0,0 |
| Canela | RS | 1 | 44.998 | 0,02 | 0,3 |
| Eldorado do Sul | RS | 1 | 41.285 | 0,02 | 0,0 |
| Estância Velha | RS | 1 | 50.022 | 0,02 | 0,0 |
| Gravataí | RS | 6 | 281.519 | 0,02 | 0,0 |
| Lajeado | RS | 2 | 84.014 | 0,02 | 0,0 |
| Montenegro | RS | 1 | 65.264 | 0,02 | 0,0 |
| Passo Fundo | RS | 5 | 203.275 | 0,02 | 0,0 |
| São Leopoldo | RS | 4 | 236.835 | 0,02 | 0,0 |
| Sapiranga | RS | 2 | 81.734 | 0,02 | 0,0 |
| Taquara | RS | 1 | 57.466 | 0,02 | 0,0 |
| Viamão | RS | 5 | 255.224 | 0,02 | 0,0 |
| Paço do Lumiar | MA | 1 | 122.197 | 0,01 | 100,0 |
| São José de Ribamar | MA | 2 | 177.687 | 0,01 | 100,0 |
| São Luís | MA | 13 | 1.101.884 | 0,01 | 85,8 |
| Fortaleza | CE | 32 | 2.669.342 | 0,01 | 0,0 |
| Maracanaú | CE | 3 | 227.886 | 0,01 | 0,0 |
| Cabedelo | PB | 1 | 67.736 | 0,01 | 0,0 |
| Candeias | BA | 1 | 87.076 | 0,01 | 0,0 |
| Dias d'Ávila | BA | 1 | 81.089 | 0,01 | 0,0 |
| Lauro de Freitas | BA | 1 | 198.440 | 0,01 | 0,0 |
| Belo Horizonte | MG | 28 | 2.512.070 | 0,01 | 0,0 |
| Contagem | MG | 9 | 663.855 | 0,01 | 0,0 |
| João Monlevade | MG | 1 | 79.910 | 0,01 | 0,0 |
| Sabará | MG | 2 | 136.344 | 0,01 | 0,0 |
| Santa Luzia | MG | 3 | 219.134 | 0,01 | 0,0 |
| Serra | ES | 6 | 517.510 | 0,01 | 0,0 |
| Barra Mansa | RJ | 2 | 184.412 | 0,01 | 0,0 |
| Duque de Caxias | RJ | 9 | 919.596 | 0,01 | 0,0 |
| Nilópolis | RJ | 2 | 162.485 | 0,01 | 0,0 |
| Rio de Janeiro | RJ | 56 | 6.718.903 | 0,01 | 0,0 |
| São Gonçalo | RJ | 6 | 1.084.839 | 0,01 | 0,0 |
| São Pedro da Aldeia | RJ | 1 | 104.476 | 0,01 | 0,0 |
| Americana | SP | 3 | 239.597 | 0,01 | 0,0 |
| Araraquara | SP | 3 | 236.072 | 0,01 | 0,0 |
| Araras | SP | 2 | 134.236 | 0,01 | 0,0 |
| Barueri | SP | 2 | 274.182 | 0,01 | 0,0 |
| Campinas | SP | 12 | 1.204.073 | 0,01 | 0,0 |
| Carapicuíba | SP | 4 | 400.927 | 0,01 | 0,0 |
| Diadema | SP | 5 | 423.884 | 0,01 | 0,0 |
| Embu-Guaçu | SP | 1 | 69.385 | 0,01 | 0,0 |
| Franca | SP | 5 | 353.187 | 0,01 | 0,0 |
| Franco da Rocha | SP | 2 | 154.489 | 0,01 | 0,0 |
| Guarulhos | SP | 9 | 1.379.182 | 0,01 | 0,0 |
| Jaú | SP | 2 | 150.252 | 0,01 | 0,0 |
| Lençóis Paulista | SP | 1 | 68.432 | 0,01 | 0,0 |
| Osasco | SP | 10 | 698.418 | 0,01 | 0,0 |
| Paulínia | SP | 1 | 109.424 | 0,01 | 0,0 |
| Presidente Prudente | SP | 2 | 228.743 | 0,01 | 0,0 |
| Santa Bárbara d'Oeste | SP | 1 | 193.475 | 0,01 | 0,0 |
| Santo André | SP | 10 | 718.773 | 0,01 | 0,0 |
| São Caetano do Sul | SP | 1 | 161.127 | 0,01 | 0,0 |
| São Paulo | SP | 70 | 12.252.023 | 0,01 | 0,0 |
| Sorocaba | SP | 9 | 679.378 | 0,01 | 0,0 |
| Sumaré | SP | 3 | 282.441 | 0,01 | 0,0 |
| Taboão da Serra | SP | 2 | 289.664 | 0,01 | 0,0 |
| Valinhos | SP | 1 | 129.193 | 0,01 | 0,0 |
| Curitiba | PR | 11 | 1.933.105 | 0,01 | 0,0 |
| Londrina | PR | 8 | 569.733 | 0,01 | 0,0 |
| Pato Branco | PR | 1 | 82.881 | 0,01 | 0,0 |
| São José dos Pinhais | PR | 4 | 323.340 | 0,01 | 0,0 |
| Sarandi | PR | 1 | 96.688 | 0,01 | 0,0 |
| Alvorada | RS | 3 | 210.305 | 0,01 | 0,0 |
| Cachoeirinha | RS | 1 | 130.293 | 0,01 | 0,0 |
| Campo Bom | RS | 1 | 66.712 | 0,01 | 0,0 |
| Erechim | RS | 1 | 105.862 | 0,01 | 0,0 |
| Esteio | RS | 1 | 83.202 | 0,01 | 0,0 |
| Guaíba | RS | 1 | 98.143 | 0,01 | 0,0 |
| Novo Hamburgo | RS | 2 | 246.748 | 0,01 | 0,0 |
| Porto Alegre | RS | 13 | 1.483.771 | 0,01 | 0,0 |
| Sapucaia do Sul | RS | 2 | 141.075 | 0,01 | 0,0 |
| Rio Crespo | RO | 0 | 3.764 | 0 | 36,6 |
| Teixeirópolis | RO | 0 | 4.308 | 0 | 100,0 |
| Brejo Grande do Araguaia | PA | 0 | 7.380 | 0 | 1,5 |
| Curionópolis | PA | 0 | 17.929 | 0 | 0,1 |
| Palestina do Pará | PA | 0 | 7.589 | 0 | 9,7 |
| Sapucaia | PA | 0 | 5.930 | 0 | 9,8 |
| Bom Jesus do Tocantins | TO | 0 | 4.894 | 0 | 8,2 |
| Cariri do Tocantins | TO | 0 | 4.382 | 0 | 0,2 |
| Crixás do Tocantins | TO | 0 | 1.722 | 0 | 0,0 |
| Jaú do Tocantins | TO | 0 | 3.849 | 0 | 10,7 |
| Juarina | TO | 0 | 2.193 | 0 | 13,5 |
| Novo Alegre | TO | 0 | 2.332 | 0 | 0,0 |
| Novo Jardim | TO | 0 | 2.722 | 0 | 1,3 |
| Porto Alegre do Tocantins | TO | 0 | 3.139 | 0 | 0,0 |
| Presidente Kennedy | TO | 0 | 3.684 | 0 | 1,0 |
| São Sebastião do Tocantins | TO | 0 | 4.805 | 0 | 0,0 |
| Taipas do Tocantins | TO | 0 | 2.148 | 0 | 6,3 |
| Tupirama | TO | 0 | 1.891 | 0 | 8,7 |
| Tupiratins | TO | 0 | 2.671 | 0 | 39,1 |
| Alcântara | MA | 0 | 22.097 | 0 | 6,5 |
| Axixá | MA | 0 | 12.130 | 0 | 0,0 |
| Belágua | MA | 0 | 7.469 | 0 | 5,0 |
| Bom Lugar | MA | 0 | 16.294 | 0 | 0,0 |
| Cedral | MA | 0 | 10.675 | 0 | 0,0 |
| Humberto de Campos | MA | 0 | 28.717 | 0 | 12,3 |
| Luís Domingues | MA | 0 | 6.951 | 0 | 100,0 |
| Magalhães de Almeida | MA | 0 | 19.826 | 0 | 0,0 |
| Porto Rico do Maranhão | MA | 0 | 5.975 | 0 | 0,0 |
| Primeira Cruz | MA | 0 | 15.315 | 0 | 7,0 |
| Santa Luzia do Paruá | MA | 0 | 25.254 | 0 | 100,0 |
| Santo Antônio dos Lopes | MA | 0 | 14.528 | 0 | 4,6 |
| São Domingos do Azeitão | MA | 0 | 7.392 | 0 | 4,7 |
| São José dos Basílios | MA | 0 | 7.641 | 0 | 0,0 |
| Turilândia | MA | 0 | 25.619 | 0 | 20,0 |
| Acauã | PI | 0 | 7.084 | 0 | 24,3 |
| Agricolândia | PI | 0 | 5.139 | 0 | 0,0 |
| Alegrete do Piauí | PI | 0 | 4.915 | 0 | 23,5 |
| Amarante | PI | 0 | 17.598 | 0 | 0,0 |
| Angical do Piauí | PI | 0 | 6.788 | 0 | 0,7 |
| Anísio de Abreu | PI | 0 | 9.880 | 0 | 0,0 |
| Antônio Almeida | PI | 0 | 3.164 | 0 | 6,9 |
| Arraial | PI | 0 | 4.727 | 0 | 6,4 |
| Assunção do Piauí | PI | 0 | 7.846 | 0 | 4,1 |
| Barra D'Alcântara | PI | 0 | 3.951 | 0 | 0,9 |
| Barreiras do Piauí | PI | 0 | 3.348 | 0 | 100,0 |
| Barro Duro | PI | 0 | 7.032 | 0 | 0,3 |
| Bela Vista do Piauí | PI | 0 | 4.015 | 0 | 19,1 |
| Beneditinos | PI | 0 | 10.467 | 0 | 5,2 |
| Bom Princípio do Piauí | PI | 0 | 5.630 | 0 | 3,0 |
| Bonfim do Piauí | PI | 0 | 5.670 | 0 | 0,0 |
| Boqueirão do Piauí | PI | 0 | 6.407 | 0 | 0,0 |
| Brasileira | PI | 0 | 8.329 | 0 | 5,9 |
| Brejo do Piauí | PI | 0 | 3.875 | 0 | 41,2 |
| Cajazeiras do Piauí | PI | 0 | 3.559 | 0 | 24,1 |
| Cajueiro da Praia | PI | 0 | 7.642 | 0 | 0,0 |
| Campo Alegre do Fidalgo | PI | 0 | 5.045 | 0 | 25,9 |
| Campo Maior | PI | 0 | 46.833 | 0 | 1,2 |
| Canavieira | PI | 0 | 3.950 | 0 | 99,0 |
| Canto do Buriti | PI | 0 | 21.187 | 0 | 34,2 |
| Capitão de Campos | PI | 0 | 11.417 | 0 | 5,8 |
| Capitão Gervásio Oliveira | PI | 0 | 4.100 | 0 | 25,8 |
| Caracol | PI | 0 | 10.916 | 0 | 1,3 |
| Cocal de Telha | PI | 0 | 4.891 | 0 | 1,7 |
| Conceição do Canindé | PI | 0 | 4.803 | 0 | 10,9 |
| Coronel José Dias | PI | 0 | 4.682 | 0 | 37,8 |
| Cristalândia do Piauí | PI | 0 | 8.294 | 0 | 31,3 |
| Demerval Lobão | PI | 0 | 13.817 | 0 | 0,0 |
| Domingos Mourão | PI | 0 | 4.355 | 0 | 21,5 |
| Eliseu Martins | PI | 0 | 4.915 | 0 | 100,0 |
| Floresta do Piauí | PI | 0 | 2.558 | 0 | 15,1 |
| Francinópolis | PI | 0 | 5.348 | 0 | 11,6 |
| Francisco Macedo | PI | 0 | 3.184 | 0 | 14,0 |
| Guaribas | PI | 0 | 4.562 | 0 | 92,7 |
| Hugo Napoleão | PI | 0 | 3.877 | 0 | 3,9 |
| Isaías Coelho | PI | 0 | 8.549 | 0 | 49,0 |
| Jaicós | PI | 0 | 19.104 | 0 | 14,3 |
| Jardim do Mulato | PI | 0 | 4.504 | 0 | 5,4 |
| Jerumenha | PI | 0 | 4.452 | 0 | 14,3 |
| João Costa | PI | 0 | 3.008 | 0 | 7,9 |
| Joca Marques | PI | 0 | 5.443 | 0 | 0,0 |
| Jurema | PI | 0 | 4.763 | 0 | 6,1 |
| Lagoa Alegre | PI | 0 | 8.542 | 0 | 0,0 |
| Lagoa do Sítio | PI | 0 | 5.177 | 0 | 19,0 |
| Madeiro | PI | 0 | 8.310 | 0 | 0,0 |
| Manoel Emídio | PI | 0 | 5.349 | 0 | 100,0 |
| Marcolândia | PI | 0 | 8.492 | 0 | 6,3 |
| Marcos Parente | PI | 0 | 4.549 | 0 | 4,3 |
| Matias Olímpio | PI | 0 | 10.936 | 0 | 0,0 |
| Miguel Leão | PI | 0 | 1.246 | 0 | 0,0 |
| Morro Cabeça no Tempo | PI | 0 | 4.532 | 0 | 100,0 |
| Murici dos Portelas | PI | 0 | 9.159 | 0 | 0,0 |
| Nossa Senhora de Nazaré | PI | 0 | 4.870 | 0 | 0,0 |
| Olho D'Água do Piauí | PI | 0 | 2.459 | 0 | 0,0 |
| Paes Landim | PI | 0 | 4.129 | 0 | 99,0 |
| Passagem Franca do Piauí | PI | 0 | 4.313 | 0 | 18,2 |
| Paulistana | PI | 0 | 20.523 | 0 | 9,5 |
| Pavussu | PI | 0 | 3.677 | 0 | 100,0 |
| Pedro Laurentino | PI | 0 | 2.536 | 0 | 2,3 |
| Nova Santa Rita | PI | 0 | 4.374 | 0 | 26,9 |
| Piracuruca | PI | 0 | 28.791 | 0 | 9,9 |
| Porto Alegre do Piauí | PI | 0 | 2.710 | 0 | 17,7 |
| Prata do Piauí | PI | 0 | 3.151 | 0 | 0,2 |
| Regeneração | PI | 0 | 17.978 | 0 | 2,5 |
| Riacho Frio | PI | 0 | 4.312 | 0 | 96,6 |
| Ribeira do Piauí | PI | 0 | 4.477 | 0 | 75,7 |
| Rio Grande do Piauí | PI | 0 | 6.432 | 0 | 100,0 |
| Santa Rosa do Piauí | PI | 0 | 5.257 | 0 | 1,7 |
| Santo Antônio dos Milagres | PI | 0 | 2.161 | 0 | 0,0 |
| São Braz do Piauí | PI | 0 | 4.448 | 0 | 0,5 |
| São Francisco de Assis do Piauí | PI | 0 | 5.755 | 0 | 36,2 |
| São Francisco do Piauí | PI | 0 | 6.423 | 0 | 33,8 |
| São Gonçalo do Gurguéia | PI | 0 | 3.041 | 0 | 100,0 |
| São Gonçalo do Piauí | PI | 0 | 5.015 | 0 | 0,0 |
| São João da Canabrava | PI | 0 | 4.608 | 0 | 3,9 |
| São João da Fronteira | PI | 0 | 6.042 | 0 | 32,5 |
| São José do Divino | PI | 0 | 5.346 | 0 | 12,3 |
| São José do Peixe | PI | 0 | 3.745 | 0 | 99,3 |
| São Julião | PI | 0 | 6.363 | 0 | 15,6 |
| São Luis do Piauí | PI | 0 | 2.644 | 0 | 3,3 |
| São Miguel da Baixa Grande | PI | 0 | 2.452 | 0 | 18,1 |
| São Miguel do Fidalgo | PI | 0 | 3.039 | 0 | 100,0 |
| Sebastião Barros | PI | 0 | 3.469 | 0 | 61,5 |
| Sigefredo Pacheco | PI | 0 | 10.041 | 0 | 20,2 |
| Socorro do Piauí | PI | 0 | 4.569 | 0 | 100,0 |
| Sussuapara | PI | 0 | 6.730 | 0 | 0,0 |
| Tamboril do Piauí | PI | 0 | 2.919 | 0 | 33,2 |
| Várzea Branca | PI | 0 | 4.947 | 0 | 1,4 |
| Várzea Grande | PI | 0 | 4.391 | 0 | 3,6 |
| Vila Nova do Piauí | PI | 0 | 2.971 | 0 | 25,1 |
| Abaiara | CE | 0 | 11.737 | 0 | 3,4 |
| Acaraú | CE | 0 | 62.641 | 0 | 53,9 |
| Alcântaras | CE | 0 | 11.714 | 0 | 0,0 |
| Apuiarés | CE | 0 | 14.600 | 0 | 89,6 |
| Baixio | CE | 0 | 6.288 | 0 | 99,9 |
| Barroquinha | CE | 0 | 15.017 | 0 | 7,7 |
| Cariré | CE | 0 | 18.448 | 0 | 0,0 |
| Catunda | CE | 0 | 10.342 | 0 | 96,0 |
| Forquilha | CE | 0 | 24.218 | 0 | 0,0 |
| General Sampaio | CE | 0 | 7.618 | 0 | 100,0 |
| Groaíras | CE | 0 | 11.068 | 0 | 0,0 |
| Hidrolândia | CE | 0 | 19.978 | 0 | 99,8 |
| Ipaporanga | CE | 0 | 11.593 | 0 | 100,0 |
| Ipaumirim | CE | 0 | 12.463 | 0 | 97,7 |
| Meruoca | CE | 0 | 15.057 | 0 | 0,0 |
| Miraíma | CE | 0 | 13.818 | 0 | 11,2 |
| Moraújo | CE | 0 | 8.724 | 0 | 0,5 |
| Pacatuba | CE | 0 | 83.432 | 0 | 0,0 |
| Palmácia | CE | 0 | 13.322 | 0 | 0,0 |
| Penaforte | CE | 0 | 9.077 | 0 | 1,7 |
| Piquet Carneiro | CE | 0 | 16.959 | 0 | 100,0 |
| Reriutaba | CE | 0 | 18.491 | 0 | 10,5 |
| Santana do Acaraú | CE | 0 | 32.452 | 0 | 2,1 |
| São Luís do Curu | CE | 0 | 13.000 | 0 | 1,4 |
| Senador Pompeu | CE | 0 | 25.496 | 0 | 38,4 |
| Tarrafas | CE | 0 | 8.592 | 0 | 99,7 |
| Tejuçuoca | CE | 0 | 19.187 | 0 | 100,0 |
| Umari | CE | 0 | 7.733 | 0 | 100,0 |
| Varjota | CE | 0 | 18.420 | 0 | 0,1 |
| Baía Formosa | RN | 0 | 9.271 | 0 | 0,2 |
| Bodó | RN | 0 | 2.223 | 0 | 12,9 |
| Bom Jesus | RN | 0 | 10.210 | 0 | 0,0 |
| Caiçara do Norte | RN | 0 | 6.549 | 0 | 1,6 |
| Coronel João Pessoa | RN | 0 | 4.912 | 0 | 100,0 |
| Doutor Severiano | RN | 0 | 7.076 | 0 | 100,0 |
| Encanto | RN | 0 | 5.638 | 0 | 100,0 |
| ES | RN | 0 | 10.505 | 0 | 0,0 |
| Ipueira | RN | 0 | 2.241 | 0 | 0,0 |
| Jandaíra | RN | 0 | 6.878 | 0 | 0,3 |
| Jardim de Angicos | RN | 0 | 2.612 | 0 | 0,3 |
| João Dias | RN | 0 | 2.654 | 0 | 27,7 |
| Lagoa de Velhos | RN | 0 | 2.732 | 0 | 0,0 |
| Lagoa Salgada | RN | 0 | 8.245 | 0 | 0,0 |
| Lucrécia | RN | 0 | 3.996 | 0 | 95,9 |
| Luís Gomes | RN | 0 | 10.116 | 0 | 100,0 |
| Marcelino Vieira | RN | 0 | 8.347 | 0 | 100,0 |
| Martins | RN | 0 | 8.725 | 0 | 100,0 |
| Montanhas | RN | 0 | 11.251 | 0 | 6,0 |
| Passagem | RN | 0 | 3.089 | 0 | 0,0 |
| Santa Maria | RN | 0 | 5.551 | 0 | 7,6 |
| Pedra Grande | RN | 0 | 3.237 | 0 | 0,0 |
| Pilões | RN | 0 | 3.838 | 0 | 100,0 |
| Riacho da Cruz | RN | 0 | 3.579 | 0 | 99,9 |
| Riachuelo | RN | 0 | 8.128 | 0 | 0,0 |
| São Fernando | RN | 0 | 3.584 | 0 | 16,1 |
| São Francisco do Oeste | RN | 0 | 4.228 | 0 | 100,0 |
| Senador Georgino Avelino | RN | 0 | 4.440 | 0 | 0,0 |
| Serra Negra do Norte | RN | 0 | 8.078 | 0 | 4,1 |
| Serrinha | RN | 0 | 6.229 | 0 | 11,8 |
| Tibau do Sul | RN | 0 | 14.180 | 0 | 0,0 |
| Várzea | RN | 0 | 5.500 | 0 | 2,3 |
| Vera Cruz | RN | 0 | 12.481 | 0 | 0,0 |
| Viçosa | RN | 0 | 1.718 | 0 | 100,0 |
| Vila Flor | RN | 0 | 3.170 | 0 | 0,0 |
| Alagoa Nova | PB | 0 | 20.849 | 0 | 4,5 |
| Alagoinha | PB | 0 | 14.489 | 0 | 0,0 |
| Aparecida | PB | 0 | 8.347 | 0 | 100,0 |
| Areial | PB | 0 | 6.998 | 0 | 3,4 |
| Bernardino Batista | PB | 0 | 3.501 | 0 | 100,0 |
| Bom Jesus | PB | 0 | 2.561 | 0 | 100,0 |
| Igaracy | PB | 0 | 6.117 | 0 | 100,0 |
| Brejo dos Santos | PB | 0 | 6.449 | 0 | 6,3 |
| Cabaceiras | PB | 0 | 5.611 | 0 | 100,0 |
| Cachoeira dos Índios | PB | 0 | 10.244 | 0 | 91,3 |
| Cacimba de Areia | PB | 0 | 3.682 | 0 | 43,0 |
| Caiçara | PB | 0 | 7.201 | 0 | 21,8 |
| Caldas Brandão | PB | 0 | 6.014 | 0 | 0,0 |
| Capim | PB | 0 | 6.523 | 0 | 0,3 |
| Carrapateira | PB | 0 | 2.659 | 0 | 100,0 |
| Coxixola | PB | 0 | 1.921 | 0 | 100,0 |
| Cuité de Mamanguape | PB | 0 | 6.353 | 0 | 26,9 |
| Duas Estradas | PB | 0 | 3.596 | 0 | 7,8 |
| Lagoa | PB | 0 | 4.666 | 0 | 100,0 |
| Lagoa de Dentro | PB | 0 | 7.719 | 0 | 24,5 |
| Logradouro | PB | 0 | 4.332 | 0 | 4,1 |
| Mari | PB | 0 | 21.837 | 0 | 2,0 |
| Marizópolis | PB | 0 | 6.617 | 0 | 100,0 |
| Nazarezinho | PB | 0 | 7.301 | 0 | 100,0 |
| Parari | PB | 0 | 1.771 | 0 | 100,0 |
| Pedra Branca | PB | 0 | 3.801 | 0 | 2,7 |
| Pilar | PB | 0 | 11.917 | 0 | 0,0 |
| Riachão do Bacamarte | PB | 0 | 4.521 | 0 | 0,0 |
| Riachão do Poço | PB | 0 | 4.509 | 0 | 0,0 |
| Riacho dos Cavalos | PB | 0 | 8.526 | 0 | 30,4 |
| Santa Cruz | PB | 0 | 6.583 | 0 | 100,0 |
| Santa Inês | PB | 0 | 3.595 | 0 | 38,1 |
| Joca Claudino | PB | 0 | 2.636 | 0 | 100,0 |
| Santa Teresinha | PB | 0 | 4.573 | 0 | 96,1 |
| Santo André | PB | 0 | 2.521 | 0 | 100,0 |
| São Bento | PB | 0 | 34.031 | 0 | 0,4 |
| São Bentinho | PB | 0 | 4.529 | 0 | 45,0 |
| São Domingos | PB | 0 | 3.073 | 0 | 100,0 |
| São Francisco | PB | 0 | 3.392 | 0 | 100,0 |
| São José da Lagoa Tapada | PB | 0 | 7.630 | 0 | 100,0 |
| São José dos Ramos | PB | 0 | 5.957 | 0 | 0,0 |
| São José de Princesa | PB | 0 | 4.003 | 0 | 0,0 |
| São José do Bonfim | PB | 0 | 3.557 | 0 | 95,1 |
| São José do Sabugi | PB | 0 | 4.141 | 0 | 9,0 |
| São Miguel de Taipu | PB | 0 | 7.368 | 0 | 0,0 |
| São Sebastião de Lagoa de Roça | PB | 0 | 11.661 | 0 | 0,0 |
| Serra da Raiz | PB | 0 | 3.148 | 0 | 6,0 |
| Serra Grande | PB | 0 | 2.909 | 0 | 100,0 |
| Serraria | PB | 0 | 6.099 | 0 | 20,5 |
| Sertãozinho | PB | 0 | 5.024 | 0 | 2,1 |
| Sobrado | PB | 0 | 7.783 | 0 | 0,0 |
| Tacima | PB | 0 | 10.911 | 0 | 17,7 |
| Tenório | PB | 0 | 3.058 | 0 | 17,9 |
| Triunfo | PB | 0 | 9.455 | 0 | 100,0 |
| Uiraúna | PB | 0 | 15.242 | 0 | 100,0 |
| Vieirópolis | PB | 0 | 5.348 | 0 | 100,0 |
| Barra de Guabiraba | PE | 0 | 14.385 | 0 | 1,9 |
| Cachoeirinha | PE | 0 | 20.380 | 0 | 0,0 |
| Camocim de São Félix | PE | 0 | 18.765 | 0 | 0,0 |
| Camutanga | PE | 0 | 8.551 | 0 | 0,0 |
| Cupira | PE | 0 | 24.107 | 0 | 0,0 |
| Fernando de Noronha | PE | 0 | 3.061 | 0 |  |
| Ferreiros | PE | 0 | 12.123 | 0 | 0,0 |
| Frei Miguelinho | PE | 0 | 15.457 | 0 | 10,3 |
| Ilha de Itamaracá | PE | 0 | 26.258 | 0 | 0,0 |
| Jucati | PE | 0 | 11.424 | 0 | 6,2 |
| Jupi | PE | 0 | 14.836 | 0 | 4,6 |
| Lagoa de Itaenga | PE | 0 | 21.429 | 0 | 0,0 |
| Maraial | PE | 0 | 11.345 | 0 | 38,1 |
| Palmeirina | PE | 0 | 7.693 | 0 | 11,6 |
| Quixaba | PE | 0 | 6.813 | 0 | 0,0 |
| Salgadinho | PE | 0 | 10.919 | 0 | 0,0 |
| Saloá | PE | 0 | 15.843 | 0 | 3,0 |
| Santa Maria do Cambucá | PE | 0 | 14.137 | 0 | 5,4 |
| São Vicente Ferrer | PE | 0 | 18.018 | 0 | 0,3 |
| Tracunhaém | PE | 0 | 13.769 | 0 | 0,0 |
| Vertente do Lério | PE | 0 | 7.618 | 0 | 24,1 |
| Belém | AL | 0 | 4.344 | 0 | 0,0 |
| Canapi | AL | 0 | 17.722 | 0 | 3,4 |
| Coité do Nóia | AL | 0 | 10.693 | 0 | 0,0 |
| Coqueiro Seco | AL | 0 | 5.845 | 0 | 0,0 |
| Dois Riachos | AL | 0 | 11.075 | 0 | 0,0 |
| Estrela de Alagoas | AL | 0 | 18.205 | 0 | 0,0 |
| Jacaré dos Homens | AL | 0 | 5.253 | 0 | 0,0 |
| Japaratinga | AL | 0 | 8.361 | 0 | 67,8 |
| Jaramataia | AL | 0 | 5.770 | 0 | 0,0 |
| Major Isidoro | AL | 0 | 19.804 | 0 | 0,0 |
| Mar Vermelho | AL | 0 | 3.514 | 0 | 0,0 |
| Mata Grande | AL | 0 | 25.216 | 0 | 0,6 |
| Minador do Negrão | AL | 0 | 5.329 | 0 | 0,0 |
| Monteirópolis | AL | 0 | 7.159 | 0 | 0,0 |
| Olho d'Água Grande | AL | 0 | 5.123 | 0 | 0,0 |
| Olivença | AL | 0 | 11.624 | 0 | 0,0 |
| Ouro Branco | AL | 0 | 11.496 | 0 | 4,4 |
| Palestina | AL | 0 | 5.011 | 0 | 0,0 |
| Pindoba | AL | 0 | 2.908 | 0 | 0,0 |
| Poço das Trincheiras | AL | 0 | 14.386 | 0 | 0,7 |
| Porto Real do Colégio | AL | 0 | 20.066 | 0 | 0,0 |
| São Brás | AL | 0 | 6.961 | 0 | 0,0 |
| Senador Rui Palmeira | AL | 0 | 13.870 | 0 | 0,0 |
| Amparo de São Francisco | SE | 0 | 2.374 | 0 | 0,0 |
| Boquim | SE | 0 | 26.816 | 0 | 0,0 |
| Campo do Brito | SE | 0 | 18.109 | 0 | 0,0 |
| Cedro de São João | SE | 0 | 5.897 | 0 | 0,0 |
| Cumbe | SE | 0 | 3.987 | 0 | 0,0 |
| Divina Pastora | SE | 0 | 5.138 | 0 | 0,0 |
| Feira Nova | SE | 0 | 5.584 | 0 | 0,0 |
| General Maynard | SE | 0 | 3.346 | 0 | 0,0 |
| Gracho Cardoso | SE | 0 | 5.818 | 0 | 0,0 |
| Malhada dos Bois | SE | 0 | 3.682 | 0 | 0,0 |
| Moita Bonita | SE | 0 | 11.335 | 0 | 0,0 |
| Muribeca | SE | 0 | 7.625 | 0 | 0,0 |
| Nossa Senhora Aparecida | SE | 0 | 8.796 | 0 | 0,2 |
| Nossa Senhora da Glória | SE | 0 | 36.924 | 0 | 0,0 |
| Pedra Mole | SE | 0 | 3.261 | 0 | 0,0 |
| Porto da Folha | SE | 0 | 28.596 | 0 | 0,0 |
| Ribeirópolis | SE | 0 | 18.652 | 0 | 0,0 |
| São Francisco | SE | 0 | 3.724 | 0 | 0,0 |
| São Miguel do Aleixo | SE | 0 | 3.930 | 0 | 0,0 |
| Telha | SE | 0 | 3.227 | 0 | 0,0 |
| Tobias Barreto | SE | 0 | 52.191 | 0 | 0,0 |
| Adustina | BA | 0 | 17.040 | 0 | 6,7 |
| Antas | BA | 0 | 19.291 | 0 | 0,0 |
| Barro Alto | BA | 0 | 14.931 | 0 | 8,3 |
| Barrocas | BA | 0 | 15.978 | 0 | 0,0 |
| Brejolândia | BA | 0 | 10.557 | 0 | 10,3 |
| Caém | BA | 0 | 9.213 | 0 | 0,0 |
| Camaçari | BA | 1 | 299.132 | 0 | 0,2 |
| Canápolis | BA | 0 | 9.711 | 0 | 0,0 |
| Candeal | BA | 0 | 8.258 | 0 | 0,4 |
| Capela do Alto Alegre | BA | 0 | 11.637 | 0 | 2,9 |
| Catolândia | BA | 0 | 3.577 | 0 | 3,9 |
| Central | BA | 0 | 17.266 | 0 | 0,1 |
| Cipó | BA | 0 | 17.300 | 0 | 0,0 |
| Contendas do Sincorá | BA | 0 | 4.066 | 0 | 13,5 |
| Curaçá | BA | 0 | 34.700 | 0 | 11,6 |
| Feira da Mata | BA | 0 | 5.665 | 0 | 16,7 |
| Firmino Alves | BA | 0 | 5.617 | 0 | 0,0 |
| Gavião | BA | 0 | 4.463 | 0 | 3,8 |
| Heliópolis | BA | 0 | 13.031 | 0 | 0,0 |
| Ibiassucê | BA | 0 | 9.224 | 0 | 0,0 |
| Ichu | BA | 0 | 6.206 | 0 | 0,0 |
| Irajuba | BA | 0 | 7.260 | 0 | 12,2 |
| Iramaia | BA | 0 | 8.537 | 0 | 17,4 |
| Iuiú | BA | 0 | 10.994 | 0 | 1,7 |
| Jacaraci | BA | 0 | 14.842 | 0 | 0,9 |
| Lajedão | BA | 0 | 3.955 | 0 | 1,1 |
| Lajedo do Tabocal | BA | 0 | 8.562 | 0 | 5,6 |
| Madre de Deus | BA | 0 | 21.093 | 0 | 0,0 |
| Mansidão | BA | 0 | 13.643 | 0 | 19,8 |
| Marcionílio Souza | BA | 0 | 10.406 | 0 | 4,3 |
| Mortugaba | BA | 0 | 12.042 | 0 | 0,3 |
| Muquém de São Francisco | BA | 0 | 11.348 | 0 | 4,2 |
| Nordestina | BA | 0 | 13.130 | 0 | 4,5 |
| Nova Fátima | BA | 0 | 7.812 | 0 | 3,1 |
| Nova Itarana | BA | 0 | 8.226 | 0 | 0,0 |
| Nova Redenção | BA | 0 | 9.118 | 0 | 2,4 |
| Pé de Serra | BA | 0 | 13.578 | 0 | 7,3 |
| Piripá | BA | 0 | 10.707 | 0 | 4,7 |
| Piritiba | BA | 0 | 24.755 | 0 | 0,0 |
| Pojuca | BA | 0 | 39.519 | 0 | 0,0 |
| Quijingue | BA | 0 | 27.580 | 0 | 7,2 |
| Quixabeira | BA | 0 | 8.972 | 0 | 0,0 |
| Riachão do Jacuípe | BA | 0 | 33.436 | 0 | 3,7 |
| Rio de Contas | BA | 0 | 12.989 | 0 | 0,8 |
| Rio do Pires | BA | 0 | 11.655 | 0 | 13,2 |
| Salvador | BA | 7 | 2.872.347 | 0 | 0,0 |
| Santo Amaro | BA | 0 | 60.069 | 0 | 0,0 |
| São Domingos | BA | 0 | 9.058 | 0 | 0,1 |
| São Francisco do Conde | BA | 0 | 39.802 | 0 | 0,0 |
| São José do Jacuípe | BA | 0 | 10.462 | 0 | 0,0 |
| São Sebastião do Passé | BA | 0 | 44.300 | 0 | 0,0 |
| Sebastião Laranjeiras | BA | 0 | 11.434 | 0 | 8,3 |
| Serrinha | BA | 0 | 80.861 | 0 | 0,0 |
| Sítio do Quinto | BA | 0 | 9.986 | 0 | 1,1 |
| Sobradinho | BA | 0 | 23.191 | 0 | 0,9 |
| Tabocas do Brejo Velho | BA | 0 | 12.518 | 0 | 11,4 |
| Tanquinho | BA | 0 | 7.918 | 0 | 0,0 |
| Tucano | BA | 0 | 50.687 | 0 | 1,8 |
| Utinga | BA | 0 | 19.178 | 0 | 2,6 |
| Várzea do Poço | BA | 0 | 9.170 | 0 | 0,0 |
| Wagner | BA | 0 | 9.345 | 0 | 0,0 |
| Acaiaca | MG | 0 | 3.994 | 0 | 0,0 |
| Aguanil | MG | 0 | 4.486 | 0 | 0,0 |
| Albertina | MG | 0 | 3.007 | 0 | 0,0 |
| Aracitaba | MG | 0 | 2.063 | 0 | 1,1 |
| Araporã | MG | 0 | 6.869 | 0 | 0,0 |
| Argirita | MG | 0 | 2.727 | 0 | 0,0 |
| Ataléia | MG | 0 | 12.868 | 0 | 7,5 |
| Barão de Monte Alto | MG | 0 | 5.397 | 0 | 0,0 |
| Belmiro Braga | MG | 0 | 3.429 | 0 | 0,0 |
| Berizal | MG | 0 | 4.735 | 0 | 8,2 |
| Bicas | MG | 0 | 14.494 | 0 | 0,0 |
| Biquinhas | MG | 0 | 2.515 | 0 | 4,6 |
| Bom Jardim de Minas | MG | 0 | 6.474 | 0 | 0,0 |
| Bom Jesus do Amparo | MG | 0 | 6.083 | 0 | 0,0 |
| Buenópolis | MG | 0 | 10.365 | 0 | 26,7 |
| Bugre | MG | 0 | 3.982 | 0 | 0,0 |
| Cabeceira Grande | MG | 0 | 6.949 | 0 | 3,6 |
| Cachoeira da Prata | MG | 0 | 3.603 | 0 | 0,0 |
| Cambuí | MG | 0 | 29.551 | 0 | 0,0 |
| Cambuquira | MG | 0 | 12.814 | 0 | 0,0 |
| Cana Verde | MG | 0 | 5.603 | 0 | 0,1 |
| Capim Branco | MG | 0 | 9.754 | 0 | 0,0 |
| Caranaíba | MG | 0 | 3.183 | 0 | 0,0 |
| Carvalhópolis | MG | 0 | 3.579 | 0 | 0,0 |
| Carvalhos | MG | 0 | 4.478 | 0 | 1,1 |
| Casa Grande | MG | 0 | 2.257 | 0 | 0,0 |
| Cássia | MG | 0 | 17.740 | 0 | 0,6 |
| Catas Altas | MG | 0 | 5.376 | 0 | 0,0 |
| Caxambu | MG | 0 | 21.656 | 0 | 0,0 |
| Chiador | MG | 0 | 2.687 | 0 | 0,0 |
| Claraval | MG | 0 | 4.843 | 0 | 0,0 |
| Cônego Marinho | MG | 0 | 7.642 | 0 | 18,1 |
| Confins | MG | 0 | 6.730 | 0 | 0,0 |
| Congonhal | MG | 0 | 11.950 | 0 | 0,0 |
| Congonhas do Norte | MG | 0 | 5.045 | 0 | 78,1 |
| Cordislândia | MG | 0 | 3.538 | 0 | 0,0 |
| Córrego Danta | MG | 0 | 3.215 | 0 | 0,0 |
| Córrego do Bom Jesus | MG | 0 | 3.704 | 0 | 0,0 |
| Couto de Magalhães de Minas | MG | 0 | 4.410 | 0 | 0,1 |
| Cristália | MG | 0 | 5.971 | 0 | 6,8 |
| Cristiano Otoni | MG | 0 | 5.150 | 0 | 0,0 |
| Crucilândia | MG | 0 | 5.034 | 0 | 0,0 |
| Cuparaque | MG | 0 | 4.982 | 0 | 0,0 |
| Curral de Dentro | MG | 0 | 7.729 | 0 | 10,5 |
| Delfim Moreira | MG | 0 | 8.025 | 0 | 0,0 |
| Delfinópolis | MG | 0 | 7.114 | 0 | 5,3 |
| Descoberto | MG | 0 | 5.013 | 0 | 0,4 |
| Desterro de Entre Rios | MG | 0 | 7.243 | 0 | 0,0 |
| Divinópolis | MG | 0 | 238.230 | 0 | 0,0 |
| Divisa Alegre | MG | 0 | 6.786 | 0 | 0,0 |
| Dom Bosco | MG | 0 | 3.677 | 0 | 6,4 |
| Dores de Campos | MG | 0 | 10.153 | 0 | 0,0 |
| Doresópolis | MG | 0 | 1.527 | 0 | 0,0 |
| Douradoquara | MG | 0 | 1.908 | 0 | 98,0 |
| ES do Dourado | MG | 0 | 4.692 | 0 | 12,8 |
| Estrela do Indaiá | MG | 0 | 3.500 | 0 | 0,0 |
| Ewbank da Câmara | MG | 0 | 3.913 | 0 | 0,0 |
| Fama | MG | 0 | 2.377 | 0 | 0,0 |
| São Gonçalo do Rio Preto | MG | 0 | 3.167 | 0 | 1,6 |
| Felisburgo | MG | 0 | 7.457 | 0 | 4,6 |
| Felixlândia | MG | 0 | 15.336 | 0 | 2,0 |
| Fortaleza de Minas | MG | 0 | 4.412 | 0 | 0,0 |
| Franciscópolis | MG | 0 | 5.391 | 0 | 16,5 |
| Goianá | MG | 0 | 3.966 | 0 | 0,0 |
| Gouveia | MG | 0 | 11.825 | 0 | 0,1 |
| Grupiara | MG | 0 | 1.388 | 0 | 98,9 |
| Guaraciama | MG | 0 | 4.972 | 0 | 3,6 |
| Heliodora | MG | 0 | 6.558 | 0 | 0,0 |
| Icaraí de Minas | MG | 0 | 11.990 | 0 | 0,1 |
| Igaratinga | MG | 0 | 10.860 | 0 | 0,0 |
| Ijaci | MG | 0 | 6.550 | 0 | 0,0 |
| Inconfidentes | MG | 0 | 7.328 | 0 | 1,2 |
| Inimutaba | MG | 0 | 7.515 | 0 | 0,7 |
| Ipaba | MG | 0 | 18.607 | 0 | 0,0 |
| Itacambira | MG | 0 | 5.385 | 0 | 68,1 |
| Itamonte | MG | 0 | 15.579 | 0 | 0,0 |
| Itanhandu | MG | 0 | 15.331 | 0 | 0,0 |
| Itapeva | MG | 0 | 9.783 | 0 | 0,0 |
| Itumirim | MG | 0 | 6.023 | 0 | 0,0 |
| Japaraíba | MG | 0 | 4.350 | 0 | 0,0 |
| Jesuânia | MG | 0 | 4.787 | 0 | 0,4 |
| Juramento | MG | 0 | 4.331 | 0 | 4,5 |
| Leandro Ferreira | MG | 0 | 3.229 | 0 | 0,0 |
| Liberdade | MG | 0 | 5.069 | 0 | 0,0 |
| Luminárias | MG | 0 | 5.446 | 0 | 0,0 |
| Mamonas | MG | 0 | 6.543 | 0 | 0,2 |
| Marilac | MG | 0 | 4.115 | 0 | 0,0 |
| Mendes Pimentel | MG | 0 | 6.446 | 0 | 0,0 |
| Moeda | MG | 0 | 4.919 | 0 | 0,0 |
| Morada Nova de Minas | MG | 0 | 8.863 | 0 | 15,5 |
| Munhoz | MG | 0 | 6.029 | 0 | 0,0 |
| Natalândia | MG | 0 | 3.311 | 0 | 9,3 |
| Olhos-d'Água | MG | 0 | 6.096 | 0 | 28,1 |
| Olímpio Noronha | MG | 0 | 2.787 | 0 | 0,0 |
| Oliveira Fortes | MG | 0 | 2.133 | 0 | 0,0 |
| Onça de Pitangui | MG | 0 | 3.148 | 0 | 0,0 |
| Passabém | MG | 0 | 1.649 | 0 | 0,1 |
| Passa Quatro | MG | 0 | 16.344 | 0 | 0,0 |
| Patrocínio do Muriaé | MG | 0 | 5.684 | 0 | 0,0 |
| Pedra do Anta | MG | 0 | 3.052 | 0 | 0,0 |
| Pequeri | MG | 0 | 3.320 | 0 | 0,0 |
| Perdões | MG | 0 | 21.390 | 0 | 0,0 |
| Pescador | MG | 0 | 4.252 | 0 | 11,5 |
| Piedade de Ponte Nova | MG | 0 | 4.140 | 0 | 0,0 |
| Piedade do Rio Grande | MG | 0 | 4.497 | 0 | 0,0 |
| Pingo-d'Água | MG | 0 | 4.941 | 0 | 0,0 |
| Pirajuba | MG | 0 | 6.199 | 0 | 0,0 |
| Piranguinho | MG | 0 | 8.596 | 0 | 0,0 |
| Presidente Juscelino | MG | 0 | 3.641 | 0 | 11,6 |
| Presidente Kubitschek | MG | 0 | 3.002 | 0 | 0,8 |
| Prudente de Morais | MG | 0 | 10.733 | 0 | 0,0 |
| Quartel Geral | MG | 0 | 3.563 | 0 | 1,1 |
| Queluzito | MG | 0 | 1.939 | 0 | 0,0 |
| Raposos | MG | 0 | 16.354 | 0 | 0,0 |
| Recreio | MG | 0 | 10.517 | 0 | 0,0 |
| Ribeirão Vermelho | MG | 0 | 4.033 | 0 | 0,0 |
| Rio Acima | MG | 0 | 10.312 | 0 | 0,0 |
| Rio Doce | MG | 0 | 2.610 | 0 | 0,0 |
| Rio Manso | MG | 0 | 5.832 | 0 | 0,0 |
| Rio Piracicaba | MG | 0 | 14.339 | 0 | 0,0 |
| Rio Preto | MG | 0 | 5.476 | 0 | 0,0 |
| Ritápolis | MG | 0 | 4.604 | 0 | 0,6 |
| Rochedo de Minas | MG | 0 | 2.305 | 0 | 0,0 |
| Rodeiro | MG | 0 | 8.109 | 0 | 0,0 |
| Santa Bárbara | MG | 0 | 31.324 | 0 | 0,0 |
| Santa Bárbara do Monte Verde | MG | 0 | 3.150 | 0 | 0,7 |
| Santa Cruz de Minas | MG | 0 | 8.604 | 0 | 0,0 |
| Santa Cruz de Salinas | MG | 0 | 4.142 | 0 | 9,8 |
| Santa Fé de Minas | MG | 0 | 3.846 | 0 | 26,3 |
| Santana do Deserto | MG | 0 | 3.976 | 0 | 0,0 |
| Santana do Garambéu | MG | 0 | 2.458 | 0 | 0,3 |
| Santa Rita de Caldas | MG | 0 | 8.949 | 0 | 8,0 |
| Santa Rita de Minas | MG | 0 | 7.212 | 0 | 0,0 |
| São Brás do Suaçuí | MG | 0 | 3.738 | 0 | 0,0 |
| São Geraldo do Baixio | MG | 0 | 4.012 | 0 | 0,4 |
| São Gonçalo do Abaeté | MG | 0 | 8.389 | 0 | 10,6 |
| São João da Mata | MG | 0 | 2.749 | 0 | 0,0 |
| São João del Rei | MG | 0 | 90.082 | 0 | 0,0 |
| São José da Barra | MG | 0 | 7.426 | 0 | 0,0 |
| São José da Lapa | MG | 0 | 23.766 | 0 | 0,0 |
| São José da Varginha | MG | 0 | 5.004 | 0 | 0,0 |
| São José do Alegre | MG | 0 | 4.196 | 0 | 0,0 |
| São José do Divino | MG | 0 | 3.860 | 0 | 5,8 |
| São Sebastião da Bela Vista | MG | 0 | 5.504 | 0 | 0,0 |
| São Sebastião do Rio Preto | MG | 0 | 1.506 | 0 | 0,3 |
| Sapucaí-Mirim | MG | 0 | 6.930 | 0 | 0,0 |
| Sarzedo | MG | 0 | 32.752 | 0 | 0,0 |
| Sem-Peixe | MG | 0 | 2.633 | 0 | 0,0 |
| Senador Amaral | MG | 0 | 5.356 | 0 | 0,0 |
| Senador Cortes | MG | 0 | 2.005 | 0 | 0,0 |
| Senador José Bento | MG | 0 | 1.502 | 0 | 0,0 |
| Serra da Saudade | MG | 0 | 781 | 0 | 1,3 |
| Serra do Salitre | MG | 0 | 11.582 | 0 | 6,8 |
| Serranos | MG | 0 | 1.963 | 0 | 0,0 |
| Silveirânia | MG | 0 | 2.261 | 0 | 0,4 |
| Tiradentes | MG | 0 | 7.981 | 0 | 0,0 |
| Toledo | MG | 0 | 6.258 | 0 | 0,0 |
| Vargem Bonita | MG | 0 | 2.153 | 0 | 0,1 |
| Vargem Grande do Rio Pardo | MG | 0 | 5.007 | 0 | 17,5 |
| Mathias Lobato | MG | 0 | 3.203 | 0 | 0,3 |
| Wenceslau Braz | MG | 0 | 2.552 | 0 | 0,0 |
| Bom Jesus do Norte | ES | 0 | 9.936 | 0 | 0,0 |
| Ibiraçu | ES | 0 | 12.479 | 0 | 0,0 |
| Iconha | ES | 0 | 13.860 | 0 | 0,0 |
| Marataízes | ES | 0 | 38.499 | 0 | 0,0 |
| Viana | ES | 0 | 78.239 | 0 | 0,0 |
| Vila Velha | ES | 2 | 493.838 | 0 | 0,0 |
| Vitória | ES | 0 | 362.097 | 0 | 0,0 |
| Belford Roxo | RJ | 1 | 510.906 | 0 | 0,0 |
| Carapebus | RJ | 0 | 16.301 | 0 | 0,0 |
| Italva | RJ | 0 | 15.207 | 0 | 0,3 |
| Mesquita | RJ | 0 | 176.103 | 0 | 0,0 |
| Queimados | RJ | 0 | 150.319 | 0 | 0,0 |
| São João de Meriti | RJ | 2 | 472.406 | 0 | 0,0 |
| São Sebastião do Alto | RJ | 0 | 9.357 | 0 | 6,5 |
| Silva Jardim | RJ | 0 | 21.774 | 0 | 0,0 |
| Trajano de Moraes | RJ | 0 | 10.626 | 0 | 0,0 |
| Águas de Santa Bárbara | SP | 0 | 6.075 | 0 | 0,1 |
| Alfredo Marcondes | SP | 0 | 4.166 | 0 | 0,0 |
| Alto Alegre | SP | 0 | 4.099 | 0 | 0,0 |
| Álvares Machado | SP | 0 | 24.915 | 0 | 0,0 |
| Alvinlândia | SP | 0 | 3.222 | 0 | 0,0 |
| Américo Brasiliense | SP | 0 | 40.504 | 0 | 0,0 |
| Analândia | SP | 0 | 4.995 | 0 | 0,0 |
| Aramina | SP | 0 | 5.620 | 0 | 0,0 |
| Arapeí | SP | 0 | 2.469 | 0 | 0,0 |
| Arco-Íris | SP | 0 | 1.791 | 0 | 0,0 |
| Artur Nogueira | SP | 0 | 54.408 | 0 | 0,0 |
| Auriflama | SP | 0 | 15.189 | 0 | 0,0 |
| Balbinos | SP | 0 | 5.735 | 0 | 0,0 |
| Barão de Antonina | SP | 0 | 3.469 | 0 | 0,0 |
| Bento de Abreu | SP | 0 | 2.980 | 0 | 0,0 |
| Bilac | SP | 0 | 8.034 | 0 | 0,0 |
| Bom Jesus dos Perdões | SP | 0 | 25.448 | 0 | 0,0 |
| Bom Sucesso de Itararé | SP | 0 | 3.954 | 0 | 0,0 |
| Borá | SP | 0 | 837 | 0 | 0,0 |
| Boracéia | SP | 0 | 4.823 | 0 | 0,0 |
| Brejo Alegre | SP | 0 | 2.865 | 0 | 0,0 |
| Cabrália Paulista | SP | 0 | 4.264 | 0 | 0,3 |
| Cafelândia | SP | 0 | 17.767 | 0 | 0,1 |
| Caiabu | SP | 0 | 4.191 | 0 | 0,0 |
| Caiuá | SP | 0 | 5.874 | 0 | 0,0 |
| Cândido Rodrigues | SP | 0 | 2.793 | 0 | 0,0 |
| Canitar | SP | 0 | 5.216 | 0 | 0,0 |
| Catiguá | SP | 0 | 7.804 | 0 | 0,0 |
| Cerquilho | SP | 0 | 48.949 | 0 | 0,0 |
| Charqueada | SP | 0 | 17.190 | 0 | 0,0 |
| Colina | SP | 0 | 18.468 | 0 | 0,0 |
| Coroados | SP | 0 | 6.058 | 0 | 0,0 |
| Corumbataí | SP | 0 | 4.055 | 0 | 0,0 |
| Cosmópolis | SP | 0 | 72.252 | 0 | 0,0 |
| Cravinhos | SP | 0 | 35.292 | 0 | 0,0 |
| Cruzália | SP | 0 | 2.073 | 0 | 0,0 |
| Dirce Reis | SP | 0 | 1.793 | 0 | 0,0 |
| Dolcinópolis | SP | 0 | 2.115 | 0 | 0,0 |
| Dourado | SP | 0 | 8.873 | 0 | 0,0 |
| Elias Fausto | SP | 0 | 17.772 | 0 | 0,0 |
| Elisiário | SP | 0 | 3.651 | 0 | 0,0 |
| Emilianópolis | SP | 0 | 3.214 | 0 | 0,0 |
| Engenheiro Coelho | SP | 0 | 20.773 | 0 | 0,0 |
| ES do Pinhal | SP | 0 | 44.330 | 0 | 0,0 |
| Fernando Prestes | SP | 0 | 5.783 | 0 | 0,0 |
| Fernão | SP | 0 | 1.716 | 0 | 0,0 |
| Flora Rica | SP | 0 | 1.464 | 0 | 0,0 |
| Florínia | SP | 0 | 2.676 | 0 | 0,0 |
| Gabriel Monteiro | SP | 0 | 2.776 | 0 | 0,0 |
| Garça | SP | 0 | 44.390 | 0 | 0,0 |
| Gavião Peixoto | SP | 0 | 4.789 | 0 | 0,0 |
| Guará | SP | 0 | 21.220 | 0 | 0,0 |
| Guaraci | SP | 0 | 11.188 | 0 | 2,9 |
| Guarani d'Oeste | SP | 0 | 2.000 | 0 | 0,0 |
| Guatapará | SP | 0 | 7.656 | 0 | 0,0 |
| Guzolândia | SP | 0 | 5.267 | 0 | 0,0 |
| Herculândia | SP | 0 | 9.526 | 0 | 0,0 |
| Holambra | SP | 0 | 14.930 | 0 | 0,0 |
| Hortolândia | SP | 1 | 230.851 | 0 | 0,0 |
| Iacri | SP | 0 | 6.321 | 0 | 0,0 |
| Iaras | SP | 0 | 9.240 | 0 | 0,0 |
| Icém | SP | 0 | 8.243 | 0 | 0,5 |
| Iepê | SP | 0 | 8.159 | 0 | 0,1 |
| Indaiatuba | SP | 1 | 251.627 | 0 | 0,0 |
| Indiana | SP | 0 | 4.885 | 0 | 0,0 |
| Indiaporã | SP | 0 | 3.897 | 0 | 0,0 |
| Inúbia Paulista | SP | 0 | 3.991 | 0 | 0,0 |
| Ipaussu | SP | 0 | 14.971 | 0 | 0,0 |
| Ipeúna | SP | 0 | 7.546 | 0 | 0,0 |
| Irapuã | SP | 0 | 7.993 | 0 | 0,0 |
| Irapuru | SP | 0 | 8.294 | 0 | 0,0 |
| Itaí | SP | 0 | 27.125 | 0 | 0,0 |
| Itajobi | SP | 0 | 15.262 | 0 | 0,0 |
| Itaju | SP | 0 | 3.835 | 0 | 0,0 |
| Itapira | SP | 0 | 74.773 | 0 | 0,0 |
| Itapirapuã Paulista | SP | 0 | 4.241 | 0 | 3,5 |
| Itapuí | SP | 0 | 13.992 | 0 | 0,0 |
| Itapura | SP | 0 | 4.906 | 0 | 0,0 |
| Jaborandi | SP | 0 | 6.929 | 0 | 0,0 |
| Jaci | SP | 0 | 7.067 | 0 | 0,0 |
| Jambeiro | SP | 0 | 6.602 | 0 | 0,0 |
| João Ramalho | SP | 0 | 4.523 | 0 | 1,0 |
| Jumirim | SP | 0 | 3.367 | 0 | 0,0 |
| Lagoinha | SP | 0 | 4.896 | 0 | 0,0 |
| Laranjal Paulista | SP | 0 | 28.516 | 0 | 0,0 |
| Leme | SP | 0 | 103.391 | 0 | 0,0 |
| Lindóia | SP | 0 | 7.980 | 0 | 0,0 |
| Lucianópolis | SP | 0 | 2.394 | 0 | 0,0 |
| Lupércio | SP | 0 | 4.584 | 0 | 0,0 |
| Macedônia | SP | 0 | 3.698 | 0 | 0,0 |
| Maracaí | SP | 0 | 14.002 | 0 | 0,5 |
| Marapoama | SP | 0 | 3.031 | 0 | 0,0 |
| Mariápolis | SP | 0 | 4.084 | 0 | 0,0 |
| Martinópolis | SP | 0 | 26.461 | 0 | 0,1 |
| Mauá | SP | 1 | 472.912 | 0 | 0,0 |
| Mendonça | SP | 0 | 5.490 | 0 | 0,0 |
| Meridiano | SP | 0 | 3.836 | 0 | 0,0 |
| Monções | SP | 0 | 2.259 | 0 | 0,0 |
| Monte Azul Paulista | SP | 0 | 19.008 | 0 | 0,0 |
| Morro Agudo | SP | 0 | 32.968 | 0 | 0,1 |
| Motuca | SP | 0 | 4.758 | 0 | 0,0 |
| Murutinga do Sul | SP | 0 | 4.486 | 0 | 0,0 |
| Nantes | SP | 0 | 3.141 | 0 | 0,1 |
| Narandiba | SP | 0 | 4.857 | 0 | 3,2 |
| Nova Aliança | SP | 0 | 6.973 | 0 | 0,0 |
| Nova Canaã Paulista | SP | 0 | 1.881 | 0 | 0,0 |
| Nova Castilho | SP | 0 | 1.267 | 0 | 0,0 |
| Nova Europa | SP | 0 | 11.186 | 0 | 0,0 |
| Nova Independência | SP | 0 | 3.969 | 0 | 0,0 |
| Novais | SP | 0 | 5.830 | 0 | 0,0 |
| Nova Odessa | SP | 0 | 60.174 | 0 | 0,0 |
| Ocauçu | SP | 0 | 4.289 | 0 | 0,0 |
| Orindiúva | SP | 0 | 7.066 | 0 | 0,0 |
| Orlândia | SP | 0 | 44.028 | 0 | 0,0 |
| Oscar Bressane | SP | 0 | 2.603 | 0 | 0,3 |
| Ouroeste | SP | 0 | 10.361 | 0 | 0,0 |
| Ouro Verde | SP | 0 | 8.562 | 0 | 0,1 |
| Palmital | SP | 0 | 22.221 | 0 | 0,0 |
| Panorama | SP | 0 | 15.777 | 0 | 0,1 |
| Paraguaçu Paulista | SP | 0 | 45.703 | 0 | 0,0 |
| Paraíso | SP | 0 | 6.454 | 0 | 0,0 |
| Parapuã | SP | 0 | 10.964 | 0 | 0,0 |
| Paulistânia | SP | 0 | 1.833 | 0 | 2,4 |
| Pedrinhas Paulista | SP | 0 | 3.093 | 0 | 0,0 |
| Pereiras | SP | 0 | 8.668 | 0 | 0,0 |
| Piacatu | SP | 0 | 5.980 | 0 | 0,0 |
| Piquerobi | SP | 0 | 3.692 | 0 | 0,0 |
| Pirangi | SP | 0 | 11.417 | 0 | 0,0 |
| Piratininga | SP | 0 | 13.636 | 0 | 0,0 |
| Pitangueiras | SP | 0 | 39.719 | 0 | 0,0 |
| Planalto | SP | 0 | 5.237 | 0 | 0,0 |
| Platina | SP | 0 | 3.550 | 0 | 0,0 |
| Poá | SP | 0 | 117.452 | 0 | 0,0 |
| Poloni | SP | 0 | 6.059 | 0 | 0,0 |
| Pompéia | SP | 0 | 22.014 | 0 | 0,0 |
| Pongaí | SP | 0 | 3.416 | 0 | 0,0 |
| Pontal | SP | 0 | 49.961 | 0 | 0,0 |
| Pontes Gestal | SP | 0 | 2.577 | 0 | 0,0 |
| Populina | SP | 0 | 4.169 | 0 | 0,1 |
| Pracinha | SP | 0 | 4.093 | 0 | 0,0 |
| Presidente Alves | SP | 0 | 4.094 | 0 | 0,0 |
| Quadra | SP | 0 | 3.804 | 0 | 0,0 |
| Quatá | SP | 0 | 14.109 | 0 | 0,1 |
| Queiroz | SP | 0 | 3.406 | 0 | 0,0 |
| Quintana | SP | 0 | 6.638 | 0 | 0,0 |
| Rafard | SP | 0 | 9.076 | 0 | 0,0 |
| Regente Feijó | SP | 0 | 20.261 | 0 | 0,0 |
| Restinga | SP | 0 | 7.593 | 0 | 0,0 |
| Ribeirão Bonito | SP | 0 | 13.219 | 0 | 0,0 |
| Ribeirão Corrente | SP | 0 | 4.718 | 0 | 0,0 |
| Ribeirão dos Índios | SP | 0 | 2.225 | 0 | 0,3 |
| Rifaina | SP | 0 | 3.629 | 0 | 0,0 |
| Rio das Pedras | SP | 0 | 35.228 | 0 | 0,0 |
| Sagres | SP | 0 | 2.432 | 0 | 0,0 |
| Sales | SP | 0 | 6.331 | 0 | 0,0 |
| Sales Oliveira | SP | 0 | 11.890 | 0 | 0,0 |
| Salto Grande | SP | 0 | 9.331 | 0 | 0,0 |
| Sandovalina | SP | 0 | 4.302 | 0 | 0,3 |
| Santa Adélia | SP | 0 | 15.480 | 0 | 0,0 |
| Santa Albertina | SP | 0 | 6.008 | 0 | 0,0 |
| Santa Cruz da Conceição | SP | 0 | 4.503 | 0 | 0,0 |
| Santa Cruz da Esperança | SP | 0 | 2.139 | 0 | 0,0 |
| Santa Gertrudes | SP | 0 | 26.898 | 0 | 0,0 |
| Santa Mercedes | SP | 0 | 2.939 | 0 | 0,0 |
| Santana da Ponte Pensa | SP | 0 | 1.487 | 0 | 0,0 |
| Santa Rita d'Oeste | SP | 0 | 2.498 | 0 | 0,0 |
| Santo Expedito | SP | 0 | 3.111 | 0 | 0,0 |
| Santópolis do Aguapeí | SP | 0 | 4.777 | 0 | 0,0 |
| São João de Iracema | SP | 0 | 1.922 | 0 | 0,0 |
| São João do Pau d'Alho | SP | 0 | 2.105 | 0 | 0,0 |
| São José da Bela Vista | SP | 0 | 8.928 | 0 | 0,0 |
| São José do Barreiro | SP | 0 | 4.147 | 0 | 0,6 |
| São Simão | SP | 0 | 15.322 | 0 | 0,2 |
| Serra Azul | SP | 0 | 14.662 | 0 | 0,0 |
| Serrana | SP | 0 | 45.107 | 0 | 0,0 |
| Tabapuã | SP | 0 | 12.407 | 0 | 0,0 |
| Taciba | SP | 0 | 6.285 | 0 | 0,0 |
| Taguaí | SP | 0 | 13.859 | 0 | 0,0 |
| Taiaçu | SP | 0 | 6.295 | 0 | 0,0 |
| Taiúva | SP | 0 | 5.566 | 0 | 0,0 |
| Taquaral | SP | 0 | 2.811 | 0 | 0,0 |
| Taquarituba | SP | 0 | 23.218 | 0 | 0,0 |
| Taquarivaí | SP | 0 | 5.852 | 0 | 0,0 |
| Tarabai | SP | 0 | 7.468 | 0 | 0,0 |
| Tarumã | SP | 0 | 15.000 | 0 | 0,0 |
| Teodoro Sampaio | SP | 0 | 23.148 | 0 | 3,8 |
| Terra Roxa | SP | 0 | 9.370 | 0 | 0,0 |
| Timburi | SP | 0 | 2.658 | 0 | 0,0 |
| Torre de Pedra | SP | 0 | 2.412 | 0 | 0,0 |
| Tupi Paulista | SP | 0 | 15.495 | 0 | 0,0 |
| Turiúba | SP | 0 | 2.016 | 0 | 0,0 |
| Turmalina | SP | 0 | 1.727 | 0 | 0,0 |
| Ubarana | SP | 0 | 6.309 | 0 | 0,0 |
| Uchoa | SP | 0 | 10.110 | 0 | 0,0 |
| União Paulista | SP | 0 | 1.844 | 0 | 0,0 |
| Uru | SP | 0 | 1.165 | 0 | 0,0 |
| Urupês | SP | 0 | 13.809 | 0 | 0,0 |
| Vera Cruz | SP | 0 | 10.843 | 0 | 0,0 |
| Vinhedo | SP | 0 | 78.728 | 0 | 0,0 |
| Viradouro | SP | 0 | 18.898 | 0 | 0,0 |
| Chavantes | SP | 0 | 12.418 | 0 | 0,0 |
| Abatiá | PR | 0 | 7.457 | 0 | 1,0 |
| Alto Paraná | PR | 0 | 14.770 | 0 | 0,0 |
| Alto Piquiri | PR | 0 | 9.836 | 0 | 0,0 |
| Amaporã | PR | 0 | 6.257 | 0 | 0,0 |
| Andirá | PR | 0 | 20.031 | 0 | 0,0 |
| Ângulo | PR | 0 | 2.928 | 0 | 0,0 |
| Arapuã | PR | 0 | 3.068 | 0 | 0,1 |
| Ariranha do Ivaí | PR | 0 | 2.108 | 0 | 0,1 |
| Astorga | PR | 0 | 26.111 | 0 | 0,0 |
| Atalaia | PR | 0 | 3.892 | 0 | 0,0 |
| Bandeirantes | PR | 0 | 31.367 | 0 | 0,0 |
| Barracão | PR | 0 | 10.275 | 0 | 0,0 |
| Barra do Jacaré | PR | 0 | 2.781 | 0 | 0,0 |
| Bela Vista da Caroba | PR | 0 | 3.511 | 0 | 0,0 |
| Bela Vista do Paraíso | PR | 0 | 15.397 | 0 | 0,1 |
| Boa Esperança | PR | 0 | 4.105 | 0 | 0,0 |
| Boa Esperança do Iguaçu | PR | 0 | 2.503 | 0 | 0,0 |
| Bom Jesus do Sul | PR | 0 | 3.541 | 0 | 0,0 |
| Brasilândia do Sul | PR | 0 | 2.651 | 0 | 0,0 |
| Cafeara | PR | 0 | 2.934 | 0 | 0,0 |
| Cafezal do Sul | PR | 0 | 4.044 | 0 | 0,0 |
| Cambará | PR | 0 | 25.360 | 0 | 0,0 |
| Cambira | PR | 0 | 7.865 | 0 | 0,0 |
| Campo Bonito | PR | 0 | 3.833 | 0 | 2,2 |
| Campo do Tenente | PR | 0 | 7.971 | 0 | 0,0 |
| Campo Magro | PR | 0 | 29.318 | 0 | 0,0 |
| Carambeí | PR | 0 | 23.415 | 0 | 0,0 |
| Céu Azul | PR | 0 | 11.765 | 0 | 0,0 |
| Conselheiro Mairinck | PR | 0 | 3.860 | 0 | 0,0 |
| Corumbataí do Sul | PR | 0 | 3.219 | 0 | 0,0 |
| Cruzeiro do Iguaçu | PR | 0 | 4.252 | 0 | 0,0 |
| Cruzmaltina | PR | 0 | 2.950 | 0 | 0,0 |
| Diamante D'Oeste | PR | 0 | 5.253 | 0 | 1,8 |
| Douradina | PR | 0 | 8.747 | 0 | 0,0 |
| Doutor Camargo | PR | 0 | 5.979 | 0 | 0,0 |
| Enéas Marques | PR | 0 | 5.961 | 0 | 0,0 |
| Engenheiro Beltrão | PR | 0 | 14.000 | 0 | 0,0 |
| Esperança Nova | PR | 0 | 1.698 | 0 | 0,0 |
| Fênix | PR | 0 | 4.762 | 0 | 0,0 |
| Floraí | PR | 0 | 4.929 | 0 | 0,0 |
| Flor da Serra do Sul | PR | 0 | 4.624 | 0 | 0,0 |
| Florestópolis | PR | 0 | 10.548 | 0 | 0,6 |
| Flórida | PR | 0 | 2.689 | 0 | 0,0 |
| Francisco Alves | PR | 0 | 6.046 | 0 | 0,0 |
| Foz do Jordão | PR | 0 | 4.650 | 0 | 0,0 |
| Godoy Moreira | PR | 0 | 2.946 | 0 | 0,0 |
| Goioxim | PR | 0 | 7.111 | 0 | 46,3 |
| Guairaçá | PR | 0 | 6.581 | 0 | 0,0 |
| Guaporema | PR | 0 | 2.243 | 0 | 0,0 |
| Guaraci | PR | 0 | 5.502 | 0 | 0,2 |
| Ibiporã | PR | 0 | 54.558 | 0 | 0,0 |
| Iguaraçu | PR | 0 | 4.404 | 0 | 0,0 |
| Inajá | PR | 0 | 3.109 | 0 | 0,0 |
| Indianópolis | PR | 0 | 4.457 | 0 | 0,0 |
| Itaguajé | PR | 0 | 4.466 | 0 | 0,0 |
| Itaipulândia | PR | 0 | 11.176 | 0 | 0,0 |
| Itambaracá | PR | 0 | 6.582 | 0 | 0,0 |
| Itambé | PR | 0 | 6.108 | 0 | 0,0 |
| Ivaiporã | PR | 0 | 31.984 | 0 | 0,0 |
| Ivaté | PR | 0 | 8.185 | 0 | 0,0 |
| Ivatuba | PR | 0 | 3.259 | 0 | 0,0 |
| Jacarezinho | PR | 0 | 39.378 | 0 | 0,0 |
| Jandaia do Sul | PR | 0 | 21.176 | 0 | 0,0 |
| Japira | PR | 0 | 4.994 | 0 | 0,0 |
| Japurá | PR | 0 | 9.425 | 0 | 0,0 |
| Jardim Alegre | PR | 0 | 11.328 | 0 | 0,0 |
| Jardim Olinda | PR | 0 | 1.331 | 0 | 0,0 |
| Joaquim Távora | PR | 0 | 11.908 | 0 | 0,0 |
| Jundiaí do Sul | PR | 0 | 3.292 | 0 | 0,0 |
| Jussara | PR | 0 | 7.013 | 0 | 0,0 |
| Lindoeste | PR | 0 | 4.676 | 0 | 0,4 |
| Lunardelli | PR | 0 | 4.794 | 0 | 0,0 |
| Maria Helena | PR | 0 | 5.677 | 0 | 0,0 |
| Marilena | PR | 0 | 7.076 | 0 | 0,0 |
| Mariópolis | PR | 0 | 6.610 | 0 | 0,0 |
| Medianeira | PR | 0 | 46.198 | 0 | 0,0 |
| Mirador | PR | 0 | 2.213 | 0 | 0,0 |
| Miraselva | PR | 0 | 1.806 | 0 | 0,0 |
| Munhoz de Melo | PR | 0 | 3.984 | 0 | 0,0 |
| Nossa Senhora das Graças | PR | 0 | 4.008 | 0 | 0,0 |
| Nova Aliança do Ivaí | PR | 0 | 1.543 | 0 | 0,0 |
| Nova América da Colina | PR | 0 | 3.445 | 0 | 0,0 |
| Nova Londrina | PR | 0 | 13.213 | 0 | 0,0 |
| Nova Olímpia | PR | 0 | 5.806 | 0 | 0,0 |
| Ourizona | PR | 0 | 3.428 | 0 | 0,0 |
| Paiçandu | PR | 0 | 41.281 | 0 | 0,0 |
| Paranacity | PR | 0 | 11.472 | 0 | 0,0 |
| Paranapoema | PR | 0 | 3.203 | 0 | 0,0 |
| Peabiru | PR | 0 | 13.996 | 0 | 0,0 |
| Perobal | PR | 0 | 6.127 | 0 | 0,0 |
| Pérola | PR | 0 | 11.234 | 0 | 0,0 |
| Pérola d'Oeste | PR | 0 | 6.347 | 0 | 0,0 |
| Pinhalão | PR | 0 | 6.326 | 0 | 0,0 |
| Porecatu | PR | 0 | 12.914 | 0 | 0,0 |
| Porto Rico | PR | 0 | 2.559 | 0 | 0,0 |
| Prado Ferreira | PR | 0 | 3.753 | 0 | 0,0 |
| Presidente Castelo Branco | PR | 0 | 5.306 | 0 | 0,0 |
| Primeiro de Maio | PR | 0 | 11.123 | 0 | 0,5 |
| Quarto Centenário | PR | 0 | 4.512 | 0 | 0,0 |
| Quatiguá | PR | 0 | 7.449 | 0 | 0,0 |
| Quatro Pontes | PR | 0 | 4.015 | 0 | 0,0 |
| Quinta do Sol | PR | 0 | 4.573 | 0 | 0,0 |
| Renascença | PR | 0 | 6.802 | 0 | 0,0 |
| Reserva do Iguaçu | PR | 0 | 8.010 | 0 | 0,6 |
| Rolândia | PR | 0 | 66.580 | 0 | 0,0 |
| Salto do Itararé | PR | 0 | 4.935 | 0 | 0,0 |
| Santa Amélia | PR | 0 | 3.324 | 0 | 0,0 |
| Santa Cecília do Pavão | PR | 0 | 3.334 | 0 | 0,0 |
| Santa Cruz de Monte Castelo | PR | 0 | 7.800 | 0 | 0,0 |
| Santa Fé | PR | 0 | 12.037 | 0 | 0,0 |
| Santa Inês | PR | 0 | 1.596 | 0 | 0,0 |
| Santa Lúcia | PR | 0 | 3.813 | 0 | 0,0 |
| Santa Mônica | PR | 0 | 3.981 | 0 | 0,0 |
| Santana do Itararé | PR | 0 | 4.992 | 0 | 0,0 |
| Santa Tereza do Oeste | PR | 0 | 10.139 | 0 | 0,0 |
| Santo Antônio do Caiuá | PR | 0 | 2.641 | 0 | 0,0 |
| Santo Inácio | PR | 0 | 5.438 | 0 | 0,0 |
| São Carlos do Ivaí | PR | 0 | 6.878 | 0 | 0,0 |
| São João do Caiuá | PR | 0 | 5.855 | 0 | 0,0 |
| São João do Ivaí | PR | 0 | 10.219 | 0 | 0,0 |
| São Jorge do Ivaí | PR | 0 | 5.551 | 0 | 0,0 |
| São Jorge do Patrocínio | PR | 0 | 5.641 | 0 | 0,0 |
| São José da Boa Vista | PR | 0 | 6.206 | 0 | 0,0 |
| São Manoel do Paraná | PR | 0 | 2.160 | 0 | 0,0 |
| São Pedro do Iguaçu | PR | 0 | 5.897 | 0 | 0,0 |
| São Pedro do Ivaí | PR | 0 | 10.981 | 0 | 0,0 |
| São Pedro do Paraná | PR | 0 | 2.313 | 0 | 0,0 |
| São Tomé | PR | 0 | 5.722 | 0 | 0,0 |
| Serranópolis do Iguaçu | PR | 0 | 4.495 | 0 | 0,0 |
| Sertaneja | PR | 0 | 5.284 | 0 | 0,2 |
| Tamboara | PR | 0 | 5.120 | 0 | 0,0 |
| Tapira | PR | 0 | 5.539 | 0 | 0,0 |
| Terra Rica | PR | 0 | 16.789 | 0 | 0,0 |
| Tunas do Paraná | PR | 0 | 8.769 | 0 | 0,0 |
| Tuneiras do Oeste | PR | 0 | 8.566 | 0 | 0,0 |
| Tupãssi | PR | 0 | 8.124 | 0 | 0,0 |
| União da Vitória | PR | 0 | 57.517 | 0 | 0,3 |
| Uniflor | PR | 0 | 2.605 | 0 | 0,0 |
| Ventania | PR | 0 | 11.892 | 0 | 0,0 |
| Verê | PR | 0 | 7.257 | 0 | 0,0 |
| Alto Paraíso | PR | 0 | 2.741 | 0 | 0,0 |
| Doutor Ulysses | PR | 0 | 5.580 | 0 | 1,5 |
| Abdon Batista | SC | 0 | 2.563 | 0 | 0,0 |
| Águas Frias | SC | 0 | 2.366 | 0 | 0,0 |
| Alfredo Wagner | SC | 0 | 10.036 | 0 | 0,0 |
| Alto Bela Vista | SC | 0 | 1.937 | 0 | 0,0 |
| Anitápolis | SC | 0 | 3.232 | 0 | 0,0 |
| Arabutã | SC | 0 | 4.267 | 0 | 0,0 |
| Armazém | SC | 0 | 8.674 | 0 | 0,0 |
| Ascurra | SC | 0 | 7.934 | 0 | 0,0 |
| Atalanta | SC | 0 | 3.210 | 0 | 0,0 |
| Bela Vista do Toldo | SC | 0 | 6.337 | 0 | 0,0 |
| Belmonte | SC | 0 | 2.706 | 0 | 0,0 |
| Bom Jardim da Serra | SC | 0 | 4.743 | 0 | 0,0 |
| Bom Retiro | SC | 0 | 9.966 | 0 | 0,1 |
| Braço do Trombudo | SC | 0 | 3.743 | 0 | 0,0 |
| Calmon | SC | 0 | 3.346 | 0 | 9,5 |
| Capão Alto | SC | 0 | 2.525 | 0 | 1,9 |
| Capivari de Baixo | SC | 0 | 24.871 | 0 | 0,0 |
| Celso Ramos | SC | 0 | 2.728 | 0 | 0,0 |
| Cerro Negro | SC | 0 | 3.124 | 0 | 7,0 |
| Cocal do Sul | SC | 0 | 16.684 | 0 | 0,0 |
| Coronel Martins | SC | 0 | 2.549 | 0 | 0,5 |
| Cunhataí | SC | 0 | 1.962 | 0 | 0,0 |
| Dona Emma | SC | 0 | 4.146 | 0 | 2,0 |
| Doutor Pedrinho | SC | 0 | 4.064 | 0 | 0,0 |
| Ermo | SC | 0 | 2.063 | 0 | 0,0 |
| Erval Velho | SC | 0 | 4.412 | 0 | 0,0 |
| Formosa do Sul | SC | 0 | 2.510 | 0 | 0,0 |
| Forquilhinha | SC | 0 | 26.793 | 0 | 0,0 |
| Frei Rogério | SC | 0 | 2.023 | 0 | 0,0 |
| Galvão | SC | 0 | 2.873 | 0 | 0,0 |
| Governador Celso Ramos | SC | 0 | 14.471 | 0 | 0,0 |
| Grão Pará | SC | 0 | 6.569 | 0 | 0,0 |
| Gravatal | SC | 0 | 11.501 | 0 | 0,0 |
| Ibiam | SC | 0 | 1.957 | 0 | 0,0 |
| Ibicaré | SC | 0 | 3.202 | 0 | 0,0 |
| Imaruí | SC | 0 | 10.135 | 0 | 0,0 |
| Imbuia | SC | 0 | 6.197 | 0 | 0,0 |
| Iomerê | SC | 0 | 2.945 | 0 | 0,0 |
| Ipira | SC | 0 | 4.446 | 0 | 0,0 |
| Irani | SC | 0 | 10.419 | 0 | 0,3 |
| Itá | SC | 0 | 6.169 | 0 | 0,0 |
| Joaçaba | SC | 0 | 30.118 | 0 | 0,0 |
| Jupiá | SC | 0 | 2.101 | 0 | 0,0 |
| Lajeado Grande | SC | 0 | 1.427 | 0 | 0,0 |
| Laurentino | SC | 0 | 6.970 | 0 | 0,0 |
| Leoberto Leal | SC | 0 | 3.041 | 0 | 2,1 |
| Lindóia do Sul | SC | 0 | 4.563 | 0 | 2,5 |
| Lontras | SC | 0 | 12.130 | 0 | 0,0 |
| Macieira | SC | 0 | 1.775 | 0 | 0,1 |
| Marema | SC | 0 | 1.797 | 0 | 0,0 |
| Matos Costa | SC | 0 | 2.520 | 0 | 0,1 |
| Mirim Doce | SC | 0 | 2.309 | 0 | 0,1 |
| Nova Itaberaba | SC | 0 | 4.331 | 0 | 0,0 |
| Novo Horizonte | SC | 0 | 2.442 | 0 | 0,3 |
| Ouro Verde | SC | 0 | 2.217 | 0 | 0,0 |
| Paial | SC | 0 | 1.505 | 0 | 0,0 |
| Painel | SC | 0 | 2.359 | 0 | 0,3 |
| Palma Sola | SC | 0 | 7.423 | 0 | 0,0 |
| Palmeira | SC | 0 | 2.627 | 0 | 0,0 |
| Passo de Torres | SC | 0 | 8.823 | 0 | 0,0 |
| Pedras Grandes | SC | 0 | 3.976 | 0 | 0,0 |
| Peritiba | SC | 0 | 2.787 | 0 | 0,0 |
| Pescaria Brava | SC | 0 | 10.091 | 0 | 0,0 |
| Pinheiro Preto | SC | 0 | 3.555 | 0 | 0,0 |
| Planalto Alegre | SC | 0 | 2.870 | 0 | 0,0 |
| Ponte Alta | SC | 0 | 4.682 | 0 | 0,5 |
| Ponte Alta do Norte | SC | 0 | 3.414 | 0 | 0,2 |
| Praia Grande | SC | 0 | 7.319 | 0 | 0,0 |
| Presidente Castello Branco | SC | 0 | 1.568 | 0 | 0,0 |
| Princesa | SC | 0 | 2.924 | 0 | 0,0 |
| Rancho Queimado | SC | 0 | 2.878 | 0 | 0,0 |
| Salete | SC | 0 | 7.642 | 0 | 1,8 |
| Saltinho | SC | 0 | 3.781 | 0 | 0,0 |
| Salto Veloso | SC | 0 | 4.718 | 0 | 0,0 |
| Santa Terezinha do Progresso | SC | 0 | 2.428 | 0 | 0,0 |
| Santiago do Sul | SC | 0 | 1.260 | 0 | 0,0 |
| São Bonifácio | SC | 0 | 2.838 | 0 | 0,0 |
| São Ludgero | SC | 0 | 13.410 | 0 | 0,0 |
| São Martinho | SC | 0 | 3.180 | 0 | 0,0 |
| São Miguel da Boa Vista | SC | 0 | 1.820 | 0 | 0,0 |
| Saudades | SC | 0 | 9.745 | 0 | 0,0 |
| Sul Brasil | SC | 0 | 2.461 | 0 | 0,0 |
| Timbó Grande | SC | 0 | 7.877 | 0 | 0,0 |
| Três Barras | SC | 0 | 19.275 | 0 | 0,0 |
| Treviso | SC | 0 | 3.929 | 0 | 0,0 |
| Treze Tílias | SC | 0 | 7.840 | 0 | 0,0 |
| Trombudo Central | SC | 0 | 7.360 | 0 | 0,0 |
| Vargem | SC | 0 | 2.477 | 0 | 5,9 |
| Vargem Bonita | SC | 0 | 4.492 | 0 | 2,1 |
| Xaxim | SC | 0 | 28.706 | 0 | 0,0 |
| Zortéa | SC | 0 | 3.363 | 0 | 0,3 |
| Balneário Rincão | SC | 0 | 12.760 | 0 | 0,0 |
| Aceguá | RS | 0 | 4.901 | 0 | 0,1 |
| Água Santa | RS | 0 | 3.748 | 0 | 0,0 |
| Agudo | RS | 0 | 16.461 | 0 | 0,0 |
| Ajuricaba | RS | 0 | 7.024 | 0 | 0,0 |
| Almirante Tamandaré do Sul | RS | 0 | 1.964 | 0 | 0,0 |
| Alto Alegre | RS | 0 | 1.638 | 0 | 10,8 |
| André da Rocha | RS | 0 | 1.333 | 0 | 38,0 |
| Arambaré | RS | 0 | 3.581 | 0 | 100,0 |
| Aratiba | RS | 0 | 6.235 | 0 | 0,0 |
| Arroio Grande | RS | 0 | 18.293 | 0 | 95,7 |
| Barão | RS | 0 | 6.171 | 0 | 0,0 |
| Barão de Cotegipe | RS | 0 | 6.623 | 0 | 0,0 |
| Barra do Quaraí | RS | 0 | 4.215 | 0 | 100,0 |
| Barra do Rio Azul | RS | 0 | 1.690 | 0 | 0,0 |
| Benjamin Constant do Sul | RS | 0 | 1.994 | 0 | 0,1 |
| Boa Vista das Missões | RS | 0 | 2.098 | 0 | 0,0 |
| Boa Vista do Cadeado | RS | 0 | 2.470 | 0 | 1,2 |
| Boa Vista do Incra | RS | 0 | 2.603 | 0 | 6,8 |
| Bom Princípio | RS | 0 | 14.055 | 0 | 0,0 |
| Bom Progresso | RS | 0 | 1.942 | 0 | 0,0 |
| Boqueirão do Leão | RS | 0 | 7.714 | 0 | 0,0 |
| Cambará do Sul | RS | 0 | 6.431 | 0 | 0,0 |
| Campestre da Serra | RS | 0 | 3.388 | 0 | 2,1 |
| Campina das Missões | RS | 0 | 5.474 | 0 | 0,0 |
| Canoas | RS | 0 | 346.616 | 0 | 0,0 |
| Canudos do Vale | RS | 0 | 1.716 | 0 | 0,0 |
| Capão Bonito do Sul | RS | 0 | 1.654 | 0 | 29,0 |
| Capitão | RS | 0 | 2.757 | 0 | 0,0 |
| Carlos Barbosa | RS | 0 | 29.833 | 0 | 0,0 |
| Carlos Gomes | RS | 0 | 1.377 | 0 | 0,0 |
| Casca | RS | 0 | 9.031 | 0 | 0,0 |
| Caseiros | RS | 0 | 3.202 | 0 | 1,9 |
| Cerrito | RS | 0 | 6.091 | 0 | 0,0 |
| Cerro Grande | RS | 0 | 2.311 | 0 | 0,0 |
| Chiapetta | RS | 0 | 3.756 | 0 | 0,2 |
| Chuí | RS | 0 | 6.704 | 0 | 100,0 |
| Cidreira | RS | 0 | 16.254 | 0 | 0,0 |
| Colinas | RS | 0 | 2.438 | 0 | 0,0 |
| Colorado | RS | 0 | 3.175 | 0 | 0,3 |
| Condor | RS | 0 | 6.753 | 0 | 0,0 |
| Constantina | RS | 0 | 9.911 | 0 | 0,3 |
| Coqueiro Baixo | RS | 0 | 1.501 | 0 | 0,0 |
| Cristal do Sul | RS | 0 | 2.847 | 0 | 0,0 |
| Cruzaltense | RS | 0 | 1.833 | 0 | 0,0 |
| Cruzeiro do Sul | RS | 0 | 12.348 | 0 | 0,0 |
| David Canabarro | RS | 0 | 4.740 | 0 | 17,2 |
| Dilermando de Aguiar | RS | 0 | 3.014 | 0 | 0,0 |
| Dois Lajeados | RS | 0 | 3.400 | 0 | 0,0 |
| Dom Pedrito | RS | 0 | 38.461 | 0 | 3,0 |
| Doutor Maurício Cardoso | RS | 0 | 4.549 | 0 | 0,0 |
| Doutor Ricardo | RS | 0 | 1.983 | 0 | 0,0 |
| Encantado | RS | 0 | 22.706 | 0 | 0,0 |
| Entre Rios do Sul | RS | 0 | 2.792 | 0 | 1,5 |
| Esmeralda | RS | 0 | 3.282 | 0 | 2,0 |
| Espumoso | RS | 0 | 15.588 | 0 | 11,8 |
| Estação | RS | 0 | 5.958 | 0 | 0,0 |
| Fagundes Varela | RS | 0 | 2.731 | 0 | 5,5 |
| Faxinal do Soturno | RS | 0 | 6.677 | 0 | 0,0 |
| Faxinalzinho | RS | 0 | 2.315 | 0 | 0,0 |
| Fazenda Vilanova | RS | 0 | 4.533 | 0 | 0,0 |
| Feliz | RS | 0 | 13.547 | 0 | 0,0 |
| Forquetinha | RS | 0 | 2.412 | 0 | 0,0 |
| Fortaleza dos Valos | RS | 0 | 4.320 | 0 | 84,1 |
| Gramado Xavier | RS | 0 | 4.325 | 0 | 9,7 |
| Guabiju | RS | 0 | 1.503 | 0 | 84,9 |
| Harmonia | RS | 0 | 4.866 | 0 | 0,0 |
| Herveiras | RS | 0 | 3.018 | 0 | 0,0 |
| Hulha Negra | RS | 0 | 6.776 | 0 | 0,0 |
| Ibarama | RS | 0 | 4.406 | 0 | 38,4 |
| Ibiraiaras | RS | 0 | 7.262 | 0 | 10,1 |
| Ibirubá | RS | 0 | 20.350 | 0 | 4,9 |
| Imbé | RS | 0 | 22.800 | 0 | 0,0 |
| Imigrante | RS | 0 | 3.120 | 0 | 0,0 |
| Independência | RS | 0 | 6.167 | 0 | 0,0 |
| Ipê | RS | 0 | 6.640 | 0 | 0,5 |
| Itaara | RS | 0 | 5.499 | 0 | 0,0 |
| Itaqui | RS | 0 | 37.620 | 0 | 99,6 |
| Ivorá | RS | 0 | 1.910 | 0 | 0,0 |
| Ivoti | RS | 0 | 24.293 | 0 | 0,0 |
| Jaboticaba | RS | 0 | 3.810 | 0 | 0,0 |
| Jacutinga | RS | 0 | 3.561 | 0 | 0,0 |
| Jaquirana | RS | 0 | 3.716 | 0 | 11,8 |
| Jari | RS | 0 | 3.503 | 0 | 88,4 |
| Lagoão | RS | 0 | 6.452 | 0 | 98,8 |
| Lagoa dos Três Cantos | RS | 0 | 1.609 | 0 | 0,0 |
| Liberato Salzano | RS | 0 | 5.219 | 0 | 0,8 |
| Linha Nova | RS | 0 | 1.714 | 0 | 0,0 |
| Maçambará | RS | 0 | 4.587 | 0 | 100,0 |
| Maratá | RS | 0 | 2.691 | 0 | 0,0 |
| Marcelino Ramos | RS | 0 | 4.402 | 0 | 0,0 |
| Mariano Moro | RS | 0 | 2.031 | 0 | 0,1 |
| Marques de Souza | RS | 0 | 4.009 | 0 | 0,0 |
| Mata | RS | 0 | 4.823 | 0 | 32,2 |
| Mato Castelhano | RS | 0 | 2.540 | 0 | 0,0 |
| Mato Leitão | RS | 0 | 4.515 | 0 | 0,0 |
| Montauri | RS | 0 | 1.453 | 0 | 0,0 |
| Monte Belo do Sul | RS | 0 | 2.548 | 0 | 0,0 |
| Morrinhos do Sul | RS | 0 | 2.949 | 0 | 0,0 |
| Morro Reuter | RS | 0 | 6.407 | 0 | 0,0 |
| Muçum | RS | 0 | 4.954 | 0 | 0,0 |
| Muliterno | RS | 0 | 1.893 | 0 | 0,3 |
| Não-Me-Toque | RS | 0 | 17.624 | 0 | 0,0 |
| Nonoai | RS | 0 | 11.695 | 0 | 0,0 |
| Nova Alvorada | RS | 0 | 3.625 | 0 | 0,0 |
| Nova Araçá | RS | 0 | 4.759 | 0 | 2,7 |
| Nova Bassano | RS | 0 | 9.916 | 0 | 5,5 |
| Nova Boa Vista | RS | 0 | 1.775 | 0 | 0,0 |
| Nova Esperança do Sul | RS | 0 | 5.352 | 0 | 100,0 |
| Nova Hartz | RS | 0 | 21.615 | 0 | 0,0 |
| Nova Pádua | RS | 0 | 2.553 | 0 | 0,0 |
| Nova Palma | RS | 0 | 6.512 | 0 | 2,0 |
| Nova Petrópolis | RS | 0 | 21.353 | 0 | 0,0 |
| Novo Barreiro | RS | 0 | 4.164 | 0 | 0,0 |
| Pantano Grande | RS | 0 | 9.174 | 0 | 1,2 |
| Pareci Novo | RS | 0 | 3.837 | 0 | 0,0 |
| Paulo Bento | RS | 0 | 2.293 | 0 | 0,0 |
| Pedras Altas | RS | 0 | 1.982 | 0 | 98,7 |
| Pedro Osório | RS | 0 | 7.730 | 0 | 0,8 |
| Picada Café | RS | 0 | 5.742 | 0 | 0,0 |
| Pinhal | RS | 0 | 2.579 | 0 | 0,0 |
| Pinto Bandeira | RS | 0 | 3.003 | 0 | 0,0 |
| Pirapó | RS | 0 | 2.304 | 0 | 61,1 |
| Poço das Antas | RS | 0 | 2.098 | 0 | 0,0 |
| Ponte Preta | RS | 0 | 1.547 | 0 | 0,0 |
| Porto Mauá | RS | 0 | 2.374 | 0 | 0,0 |
| Porto Vera Cruz | RS | 0 | 1.360 | 0 | 0,0 |
| Pouso Novo | RS | 0 | 1.639 | 0 | 0,0 |
| Presidente Lucena | RS | 0 | 2.901 | 0 | 0,0 |
| Protásio Alves | RS | 0 | 1.947 | 0 | 3,7 |
| Quaraí | RS | 0 | 22.687 | 0 | 98,7 |
| Quatro Irmãos | RS | 0 | 1.849 | 0 | 0,0 |
| Quevedos | RS | 0 | 2.788 | 0 | 71,1 |
| Relvado | RS | 0 | 2.090 | 0 | 0,0 |
| Restinga Seca | RS | 0 | 15.789 | 0 | 0,1 |
| Rolador | RS | 0 | 2.323 | 0 | 0,4 |
| Rondinha | RS | 0 | 5.130 | 0 | 0,0 |
| Salvador do Sul | RS | 0 | 7.799 | 0 | 0,0 |
| Santa Clara do Sul | RS | 0 | 6.603 | 0 | 0,0 |
| Santa Margarida do Sul | RS | 0 | 2.562 | 0 | 100,0 |
| Santa Tereza | RS | 0 | 1.729 | 0 | 0,0 |
| Santo Antônio do Palma | RS | 0 | 2.128 | 0 | 0,0 |
| Santo Augusto | RS | 0 | 13.885 | 0 | 0,0 |
| Santo Cristo | RS | 0 | 14.257 | 0 | 0,0 |
| Santo Expedito do Sul | RS | 0 | 2.324 | 0 | 22,2 |
| São Domingos do Sul | RS | 0 | 3.074 | 0 | 1,6 |
| São Francisco de Paula | RS | 0 | 21.710 | 0 | 3,7 |
| São João da Urtiga | RS | 0 | 4.657 | 0 | 9,4 |
| São João do Polêsine | RS | 0 | 2.552 | 0 | 0,0 |
| São Jorge | RS | 0 | 2.824 | 0 | 28,8 |
| São José das Missões | RS | 0 | 2.537 | 0 | 0,0 |
| São José do Herval | RS | 0 | 1.971 | 0 | 2,9 |
| São José do Hortêncio | RS | 0 | 4.804 | 0 | 0,0 |
| São José do Ouro | RS | 0 | 6.933 | 0 | 0,0 |
| São José do Sul | RS | 0 | 2.408 | 0 | 0,0 |
| São Marcos | RS | 0 | 21.556 | 0 | 0,0 |
| São Martinho | RS | 0 | 5.426 | 0 | 0,0 |
| São Martinho da Serra | RS | 0 | 3.234 | 0 | 0,4 |
| São Valentim do Sul | RS | 0 | 2.242 | 0 | 0,2 |
| São Vendelino | RS | 0 | 2.243 | 0 | 0,0 |
| Segredo | RS | 0 | 7.421 | 0 | 32,8 |
| Senador Salgado Filho | RS | 0 | 2.779 | 0 | 0,0 |
| Sentinela do Sul | RS | 0 | 5.581 | 0 | 99,5 |
| Serafina Corrêa | RS | 0 | 17.502 | 0 | 0,0 |
| Sério | RS | 0 | 1.962 | 0 | 0,0 |
| Sertão Santana | RS | 0 | 6.486 | 0 | 95,8 |
| Severiano de Almeida | RS | 0 | 3.657 | 0 | 0,0 |
| Silveira Martins | RS | 0 | 2.384 | 0 | 0,0 |
| Tapera | RS | 0 | 10.584 | 0 | 0,0 |
| Taquaruçu do Sul | RS | 0 | 3.072 | 0 | 0,0 |
| Terra de Areia | RS | 0 | 11.204 | 0 | 0,0 |
| Tio Hugo | RS | 0 | 3.030 | 0 | 0,0 |
| Toropi | RS | 0 | 2.806 | 0 | 27,3 |
| Tramandaí | RS | 0 | 51.715 | 0 | 0,0 |
| Travesseiro | RS | 0 | 2.336 | 0 | 0,0 |
| Três Cachoeiras | RS | 0 | 11.053 | 0 | 0,0 |
| Três Coroas | RS | 0 | 28.220 | 0 | 0,5 |
| Três Palmeiras | RS | 0 | 4.271 | 0 | 0,6 |
| Tucunduva | RS | 0 | 5.678 | 0 | 0,0 |
| Tunas | RS | 0 | 4.569 | 0 | 100,0 |
| Tupanci do Sul | RS | 0 | 1.472 | 0 | 1,7 |
| Tupanciretã | RS | 0 | 23.948 | 0 | 10,4 |
| Tupandi | RS | 0 | 4.855 | 0 | 0,0 |
| Vale do Sol | RS | 0 | 11.781 | 0 | 0,6 |
| Vale Real | RS | 0 | 5.913 | 0 | 0,0 |
| Vanini | RS | 0 | 2.113 | 0 | 6,3 |
| Vespasiano Correa | RS | 0 | 1.815 | 0 | 0,0 |
| Viadutos | RS | 0 | 4.756 | 0 | 0,0 |
| Victor Graeff | RS | 0 | 2.882 | 0 | 0,0 |
| Vila Flores | RS | 0 | 3.385 | 0 | 0,8 |
| Vila Lângaro | RS | 0 | 2.091 | 0 | 0,0 |
| Vila Nova do Sul | RS | 0 | 4.280 | 0 | 99,4 |
| Vista Alegre do Prata | RS | 0 | 1.561 | 0 | 9,7 |
| Vista Gaúcha | RS | 0 | 2.851 | 0 | 0,0 |
| Vitória das Missões | RS | 0 | 3.133 | 0 | 0,0 |
| Xangri-lá | RS | 0 | 16.408 | 0 | 0,0 |
| Brasilândia | MS | 0 | 11.872 | 0 | 9,8 |
| Corguinho | MS | 0 | 5.947 | 0 | 31,0 |
| Glória de Dourados | MS | 0 | 9.965 | 0 | 0,0 |
| Iguatemi | MS | 0 | 16.078 | 0 | 1,5 |
| Japorã | MS | 0 | 9.110 | 0 | 0,0 |
| Jateí | MS | 0 | 4.027 | 0 | 1,9 |
| Novo Horizonte do Sul | MS | 0 | 3.814 | 0 | 1,0 |
| Rio Negro | MS | 0 | 4.831 | 0 | 1,1 |
| Vicentina | MS | 0 | 6.102 | 0 | 0,0 |
| Novo Horizonte do Norte | MT | 0 | 4.004 | 0 | 0,6 |
| Ponte Branca | MT | 0 | 1.576 | 0 | 0,7 |
| Ribeirãozinho | MT | 0 | 2.405 | 0 | 0,1 |
| Rio Branco | MT | 0 | 5.156 | 0 | 0,6 |
| Rondolândia | MT | 0 | 4.001 | 0 | 26,3 |
| Serra Nova Dourada | MT | 0 | 1.650 | 0 | 18,8 |
| Nova Guarita | MT | 0 | 4.519 | 0 | 7,9 |
| Água Limpa | GO | 0 | 1.850 | 0 | 2,5 |
| Aloândia | GO | 0 | 1.995 | 0 | 0,0 |
| Anhanguera | GO | 0 | 1.149 | 0 | 0,0 |
| Aporé | GO | 0 | 4.198 | 0 | 8,7 |
| Araçu | GO | 0 | 3.522 | 0 | 0,0 |
| Aurilândia | GO | 0 | 3.120 | 0 | 0,2 |
| Avelinópolis | GO | 0 | 2.417 | 0 | 0,0 |
| Barro Alto | GO | 0 | 11.167 | 0 | 2,0 |
| Bonfinópolis | GO | 0 | 9.706 | 0 | 0,0 |
| Buriti de Goiás | GO | 0 | 2.488 | 0 | 0,0 |
| Cachoeira Dourada | GO | 0 | 8.067 | 0 | 0,0 |
| Campestre de Goías | GO | 0 | 3.630 | 0 | 1,0 |
| Campos Verdes | GO | 0 | 2.141 | 0 | 0,9 |
| Castelândia | GO | 0 | 3.435 | 0 | 0,1 |
| Chapadão do Céu | GO | 0 | 10.167 | 0 | 3,0 |
| Córrego do Ouro | GO | 0 | 2.327 | 0 | 0,1 |
| Cumari | GO | 0 | 2.854 | 0 | 1,1 |
| Damolândia | GO | 0 | 2.938 | 0 | 0,0 |
| Edealina | GO | 0 | 3.699 | 0 | 2,5 |
| Fazenda Nova | GO | 0 | 5.637 | 0 | 1,6 |
| Firminópolis | GO | 0 | 13.292 | 0 | 0,0 |
| Formoso | GO | 0 | 4.248 | 0 | 0,0 |
| Gouvelândia | GO | 0 | 5.898 | 0 | 9,4 |
| Hidrolina | GO | 0 | 3.564 | 0 | 0,5 |
| Israelândia | GO | 0 | 2.800 | 0 | 0,5 |
| Itauçu | GO | 0 | 8.938 | 0 | 0,0 |
| Jandaia | GO | 0 | 6.048 | 0 | 0,0 |
| Lagoa Santa | GO | 0 | 1.588 | 0 | 3,9 |
| Marzagão | GO | 0 | 2.236 | 0 | 0,0 |
| Maurilândia | GO | 0 | 14.080 | 0 | 0,0 |
| Moiporá | GO | 0 | 1.529 | 0 | 0,0 |
| Nova América | GO | 0 | 2.352 | 0 | 0,0 |
| Nova Aurora | GO | 0 | 2.210 | 0 | 0,9 |
| Nova Glória | GO | 0 | 8.164 | 0 | 0,0 |
| Ouvidor | GO | 0 | 6.667 | 0 | 0,7 |
| Palestina de Goiás | GO | 0 | 3.464 | 0 | 2,8 |
| Porteirão | GO | 0 | 3.881 | 0 | 0,0 |
| Rialma | GO | 0 | 10.918 | 0 | 0,0 |
| Rianápolis | GO | 0 | 4.801 | 0 | 0,0 |
| Rio Quente | GO | 0 | 4.493 | 0 | 0,0 |
| Sanclerlândia | GO | 0 | 7.637 | 0 | 0,0 |
| Santo Antônio da Barra | GO | 0 | 4.821 | 0 | 1,7 |
| Santo Antônio de Goiás | GO | 0 | 6.283 | 0 | 0,0 |
| São Francisco de Goiás | GO | 0 | 6.264 | 0 | 0,0 |
| São Patrício | GO | 0 | 2.036 | 0 | 0,0 |
| Turvânia | GO | 0 | 4.598 | 0 | 0,0 |
| Turvelândia | GO | 0 | 5.281 | 0 | 0,2 |
| Note: Own elaboration. Source: SINAN/DATASUS and IBGE | | | |  |  |
