# Supplementary material for: Geographical accessibility to the supply of antiophidic sera in Brazil: Timely access possibilities
Source: PLoS One. 2022 Jan 13;17(1):e0260326. doi: 10.1371/journal.pone.0260326 (PMC8757981; doi:10.1371/journal.pone.0260326)
Supplement: Attachment — Submitted filename: Review October1st.pdf [file pone.0260326.s003.pdf]

Abstract

- Methods must describe: the type of study design.

Methods

- Data analysis: it is necessary to describe, as well as the association measures which were used, the statistical tests and the level of significance considered.
