# Supplementary material for: Geographical accessibility to the supply of antiophidic sera in Brazil: Timely access possibilities
Source: PLoS One. 2022 Jan 13;17(1):e0260326. doi: 10.1371/journal.pone.0260326 (PMC8757981; doi:10.1371/journal.pone.0260326)
Supplement: Attachment — Submitted filename: Response to Reviewers.docx [file pone.0260326.s004.docx]

Rio de Janeiro, October 21^st^, 2021

Rebuttal Letter

Dear editors and reviewers,

Thank you for the revision and suggestions to improve our manuscript. In order to resubmit it, we answer the points that the academic editor and one of the reviewers raised about our work. First, we respond to the Journal Requirements and then to the requests and suggestions, putting the points in italic and our answers just below.

**Journal Requirements**

*Please review your reference list to ensure that it is complete and correct. If you have cited papers that have been retracted, please include the rationale for doing so in the manuscript text, or remove these references and replace them with relevant current references. Any changes to the reference list should be mentioned in the rebuttal letter that accompanies your revised manuscript. If you need to cite a retracted article, indicate the article’s retracted status in the References list and also include a citation and full reference for the retraction notice.*

The reference list was reviewed in order to correct it, considering the reference style of International Committee of Medical Journal Editors (ICMJE). As a new reference was included, the reference indications were changed in the text and also the reference list.

**Review Comments to the Author**

*Reviewer #1: Abstract*

*Methods must describe: the type of study design.*

The abstract was updated considering the request, adding the type of study design.

*Methods*

*Data analysis: it is necessary to describe, as well as the association measures which were used, the statistical tests and the level of significance considered.*

In order to improve the information about the travelling time calculation, the first paragraph of the Methods section was changed. Also, a new reference was included, to better characterize the algorithm considered in ACCESSMOD 5.0, the software which was used to estimate traveling time to health facilities. The Distance and time to reach health facilities calculation subsection was altered in order to include this information.

There is no other information about the association measures which were used, the statistical tests and the level of significance considered, because we do not use specific statistical analysis and even other authors that use the software do not bring those details. But the referred new article can provide a more detailed information about the travel time cost surface model.

Dijkstra, E.W. A note on two problems in connexion with graphs. Numer. Math. 1959;1: 269–271. <https://doi.org/10.1007/BF01386390>

Kind regards,

Ricardo Antunes Dantas de Oliveira, Diego Ricardo Xavier Silva and Maurício Gonçalves e Silva
